# Efficient symptomatic treatment and viral load reduction for children with influenza virus infection by nasal-spraying *Bacillus* spore probiotics

**DOI:** 10.1038/s41598-023-41763-5

**Published:** 2023-09-08

**Authors:** Tu Thanh Tran, Thuy Thi Bich Phung, Dien Minh Tran, Huyen Thi Bui, Phuc Thanh Thi Nguyen, Tam Thi Vu, Nga Thi Phuong Ngo, Mai Thi Nguyen, Anh Hoa Nguyen, Anh Thi Van Nguyen

**Affiliations:** 1 International Center, Vietnam National Children’s Hospital, No. 18/879 La Thanh, Dong Da, Hanoi, Vietnam; 2 Department of Molecular Biology for Infectious Diseases, Vietnam National Children’s Hospital, No. 18/879 La Thanh, Dong Da, Hanoi, Vietnam; 3 Department of Surgical Intensive Care Unit, Vietnam National Children’s Hospital, No. 18/879 La Thanh, Dong Da, Hanoi, Vietnam; 4 Key Laboratory of Enzyme and Protein Technology, VNU University of Sciences, Vietnam National University, Hanoi, 334 Nguyen Trai, Thanh Xuan, Hanoi, Vietnam; 5 Spobiotic Research Center, ANABIO R&D Ltd. Company, No. 22, Lot 7, 8 Van Khe Urban, La Khe, Ha Dong, Hanoi, Vietnam; 6 LiveSpo Pharma Ltd. Company, N03T5, Ngoai Giao Doan Urban, Bac Tu Liem, Hanoi, Vietnam

**Keywords:** Influenza virus, Randomized controlled trials, Paediatric research, Clinical microbiology

## Abstract

Influenza virus is a main cause of acute respiratory tract infections (ARTIs) in children. This is the first double-blind, randomized, and controlled clinical trial examining the efficacy of nasal-spraying probiotic LiveSpo Navax, which contains 5 billion of *Bacillus subtilis* and *B. clausii* spores in 5 mL, in supporting treatment of influenza viral infection in pediatric patients. We found that the nasal-spraying *Bacillus* spores significantly shortened the recovery period and overall treatment by 2 days and increased treatment effectiveness by 58% in resolving all ARTIs’ symptoms. At day 2, the concentrations of influenza virus and co-infected bacteria were reduced by 417 and 1152 folds. Additionally, the levels of pro-inflammatory cytokines IL-8, TNF-α, and IL-6 in nasopharyngeal samples were reduced by 1.1, 3.7, and 53.9 folds, respectively. Compared to the standard control group, treatment regimen with LiveSpo Navax demonstrated significantly greater effectiveness, resulting in 26-fold reduction in viral load, 65-fold reduction in bacterial concentration, and 1.1–9.5-fold decrease in cytokine levels. Overall, nasal-spraying *Bacillus* spores can support the symptomatic treatment of influenza virus-induced ARTIs quickly, efficiently and could be used as a cost-effective supportive treatment for respiratory viral infection in general.

**Clinical trial registration no**: NCT05378022 on 17/05/2022.

## Introduction

Influenza A and B are the two major types that cause seasonal flu epidemics, in particular, Acute Respiratory Tract Infections (ARTIs) in young children and infants. Although influenza infection initially causes uncomplicated symptoms such as fever, cough, sore throat, runny nose, ect, it has also been associated with other serious complications such as pneumonia, bronchitis, and respiratory failure^1–5^. According to a systematic review and meta-analysis of the global burden of seasonal influenza respiratory infections in young children, about 28,000–111,500 children under the age of 5 died as a consequence of influenza infections in 2008, of which 99% of these deaths occurred in developing countries^6^. Interleukin-6 (IL-6), IL-8, and tumor necrosis factor-α (TNF-α) are potential biomarkers for influenza infection because elevated levels of these pro-inflammatory cytokines in both bronchial epithelial cells and clinical patients' nasopharyngeal lavage fluid correlates with viral loads and scores of typical influenza symptoms^7–10^.

Influenza vaccination requires annual injections, which made it difficult for young children to get effectively immunized^11^. Moreover, antiviral nucleotide drugs including Tamiflu (oseltamivir) are unsafe for young children and is recommended only for high-risk patients^12–15^. To avoid bacterial co-infection and worsening symptoms, treatment for pediatric influenza infection focuses on reducing fever, improving nutrition, and cleansing the nasal airway. Antibiotics are only prescribed when there are co-infecting pathogens, such as *Streptococcus pneumoniae*, *Haemophilus influenzae,* and *Moraxella catarrhalis*^16–18^. Therefore, preventing co-infection will also reduce the wide-spread use of antibiotics.

Probiotics have recently emerged as promising safe candidates for supportive treatment of ARTIs, which can help reducing antibiotic dependence^19^. A number of clinical trials have shown that *Lactobacillus rhamnosus* GG, *Bacillus coagulans* GBI-30, and *Bifidobacterium animalis ssp. lactis* Bb12 reduce the symptoms and prevent respiratory tract infections by modulating proinflammatory cytokines including IL-6, IL-8, TNF-α while stimulating secretion of Immunoglobulin A (IgA) by mucosal lymphocytes^20–22^. However, no evidence is yet available to support the use of those probiotics in lowering the occurrence and viral loads in the nasal tract^20,23–25^. Furthermore, oral digested probiotics do not provide immediate benefits, hence are typically used as preventive care of ARTIs. As a result, alternate delivery routes are needed for the use of probiotics in treatment of ARTIs. Our recent study demonstrated that nasal-spraying *Bacillus* spore liquid-form probiotics (LiveSpo Navax) can rapidly and effectively relieve symptoms of ARTIs due to respiratory syncytial virus (RSV) infection and exhibit strong impacts in reducing the viral load and inflammation. The finding established that administering probiotics through nasal spray could be a quick and effective symptomatic treatment for ARTIs^26^. Because the mechanism of interaction between *Bacillus* spores and virus is non-specific, our findings suggested that nasal-spraying *Bacillus* spores may also be effective toward other rapidly emerging RTIs viruses such as influenza virus. In the following study, we further conducted the double-blind, randomized, and controlled clinical trial examining the efficacy of LiveSpo Navax in supporting treatment of young children with acute respiratory symptoms due to influenza infection. The results demonstrated strong efficacy of LiveSpo Navax in reducing infection symptoms and supporting rapid recovery of patients.

## Results

### Trial design and participants’ baseline characteristics

**Figure 1:**
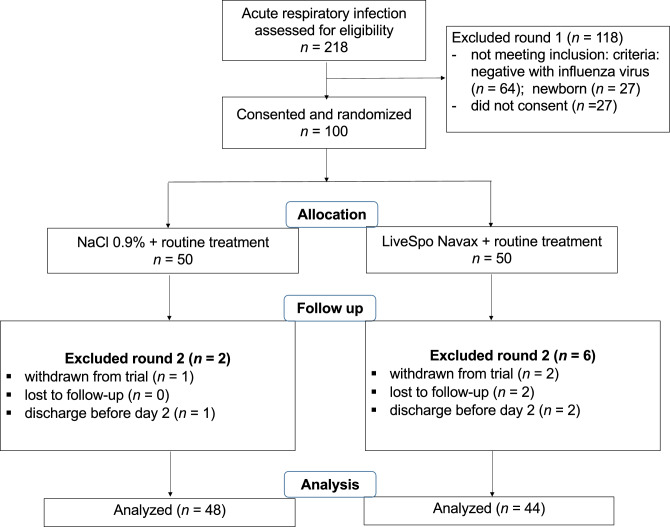
Diagram showing the NCT05378022 clinical trial design and data analysis from December 2020 to April 2022.

The trial was conducted from December 2020 to April 2022, starting with a cohort of 100 patients equally assigned into two groups: the control group who received standard care, and the Navax group who received LiveSpo Navax in addition to standard care. Each group received 3 sprays of either control 0.9% NaCl or LiveSpo Navax daily. At the end of treatment period, 48 patients in the control group and 44 patients in the Navax group were included in final analysis (Fig. 1).

Patients’ demographic, clinical and sub-clinical characteristics were shown in Table 1. There is no significant difference in (i) age, (ii) gender distribution, and (iii) admitted day between groups (*p* > 0.05). Baseline clinical and subclinical characteristics including runny nose, dry rales, moist rales, body temperature (°C), pulse oxymetry (SpO_2_) (%), pulse and breath (beats/min), influenza viral load and bacterial co-infection measured by real-time qPCR in nasopharyngeal samples, cardiopulmonary X-ray, total white blood cells, CRP were not substantially different between two groups before treatment (*p* > 0.05) (Table 2).

**Table 1:** Demographic and clinical characteristics of influenza-infected children before, during and at the end of treatment.

Characteristic	Control group (N = 48)			Navax group (N = 44)			*p *value		
	Before treatment	After treatment		Before treatment	After treatment		Before treatment	After treatment	
	Day 0	Day 2	Day 5	Day 0	Day 2	Day 5	Day 0	Day 2	Day 5
Age (months)									
≥ 4–12, *n* (%)	9 (18.75)			7 (15.91)			0.72		
> 12–84, *n* (%)	39 (81.25)			37 (84.09)					
Gender									
Male, *n* (%)	25 (52.08)			24 (54.55)			0.81		
Female, *n* (%)	23 (47.92)			20 (45.45)					
Days of sickness before treatment	3.1			2.9			0.57		
Typical symptoms due to influenza infection n (%)									
Runny nose,* n* (%)	48 (100)	48 (100)	12 (25.00)	44 (100)	30 (68.18)	0 (0.00)	0.97	0.01	0.02
Dry rales, *n* (%)	33 (68.75)	32 (66.67)	10 (20.83)	22 (50.00)	18 (40.91)	0 (0.00)	0.07	0.01	0.03
Moist rales *n* (%)	21 (43.75)	21 (43.75)	6 (12.50)	13 (39.55)	12 (27.27)	0 (0.00)	0.16	0.10	0.08
Temperature (℃)									
Normal (≤ 37.5), *n* (%)	4 (8.33)	13 (27.08)	48 (100)	3 (6.82)	31 (70.45)	44 (100)	0.78	0.0001	0.97
Fever (> 37.5), *n* (%)	44 (91.67)	35 (72.92)	0 (0.00)	41 (93.18)	13 (29.55)	0 (0.00)			
SpO_2_ (%)									
≥ 95%, *n* (%)	48(100)	48(100)	48(100)	44 (100)	44 (100)	44 (100)	0.97	0.97	0.97
< 95%, *n* (%)	0 (0.00)	0 (0.00)	0 (0.00)	0 (0.00)	0 (0.00)	0 (0.00)			
Pulse (beats/min)									
≥ 4–12 months (> 140), *n* (%)	3 (6.82)	3 (6.25)	0 (0.00)	3 (6.82)	2 (4.55)	0 (0.00)	0.91	0.72	–^a^
> 12 months (> 120), *n* (%)	28 (58.33)	26 (54.17)	0 (0.00)	25 (56.82)	16 (36.36)	0 (0.00)	0.88	0.09	–^a^
Breath (beats/min)									
≥ 4–12 months (> 40), *n* (%)	2 (4.17)	1 (2.08)	0 (0.00)	1 (2.27)	0 (0.00)	0 (0.00)	0.61	0.53	–^a^
> 12 months (> 32), *n* (%)	20 (41.67)	11 (22.92)	0 (0.00)	12 (27.27)	6 (13.64)	0 (0.00)	0.15	0.26	–^a^

–^a^: There is no remained patients to compare the significance.

**Table 2:** Sub-clinical characteristics of influenza-infected children before treatment and treatment therapy.

Characteristic	Control group (N = 48)		Navax group (N = 44)			*p *value
	Total, *n* (%)	Min–max	Total, *n* (%)	Min–max		
Cardiopulmonary X-ray						
Osler’s nodes	32 (66.67)		25 (56.82)			0.33
Hyperinflation	2 (4.17)		0 (0.00)			0.32
Osler’s nodes and hyperinflation	3 (6.25)		5 (11.36)			0.39
Normal	11 (22.92)		14 (31.82)			0.34
Influenza positive						
Rapid test	48 (100)		44 (100)			0.97
Real-time PCR (C_t_)	48 (100)	17.61–32.57	44 (100)	15.55–29.00		0.97
Hematology and biochemistry						
Total white blood cells (G/L)		2.86–18.20		5.71–25.46		
< 6.0	4 (8.34)		2 (4.55)			0.47
6.0–10.0 (G/L)	22 (45.83)		27 (61.36)			0.20
> 10.0 (G/L)	22 (45.83)		15 (34.09)			0.19
Total red blood cells (T/L)		3.64–6.13		3.66 – 6.14		
< 3.0 (T/L)	0 (0.00)		0 (0.00)			–^a^
3.0–5.0 (T/L)	41 (85.42)		34 (77.27)			0.32
> 5.0 (T/L)	7 (14.58)		10 (22.73)			0.32
Total platelet count (G/L)		166–801		176–468		
< 140 (G/L)	0 (0.00)		0 (0.00)			–^a^
140–440 (G/L)	43 (89.58)		40 (90.91)			0.83
> 440 (G/L)	5 (10.42)		4 (9.09)			0.83
CRP (mg/L)		0.08–111.46		0.46–41. 27		
≤ 6.0	33 (68.75)		26 (59.09)			0.34
> 6.0	15 (31.25)		18 (40.91)			
Bacterial co-infection	15 (31.25)	22–30	22 (50.00)	20–30		0.07
*H. influenzae (HI)*	9 (18.75)		13 (29.55)			0.23
*S. pneumoniae (SP)*	4 (8.33)		7 (15.91)			0.27
*H. influenzae & S. pneumoniae (HI-SP)*	2 (4.17)		0 (0.00)			0.32
*M. catarrhalis (MC)*	0 (0.00)		2 (4.55)			0.32
Treatment therapy						
Routine treatment	- Oral administrative drugs: antipyretic paracetamol (Efferegant); expectorant Carbocysteine (Carbothiol); antiviral Oseltamivir phosphate (Tamiflu); antibiotics e.g. cefotaxim (Goldcefo), Amoxicillin/acid clavulanic (Augmentin) based on the results of antibiotic susceptibility test - Aerosol therapy: bronchodilator e.g. salbutamol (Ventolin inhaler) or budesonide (Pulmicort Respules)					
Nasal-spraying treatment	NaCl 0.9%	NaCl 0.9% plus *B. subtilis* and *B. clausii* at 5 billion CFU/5 mL (LiveSpo Navax)				

–^a^: There is no remained patients to compare the significance.

### Safety and symptomatic-reducing effects of nasal-spraying *Bacillus* spores

**Figure 2:**
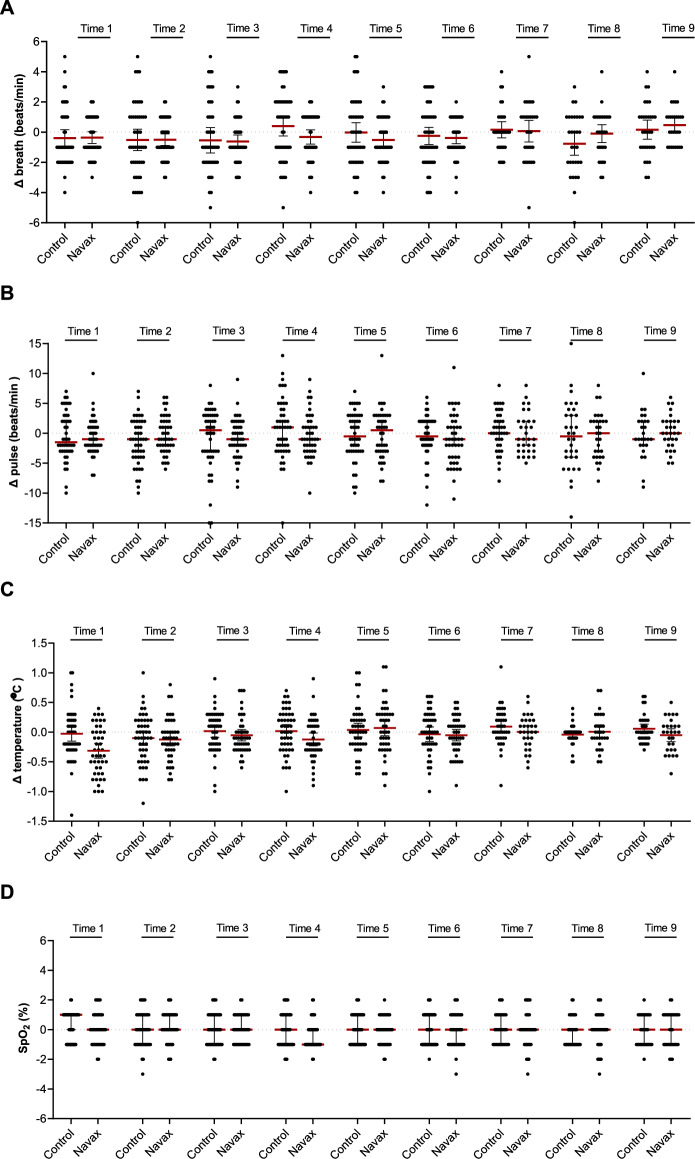
Clinical assessment of breath (**A**), pulse (**B**), temperature (**C**) and pulse oxygen (**D**) between before and after nasal-spraying with LiveSpo Navax (in diagonal-strip pattern) and 0.9% NaCl physiological saline (in white pattern), across 9 spraying times over 3 days. Graphs showing median values (red lines), lower-upper confidence limits (black lines), and measurements from individual patients (dots).

During 5-day treatment, no abnormal changes in breathing, pulse, body temperature, and pulse oxymetry were observed upon spraying *Bacillus* spores. The four clinical indicators were recorded before and after spraying of LiveSpo Navax or 0.9% NaCl 3 times a day for the first 3 days of treatment. As shown in Fig. 2, breath, pulse, temperature, and SpO_2_ values of both groups fluctuated within only 1 and 3 beats/min, 0.1 °C, 0.5%, respectively. All participants in both groups had no symptom of local bacterial infection, or had any digestive problems such as vomiting, diarrhea and no abnormality in vital signs after the spraying procedure. The results showed that administering *Bacillus* spores through nasal-spraying is safe for pediatric influenza patients during the treatment period.

**Figure 3:**
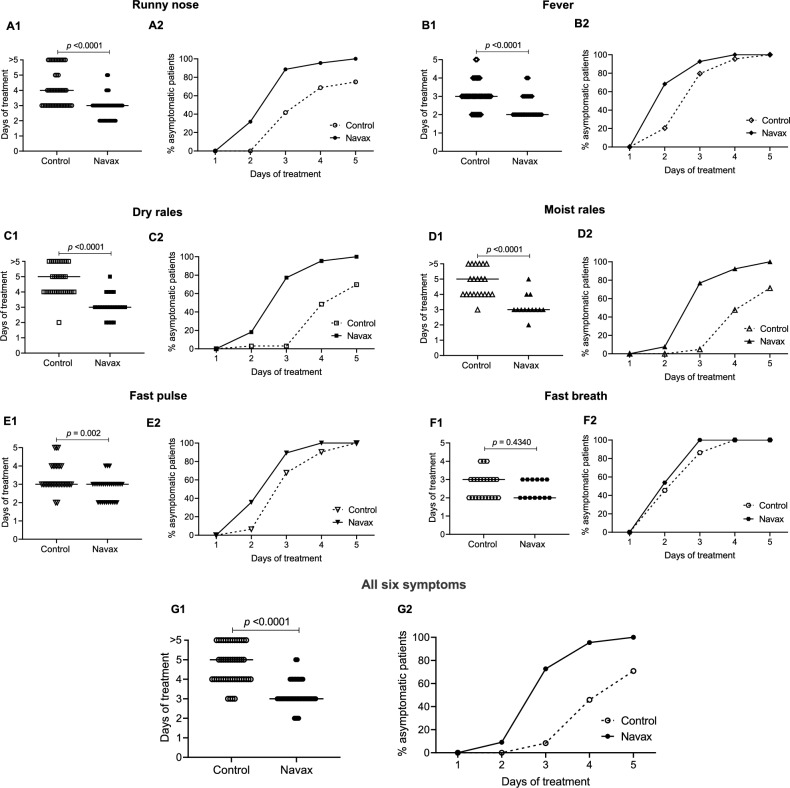
Days of treatment and percentage (%) of asymptomatic patients observed over days of treatment for symptoms: (**A**) runny nose; (**B**) fever; (**C**) dry rales; (**D**) moist rales; (**E**) fast pulse; (**F**) fast breath; and (**G**) all six symptoms in the Control (dashed lines) and Navax (solid lines) groups. More than five days of treatment refers to symptoms that were not resolved by day 5. Mann–Whitney test was performed to evaluate statistical significance.

We next assessed typical clinical signs of influenza infection in patients at days 2 and 5 to evaluate efficacy of *Bacillus* spores. At day 2, the Navax group had a statistically significant lower percentage of patients with several symptoms, including runny nose (*p* = 0.01), fever (*p* = 0.0001), dry rales (*p* = 0.01) compared to the Control group (Table 1). At day 5, there was no patient with runny nose (*p* = 0.02) and dry rales (*p* = 0.03) in Navax group, whereas in the Control group, there were still 10 out of 48 patients exhibiting runny nose symptoms and 12 out of 33 patients showing dry rales symptoms (Table 1). Because most patients had recovered by day 2, there was no statistically significant difference between the two groups in terms of pulse oximetry or fast breath (Table 1). The median days of treatment (defined as the first day during the course of treatment when a patient no longer exhibits a particular symptom) and time-course dependent percentage (%) of patients with symptom-free status in the two groups were then analyzed and shown in Fig. 3A1–F1 and A2–F2. Patients in the Navax group experienced remarkably faster recovery, with relief from runny nose observed 1 day earlier, fever subsiding 1 day earlier, and dry and moist rales alleviating 2 days earlier compared to the Control group (Fig. 3A1–D1, *p* < 0.0001). Although fast breath was also recovered a day earlier with Navax treatment, the difference between the two groups was not statistically significant (*p* = 0.4340; Fig. 3F1). The recovery time of the fast pulse symptom was similar in both groups (Fig. 3E1). Furthermore, the data shown in Fig. 3G1 demonstrated that LiveSpo Navax treatment resulted in a 2-day faster improvement (*p* < 0.0001) for all six clinical symptoms of influenza infection in pediatric patients.

We also noted that the Days of Treatment when 50% patients are no longer symptomatic (DT_50_) for each symptoms as well as DT_50_ for all six symptoms in the Navax group were shorter compared to those of the Control group. Specifically, Navax treatment reduced DT_50_ for runny nose, fever, dry rales, moist rales, fast pulse, fast breath, and DT_50_ for all six symptoms by 40%, 50%, 60%, 60%, 20%, 10%, and 58%, respectively (Fig. 3A2–G2). Overall, nasal-spraying *Bacillus* spores alleviated major influenza infection symptoms 1 to 2-day earlier and with 10–60% greater effectiveness. As a result, the overall treatment duration was reduced by 2 days, and the treatment effectiveness for complete symptomatic recovery increased by 58%.

### Reduction in influenza viral load and concentration of co-infecting bacteria by nasal-spraying *Bacillus* spores

**Figure 4:**
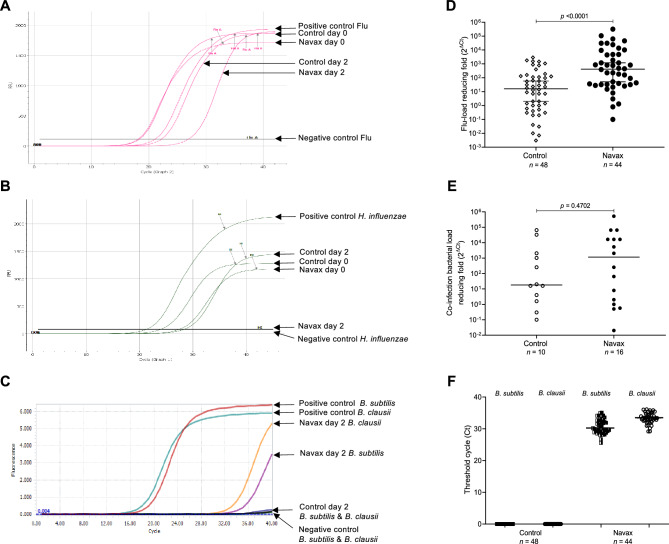
Real-time PCR amplification curves specifically for influenza virus (**A**), co-infection *H. influenzae* (**B**), and *B. subtilis* + *B. clausii* (**C**) taken from representative nasopharyngeal sample of Control and Navax groups at day 0 and 2 of treatment; Reducing-fold levels (2^△Ct^) of influenza viral load (**D**) and concentration of co-infecting bacteria (**E**), and Threshold cycle (C_t_) of fluorescent signals for *B. subtilis* and *B. clausii* (**F**) measured in nasopharyngeal samples of Control and Navax groups at day 2 compared to day 0. The Mann–Whitney test was used to calculate the median difference of these indicators in the two groups. 95% CI for median in each group and the median difference between the two groups were shown in Fig. 4D,F. The significance level of all analyzes was set at the *p* < 0.05.

To determine how *Bacillus* spores act to relieve symptoms, we measured 2^△Ct^ values, which represent the fold changes in influenza viral loads and concentration of bacterial co-infection in nasopharyngeal samples. Assessments were performed on day 2, during which we observed significant benefit of Navax treatment in improving symptoms of influenza-infected patients. As shown in Fig. 4A,B, representative amplification curves measuring the viral load and concentration of bacterial co-infection (*S. pneumoniae* and *H. influenzae*) showed significantly lower (or not detectable) presence of viruses and bacteria at day 2 in both in Control and Navax groups. However, amplification curves in Navax shifted significantly to the higher or even non-detectable range than those in Control group, indicating that Navax more effectively reduced both influenza viral load and *H. influenzae* concentration. Similar results were observed in other samples of both groups (Fig. S1). Furthermore, the 2^△Ct^ data revealed that influenza viral load in Navax group was reduced by 417-fold, which was nearly 26-fold more effective than the 16-fold reduction in Control group (*p* < 0.0001, Fig. 4D). Measurement of average change in bacterial co-infection showed that *H. influenzae* and/or *S. pneumoniae* concentration in the Navax group was reduced by nearly 65-fold whereas 18-fold reduction was observed in the Control group. However, due to the wide range of variables in bacterial concentration, the statistical analysis did not yield a significant result (*p* = 0.4702; Fig. 4E). We also measured *B. subtilis* and *B. clausii* in nasopharyngeal samples at day 2 to ensure that probiotics or 0.9% NaCl being used properly. There were strong signals in the Navax group (median C_t_ value of 30.18 and 33.34) and non-detectable signals in the Control group for *B. subtilis* and *B. clausii* (Fig. 4C,F), confirming that the patients were taking the correct medication. Altogether, we showed that nasal-spraying *Bacillus* spores increased the efficacy of standard medication therapy by substantially lowering influenza viral load.

### Immune-modulatory properties of nasal-spraying *Bacillus* spores

**Figure 5:**
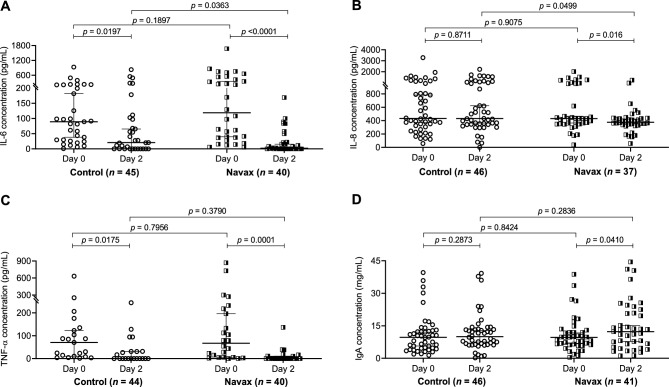
Pro-inflammatory cytokines levels (pg/mL) (**A–C**) and IgA level (mg/mL) (**D**) in nasopharyngeal samples of Control and Navax groups at day 2 compared to day 0. The Wilcoxon test was used to calculate the median differences in IL-6, IL-8, TNF-α, and IgA levels at day 0 and day 2 in each group. The Mann–Whitney test was used to compared cytokine concentrations between the two groups. Only samples with measurable cytokine and IgA concentrations at day 0 were included in the statistical analysis. 95% CI for median in each group and the median difference between the two groups were shown. The significance level of all analyzes was set at the *p* < 0.05.

Next, we examined how levels of common pro-inflammatory cytokines including IL-6, IL-8, and TNF-α, as well as levels of mucosal Immunoglobulin A (IgA) in nasopharyngeal samples were altered over the course of treatment. We anticipated that sprayed *Bacillus* spores can compete with influenza virus for interactions with nasal epithelium, resulting in a reduced systemic inflammatory response. At day 0, the levels of IL-6 and IL-8, and TNF-α in both groups were high and generally comparable (*p* > 0.05). After 2 days of Navax treatment, we observed that IL-6 level was markedly reduced by 53.9 folds (*p* < 0.0001) (Fig. 5A). Both IL-8 and TNF-α levels showed significant decrease by 1.1 (*p* = 0.016), 3.7 (*p* = 0.0001) fold, respectively (Fig. 5B,C). In Control group, although there was significant reduction in IL-6 (*p* = 0.0197) and TNF-α (*p* = 0.0175) levels, no change in IL-8 levels (*p* = 0.8711) was detected after 2 days of treatment. While TNF-α levels were effectively reduced to zero in both groups (Fig. 5C), IL-6, IL-8 levels exhibited 9.5-fold (*p* = 0.0363) and 1.1-fold (*p* = 0.0499) greater reduction upon Navax treatment (Fig. 5A,B).

Furthermore, we evaluated how spraying *Bacillus* spores influences IgA levels in nasopharyngeal. We found that there was slight induction of IgA in both Control and Navax groups (Fig. 5D). However, the 1.3-fold increase in IgA level observed in Navax group is more robust, resulting in statistically significant difference between day 0 vs. day 2 (*p* = 0.0410). The data indicated that nasal-spraying *Bacillus* spores significantly decreased IL-6, IL-8, and TNF-α cytokine overreacted production, and to some extend increase nasal IgA level in immunological responses to influenza infection.

## Discussion

The significant prevalence of respiratory viral infections emphasizes the importance of a safe, effective, and inexpensive antiviral treatment for vulnerable patients like young children. Probiotic therapy has recently emerged as an exciting avenue supported by encouraging results from clinical trials during which the use of probiotic therapy in foods can prevent respiratory tract infections (RTIs). *Bacillus* probiotics can maintain their efficacy for a long time due to their ability to form heat-stable spores under starvation^27–29^. Currently, only *B. subtilis* and *B. clausii* probiotics can be made as ultra-pure, concentrated spore liquid-formulation for direct nasal-spraying. In this reported clinical trial, the data showed that LiveSpo Navax was safe for all of the participants, providing 1–2-day earlier and 10–60% more effective in resolution of symptoms. Notably, after 5-day treatment, while 12.5–25% patients in Control group still experienced runny nose and dry rales, all patients in Navax group were fully recovered. This data is consistent with our recent study in pediatric RSV infected patients^26^, further reinforcing our hypothesis that the nasal-spraying *Bacillus* spores non-specifically interact with nasal mucosal immune system, virus, and bacteria, thereby can support treatment of rapidly emerging RTIs virus in general such as RSV, influenza virus. This may also be applicable for adenovirus, rhinovirus, and coronavirus, etc. When compared to other recent trials of orally digested probiotics, our results showed a much faster response (days vs. months) and stronger efficacy (hundreds folds more effective than standard care) when used as a supportive therapy of ARTIs. In previously reported clinical trials in young children using oral administrative probiotics, a slight improvement in symptomatic relief was observed only after a 3-month regimen of *B. coagulans* GBI-30 6086 probiotics^21^. No significant reduction in the incidence of respiratory infection was documented with 3.5-month usage of *B. subtilis* DE111 probiotics^30^.

The sprayed *Bacillus* spores can provide an additional barrier of protection to help increase the effectiveness of preventing respiratory viral infection, especially in immunized cases having low in-situ antibody levels in the nasal tract or appearance of new immunity-escape mutations. We showed that LiveSpo Navax therapy were 26-fold more effective than standard-of-care therapy in reducing influenza viral load. These findings are agreeable with the previous *in-vitro* and animal study reporting that one *B. subtilis* spores can non-specifically adsorb eight H5N1 influenza virions and that the virus-spore complexes can function as a nasal-drop vaccine with 80% protection of mice against H5N1 viral challenge infection at 5LD_50_ dosage^31^. Hong et al.^32^ have also shown that nasal drops of *B. subtilis* spores lowered influenza level by 4–5 folds in mice by improving the antiviral function of alveolar macrophages^32^. Co-infections caused by *S. pneumoniae* and *H. influenza* worsen the respiratory failure, lengthen the treatment period, raise the cost of antibiotic treatment and further cause antibiotic-resistance^33^. Our data showed that at day 2 of nasal-spraying *Bacillus* spores, the concentration of *H. influenzae* and/or *S. pneumoniae* was reduced by 1152 folds compared to day 0, and 65-fold more effective than in the Control group. However, due to the range of variables in measurement as well as the small size of the patient cohort represented with co-infection (*n* = 10 in Control vs. *n* = 16 in Navax groups), the observed difference did not reach statistical significance. Nevertheless, the data suggests a trend in reduction of bacterial co-infection in nasal tract when *Bacillus* spores was used. The data is also consistent with our recent data showing most of RSV patients with bacterial co-infection in Navax group achieved complete clearance of infection at day 3 of treatment while less-effective reduction in bacterial concentration was observed in the majority of patients in the Control group^26^.

Innate immunity plays an important role in influenza. T-helper responses are critical in stimulating cytotoxic T lymphocyte (CTL) proliferation, as well as the production of cytokines^31^. In infants with acute influenza infection, inflammation in the upper and lower airways is dominated by an intense neutrophilia, with overreacted release of pro-inflammatory cytokines such as IL-6, IL-8, and TNF-α produced in response to influenza^34^. According to our findings, *Bacillus* spore spraying could modulate “cytokine storm” in the nasal cavity of influenza-infected patients by regulating cell-mediated immune responses. At day 0, the levels of cytokines were at their peaks in nasopharyngeal samples, with IL-8 exhibited the highest level, followed by IL-6, and TNF-α. The data is consistent with a recent report by Garcia et al^35^. After 2 days of treatment, levels of IL-6 and IL-8 in nasopharyngeal samples of patients in the Navax group were reduced by 9.5 and 1.1 folds compared to the Control group. Meanwhile the reduction of TNF-α in both groups was equal (about fourfold reduced). Several *in-vitro* and animal studies have demonstrated that specific probiotic strains can provide protection against virus infections by regulating cytokine responses in respiratory epithelial cells^20,22^. Therefore, this study, along with the recently published study involving RSV^26^ support the notion that while cell mediated immunity does not specially prevent viral infection, it can facilitate recovery of infection related symptoms.

**Figure 6:**
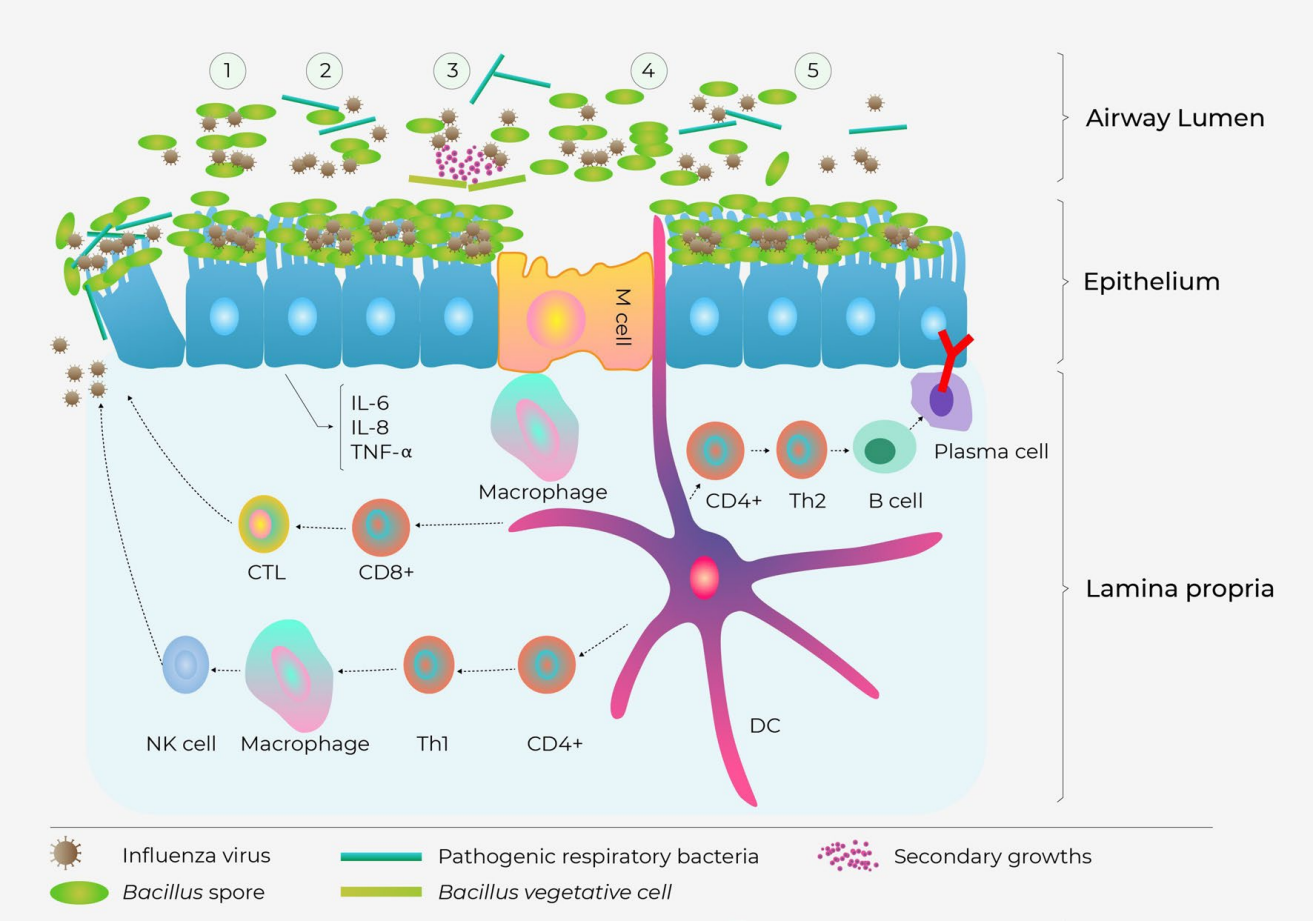
Schematic presentation of possible mechanisms of actions for nasal-spraying *Bacillus* spores against viral infection (created by Adobe Illustrator 2020, URL: https://www.adobe.com/products/illustrator.html, and adapted from Lehtoranta^20^). (1) *Bacillus* spores adsorb virions via non-specific interaction with their outer coat proteins, thereby eliminating or inactivating and the virus; (2) Abundant *Bacillus* spores adhering to the epithelial surface may block attachment of viral and co-infecting pathogens, covering receptor sites in a non-specific manner; (3) *Bacillus* spores may germinate and produce antimicrobial compounds that have direct antibacterial activity against bacterial co-infection; (4) *Bacillus* spores activate macrophages and dendritic cells (DCs): (i) CD8 + T lymphocytes differentiate into cytotoxic T lymphocytes (CTLs), (ii) Th1 differentiate from CD4 + T lymphocytes, then activate phagocytes and natural killer cells, thereby destroying virus-infected cells, (iii) Th2 differentiate from CD4 + T lymphocytes and then stimulate the proliferation of B lymphocytes that differentiate into plasma cells, which may travel back infection site to secrete IgA to neutralize the virus; (5) *Bacillus* spores compete with viruses for interaction with epithelium, thereby modulate hyper-induction of pro-inflammatory cytokines such as IL-6, IL-8, and TNF-α.

Previous studies have shown that probiotics can enhance immune response by activating IgA secretion and boosting the activity of key immune components like Peyer’s plaques, neutrophils, macrophages, natural killer cells, mesenteric lymph nodes, and intraepithelial lymphocytes. These immune-enhancing mechanisms were implied among the main effects of probiotics in defending against and managing respiratory infections^36,37^. IgA is the predominant immunoglobulin in the respiratory tract. It is known that a large repertoire of IgA produced by B lymphocytes plays an important role in protecting against viral infections, defending the epithelial barriers from pathogens, and regulating excessive immune responses in inflammatory diseases^38^. Here, we observed a significant 1.3-fold increase in IgA levels in the Navax group after 2 days of treatment, while there were also significant changes in the concentrations of influenza virus, bacterial co-infection, and cytokines. The correlative relationship suggests that the antigenic outermost proteins of *Bacillus* spores may induce secretion of mucosal IgA in the nasal tract. This, in turn, may assist in defending against influenza viruses and bacterial co-infection and regulating overactivation of the immune system. However, we noted that the observed 1.2-fold increase in IgA levels of the Navax group compared to the Control group at day 2 was not statistically significant. On the other hand, it is possible that Navax treatment may also enhance immunity against the virus and/or co-infecting bacterial pathogens. Additional sampling of patients at the later time points might provide better assessments of immune responses. However, due to ethical concerns in research involving young children, collection of nasopharyngeal samples was limited to only once for each time point day 0 and 2. Future studies to increase the sample size will help further substantiate the initial observation. It is also noted that the limited materials obtained from nasopharyngeal samples was only sufficient for measuring the viral loads, bacterial co-infection, *Bacillus* spores by real-time RT/PCR, and concentrations of the three cytokines and IgA by conventional ELISA method. Therefore, other pro-inflammatory cytokines with potential prognostic value in influenza infection relating to Th1/Th2 balance were not included in the evaluation. The procedure thus limited the evaluation of other immuno-stimulatory parameters that regulate immune cell proliferation and differentiation following influenza infection for better understanding mechanisms of action for nasal-spraying *Bacillus* spores against influenza infection. It also restricted the assessment of changes in viral and bacterial loads, as well as immune indices, over time. As a result, the constraints hinder our interpretability of the supporting data for subclinical parameters influenced by the nasal-spraying of *Bacillus* spores. Taken all together, we propose that sprayed *Bacillus* spores alleviate influenza virus infection in nasal tracts via multiple pathways (the proposed model presented in Fig. 6).

Because the mechanism of interaction between *Bacillus* spores with nasal mucosal immune system, virus, and bacteria is non-specific, our findings in both this study and the published RSV study^26^ provide strong evidence that nasal-spraying *Bacillus* spores could be useful for supportive treatment of rapidly emerging RTIs viruses, including influenza virus, RSV, and adenovirus, rhinovirus, and coronavirus, among others. Nasal-spraying *Bacillus* spores can provide an additional barrier of protection which increases the overall effectiveness of vaccines and drugs in preventing and treating viral infection as well as reduces the cost and treatment time of antibiotics for bacterial co-infection. The probiotic treatment is easy to administer, low cost with the use of bioreactors, and effective against respiratory viral infection. These factors make it an ideal solution for developing countries that have limited medical resources and face challenges with hospital overload. In a near future, a clinical trial should be conducted to evaluate the preventive effects of nasal-spraying *Bacillus* spores on respiratory tract infections in children during the transitional season, especially when there is a high prevalence of viral and bacterial pathogens.

## Conclusion

This is the first clinical-trial to demonstrate the safety and effects of nasal-spraying *Bacillus* liquid-form probiotics in the symptomatic treatment of acute ARTIs in children infected with influenza virus. This novel supportive approach has shortened the treatment period by 2 days and improved symptomatic treatment efficacy by 58%. The sprayed *Bacillus* spores significantly inhibited the multiplications of influenza virus (417 folds), reduced bacterial co-infection concentrations (1152 folds), decreased the overreacted immune response of epithelium cells to lower IL-6 (53.9-fold), IL-8 (1.1-fold), and TNF-α (3.7-fold) pro-inflammatory cytokine levels, and increased IgA (by 1.3-fold) in nasal tract right at day 2 of treatment period. This was 26-fold (for viral loads), 65-fold (for bacterial concentrations), 1.1–9.5-fold (for cytokine levels), and 1.3-fold (for IgA levels) more effective than the control standard-of-care group.

## Materials and methods

### Materials

Nasal-spraying probiotics LiveSpo Navax (LiveSpo Pharma, Hanoi, Vietnam) was formulated as a 0.9% NaCl physiological saline suspension containing *Bacillus subtilis* ANA4 (accession no. MT123906.1 in NCBI) and *B. clausii* ANA39 (accession no. MT275656.1 in NCBI) spores at ≥ 5 × 10^9^ CFU/ 5 mL^26^. The product was manufactured as a Class-A medical device product (No: 210001337/PCBA-HN) under ISO 13,485:2016 standard. Taste and smell were indistinguishable between LiveSpo Navax and 0.9% NaCl physiological saline (B.Braun, Germany). Because of the opaque plastic container, the color and turbidity of LiveSpo Navax suspension is unrecognizable.

### Ethical issues, study design, and patient collection

This study received ethics approval by the Ethics Committee in Medical Research of the Vietnam National Children’s Hospital under Decision No. 441/BVNTW-VNCSKTE, and was conducted with the ethical principles in accordance with the Helsinki statement and the ICH GCP guidelines, the Health Department's current ethical regulations and standards of research using subject’s human. All parents of pediatric patients who volunteered to participate in the study were given information about the study and signed an informed consent form. The study was registered with ClinicalTrials.gov of US. National Library of Medicine with Identifier No: NCT05378022 on 17/05/2022.

This was a double-blind, randomized, controlled intervention pilot study. Patients were randomly assigned to either the control group (named “Control” group) received 0.9% NaCl or the experimental group (named “Navax” group) received the probiotics LiveSpo Navax. The study was implemented at the International Center, Vietnam National Children’s Hospital from December 2020 to April 2022. The size of the patient cohorts (*n* = 43 per group) was calculated based on a hypothesis that LiveSpo Navax treatment effectively alleviates influenza-infection symptoms by 25% more than standard of care with α = 0.05 and the power level of 0.8^26^. A total of 218 participants were screened for eligibility and 100 eligible participants (*n* = 50 per group) were randomly assigned by lottery to Control and Navax group to reduce the risk of about 20% patient’s drop out during follow-up treatment. After parents of children signed the informed consent form, the chief nurse randomly selected paper sheets with the coding numbers 1 or 2 from a carton box and immediately assigned the coding number to each participant. The Control and Navax groups were assigned the numbers 1 and 2, respectively, and this information was also confidential to parents of children, nurses, and investigators, with the exception of the principal investigator and the data analyst^26^. The flowchart of the study design is shown in Fig. 1.

The inclusion criteria for this study included children of both genders, aged between 4 months and 7 years, who were admitted to the hospital due to upper respiratory infections and tested positive for both flu A and flu B through rapid testing and had consents from parents. Exclusion criteria included newborn babies, having a history of drug allergies, the need for oxygen therapy, being discharged before day 2, being lost to follow-up, being withdrawn from the trial, continuing in the trial but missing data, receiving flu vaccination within one year, and meeting the criteria for psychiatric disorders other than depression and/or anxiety.

### Questionnaires, treatment procedures, and clinical observation

The patient's parents were required to provide information of their children. Nurses were given coded sprayers in the form of blind samples and were educated to use the sprayers with dosages of about 50 µl 0.9% NaCl physiological saline (with/without 2.5 × 10^8^ *Bacillus* spores) per each nasal cavity/ time × 3 times/ day directly into the nasal cavity continuously for maximal follow-up 5 days of treatment. As shown in Table 2, the nasal spray products were applied in parallel with routine treatment drugs at hospital. Antibiotics were assigned for all patients who tested positive for co-infection pathogens. For primary outcomes, patients were monitored daily during treatment for typical clinical symptoms of influenza infections, including runny nose, dry rales, moist rales, body temperature (^o^C), pulse oxymetry (SpO_2_) (%), pulse (beats/min), and breath (beats/min) until discharged.

### Routine diagnostics at hospital

Screening of influenza-infected cases from nasopharyngeal samples at day 0 was firstly conducted by using “BD Veritor System for Detection of Flu A + B” kit (Bection Dickison, NJ, US). Serum C-reactive protein (CRP) concentrations and white blood cell counts were measured to access the level of infection. Cardiopulmonary X-ray was appointed for visualization of lung hyperinflation, osler’s nodes, atelectasis… Due to ethical concerns, all these tests were conducted only at day 0 (Table 2).

### Real-time PCR for detection of microorganism in nasopharyngeal samples

DNA/RNA from 200 µl nasopharyngeal specimens (repeated twice) was extracted by MagNA Pure LC Total Nucleic Acid Isolation Kit (Roche Diagnostics, IA, US), and 100 µl of the purified DNA/RNA was aliquoted into three PCR tubes (approximately 30 µl/tube) for storage at – 80 °C.

As secondary outcomes, semi-quantitative assays for measuring changes in viral load of influenza virus (types A and B) and bacterial co-infection concentrations (*Streptococcus pneumoniae* and *Haemophilus influenzae*) in nasal tract between days 0 and 2 was conducted by the real-time RT-PCR/PCR, following a similar methodology as described previously^26,39–41^,. The primers and probes used for specific amplification of influenza virus types A, B, and Ribonuclease P as internal control^39–41^ by real-time RT-PCR TaqMan probe was presented in Table S1. Reactions were set up to include an initial reverse transcription at 50 °C for 30 min, 95 °C for 2 min, followed by 45 amplification and detection cycles at 95 °C for 15 s, 55 °C for 30 s. To simultaneously detect multiple bacterial co-infections, the Allplex Respiratory Panel 4 kit (Seegene, Seoul, Korea) was utilized. This commercial kit allows for the detection of seven pathogenic bacterial species, including *Bordetella parapertussis* (BPP), *Bordetella pertussis* (BP), *Chlamydophila pneumoniae* (CP), *Haemophilus influenzae* (HI), *Legionella pneumophila* (LP), *Mycoplasma pneumoniae* (MP), *Streptococcus pneumoniae* (SP). Reactions following company’s protocol used an initial denaturation at 95 °C for 15 min, followed by 45 amplification and detection cycles at 95 °C for 10 s, 60 °C for 1 min, 72 °C for 10 s. The read-out standardization for the analysis of influenza virus and bacterial co-infection needed to be adjusted to a C_t_ of < 40 to confirm whether they are a true positive or not. The protocols for influenza virus and the bacterial co-infection detection had been standardized according to ISO 15,189:2012 criteria and routinely employed for testing of clinical samples in Department of Molecular Biology for Infectious Diseases, Vietnam National Children’s Hospital.

Detection of *B. subtilis* ANA4 and *B. clausii* ANA39 in nasopharyngeal samples was also conducted at day 0 and day 2 by real-time PCR SYBR Green using primers specific for detection of *B. subtilis*^42^ and *B. clausii*^43^ (Table S1) at the following condition: 95 °C for 10 min, amplification for 40 cycles at 95 °C for 15 s, 60 °C for 20 s, 72 °C for 30 s. The read-out standardization for *B. subtilis* and *B. clausii* analysis was set at C_t_ < 35 to confirm true positive. The protocol for *B. subtilis* and *B. clausii* detection had been developed following the ISO 17,025:2017 guideline and applied for research purpose only at Department of Molecular Biology for Infectious Diseases, Vietnam National Children’s Hospital.

### ELISA assays for cytokine and IgA levels

Other secondary outcomes including (i) pro-inflammatory cytokine levels (pg/mL) of interleukin (IL-6, IL-8) and TNF-α, and (ii) IgA levels (mg/mL) in nasopharyngeal samples at days 0 and 2 were quantified using an enzyme-linked immunosorbent assay kit (ELISA). IL-6 and TNF-α were quantified from 100 µL samples by the Human IL-6 DuoSet ELISA and the Human TNF-α ELISA kit, respectively (R&D Systems, MN, US). IL-8 was quantified by 50 µL samples by IL-8 Human ELISA kit (Invitrogen/Thermo Fisher Scientific, MA, US). IgA was quantified by 10 µL samples by Human IgA ELISA kit (Invitrogen/Thermo Fisher Scientific, MA, US).

### Data collection and statistical analysis

Individual medical records were collected, and the patient's information was then gathered in a data set. The reduction levels (2^△Ct^) of influenza viral load and bacterial co-infection concentrations. △C_t_ for target genes was calculated as C_t_ (threshold cycle) at day 2–C_t_ at day 0 while C_t_ of internal control was adjusted to be equal among all samples. Tabular analysis was conducted on dichotomous variables using either χ^2^ test, Fisher’s exact test. The Wilcoxon test or Mann–Whitney test was utilized to compare the medians of quantitative variables within the same group or between two groups. GraphPad Prism v8.4.3 software was used for statistical and graphical analyses (GraphPad Software, CA, USA). All analyses were conducted with a significance level of *p* < 0.05.

### Supplementary Information

Supplementary Figure S1.

Supplementary Information.

## Data Availability

The datasets generated during this current study is available at following URLs: 1. https://anabio.com.vn/documents/; 2. https://orcid.org/0000-0001-6714-068X.
