# Supplementary material for: Efficient symptomatic treatment and viral load reduction for children with influenza virus infection by nasal-spraying Bacillus spore probiotics
Source: Sci Rep. 2023 Sep 8;13:14789. doi: 10.1038/s41598-023-41763-5 (PMC10491672; doi:10.1038/s41598-023-41763-5)
Supplement: Supplementary file 1 — Supplementary Figure S1. [file 41598_2023_41763_MOESM1_ESM.pdf]

Supplemental Fig. S1

No	Patient code	(A) FLU	(B) *B. subtilis & B. clausii*
			Red — Positive Control (PC) of *B. subtilis*<br>Blue — PC of *B. clausii*<br>Black — Negative control (NC) of *B. subtilis*, NC of *B. clausii*<br>Sample code_S: curves specific for *B. subtilis*; Sample code_C: curves specific for *B. clausii*
1	C01_D0	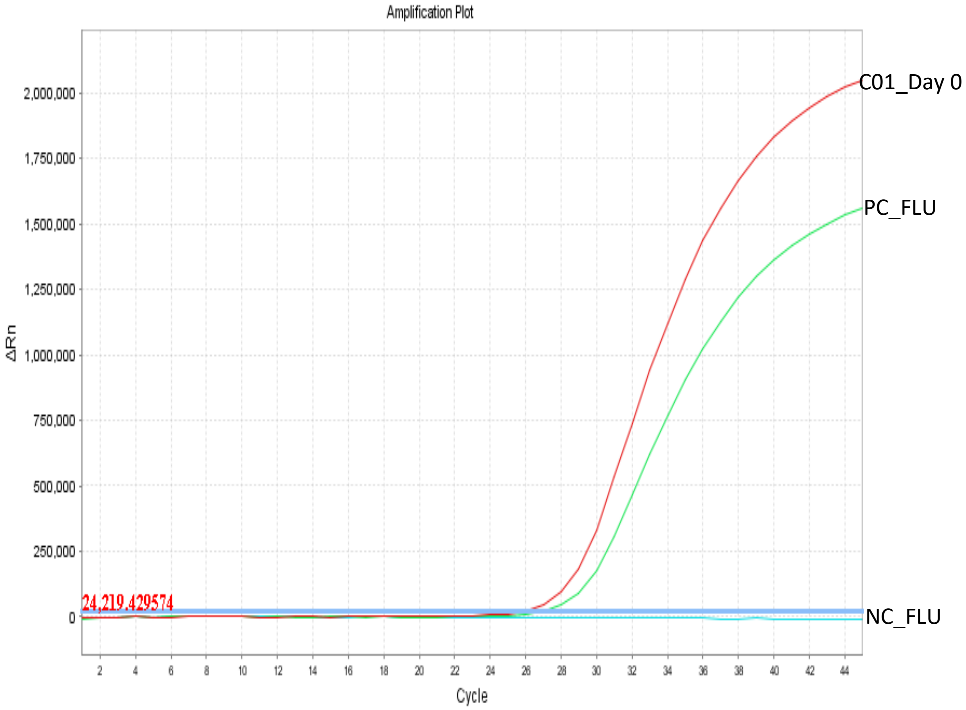	

<table>
<tr>
<td></td>
<td>C01_D2</td>
<td>
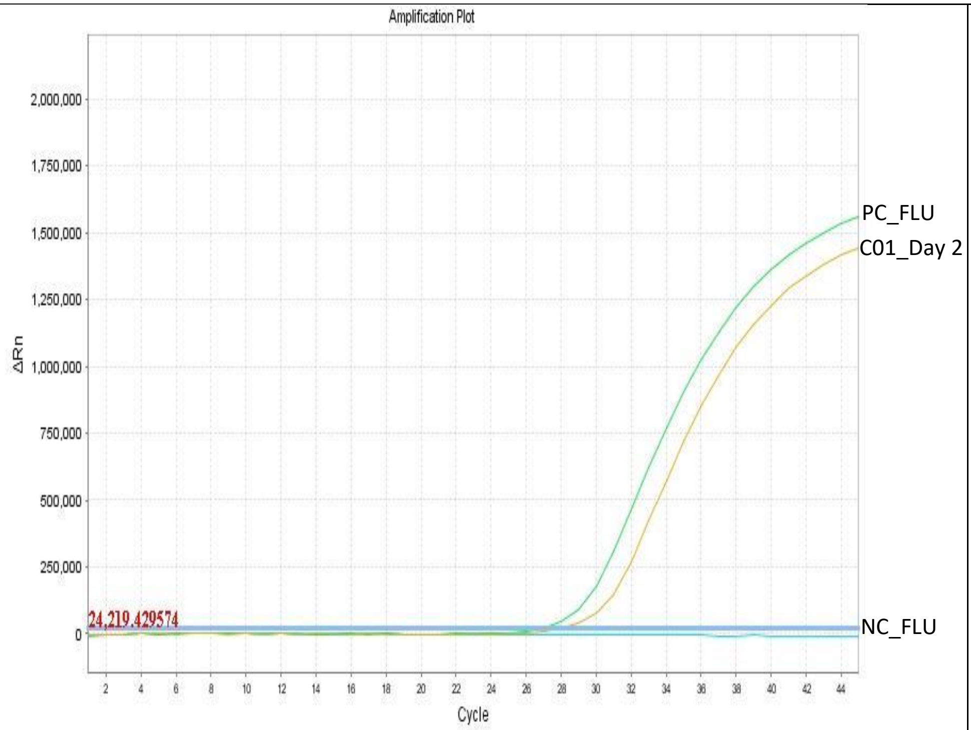

</td>
<td>
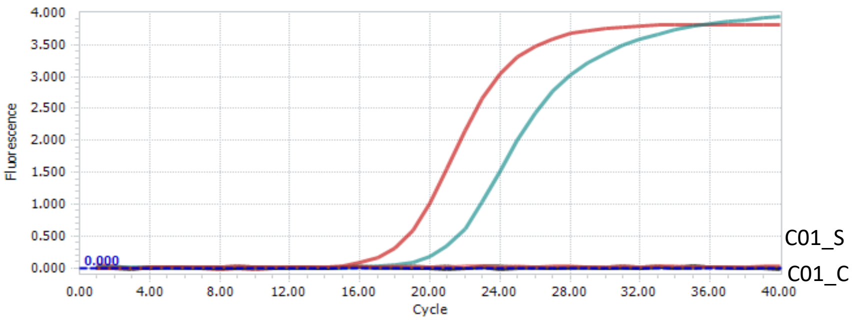

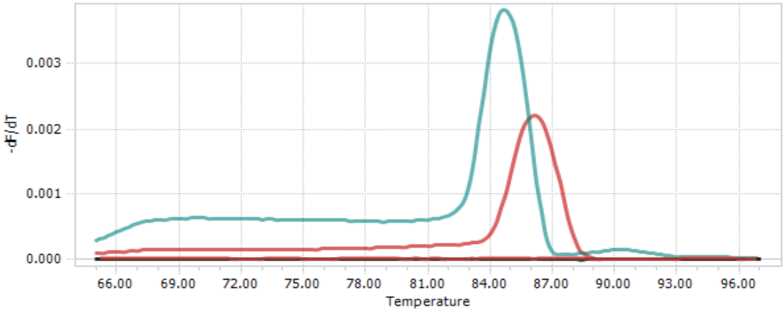

</td>
</tr>
</table>

2	C02_D0	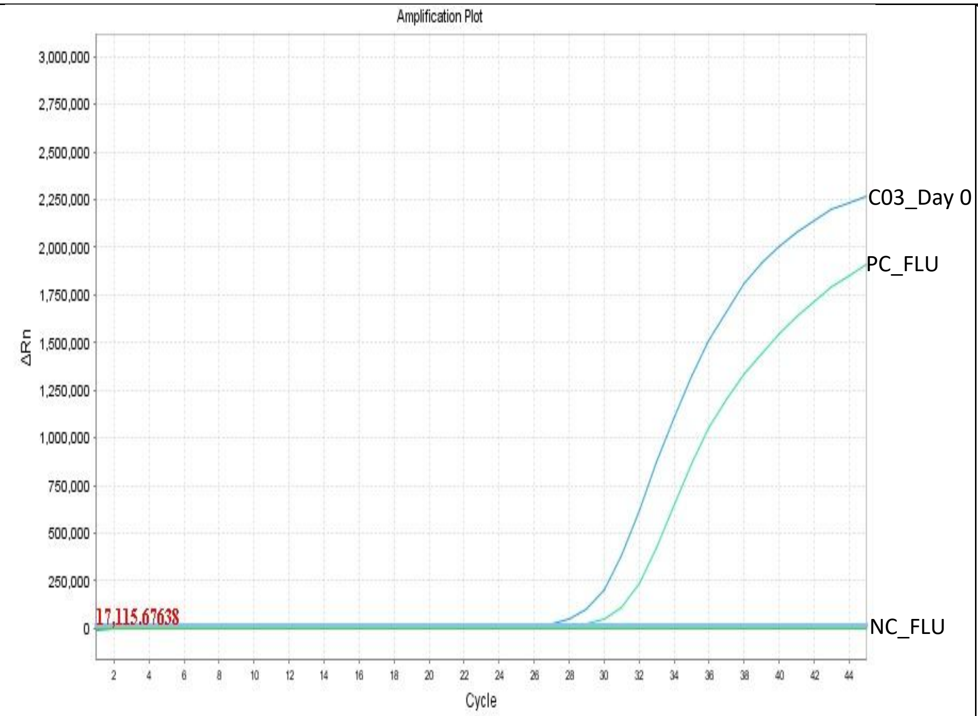Amplification Plot
3,000,000
2,750,000
2,500,000
2,250,000
2,000,000
1,750,000
1,500,000
1,250,000
1,000,000
750,000
500,000
250,000
0
ΔRn
17,115.67638
2 4 6 8 10 12 14 16 18 20 22 24 26 28 30 32 34 36 38 40 42 44
Cycle
C03_Day 0
PC_FLU
NC_FLU	

<table>
<tr>
<td></td>
<td>C02_D2</td>
<td>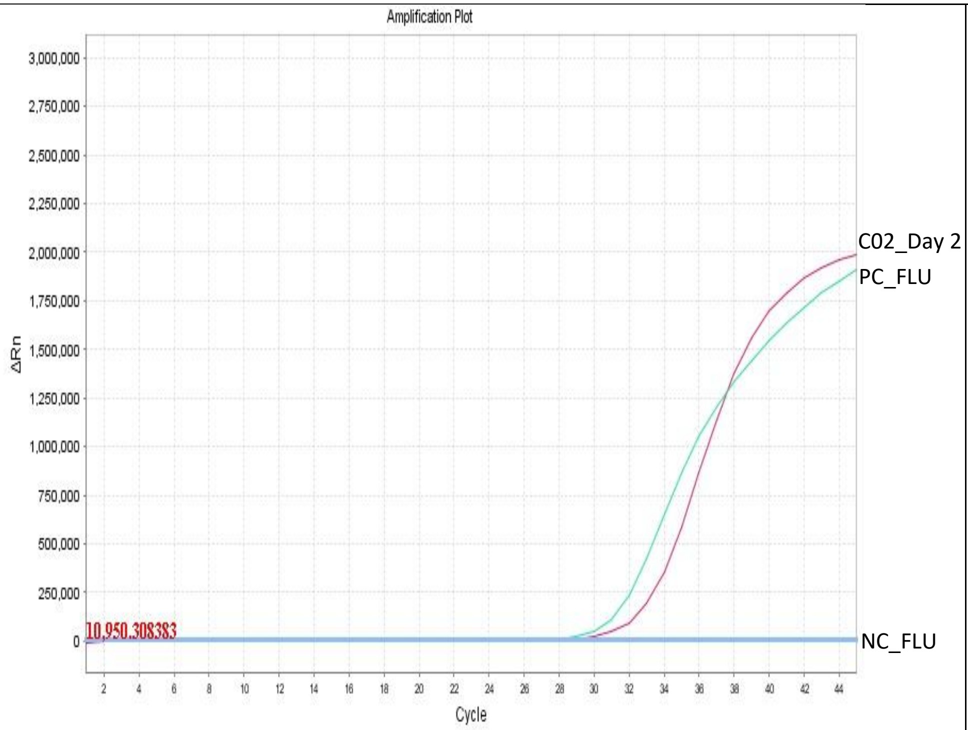
</td>
<td>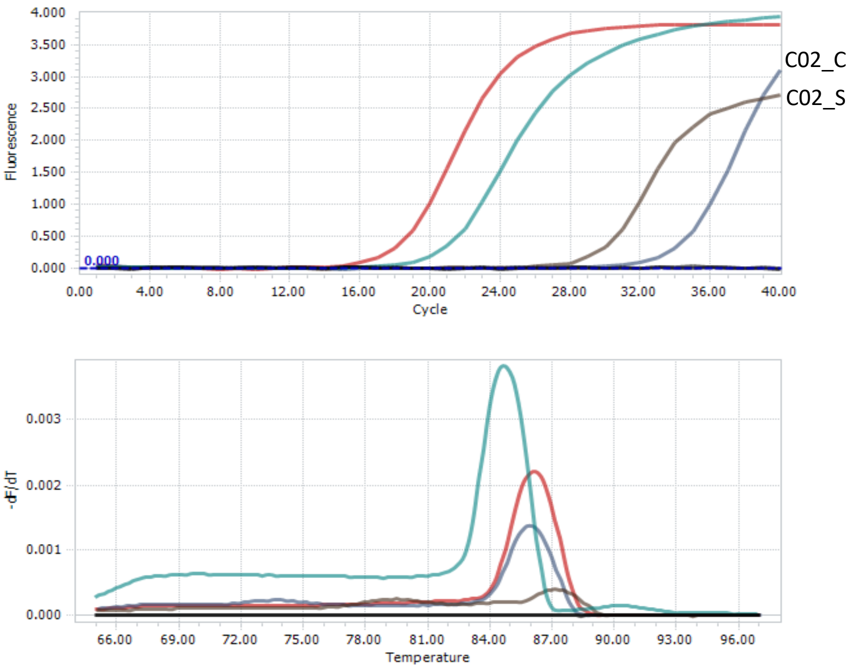
</td>
</tr>
</table>

3	C04_D0	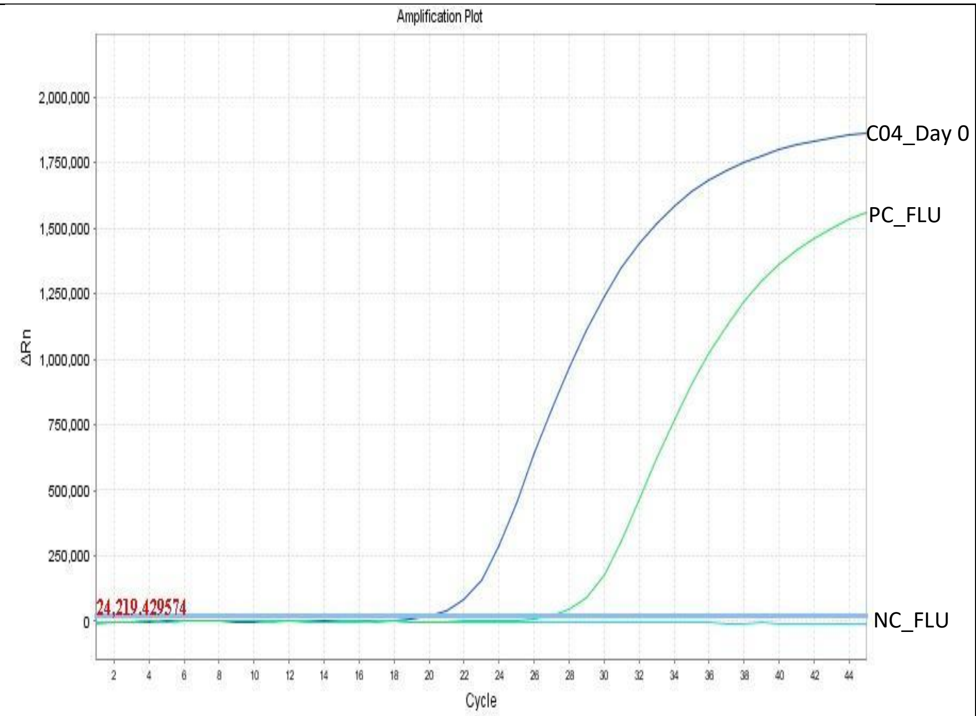Amplification Plot<br>ΔRn<br>Cycle<br>2,000,000<br>1,750,000<br>1,500,000<br>1,250,000<br>1,000,000<br>750,000<br>500,000<br>250,000<br>0<br>24,219.429574<br>C04_Day 0<br>PC_FLU<br>NC_FLU	

	C04_D2	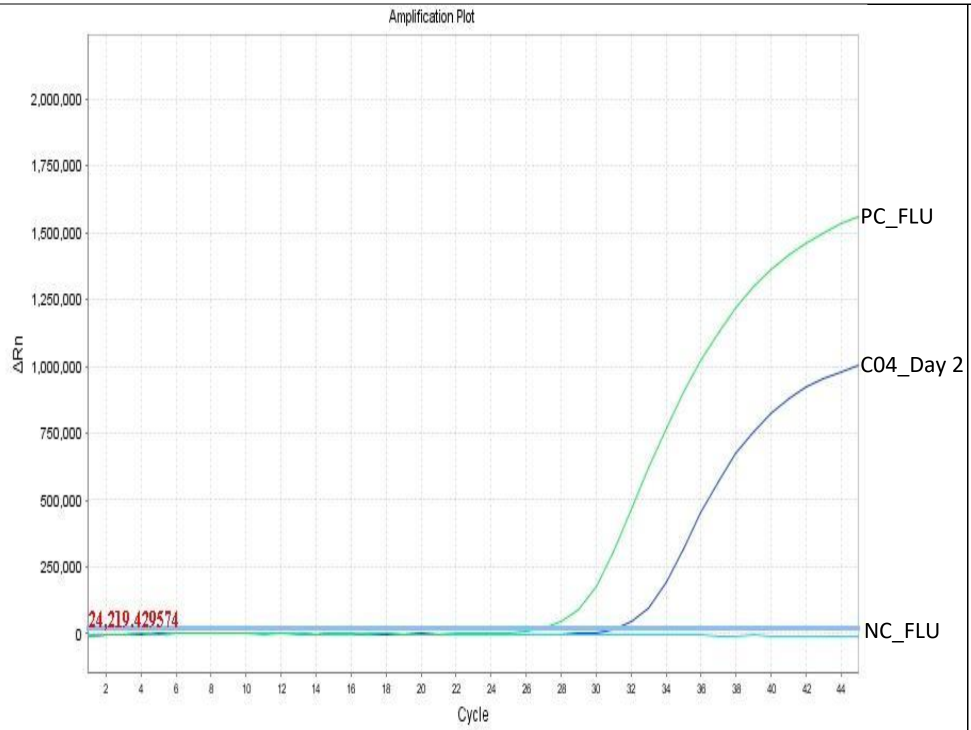	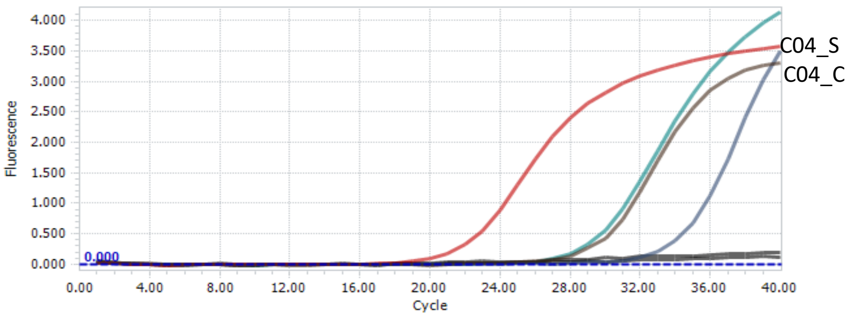 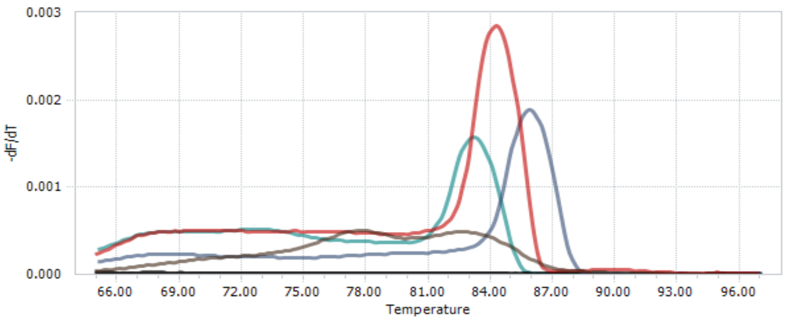

Amplification Plot
ΔRn
2,000,000
1,750,000
1,500,000
1,250,000
1,000,000
750,000
500,000
250,000
0
24,219.429574
2 4 6 8 10 12 14 16 18 20 22 24 26 28 30 32 34 36 38 40 42 44
Cycle
PC_FLU
C04_Day 2
NC_FLU

Fluorescence
4.000
3.500
3.000
2.500
2.000
1.500
1.000
0.500
0.000
0.000
0.00 4.00 8.00 12.00 16.00 20.00 24.00 28.00 32.00 36.00 40.00
Cycle
C04_S
C04_C

-dF/dT
0.003
0.002
0.001
0.000
66.00 69.00 72.00 75.00 78.00 81.00 84.00 87.00 90.00 93.00 96.00
Temperature

4	C05_D0	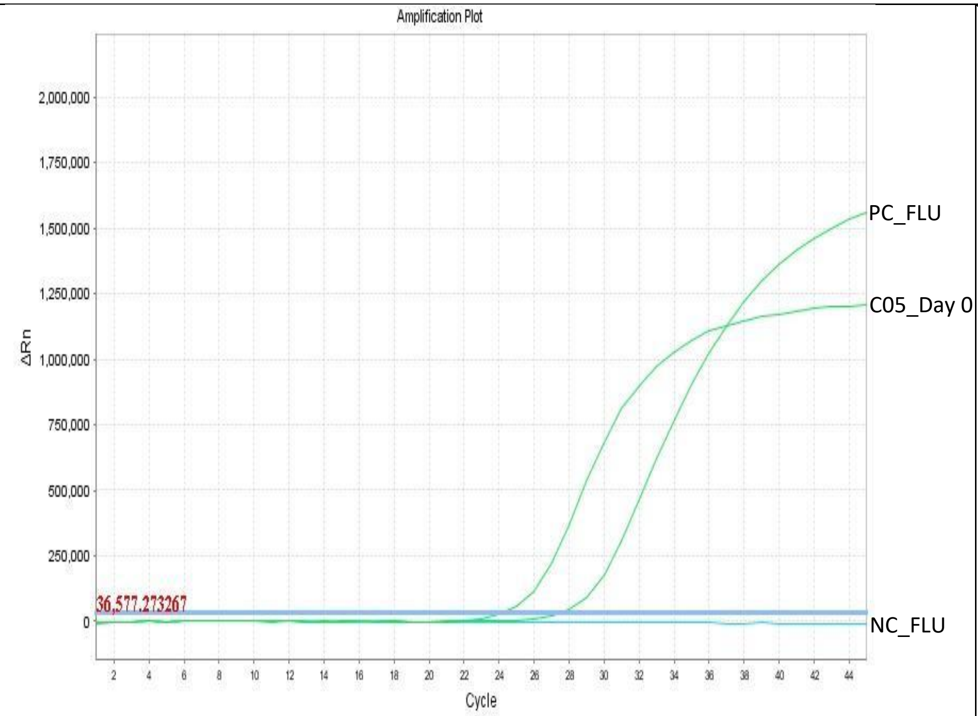Amplification Plot
2,000,000
1,750,000
1,500,000
1,250,000
1,000,000
750,000
500,000
250,000
0
ΔRn
36,577.273267
2
4
6
8
10
12
14
16
18
20
22
24
26
28
30
32
34
36
38
40
42
44
Cycle
PC_FLU
C05_Day 0
NC_FLU	

C05_D2

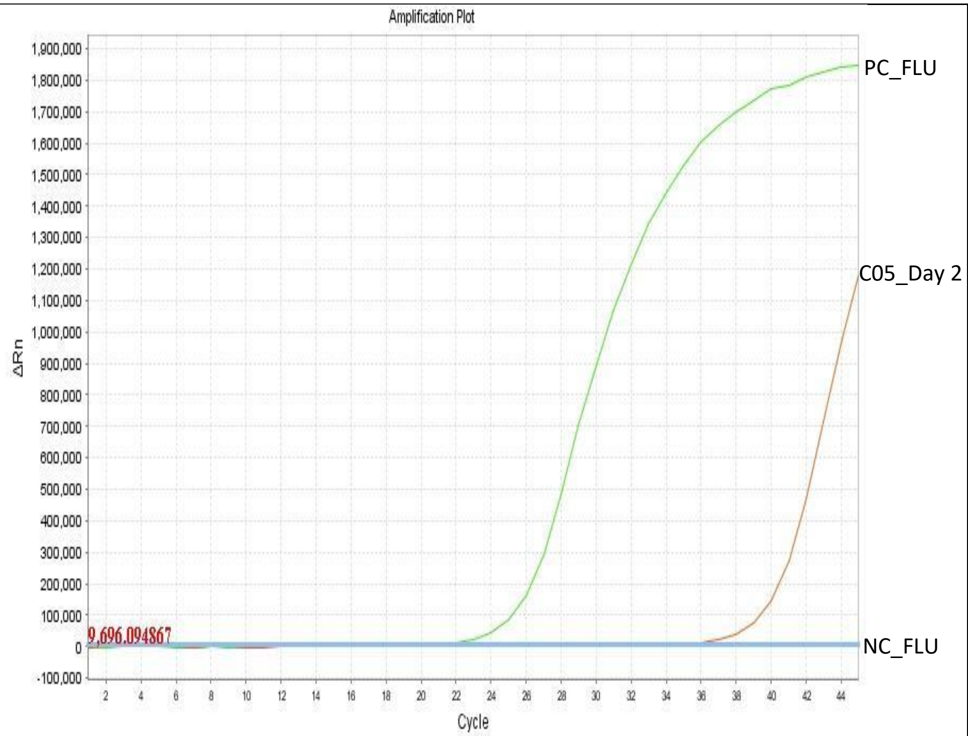
Amplification Plot
ΔRn
Cycle
9,696.094867
PC_FLU
C05_Day 2
NC_FLU

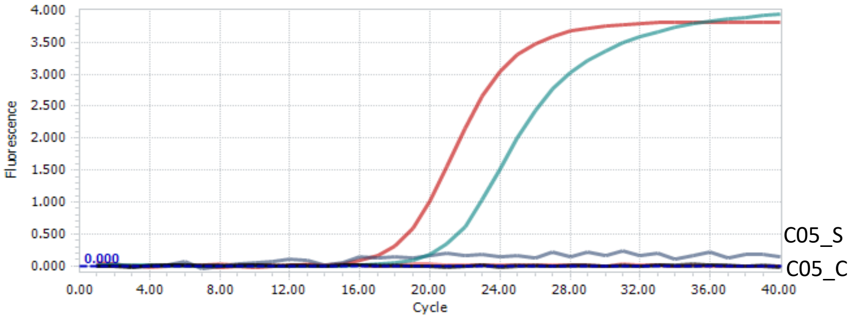
Fluorescence
Cycle
0.000
C05_S
C05_C

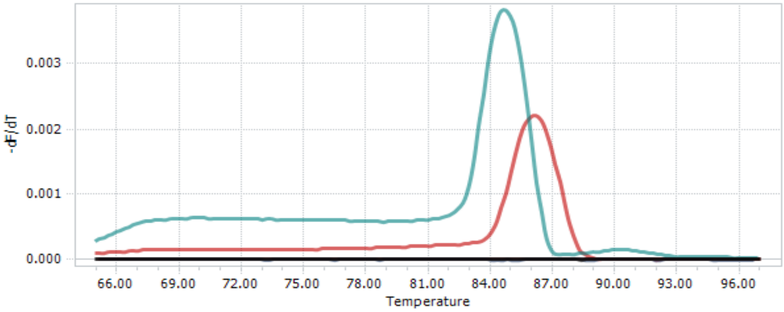
-dF/dT
Temperature

5	C06_D0	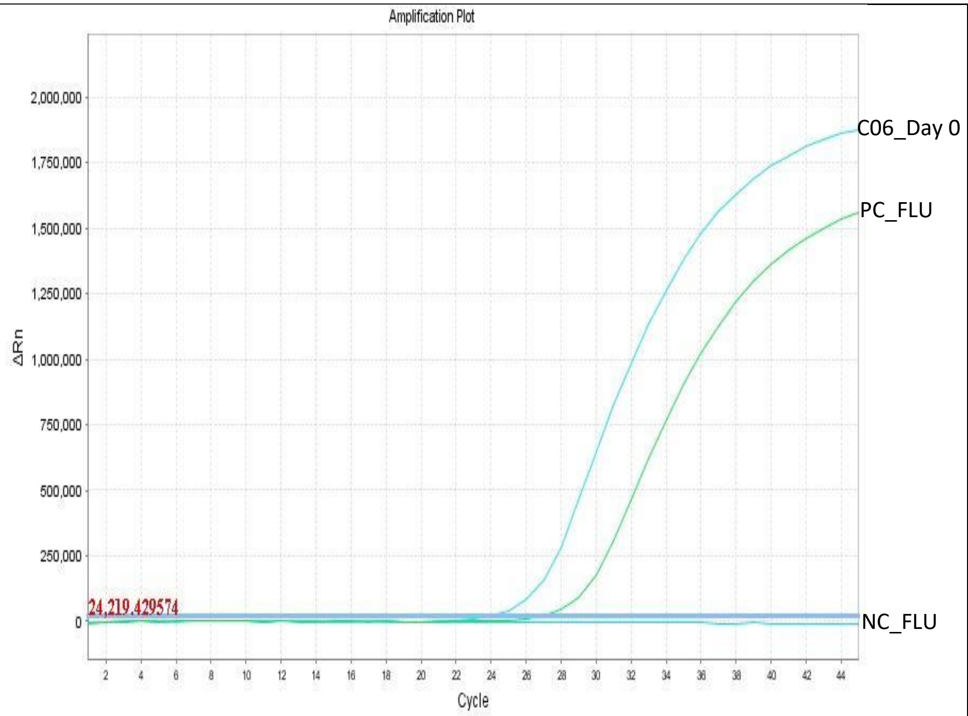Amplification Plot<br>ΔRn<br>Cycle<br>24,219.429574<br>C06_Day 0<br>PC_FLU<br>NC_FLU	

	C06 _D2	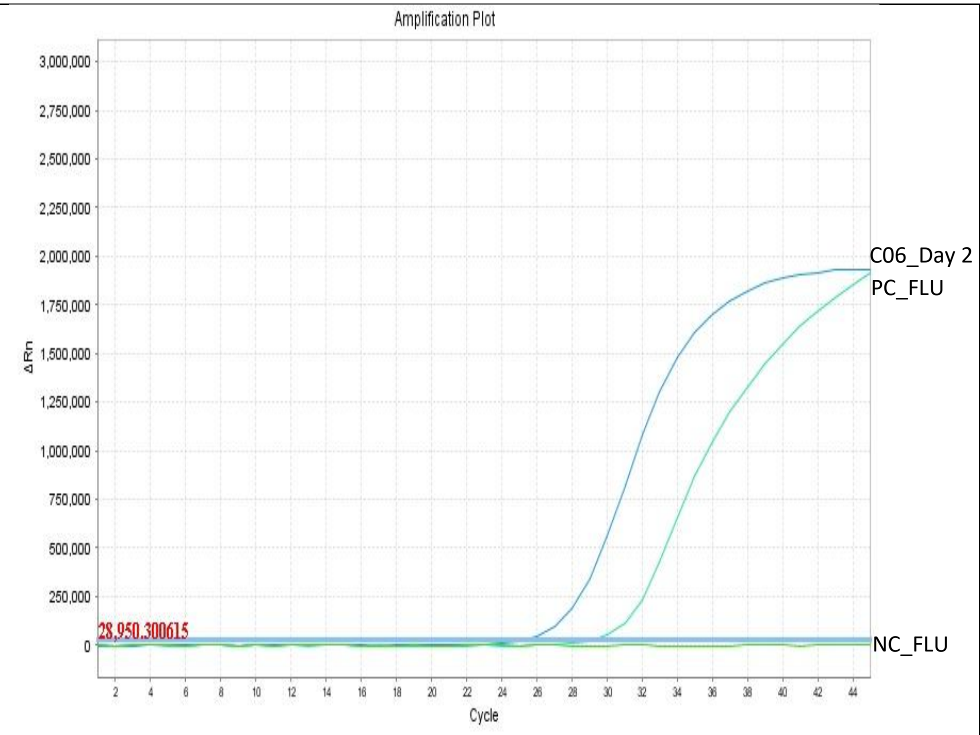 Amplification Plot<br>ΔRn<br>Cycle<br>28,950.300615<br>C06_Day 2<br>PC_FLU<br>NC_FLU	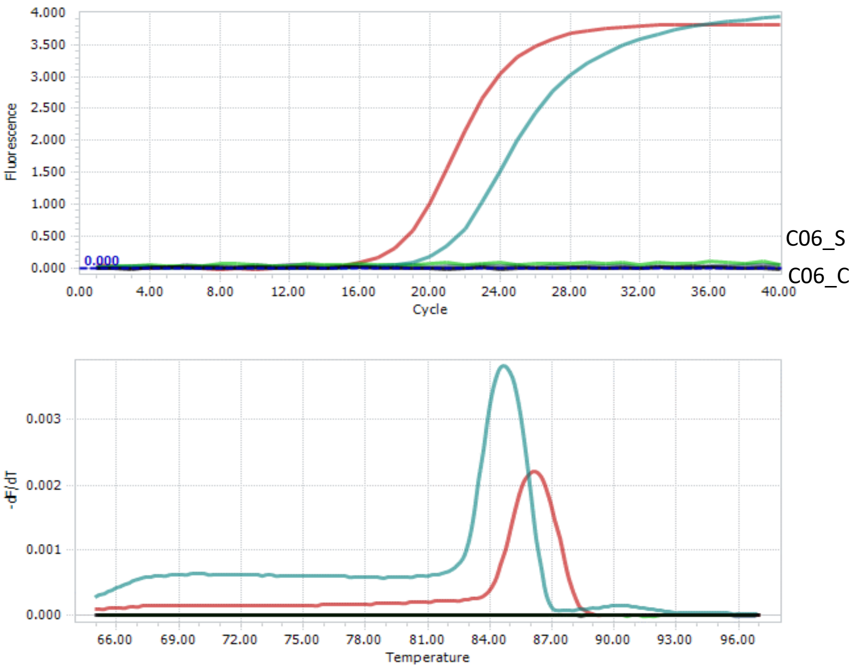 Fluorescence<br>Cycle<br>0.000<br>C06_S<br>C06_C<br>-dF/dT<br>Temperature

6	C07_D0	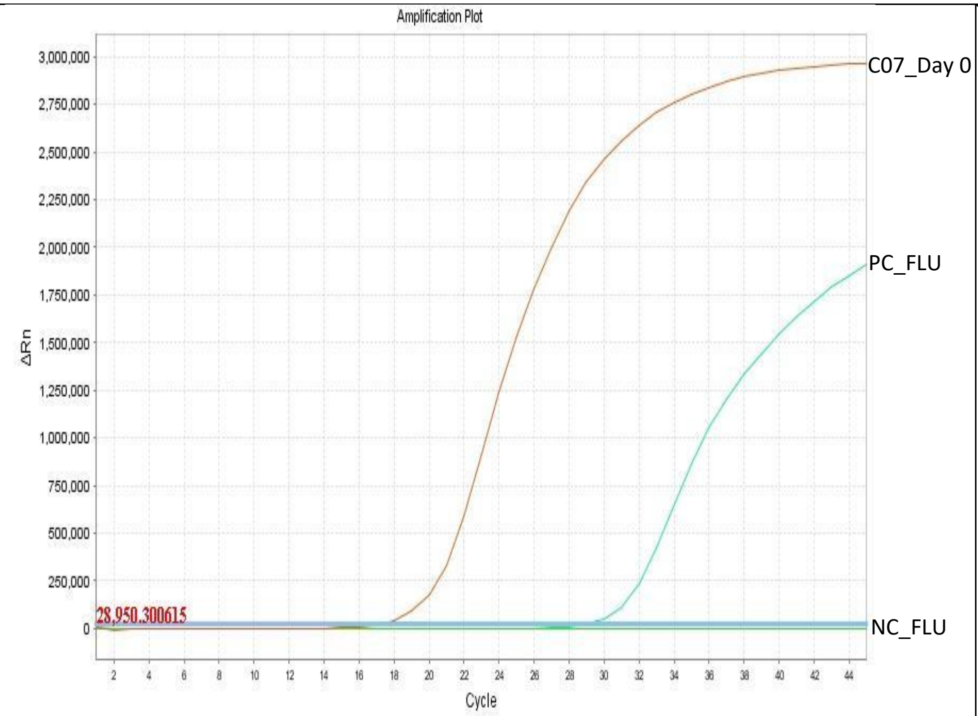Amplification Plot<br>3,000,000<br>2,750,000<br>2,500,000<br>2,250,000<br>2,000,000<br>1,750,000<br>1,500,000<br>1,250,000<br>1,000,000<br>750,000<br>500,000<br>250,000<br>0<br>ΔRn<br>28,950.300615<br>2 4 6 8 10 12 14 16 18 20 22 24 26 28 30 32 34 36 38 40 42 44<br>Cycle<br>C07_Day 0<br>PC_FLU<br>NC_FLU	

<table>
<tr>
<td></td>
<td>C07_D2</td>
<td>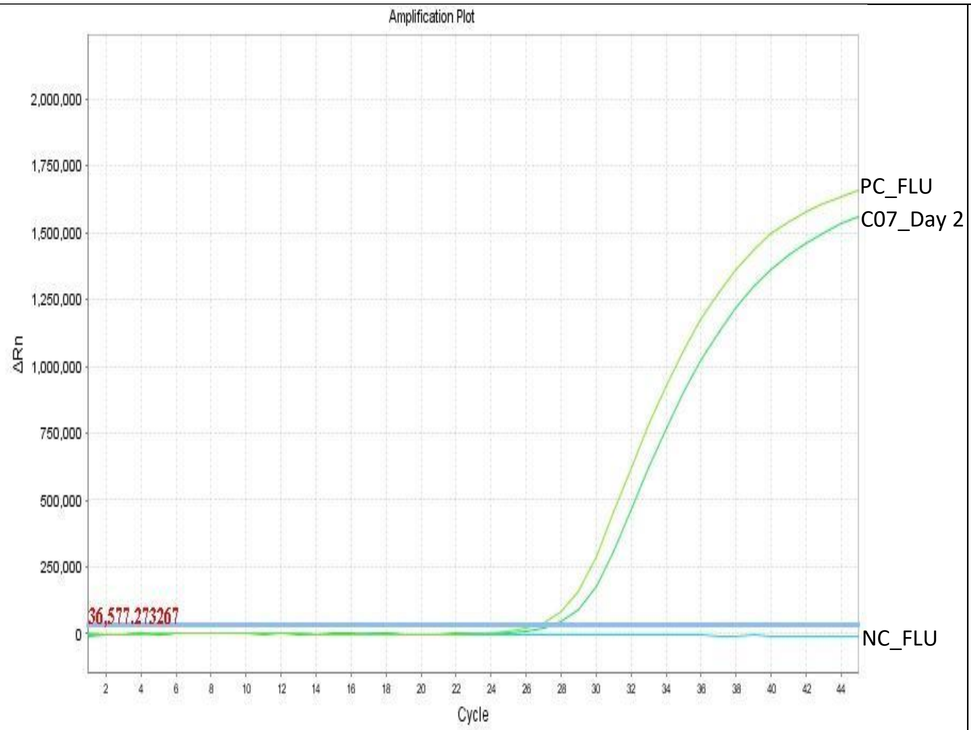
</td>
<td>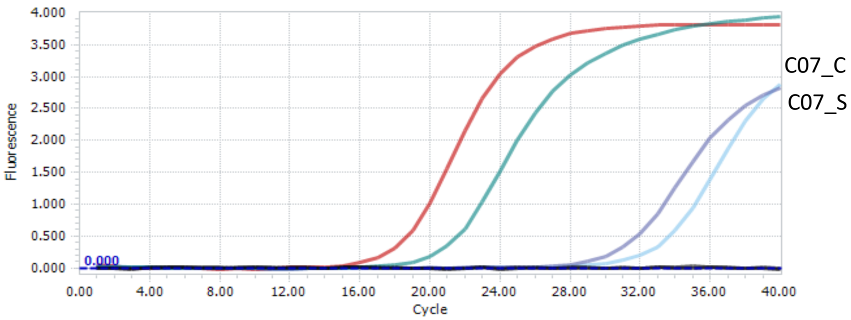

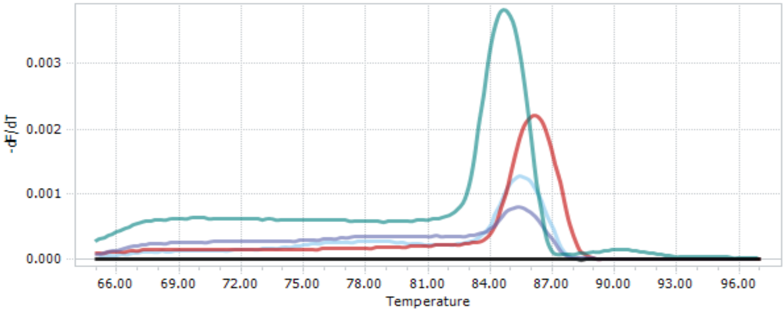
</td>
</tr>
</table>

7	C08_D0	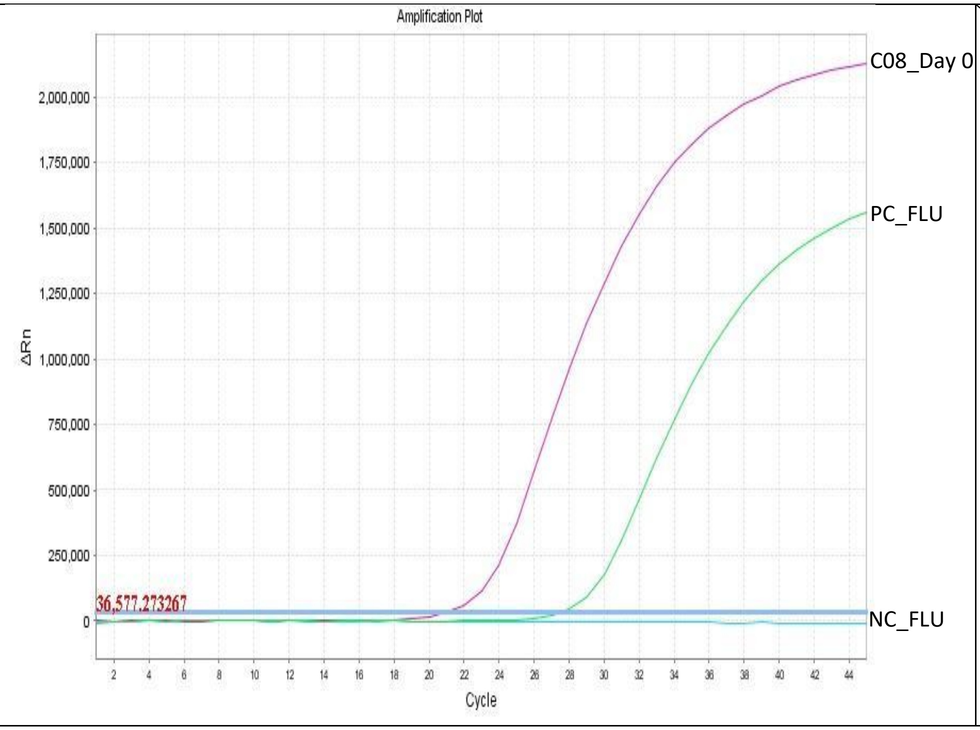Amplification Plot
C08_Day 0
PC_FLU
NC_FLU
36,577.273267
ΔRn
Cycle
2,000,000
1,750,000
1,500,000
1,250,000
1,000,000
750,000
500,000
250,000
0
2
4
6
8
10
12
14
16
18
20
22
24
26
28
30
32
34
36
38
40
42
44	

C08_D2

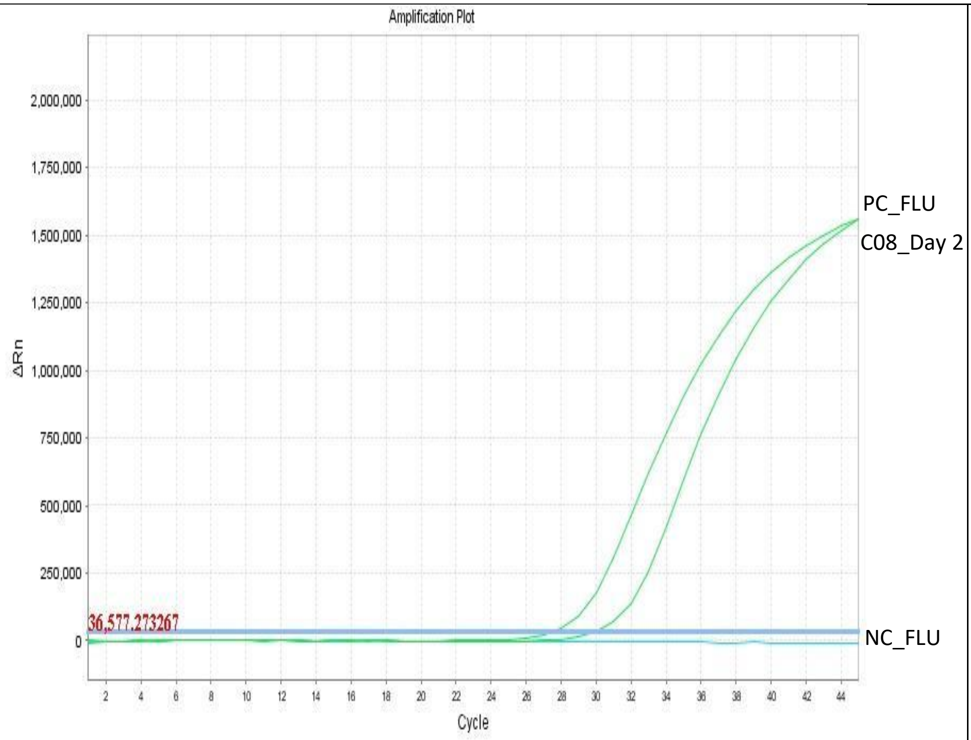
Amplification Plot
ΔRn
Cycle
36,577.273267
PC_FLU
C08_Day 2
NC_FLU

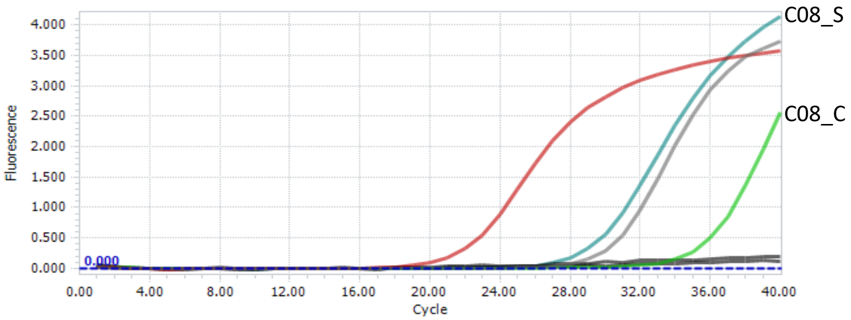
Fluorescence
Cycle
C08_S
C08_C

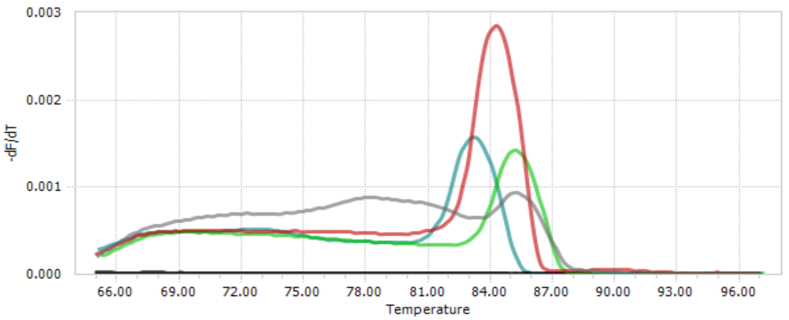
-dF/dT
Temperature

8	C09_D0	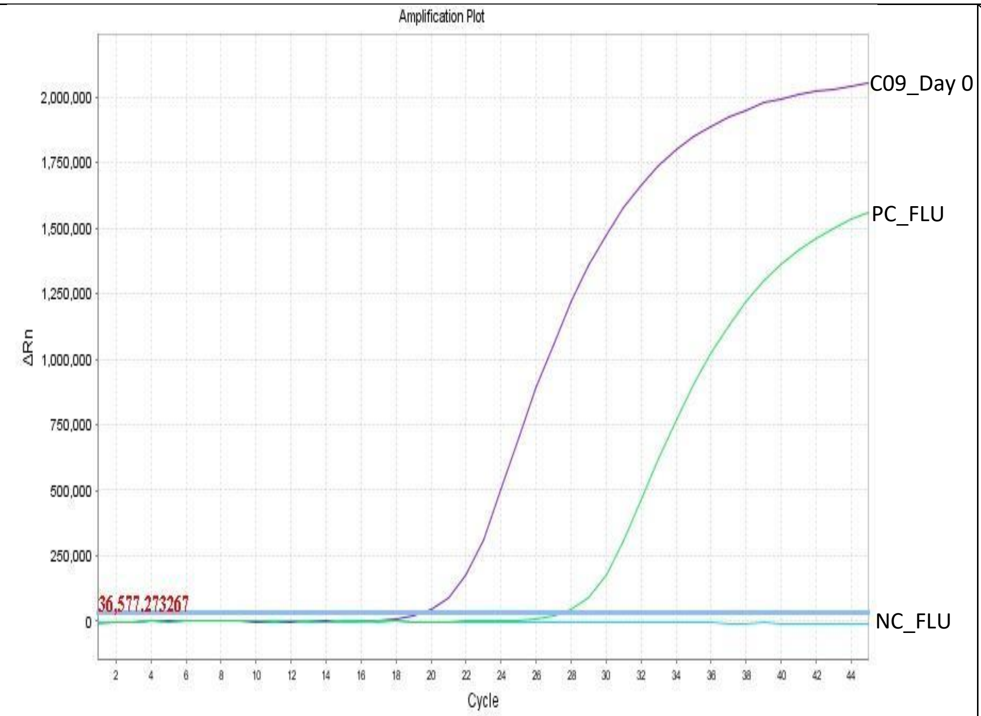Amplification Plot
2,000,000
1,750,000
1,500,000
1,250,000
1,000,000
750,000
500,000
250,000
0
ΔRn
36,577.273267
2 4 6 8 10 12 14 16 18 20 22 24 26 28 30 32 34 36 38 40 42 44
Cycle
C09_Day 0
PC_FLU
NC_FLU	

<table>
<tr>
<td></td>
<td>C09
_D2</td>
<td>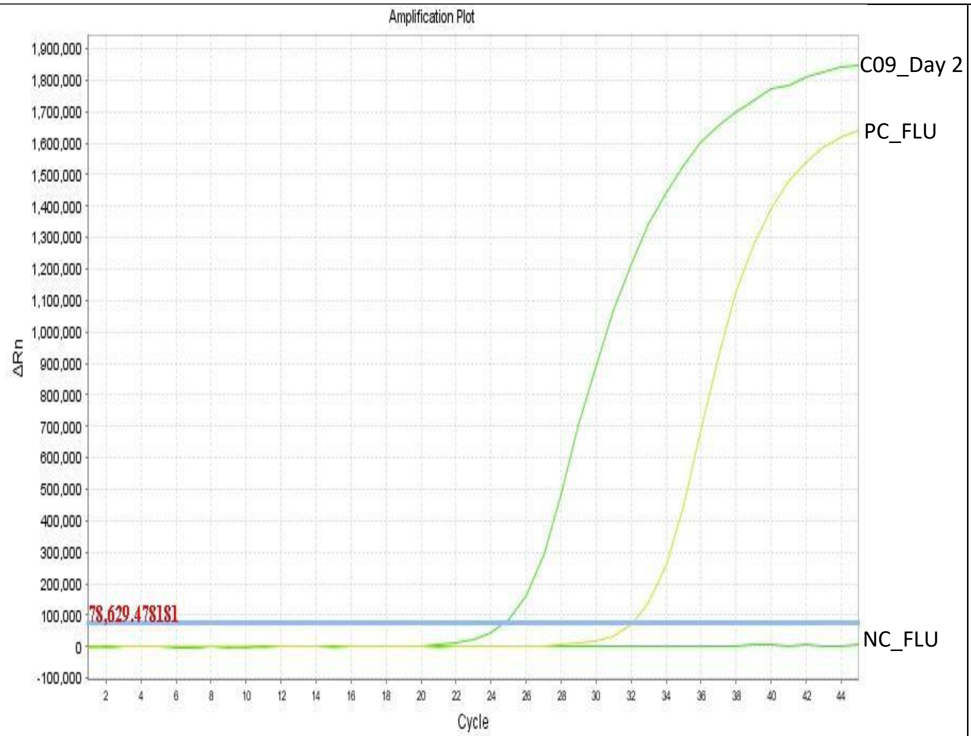
</td>
<td>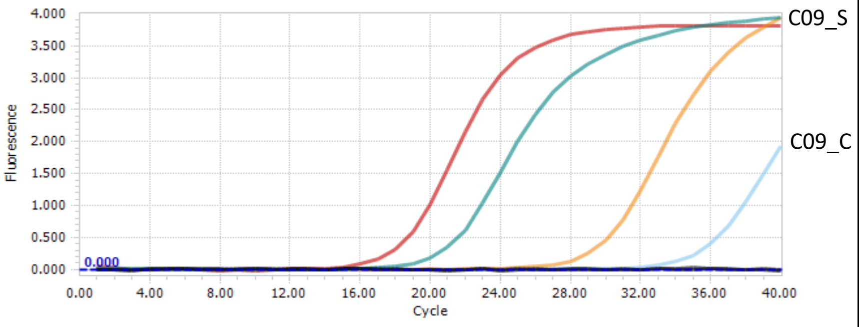

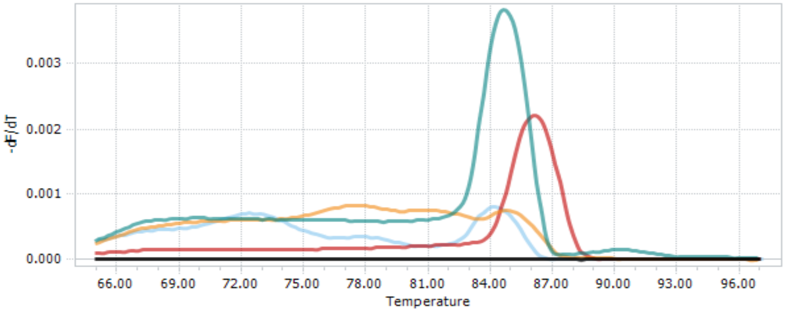
</td>
</tr>
</table>

9	C10_D0	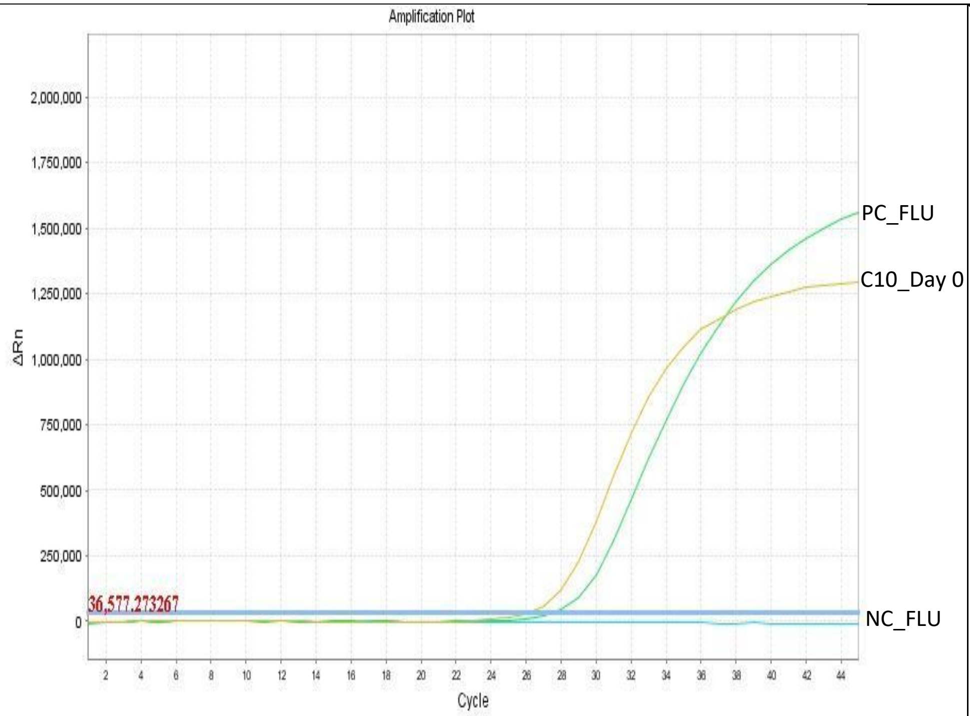Amplification Plot
2,000,000
1,750,000
1,500,000
1,250,000
1,000,000
750,000
500,000
250,000
0
ΔRn
36,577.273267
2
4
6
8
10
12
14
16
18
20
22
24
26
28
30
32
34
36
38
40
42
44
Cycle
PC_FLU
C10_Day 0
NC_FLU	

	C10_D2	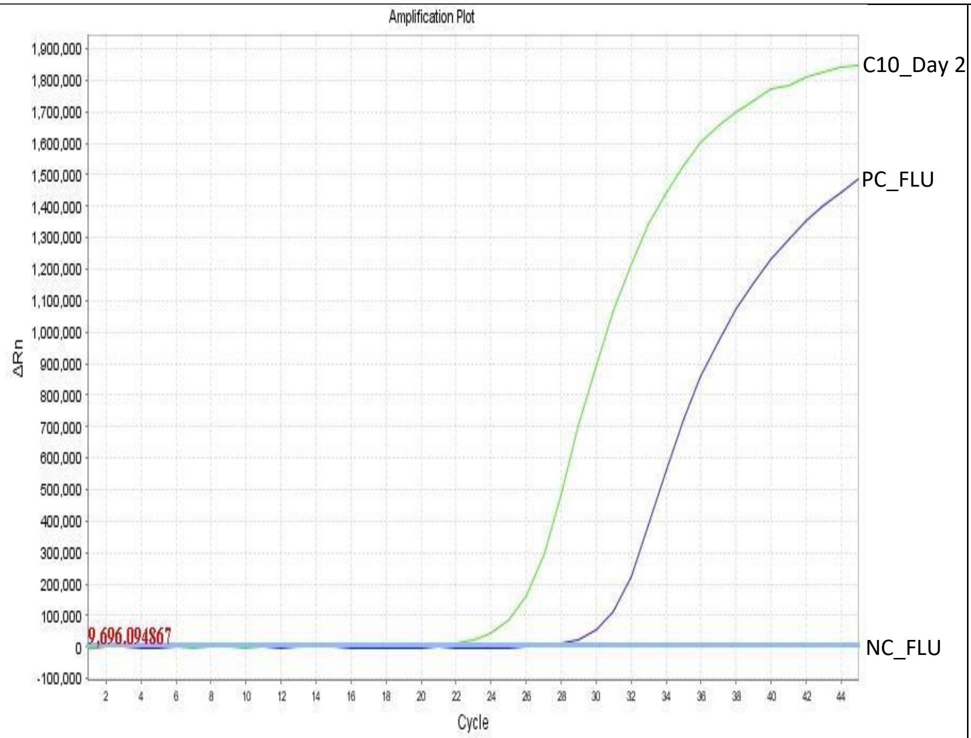 Amplification Plot<br>ΔRn<br>Cycle<br>C10_Day 2<br>PC_FLU<br>NC_FLU<br>9,696.094867	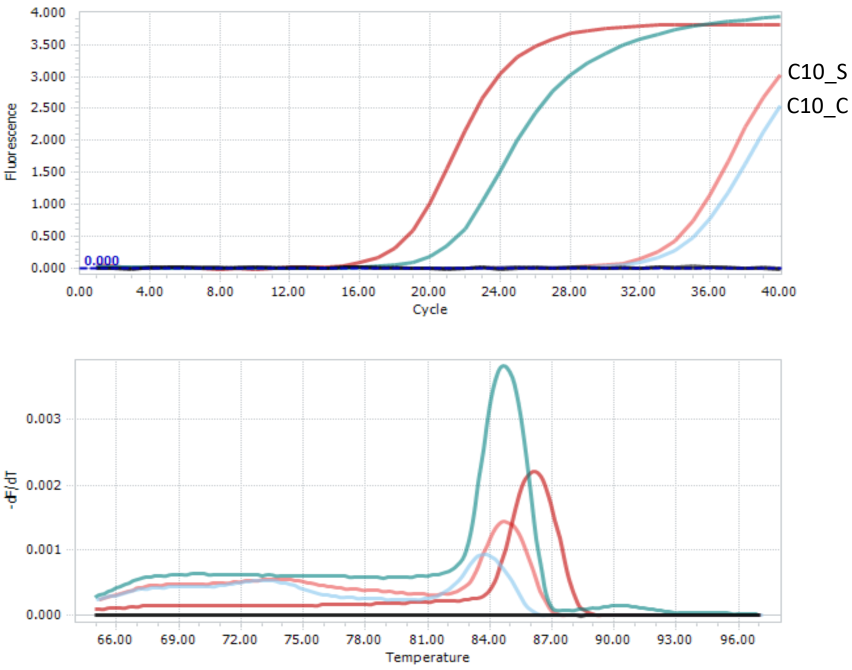 Fluorescence<br>Cycle<br>C10_S<br>C10_C<br>0.000<br>-dF/dT<br>Temperature

10	C11_D0	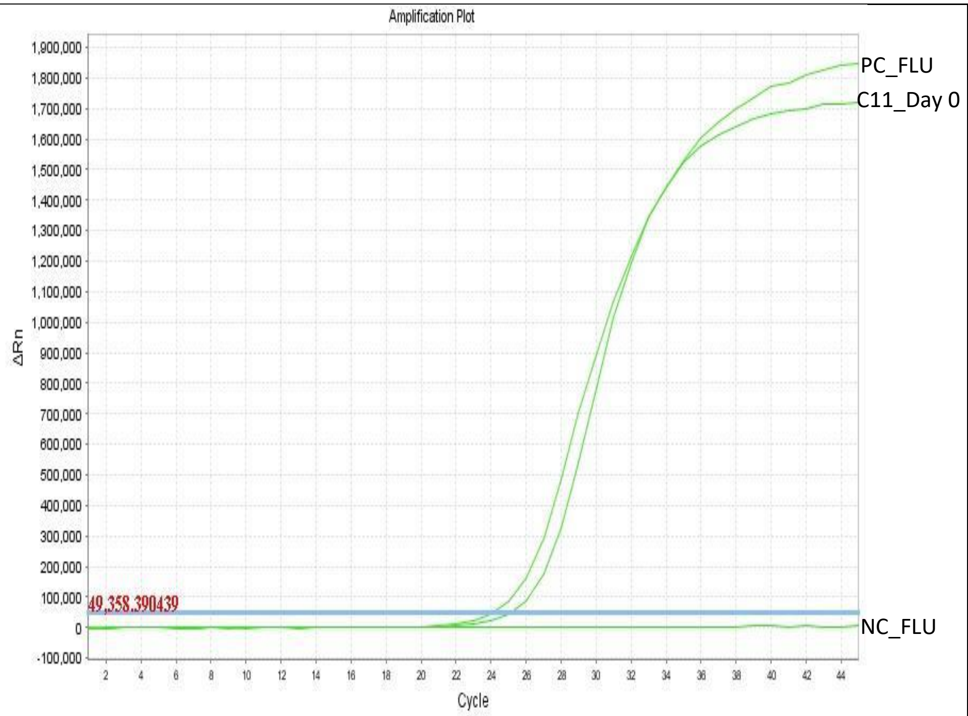Amplification Plot<br>PC_FLU<br>C11_Day 0<br>NC_FLU<br>49,358.390439<br>ΔRn<br>Cycle	

	C11_D2	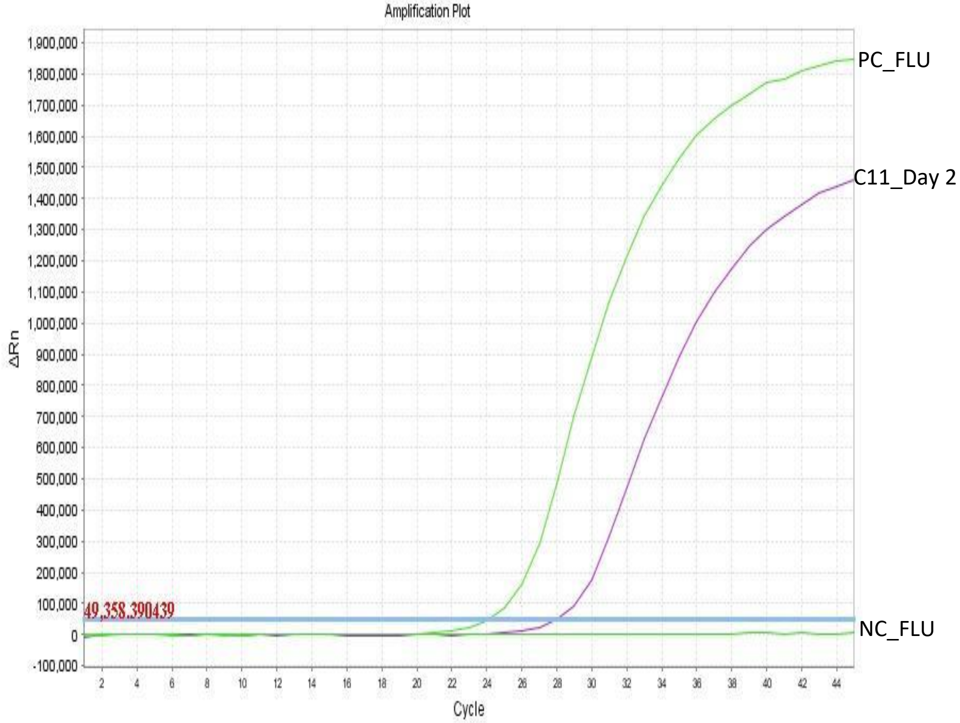	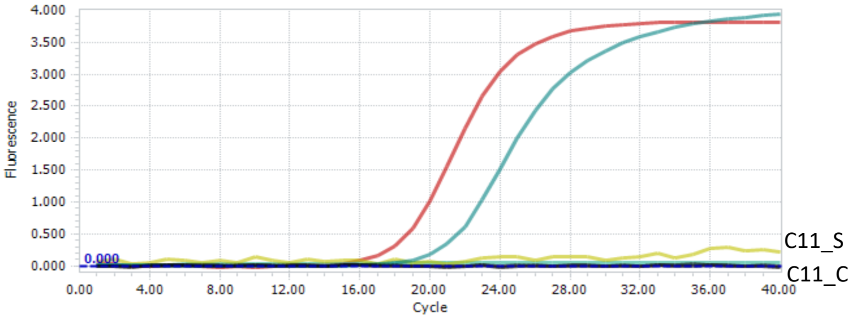 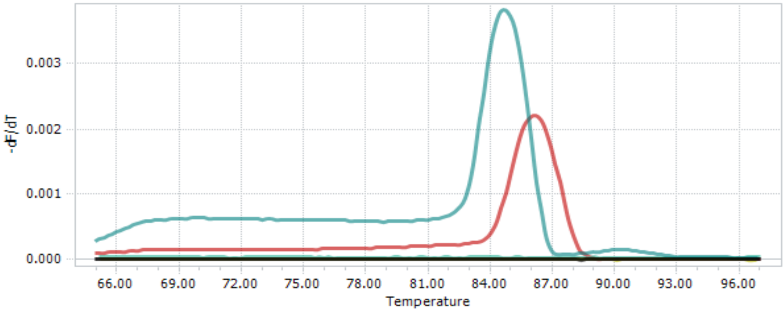

Amplification Plot
ΔRn
Cycle
49,358.390439
PC_FLU
C11_Day 2
NC_FLU

Fluorescence
Cycle
0.000
C11_S
C11_C

-dF/dT
Temperature

11	C12_D0	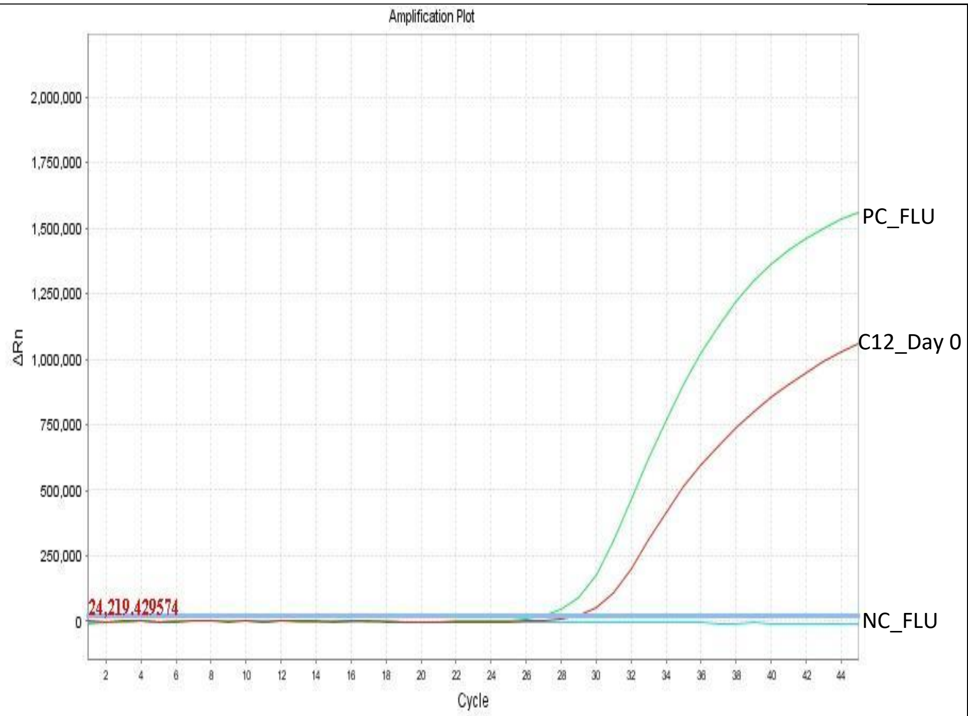Amplification Plot
2,000,000
1,750,000
1,500,000
1,250,000
1,000,000
750,000
500,000
250,000
0
ΔRn
Cycle
24,219.429574
PC_FLU
C12_Day 0
NC_FLU	

	C12_D2	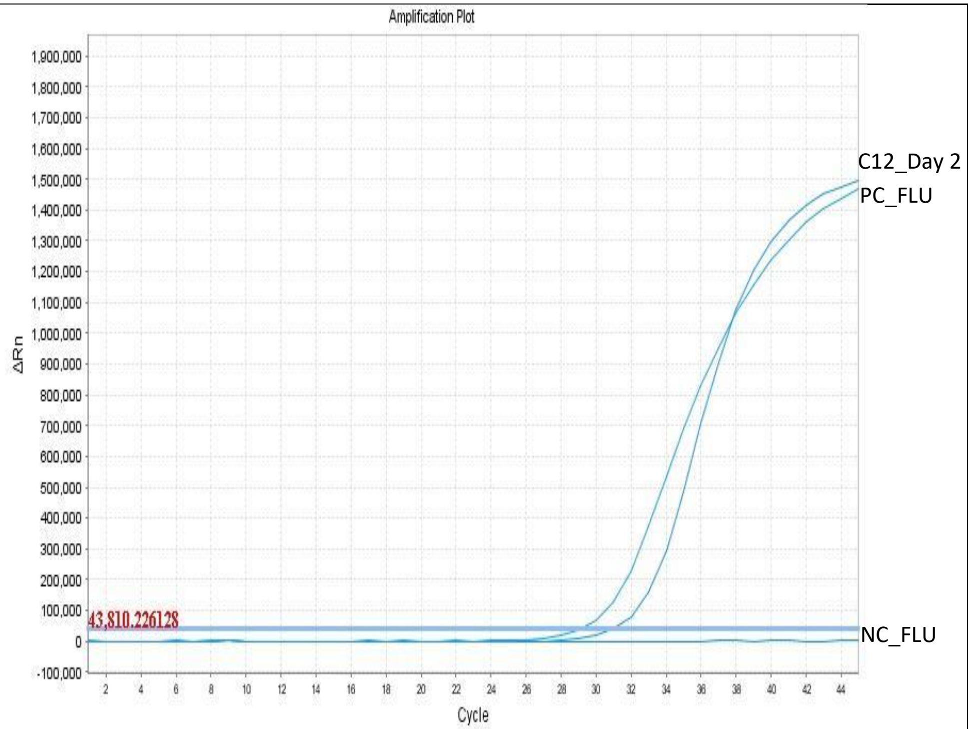	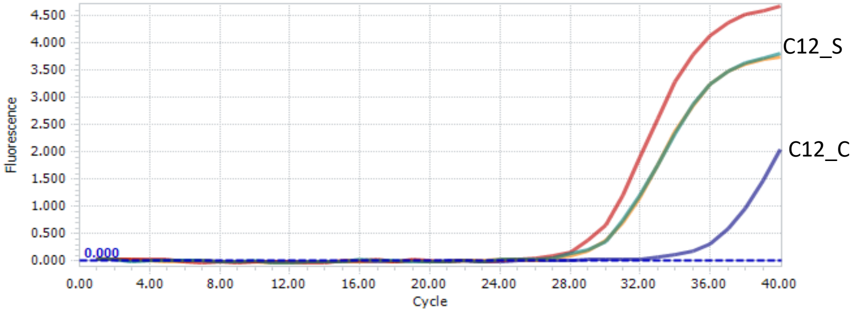 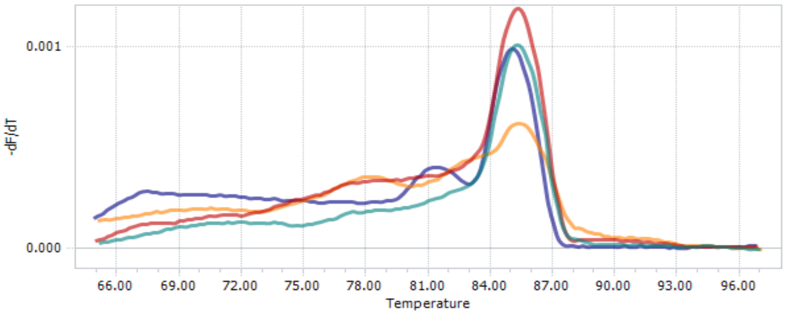

Amplification Plot
ΔRn
1,900,000
1,800,000
1,700,000
1,600,000
1,500,000
1,400,000
1,300,000
1,200,000
1,100,000
1,000,000
900,000
800,000
700,000
600,000
500,000
400,000
300,000
200,000
100,000
0
-100,000
43,810.226128
2 4 6 8 10 12 14 16 18 20 22 24 26 28 30 32 34 36 38 40 42 44
Cycle
C12_Day 2
PC_FLU
NC_FLU

Fluorescence
4.500
4.000
3.500
3.000
2.500
2.000
1.500
1.000
0.500
0.000
0.000
0.00 4.00 8.00 12.00 16.00 20.00 24.00 28.00 32.00 36.00 40.00
Cycle
C12_S
C12_C

-dF/dT
0.001
0.000
66.00 69.00 72.00 75.00 78.00 81.00 84.00 87.00 90.00 93.00 96.00
Temperature

12	C13_D0	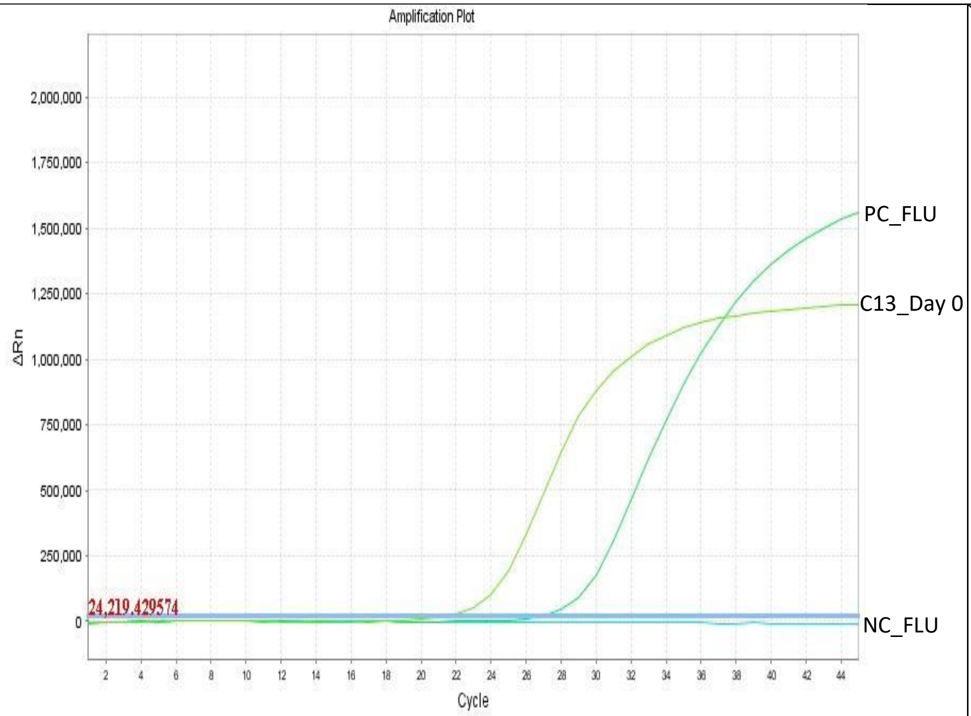Amplification Plot<br>ΔRn<br>2,000,000<br>1,750,000<br>1,500,000<br>1,250,000<br>1,000,000<br>750,000<br>500,000<br>250,000<br>0<br>24,219.429574<br>Cycle<br>PC_FLU<br>C13_Day 0<br>NC_FLU	

	C13 _D2	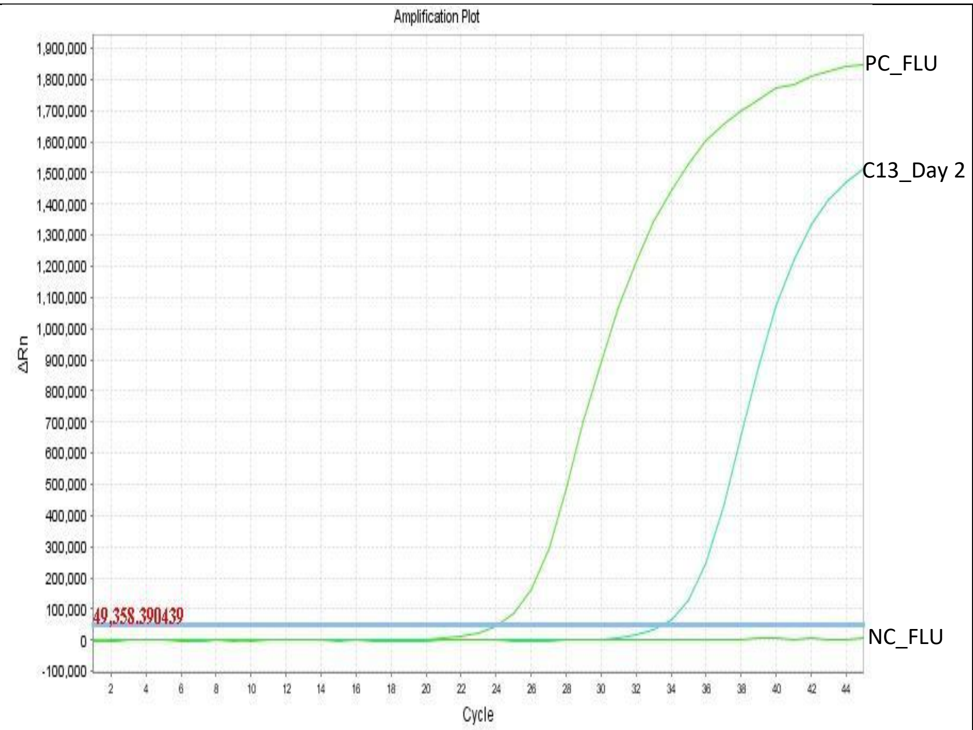	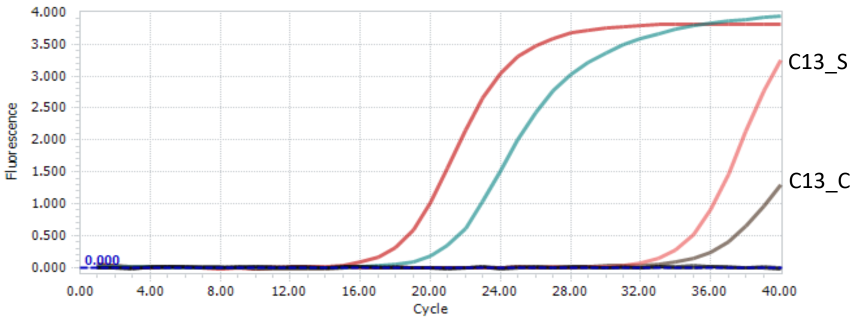 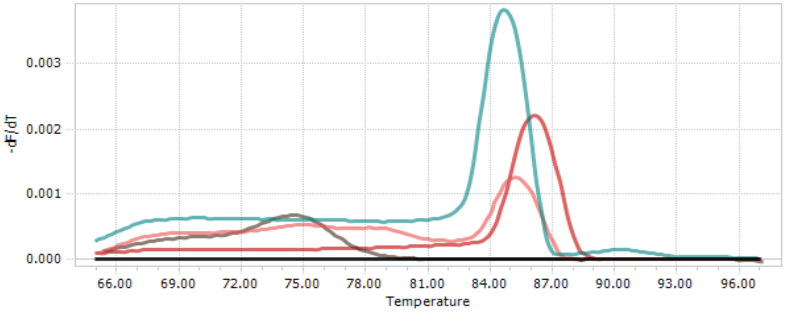

Amplification Plot
ΔRn
Cycle
49,358.390439
PC_FLU
C13_Day 2
NC_FLU

Fluorescence
Cycle
0.000
C13_S
C13_C

-dF/dT
Temperature

13	C14_D0	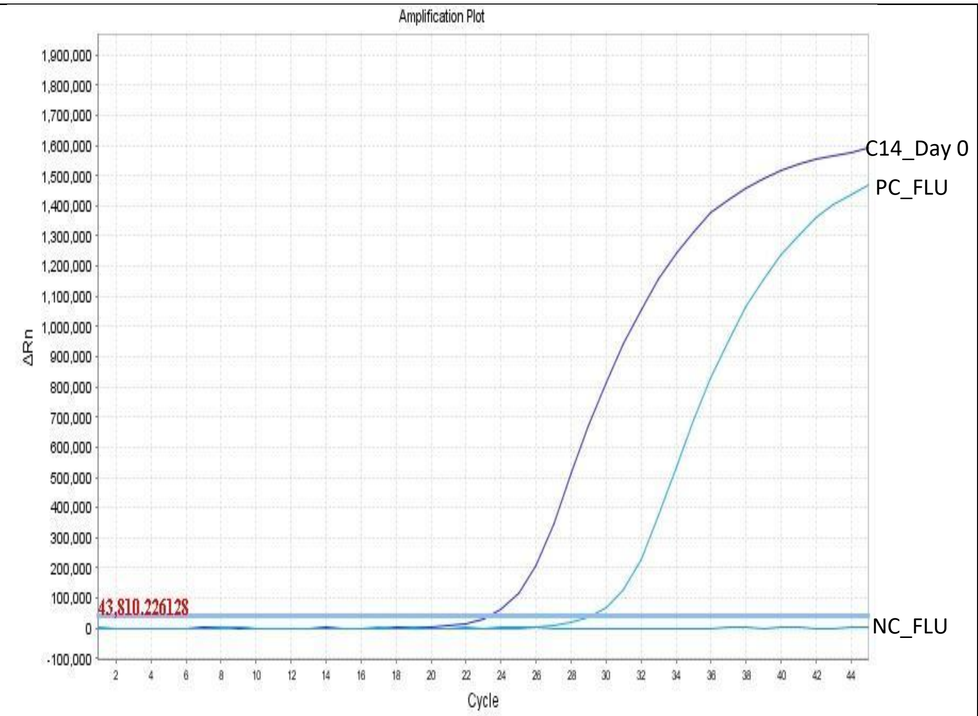Amplification Plot
1,900,000
1,800,000
1,700,000
1,600,000
1,500,000
1,400,000
1,300,000
1,200,000
1,100,000
1,000,000
900,000
800,000
700,000
600,000
500,000
400,000
300,000
200,000
100,000
0
-100,000
ΔRn
43,810.226128
2 4 6 8 10 12 14 16 18 20 22 24 26 28 30 32 34 36 38 40 42 44
Cycle
C14_Day 0
PC_FLU
NC_FLU	

	C14_D2	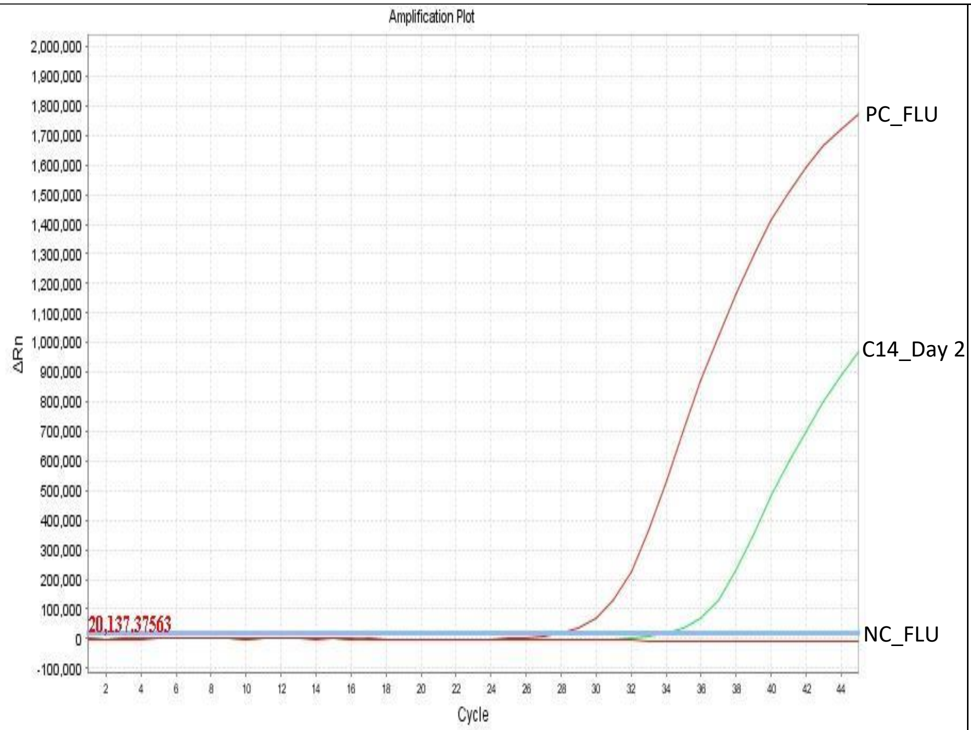	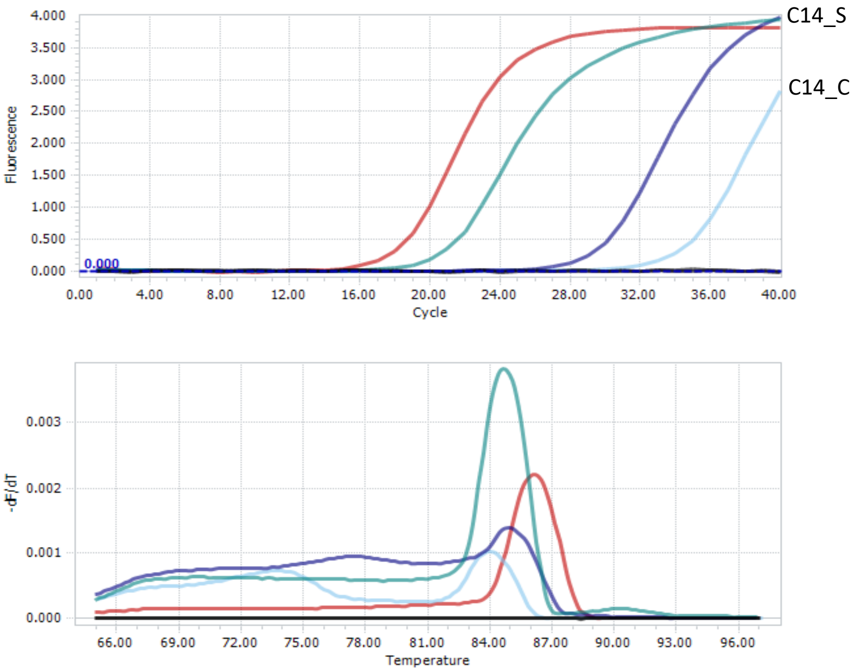

Amplification Plot
ΔRn
Cycle
20,137.37563
PC_FLU
C14_Day 2
NC_FLU

Fluorescence
Cycle
0.000
C14_S
C14_C
-dF/dT
Temperature

14	C15_D0	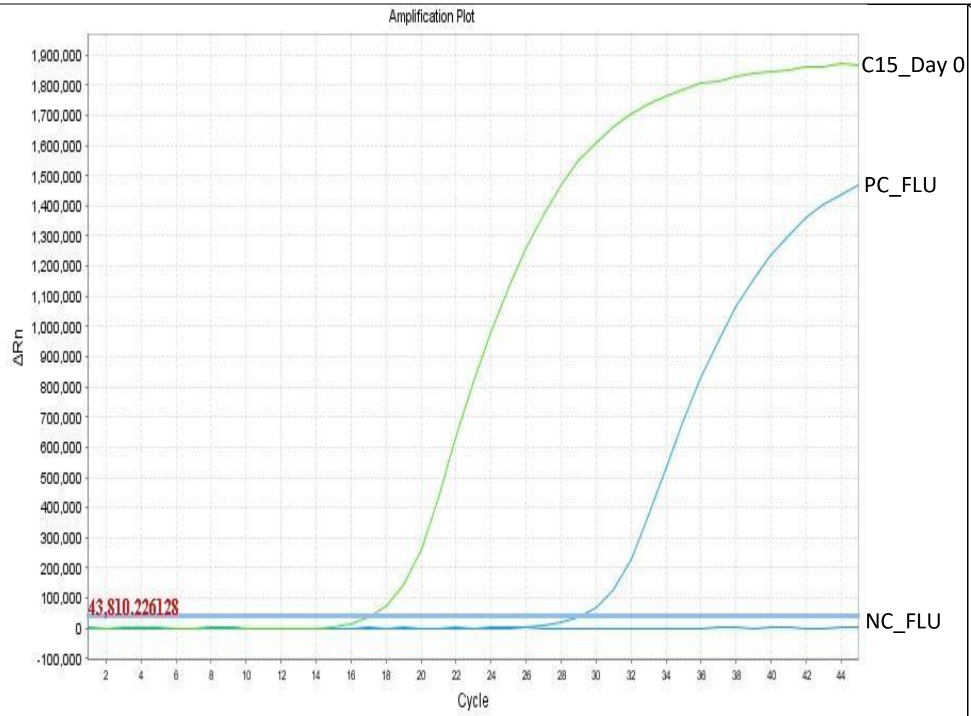Amplification Plot
ΔRn
Cycle
43,810.226128
C15_Day 0
PC_FLU
NC_FLU	

	C15 _D2	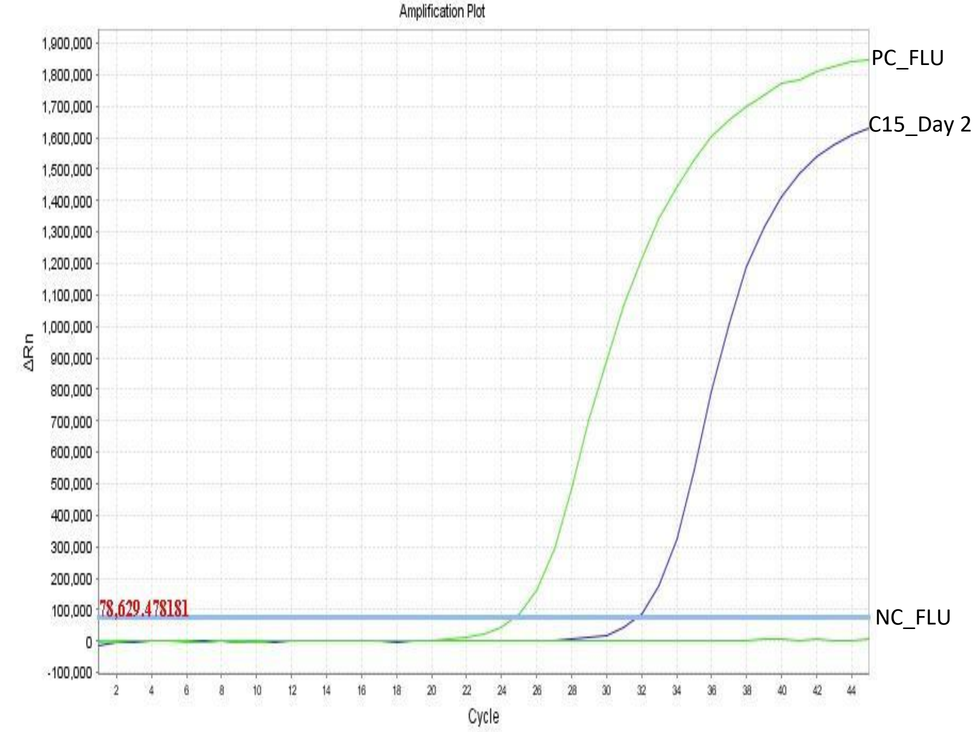 Amplification Plot<br>ΔRn<br>Cycle<br>78,629.478181<br>PC_FLU<br>C15_Day 2<br>NC_FLU 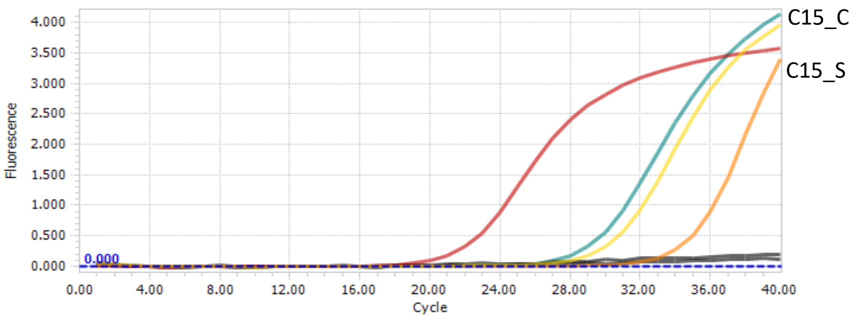 Fluorescence<br>Cycle<br>0.000<br>C15_C<br>C15_S 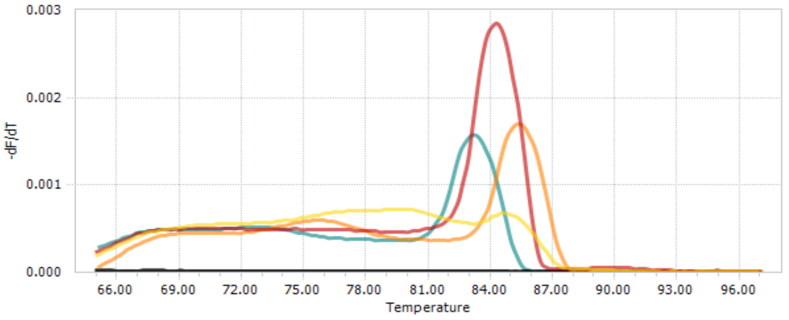 -dF/dT<br>Temperature

15	C16_D0	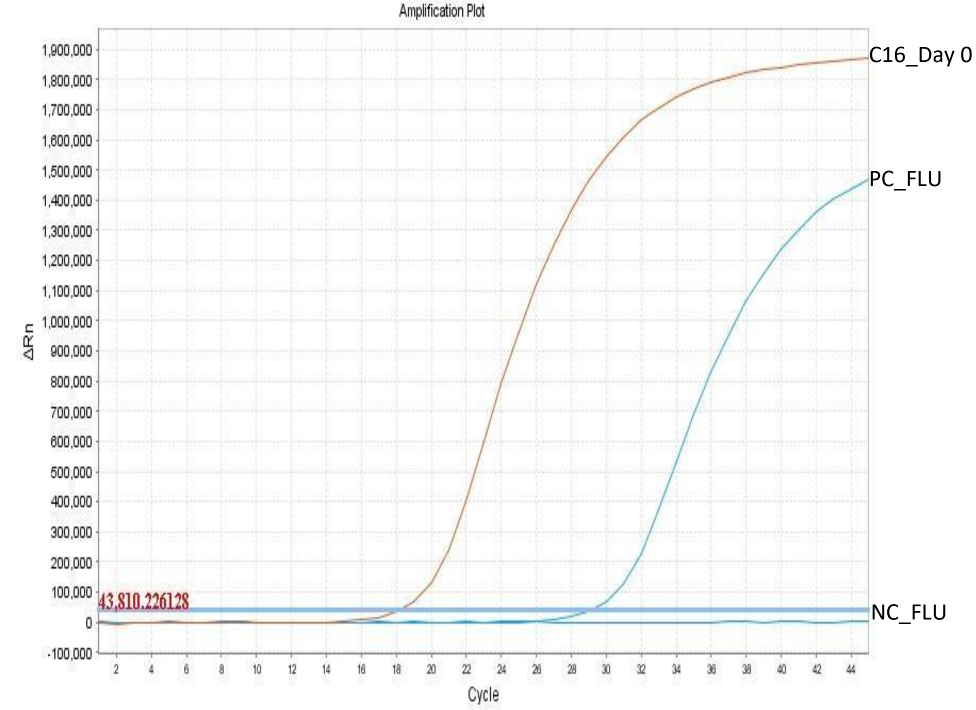Amplification Plot
C16_Day 0
PC_FLU
NC_FLU
43,810.226128
ΔRn
Cycle	

	C16_D2	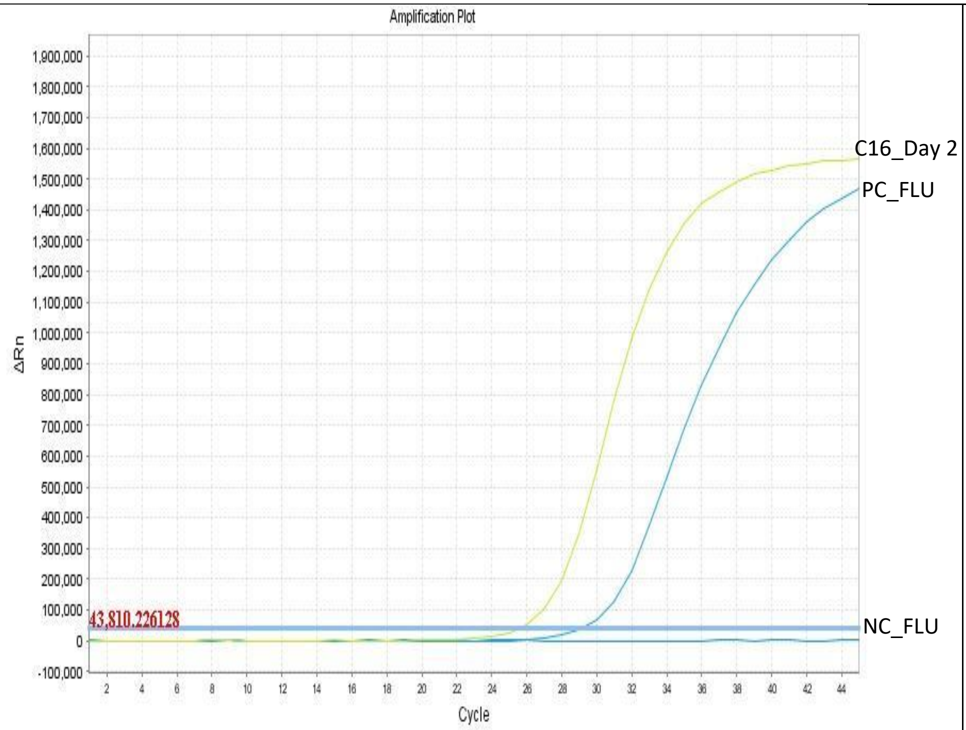	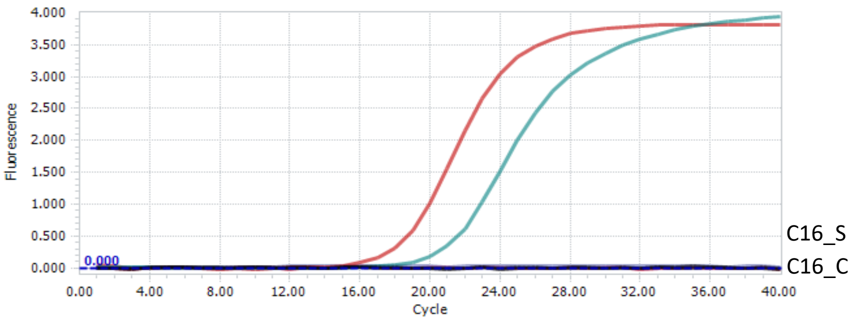 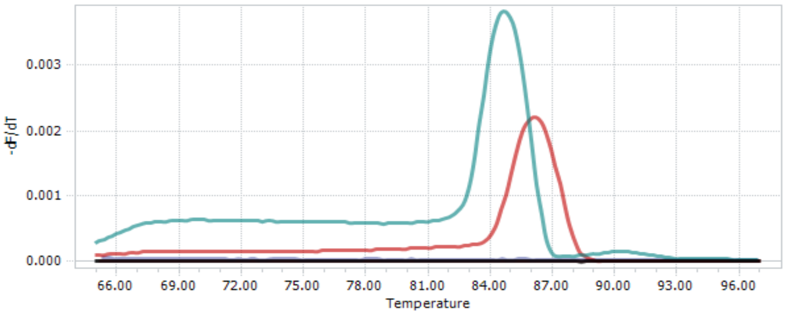

Amplification Plot
ΔRn
Cycle
43,810.226128
C16_Day 2
PC_FLU
NC_FLU

Fluorescence
Cycle
0.000
C16_S
C16_C

-dF/dT
Temperature

16	C18_D0	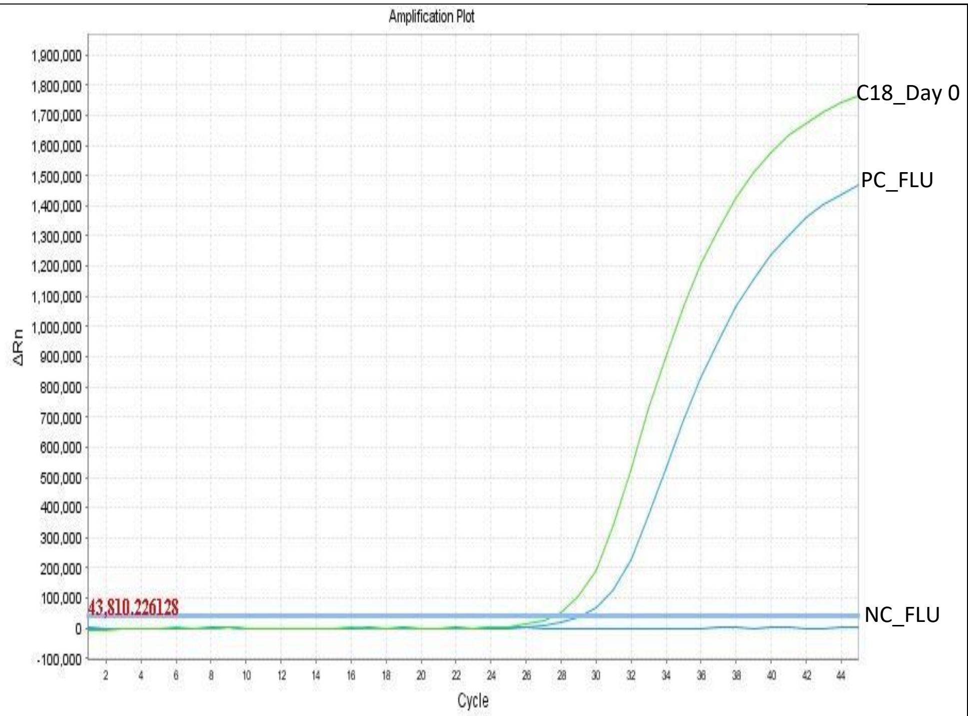Amplification Plot<br>C18_Day 0<br>PC_FLU<br>NC_FLU<br>43,810.226128<br>ΔRn<br>Cycle	

C18_D2

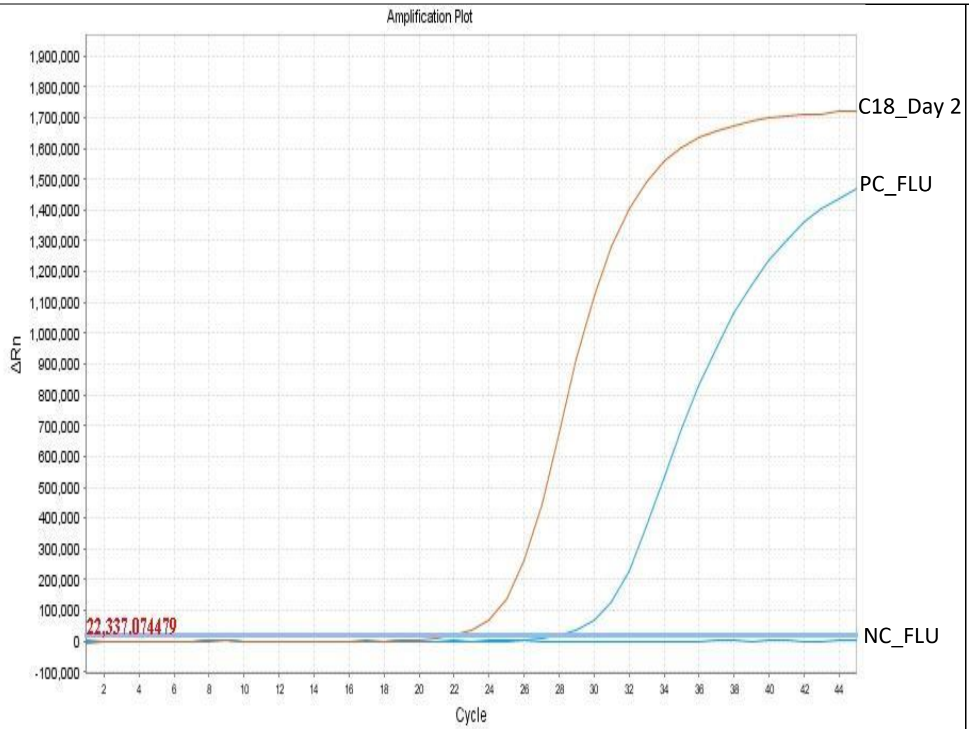
Amplification Plot
ΔRn
Cycle
22,337.074479
C18_Day 2
PC_FLU
NC_FLU

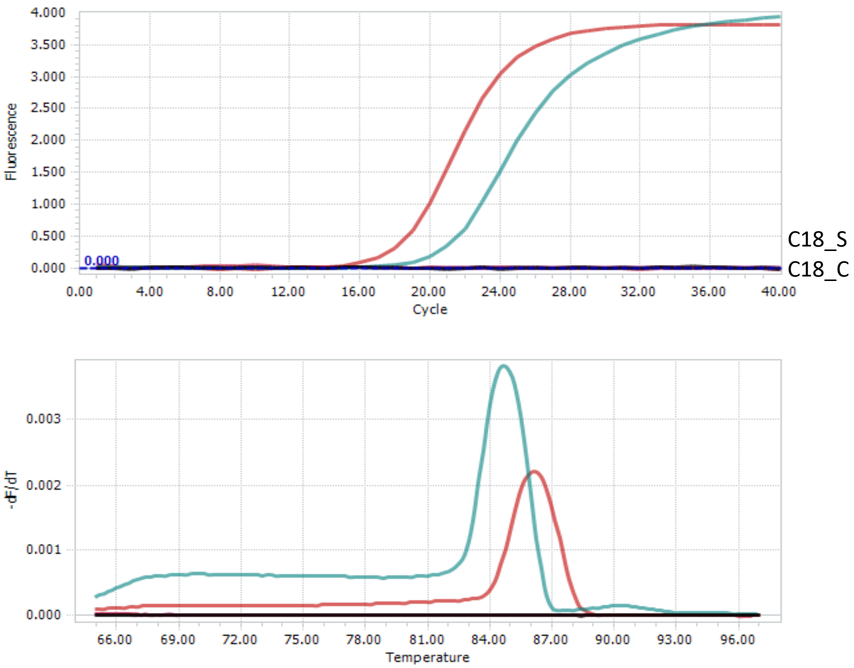
Fluorescence
Cycle
0.000
C18_S
C18_C
-dF/dT
Temperature

17	C20_D0	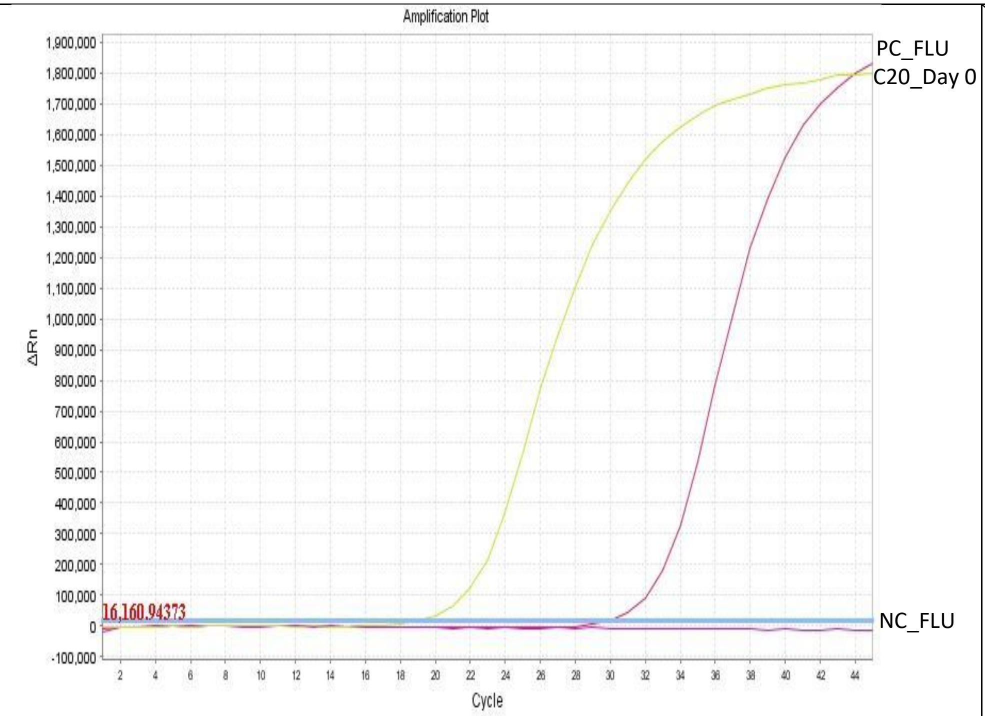Amplification Plot<br>PC_FLU<br>C20_Day 0<br>NC_FLU<br>16,160.94373<br>ΔRn<br>Cycle	

<table>
<tr>
<td></td>
<td>C20_D2</td>
<td>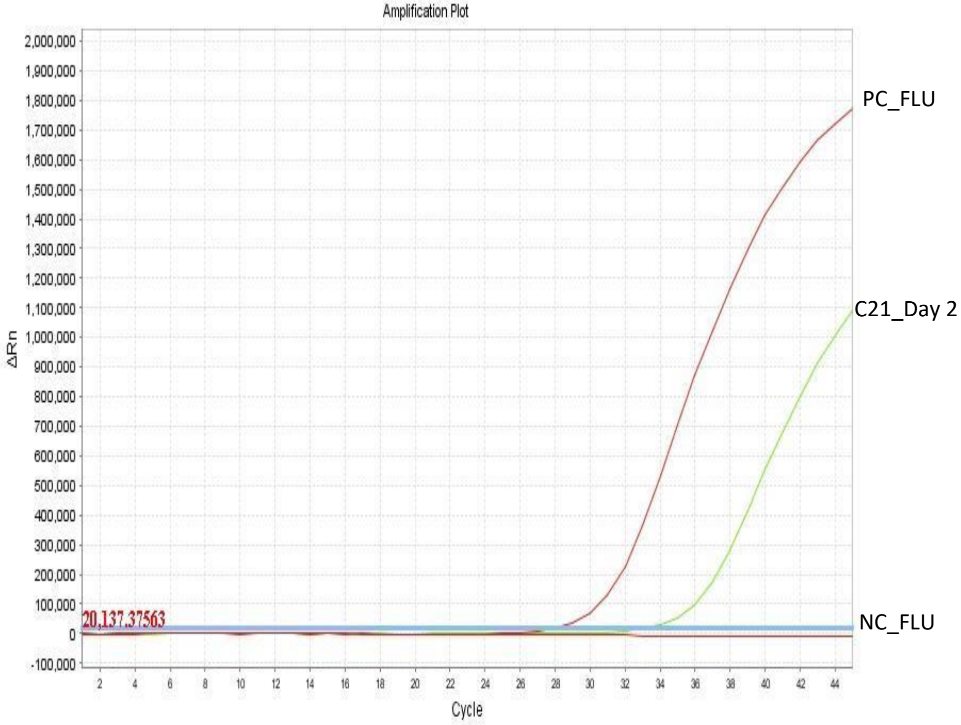
</td>
<td>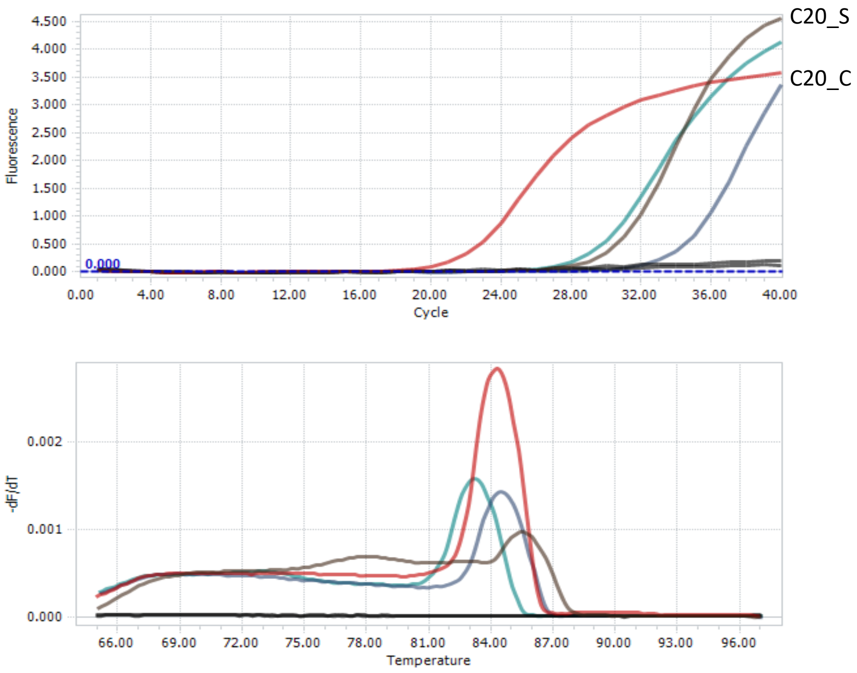
</td>
</tr>
</table>

18	C21_D0	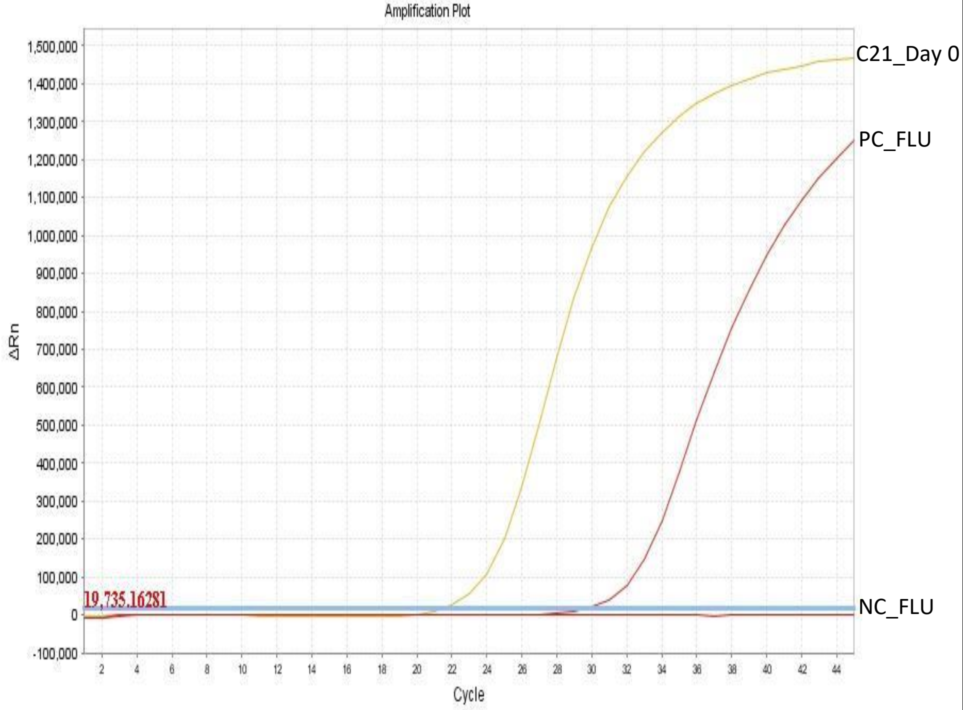Amplification Plot<br>C21_Day 0<br>PC_FLU<br>NC_FLU<br>19,735.16281<br>ΔRn<br>Cycle<br>1,500,000<br>1,400,000<br>1,300,000<br>1,200,000<br>1,100,000<br>1,000,000<br>900,000<br>800,000<br>700,000<br>600,000<br>500,000<br>400,000<br>300,000<br>200,000<br>100,000<br>0<br>-100,000<br>2 4 6 8 10 12 14 16 18 20 22 24 26 28 30 32 34 36 38 40 42 44	

C21_D2

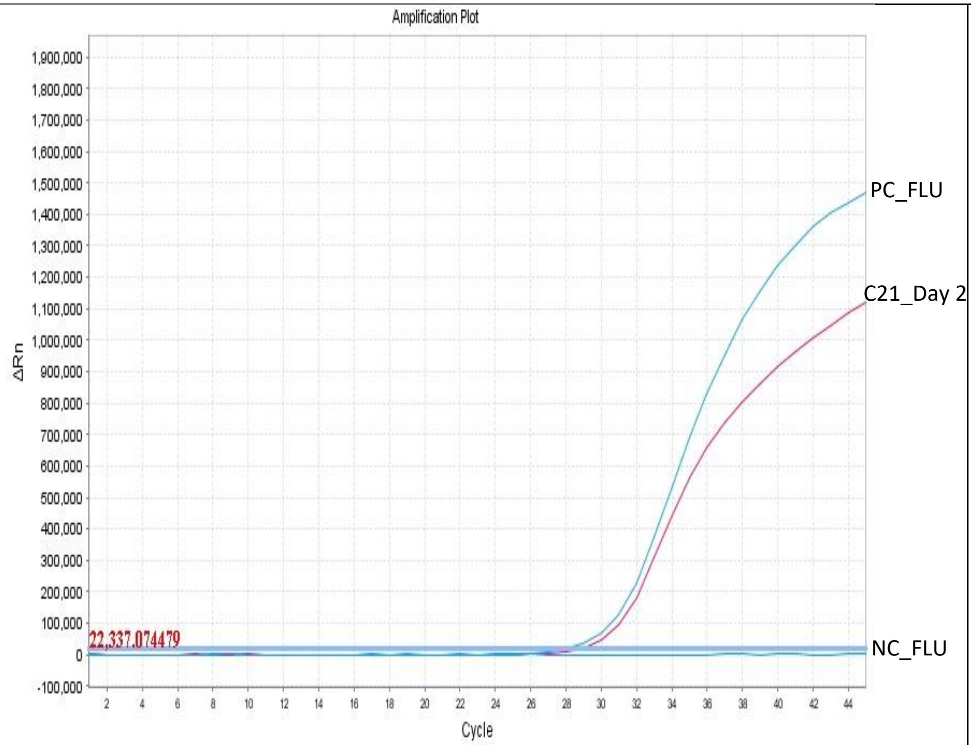
Amplification Plot
ΔRn
Cycle
22,337.074479
PC_FLU
C21_Day 2
NC_FLU

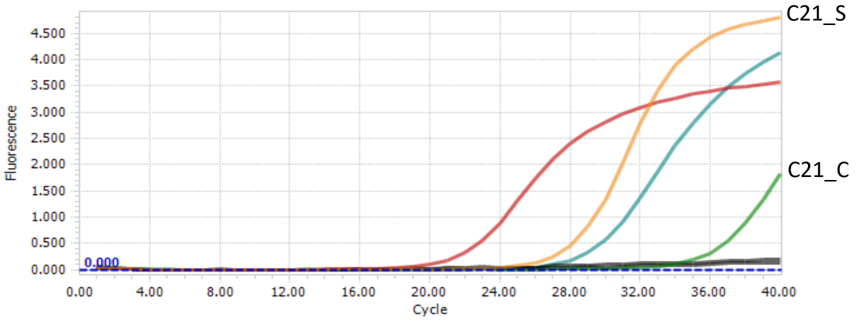
Fluorescence
Cycle
0.000
C21_S
C21_C

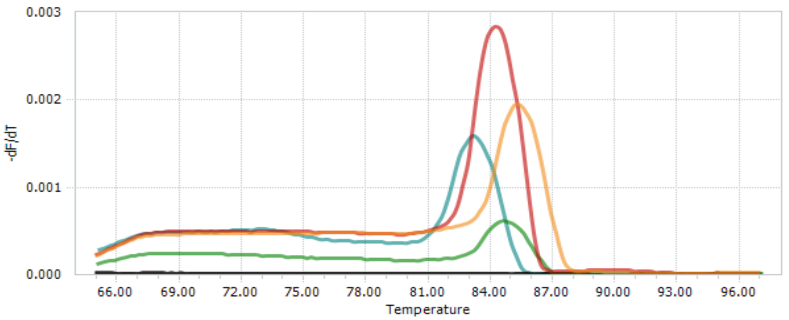
-dF/dT
Temperature

19	C22_D0	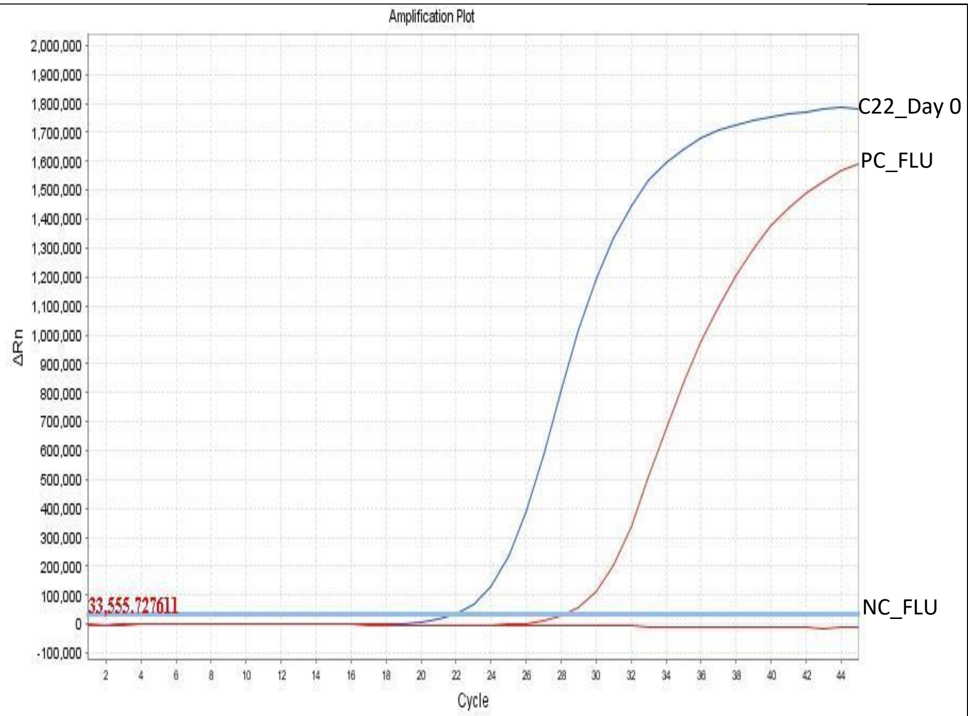Amplification Plot<br>ΔRn<br>Cycle<br>33,555.727611<br>C22_Day 0<br>PC_FLU<br>NC_FLU	

	C22_D2	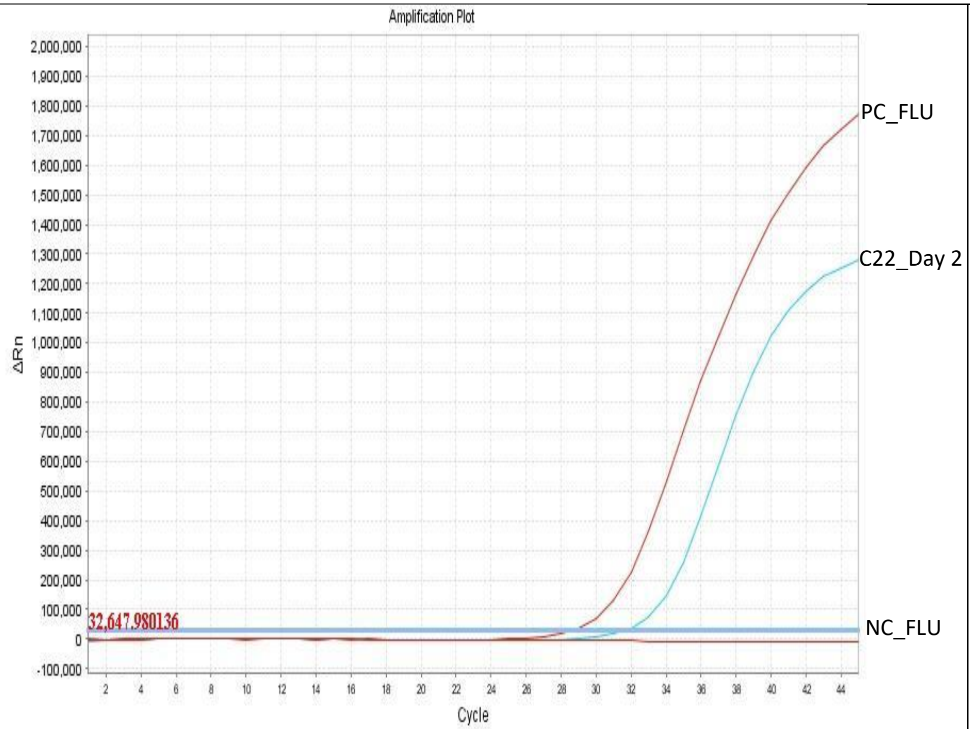	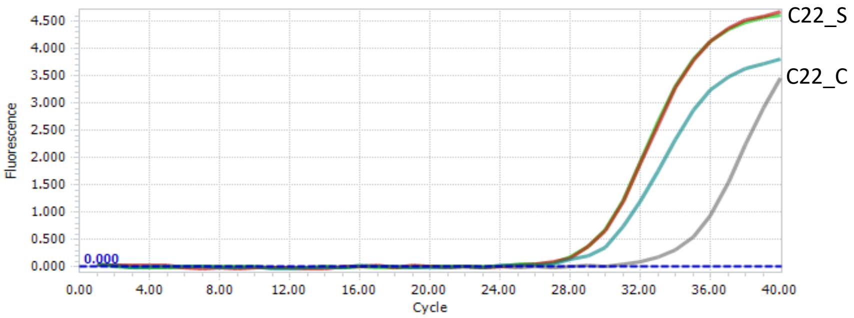 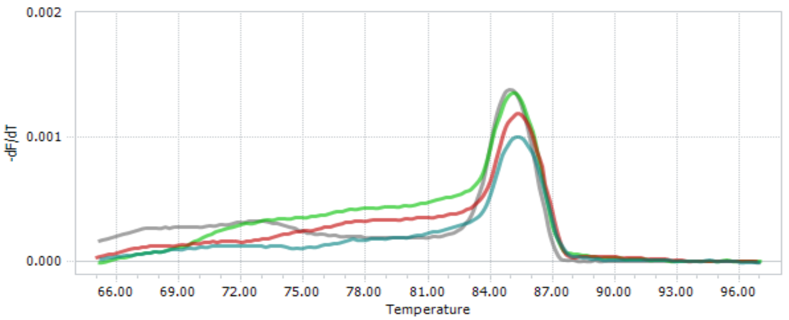

Amplification Plot
ΔRn
Cycle
32,647.980136
PC_FLU
C22_Day 2
NC_FLU

Fluorescence
Cycle
0.000
C22_S
C22_C

-dF/dT
Temperature

20	C23_D0	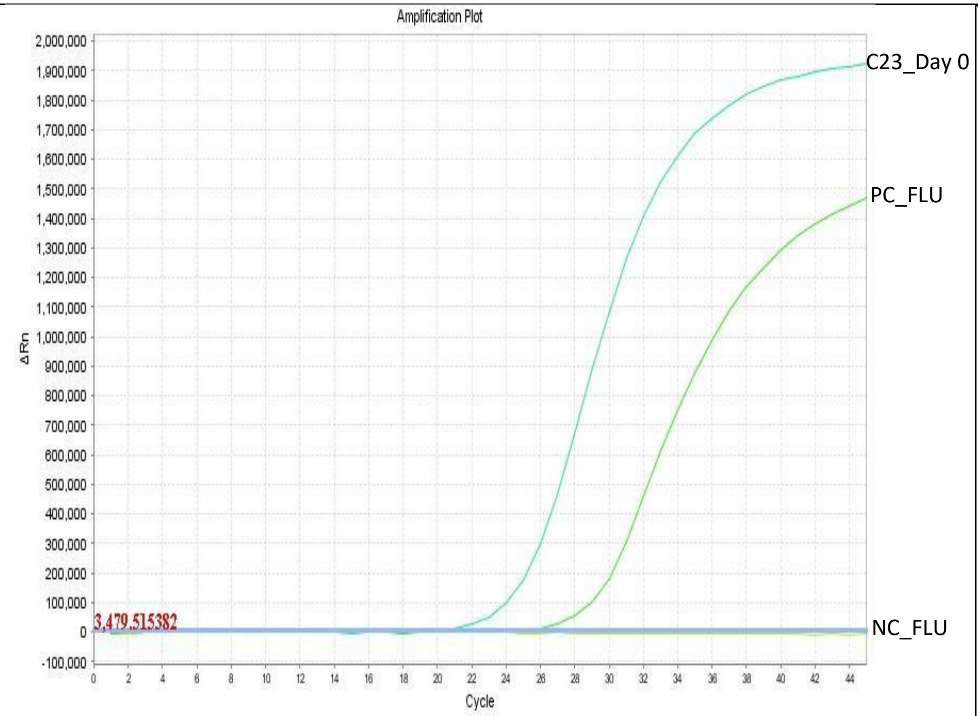Amplification Plot
C23_Day 0
PC_FLU
NC_FLU
3,479.515382
ΔRn
Cycle	

	C23_D2	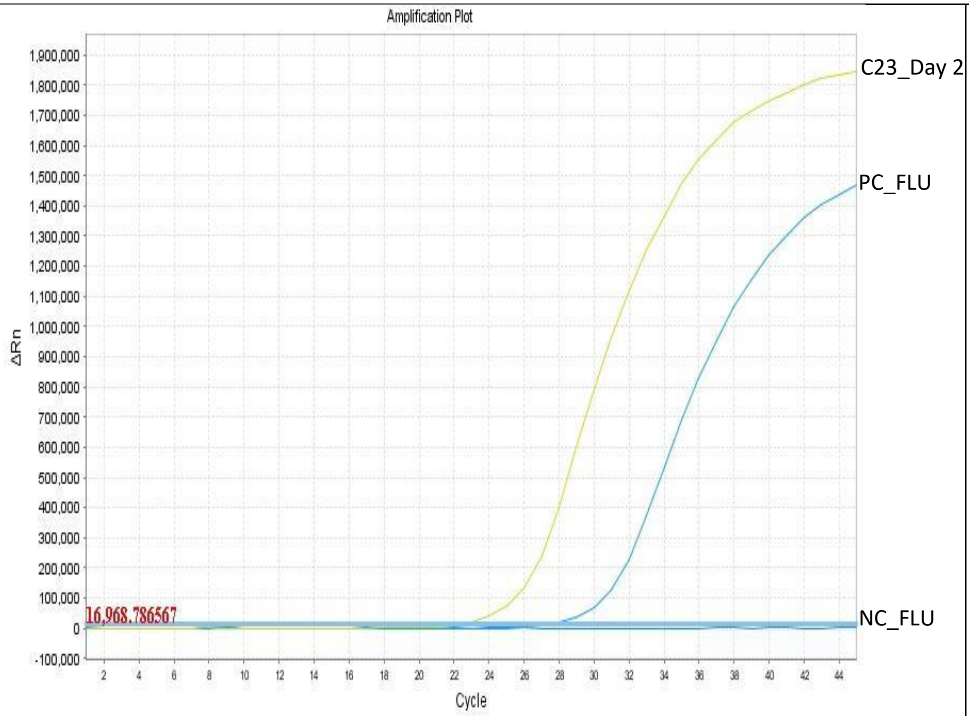 Amplification Plot<br>C23_Day 2<br>PC_FLU<br>NC_FLU<br>16,968.786567<br>ΔRn<br>Cycle	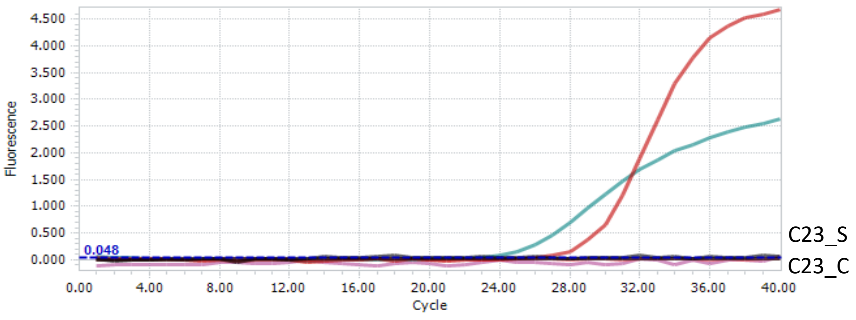 Fluorescence<br>Cycle<br>0.048<br>C23_S<br>C23_C 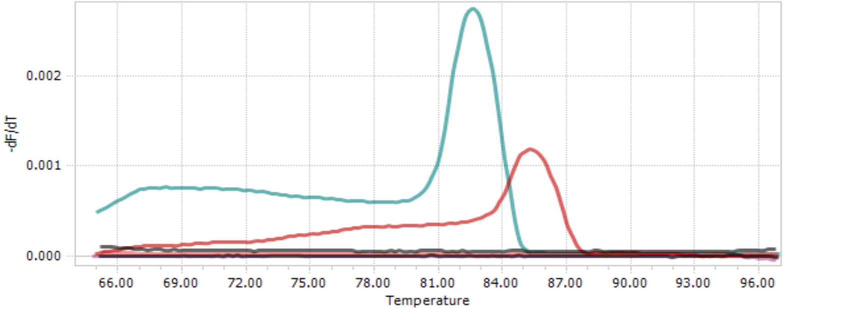 -dF/dT<br>Temperature

21	C24_D0	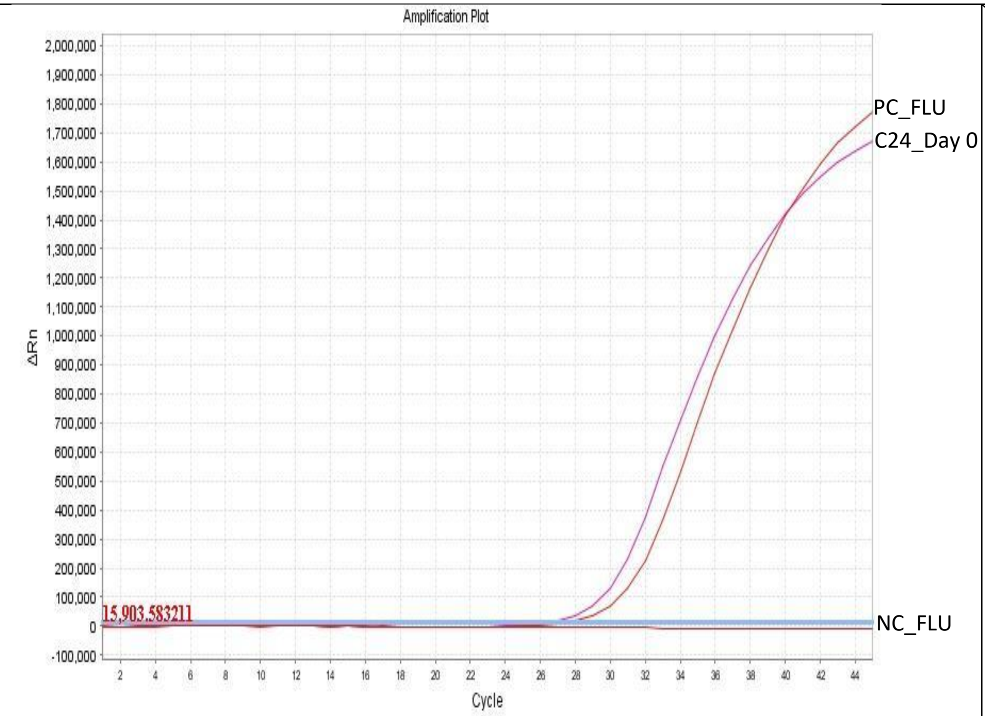Amplification Plot<br>PC_FLU<br>C24_Day 0<br>NC_FLU<br>15,903.583211<br>ΔRn<br>Cycle	

	C24_D2	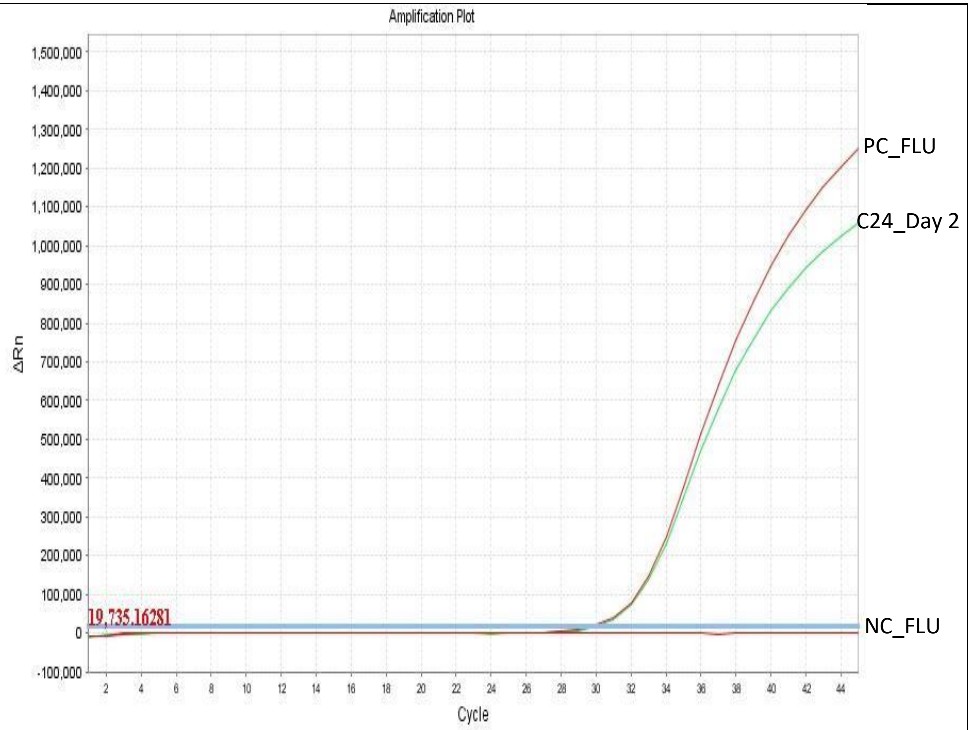	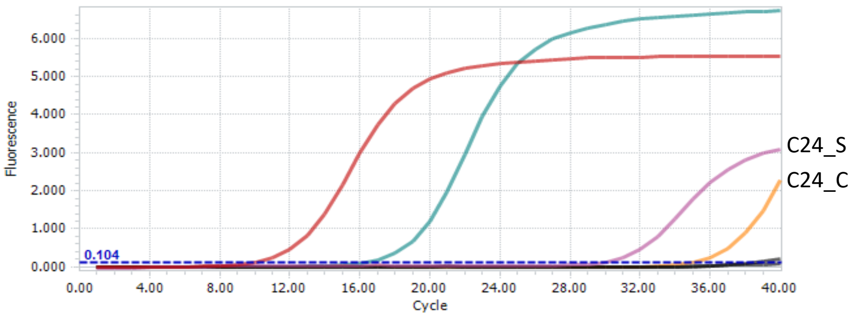 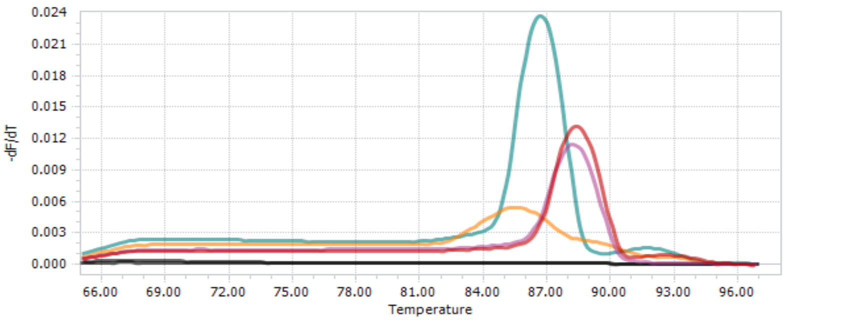

Amplification Plot
ΔRn
Cycle
19,735.16281
PC_FLU
C24_Day 2
NC_FLU

Fluorescence
Cycle
0.104
C24_S
C24_C

-dF/dT
Temperature

22	C25_D0	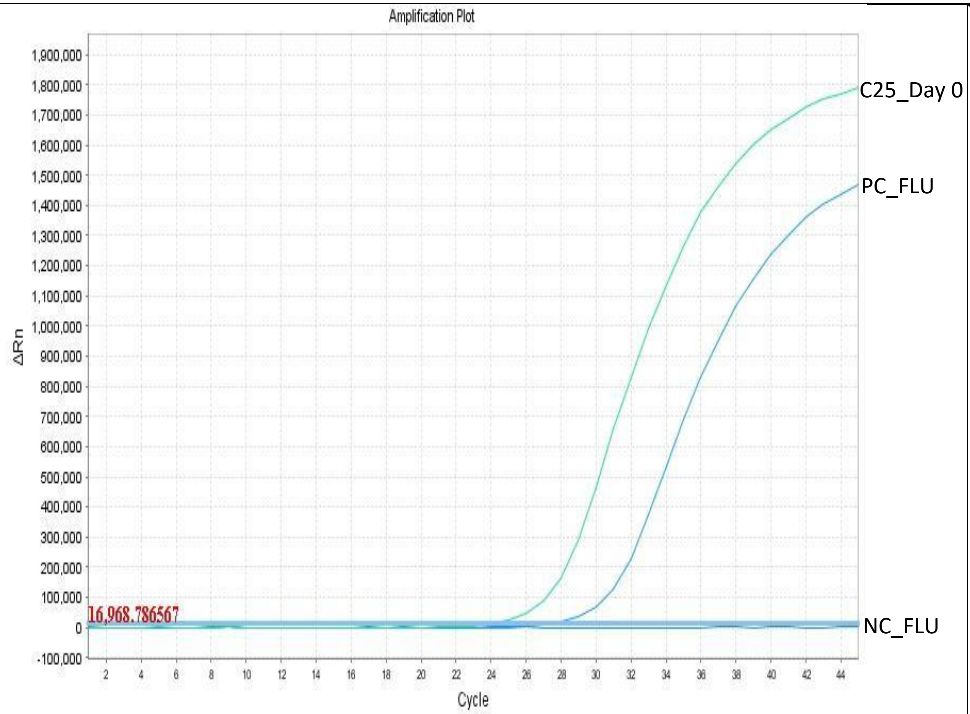Amplification Plot
1,900,000
1,800,000
1,700,000
1,600,000
1,500,000
1,400,000
1,300,000
1,200,000
1,100,000
1,000,000
900,000
800,000
700,000
600,000
500,000
400,000
300,000
200,000
100,000
0
-100,000
ΔRn
16,968.786567
2 4 6 8 10 12 14 16 18 20 22 24 26 28 30 32 34 36 38 40 42 44
Cycle
C25_Day 0
PC_FLU
NC_FLU	

	C25_D2	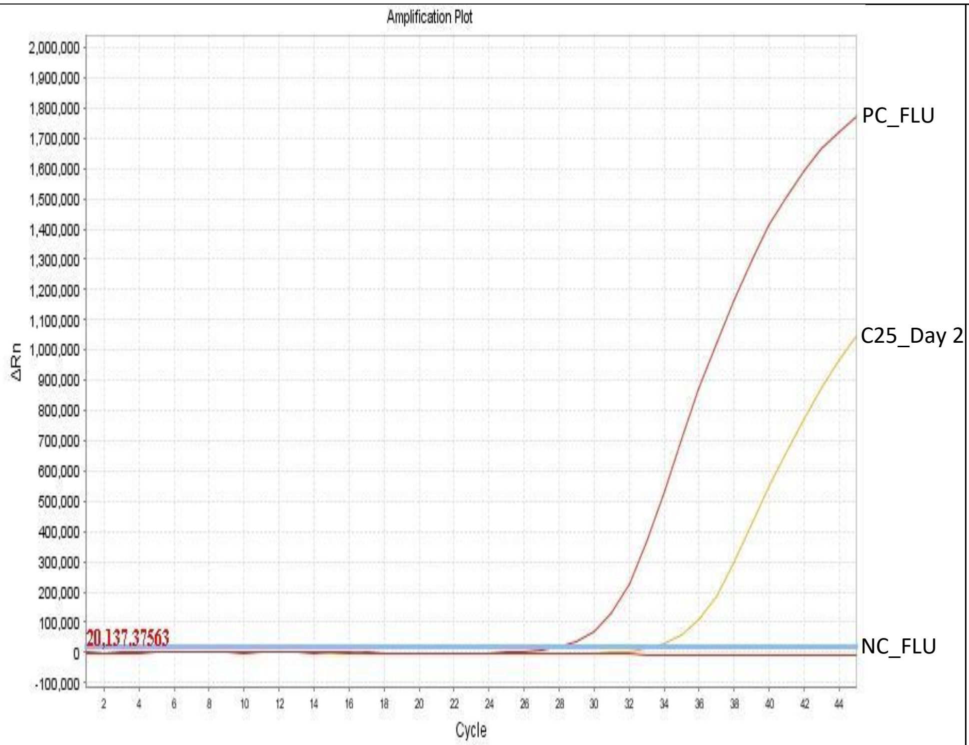	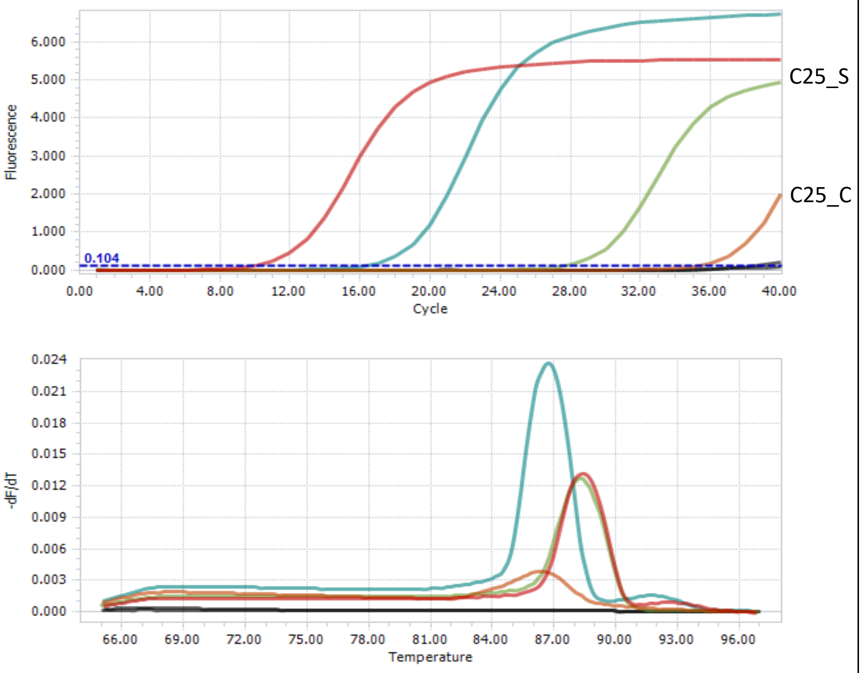

Amplification Plot
ΔRn
Cycle
20,137.37563
PC_FLU
C25_Day 2
NC_FLU

Fluorescence
Cycle
0.104
C25_S
C25_C
-dF/dT
Temperature

23	C26_D0	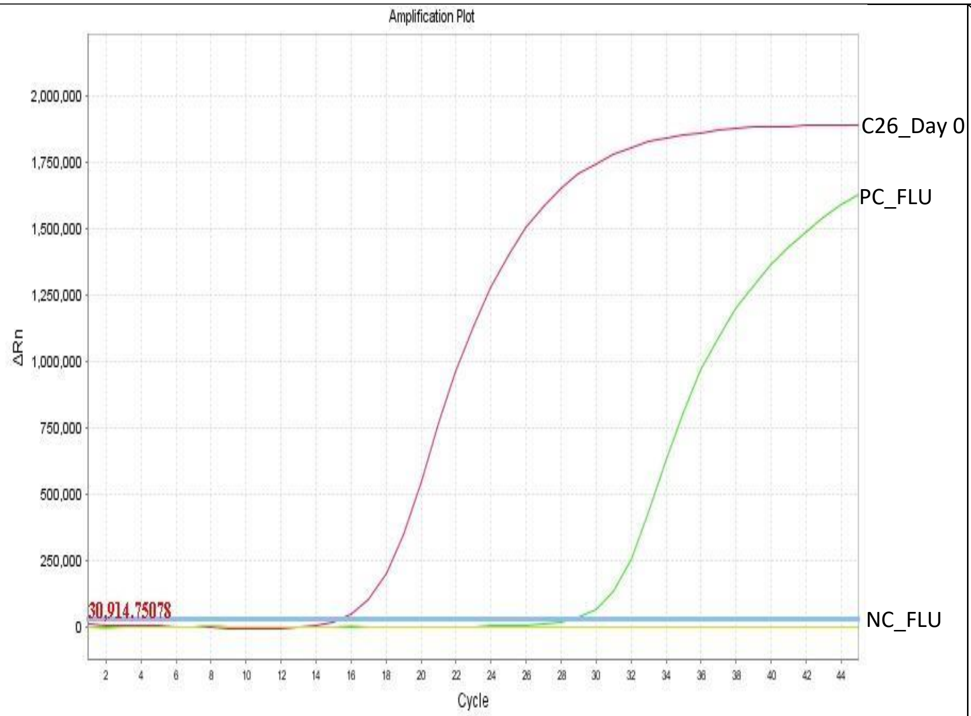Amplification Plot
2,000,000
1,750,000
1,500,000
1,250,000
1,000,000
750,000
500,000
250,000
0
ΔRn
30,914.75078
2
4
6
8
10
12
14
16
18
20
22
24
26
28
30
32
34
36
38
40
42
44
Cycle
C26_Day 0
PC_FLU
NC_FLU	

C26_D2

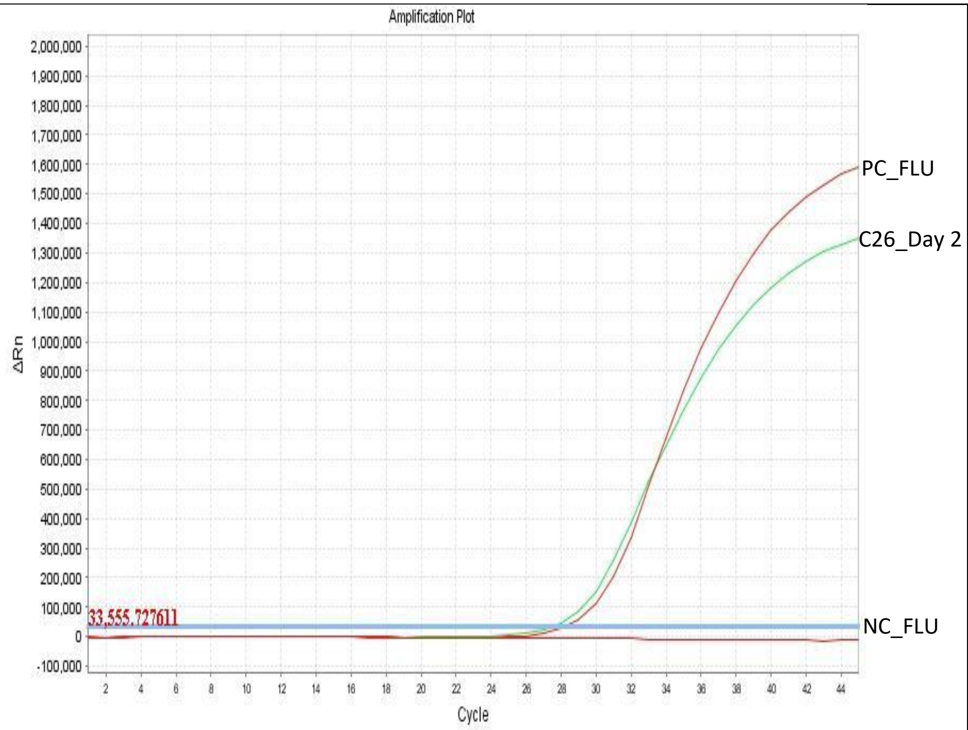
Amplification Plot
ΔRn
Cycle
33,555.727611
PC_FLU
C26_Day 2
NC_FLU

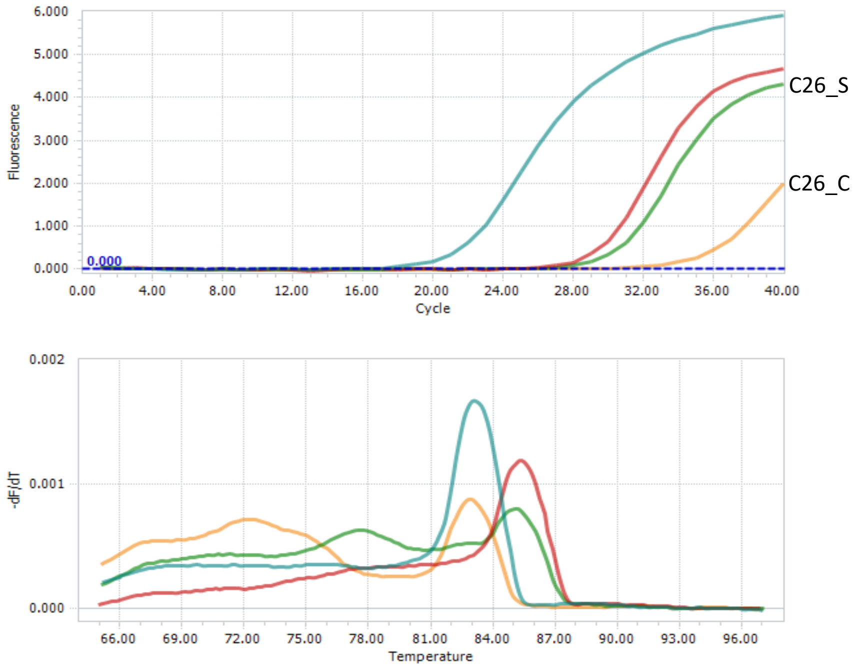
Fluorescence
Cycle
0.000
C26_S
C26_C
-dF/dT
Temperature

24	C27_D0	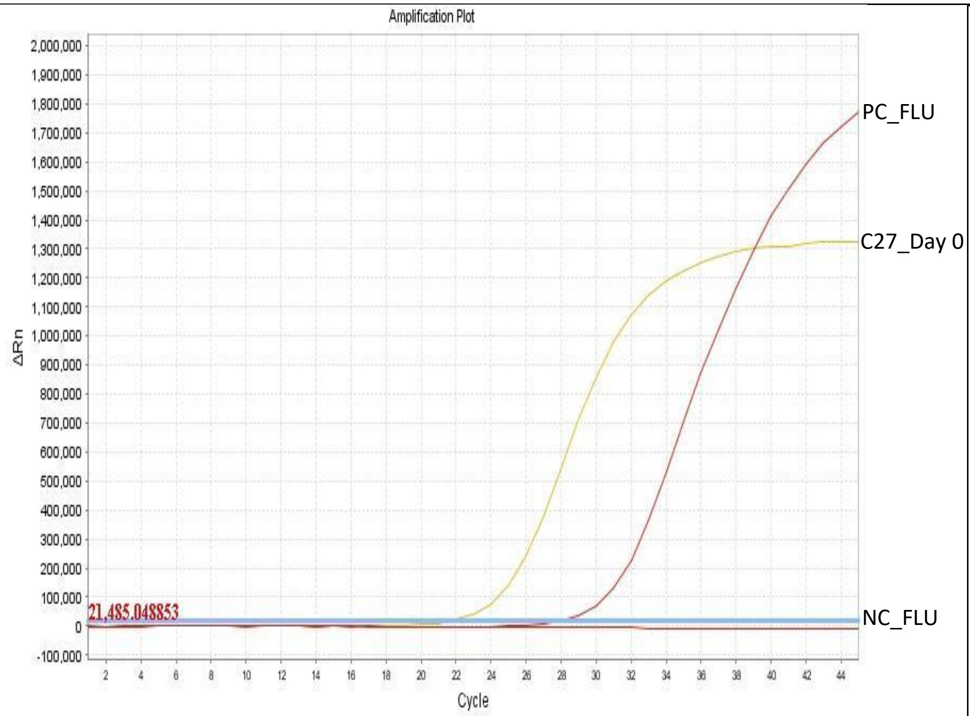Amplification Plot
2,000,000
1,900,000
1,800,000
1,700,000
1,600,000
1,500,000
1,400,000
1,300,000
1,200,000
1,100,000
1,000,000
900,000
800,000
700,000
600,000
500,000
400,000
300,000
200,000
100,000
0
-100,000
ΔRn
2
4
6
8
10
12
14
16
18
20
22
24
26
28
30
32
34
36
38
40
42
44
Cycle
21,485.048853
PC_FLU
C27_Day 0
NC_FLU	

	C27_D2	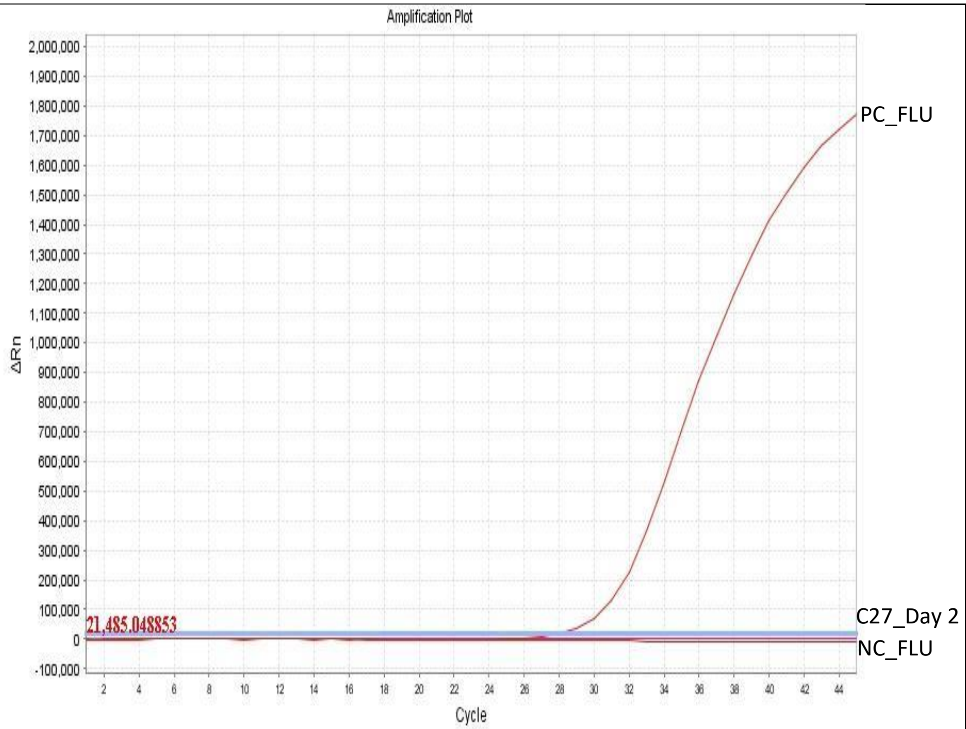 Amplification Plot<br>ΔRn<br>Cycle<br>21,485.048853<br>PC_FLU<br>C27_Day 2<br>NC_FLU	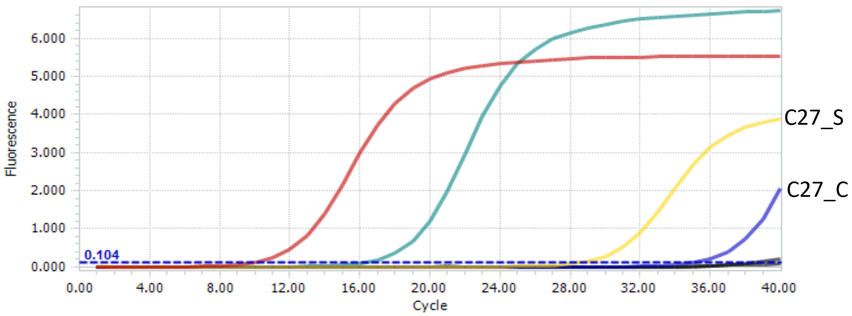 Fluorescence<br>Cycle<br>0.104<br>C27_S<br>C27_C 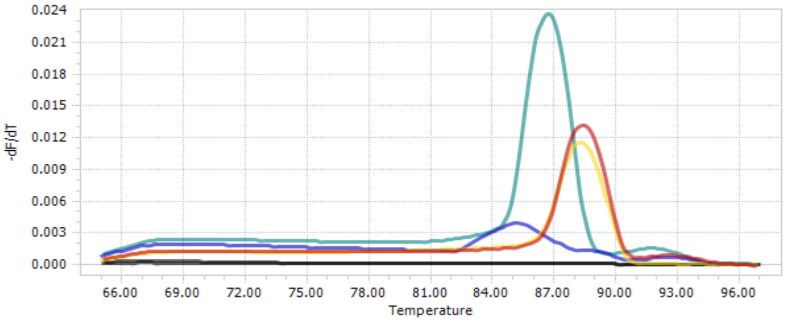 -dF/dT<br>Temperature

25	C28_D0	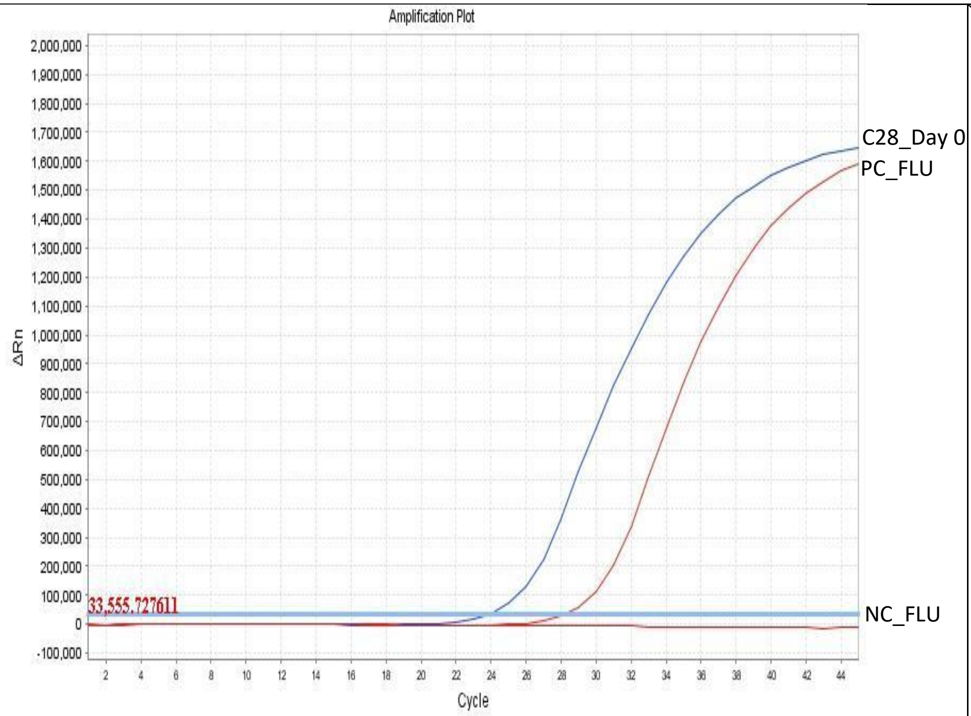Amplification Plot
2,000,000
1,900,000
1,800,000
1,700,000
1,600,000
1,500,000
1,400,000
1,300,000
1,200,000
1,100,000
1,000,000
900,000
800,000
700,000
600,000
500,000
400,000
300,000
200,000
100,000
0
-100,000
ΔRn
33,555.727611
2 4 6 8 10 12 14 16 18 20 22 24 26 28 30 32 34 36 38 40 42 44
Cycle
C28_Day 0
PC_FLU
NC_FLU	

<table>
<tr>
<td></td>
<td>C28_D2</td>
<td>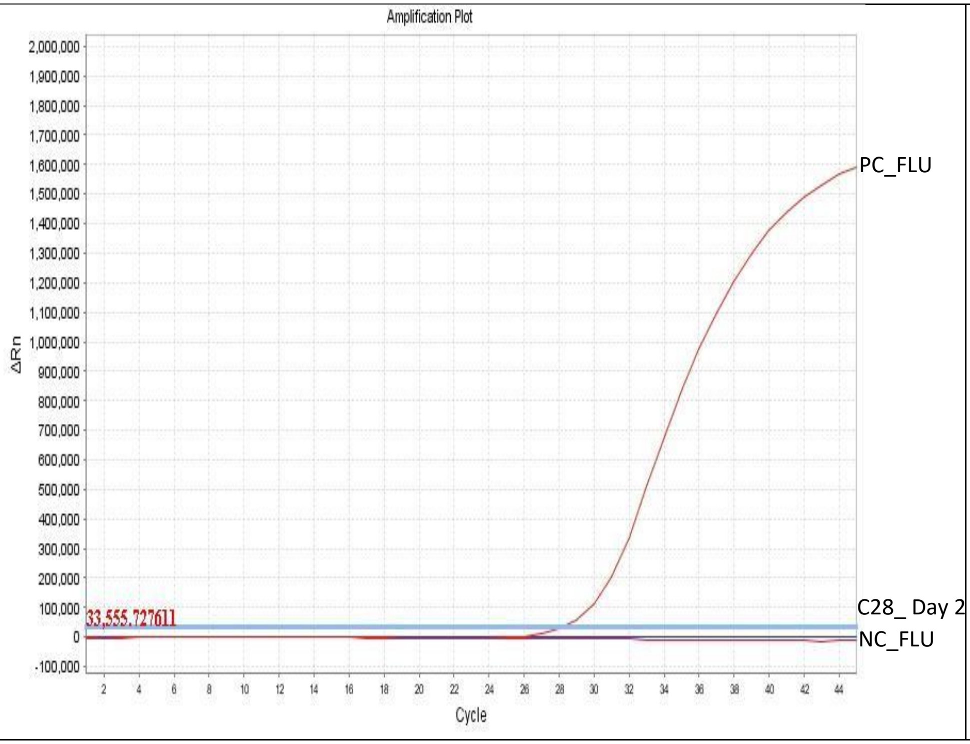
</td>
<td>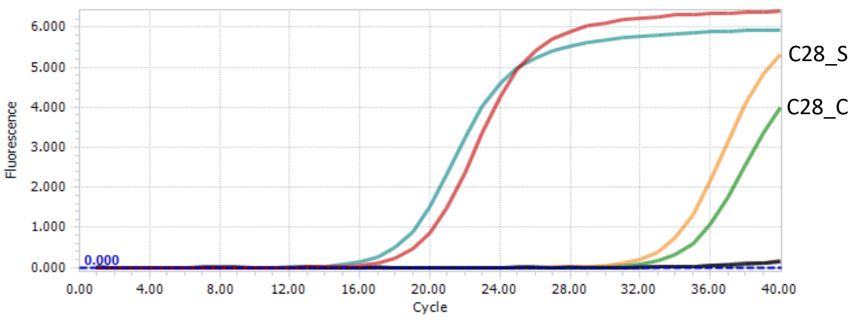
</td>
</tr>
</table>

26	C29_D0	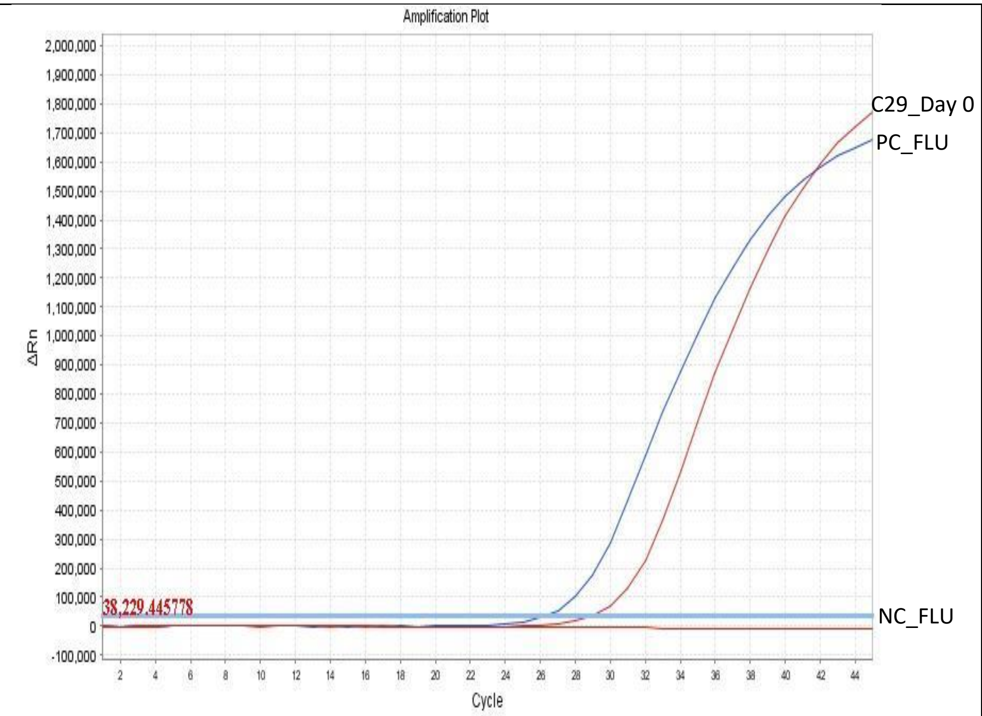Amplification Plot<br>ΔRn<br>Cycle<br>38,229.445778<br>C29_Day 0<br>PC_FLU<br>NC_FLU	

	C29_D2		

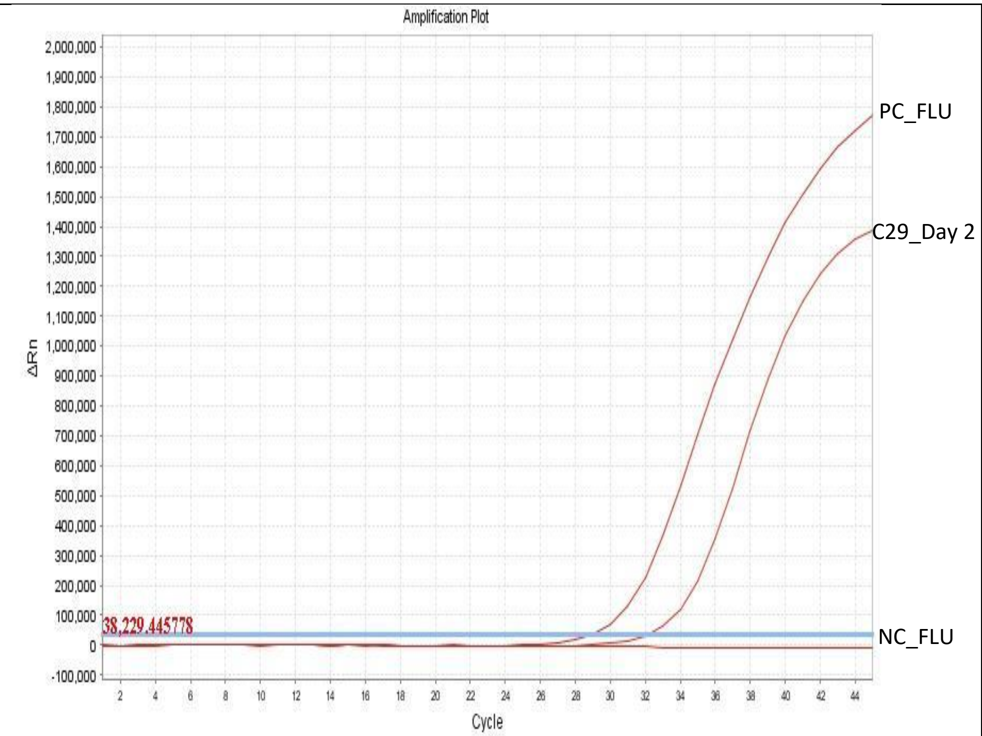

Amplification Plot
ΔRn
Cycle
PC_FLU
C29_Day 2
NC_FLU
38,229.445778
2,000,000
1,900,000
1,800,000
1,700,000
1,600,000
1,500,000
1,400,000
1,300,000
1,200,000
1,100,000
1,000,000
900,000
800,000
700,000
600,000
500,000
400,000
300,000
200,000
100,000
0
-100,000
2 4 6 8 10 12 14 16 18 20 22 24 26 28 30 32 34 36 38 40 42 44

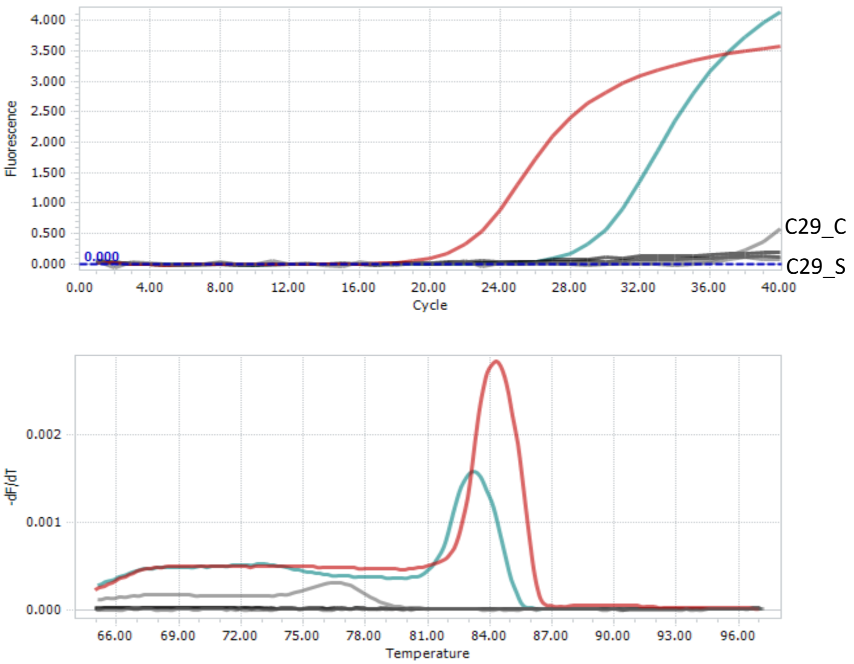

Fluorescence
Cycle
C29_C
C29_S
0.000
4.000
3.500
3.000
2.500
2.000
1.500
1.000
0.500
0.000
0.00 4.00 8.00 12.00 16.00 20.00 24.00 28.00 32.00 36.00 40.00
-dF/dT
Temperature
0.002
0.001
0.000
66.00 69.00 72.00 75.00 78.00 81.00 84.00 87.00 90.00 93.00 96.00

27	C30_D0	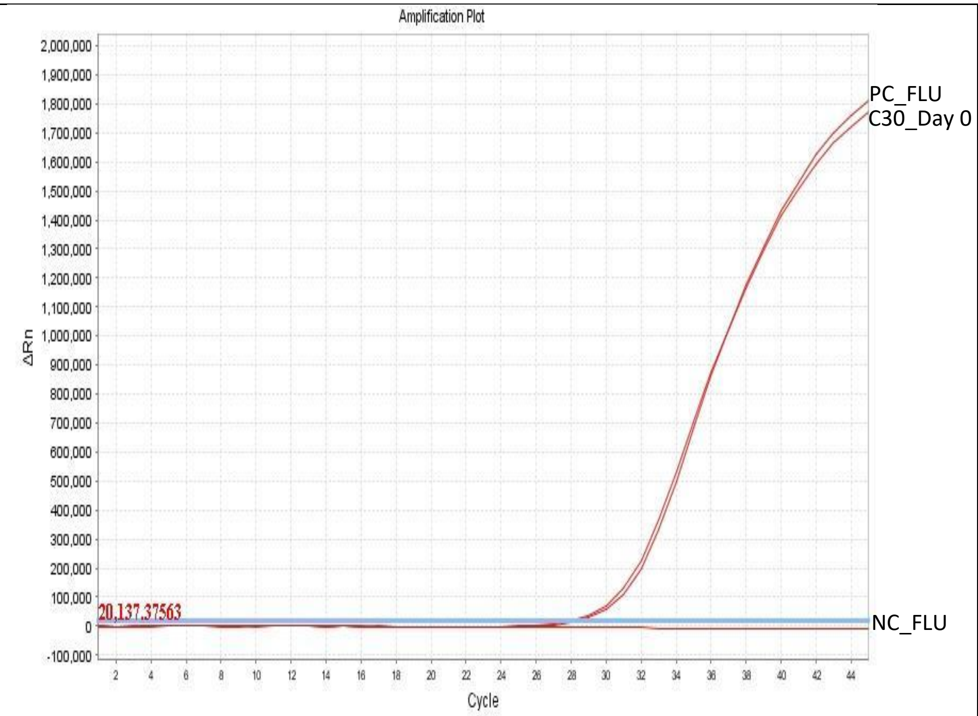Amplification Plot
ΔRn
Cycle
20,137.37563
PC_FLU
C30_Day 0
NC_FLU	

	C30_D2	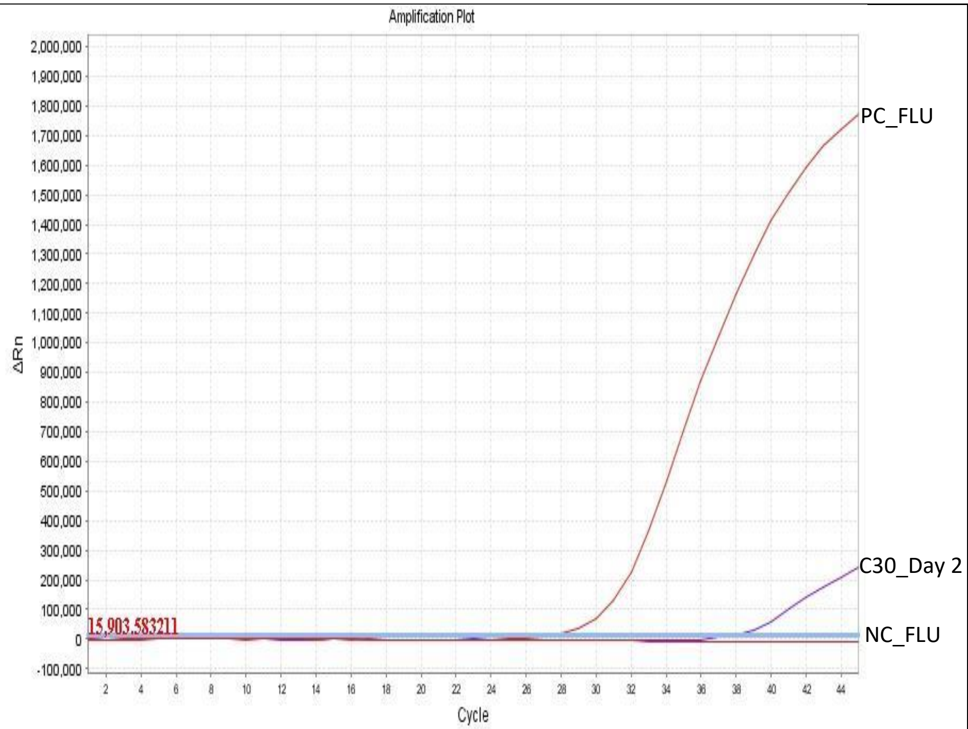Amplification Plot<br>ΔRn<br>Cycle<br>15,903.583211<br>PC_FLU<br>C30_Day 2<br>NC_FLU	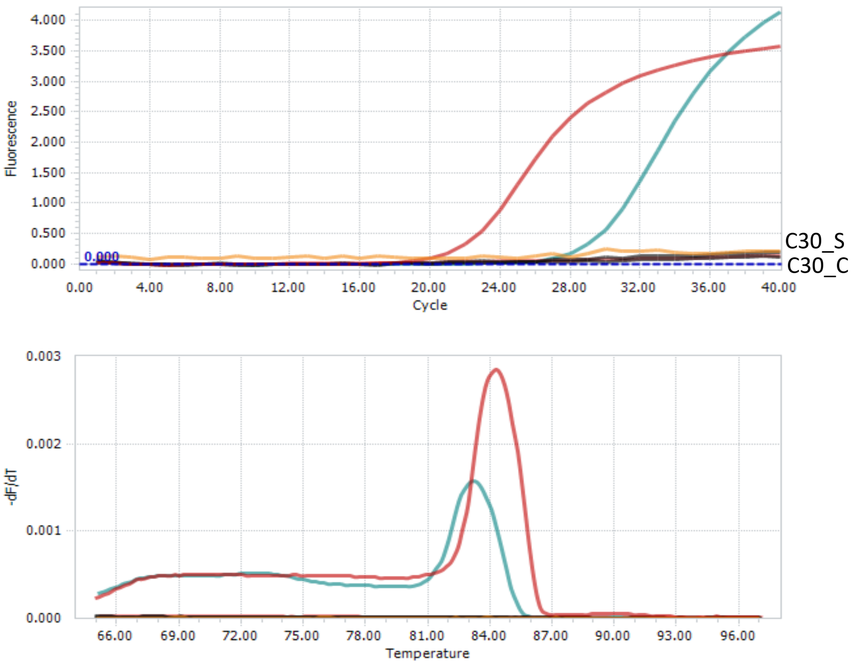Fluorescence<br>Cycle<br>C30_S<br>C30_C<br>-dF/dT<br>Temperature

28	C31_D0	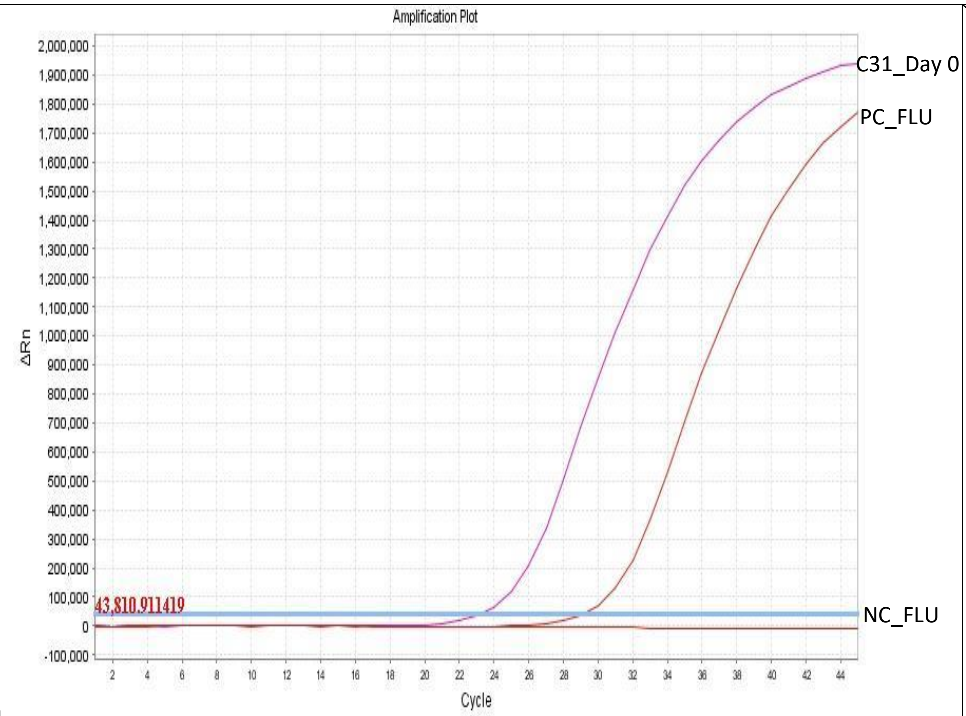Amplification Plot<br>C31_Day 0<br>PC_FLU<br>NC_FLU<br>43,810.911419<br>ΔRn<br>Cycle<br>1	

C31_D2

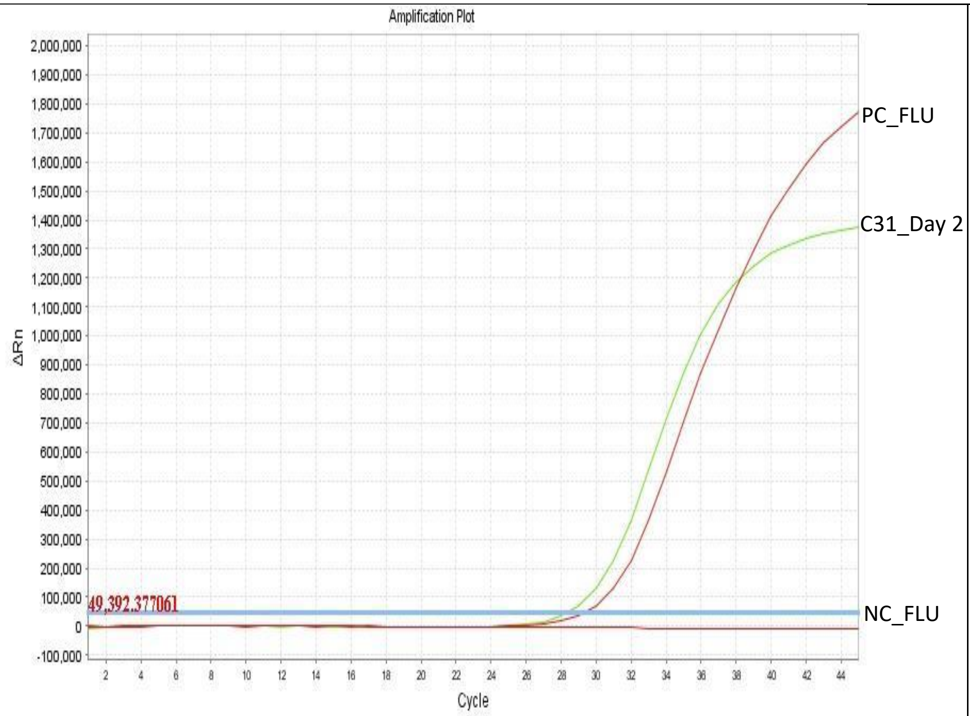

Amplification Plot
ΔRn
Cycle
49,392.377061
PC_FLU
C31_Day 2
NC_FLU

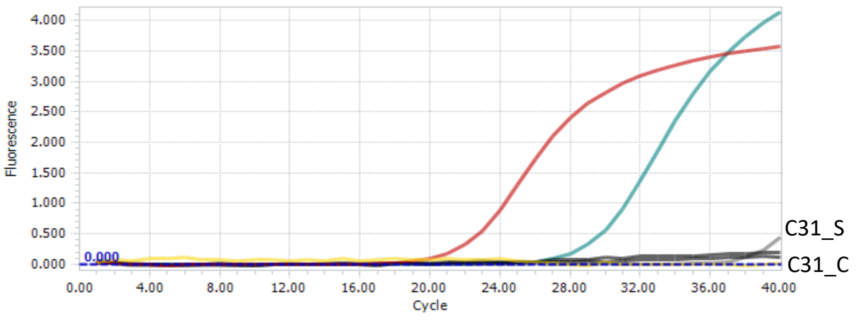

Fluorescence
Cycle
0.000
C31_S
C31_C

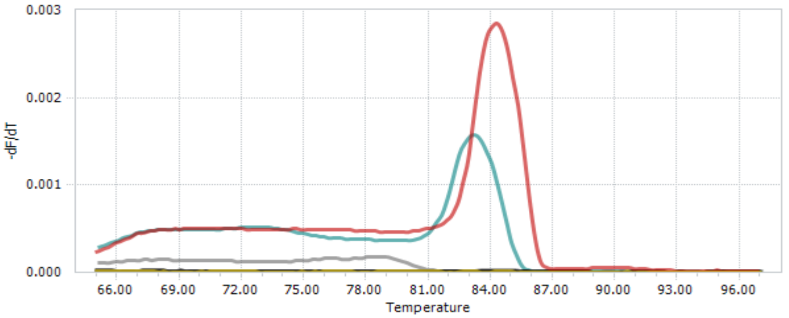

-dF/dT
Temperature

29	C32_D0	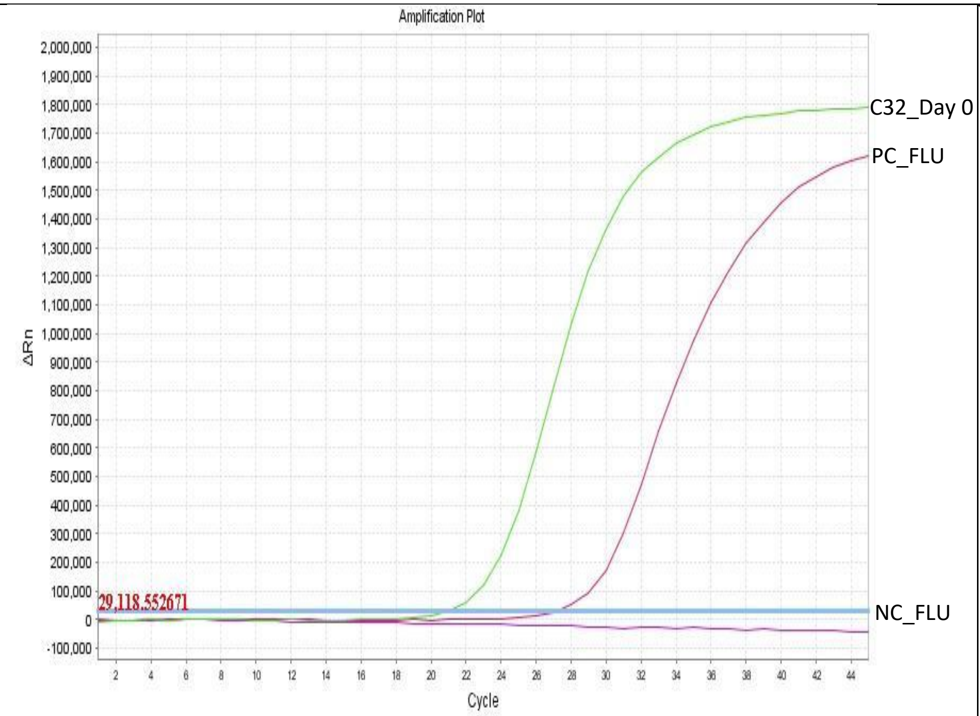Amplification Plot
2,000,000
1,900,000
1,800,000
1,700,000
1,600,000
1,500,000
1,400,000
1,300,000
1,200,000
1,100,000
1,000,000
900,000
800,000
700,000
600,000
500,000
400,000
300,000
200,000
100,000
0
-100,000
ΔRn
29,118.552671
2 4 6 8 10 12 14 16 18 20 22 24 26 28 30 32 34 36 38 40 42 44
Cycle
C32_Day 0
PC_FLU
NC_FLU	

<table>
<tr>
<td></td>
<td>C32_D2</td>
<td>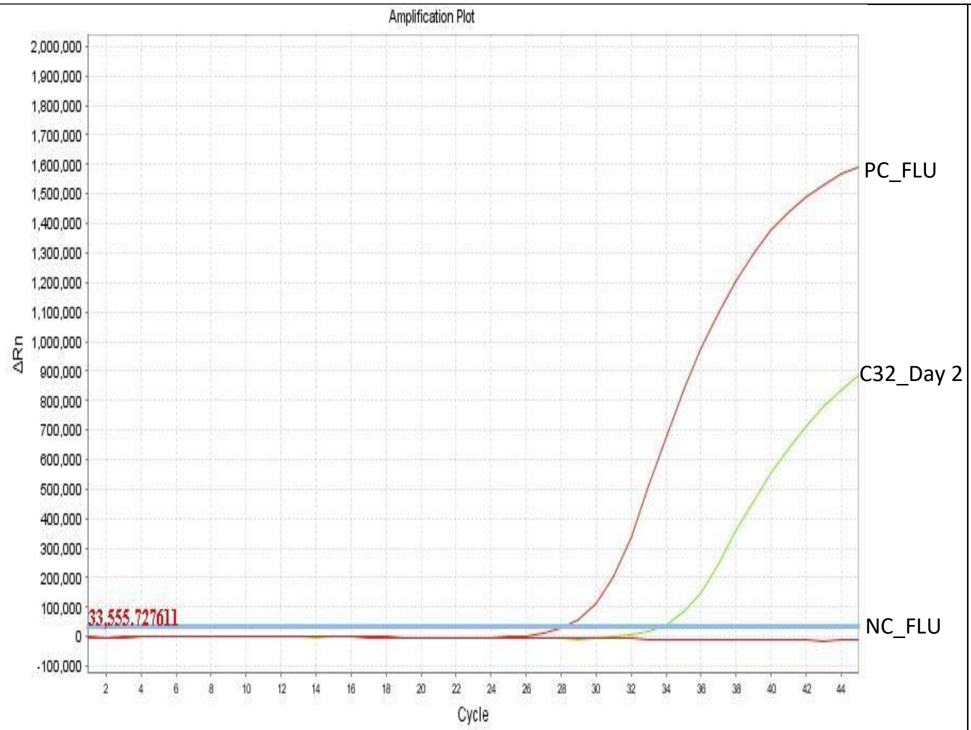
</td>
<td>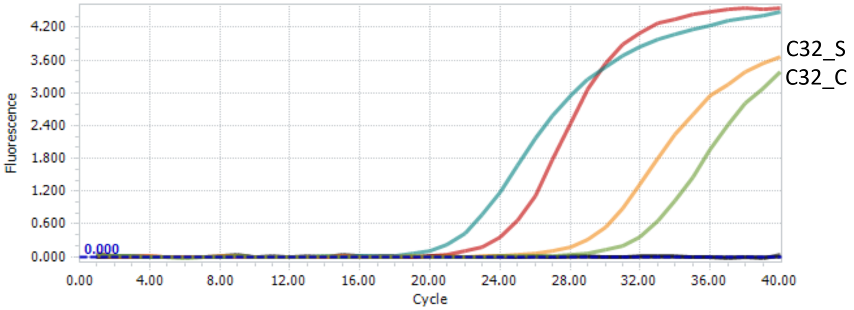

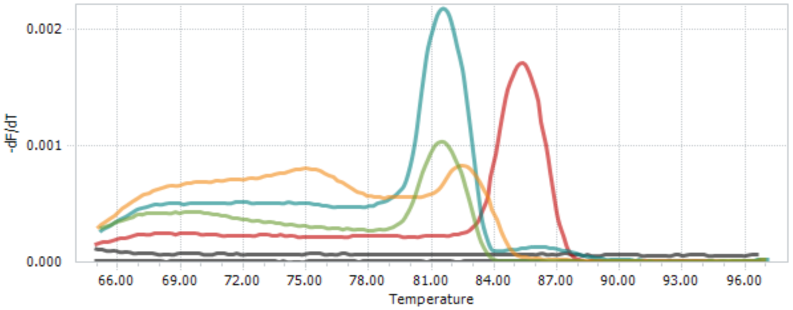
</td>
</tr>
</table>

30	C33_D0	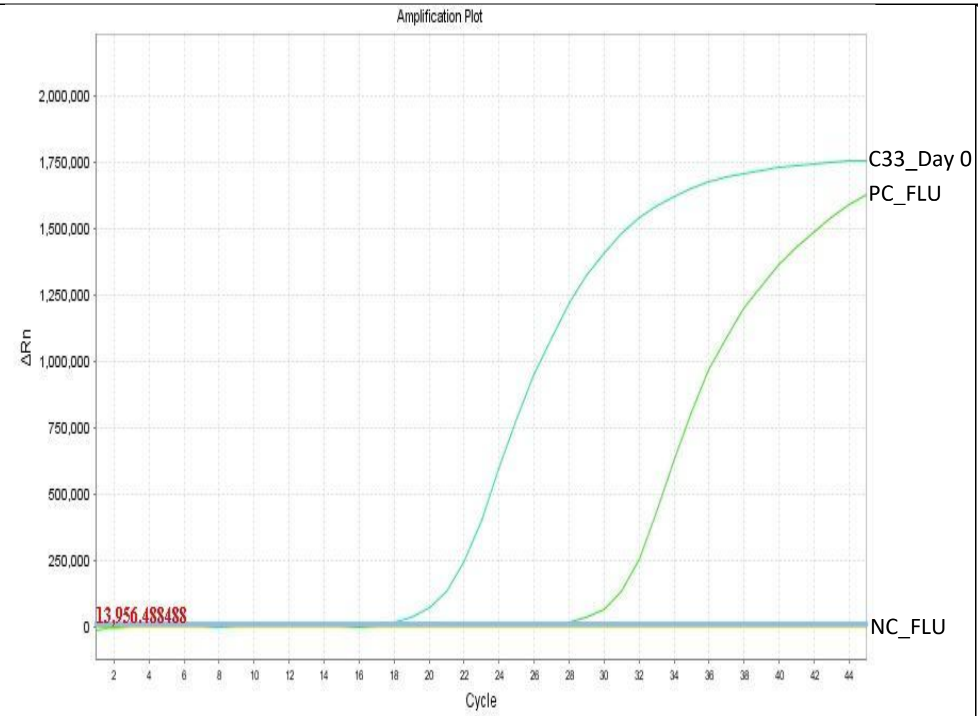Amplification Plot
2,000,000
1,750,000
1,500,000
1,250,000
1,000,000
750,000
500,000
250,000
0
ΔRn
13,956.488488
2
4
6
8
10
12
14
16
18
20
22
24
26
28
30
32
34
36
38
40
42
44
Cycle
C33_Day 0
PC_FLU
NC_FLU	

C33_D2

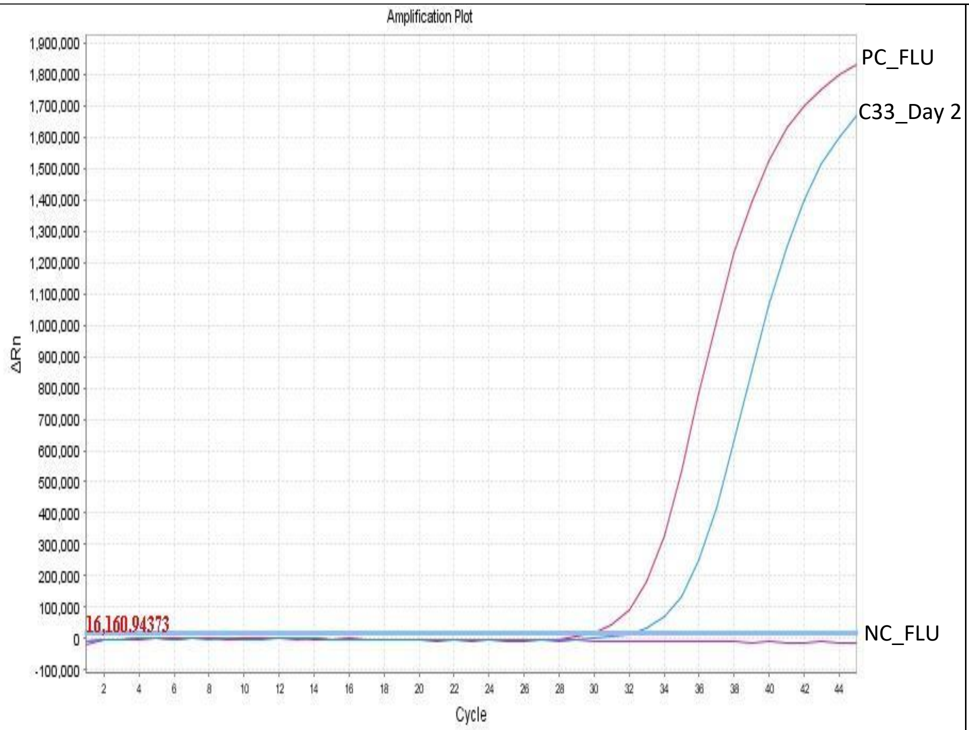

Amplification Plot
PC_FLU
C33_Day 2
NC_FLU
16,160.94373
ΔRn
Cycle
1,900,000
1,800,000
1,700,000
1,600,000
1,500,000
1,400,000
1,300,000
1,200,000
1,100,000
1,000,000
900,000
800,000
700,000
600,000
500,000
400,000
300,000
200,000
100,000
0
-100,000
2 4 6 8 10 12 14 16 18 20 22 24 26 28 30 32 34 36 38 40 42 44

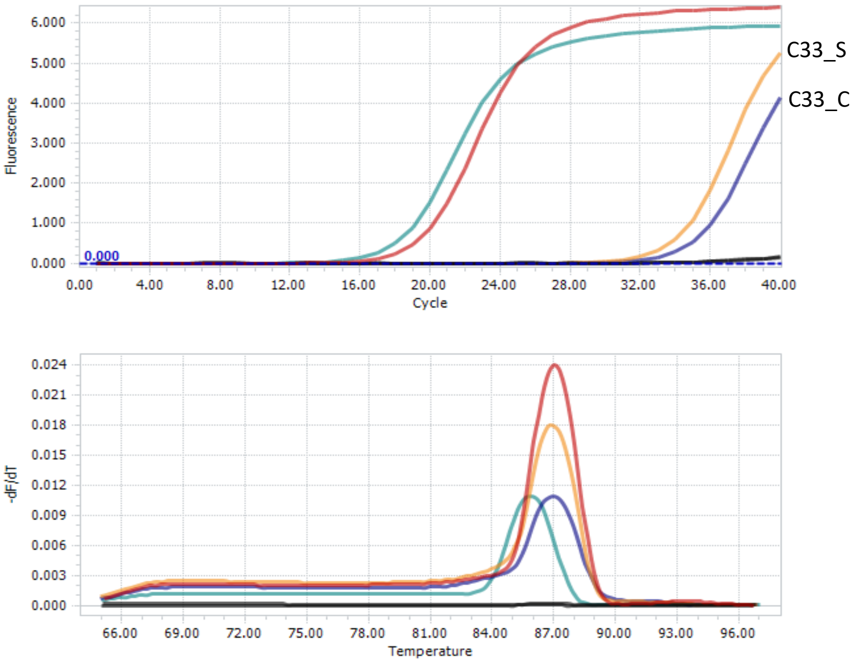

C33_S
C33_C
Fluorescence
6.000
5.000
4.000
3.000
2.000
1.000
0.000
0.000
0.00 4.00 8.00 12.00 16.00 20.00 24.00 28.00 32.00 36.00 40.00
Cycle
-dF/dT
0.024
0.021
0.018
0.015
0.012
0.009
0.006
0.003
0.000
66.00 69.00 72.00 75.00 78.00 81.00 84.00 87.00 90.00 93.00 96.00
Temperature

31	C34_D0	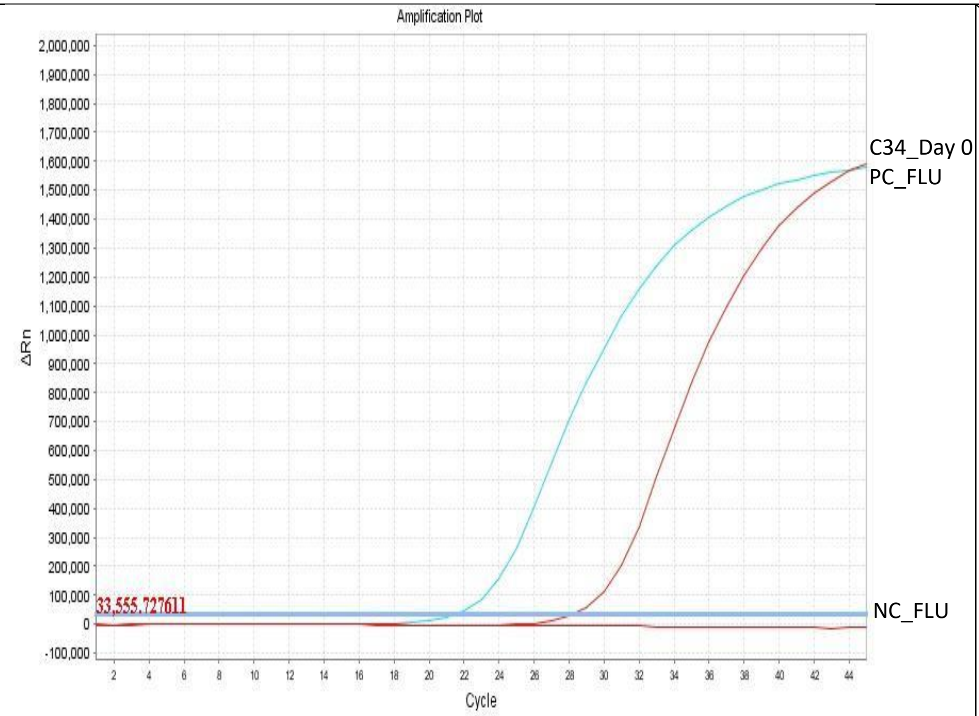Amplification Plot
ΔRn
Cycle
33,555.727611
C34_Day 0
PC_FLU
NC_FLU	

	C34_D2	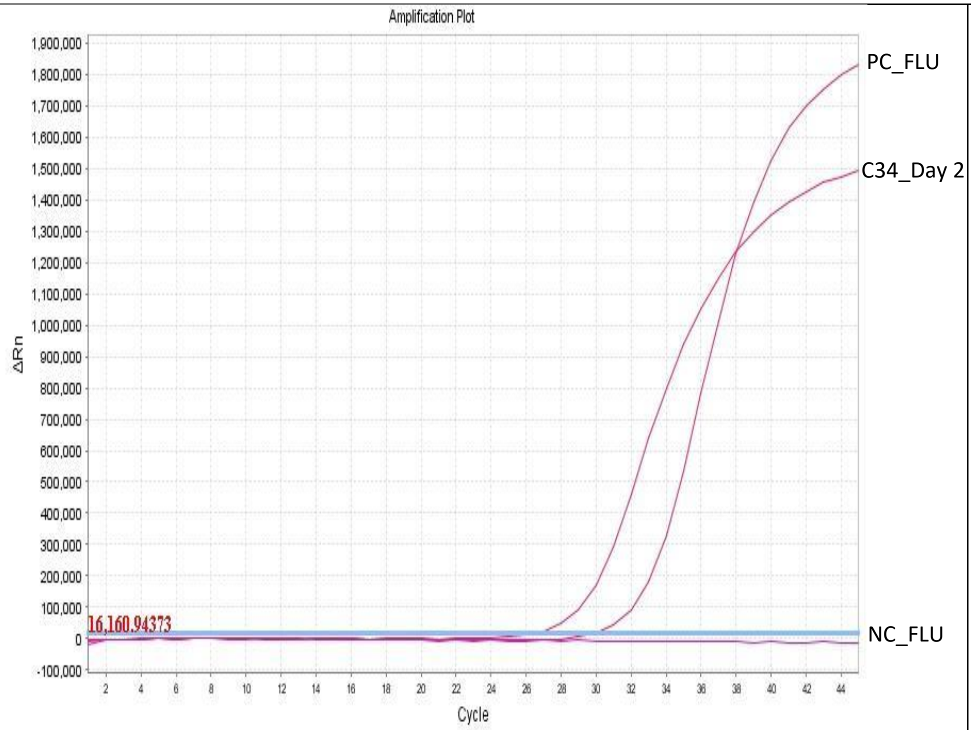 Amplification Plot<br>PC_FLU<br>C34_Day 2<br>NC_FLU<br>16,160.94373<br>ΔRn<br>Cycle	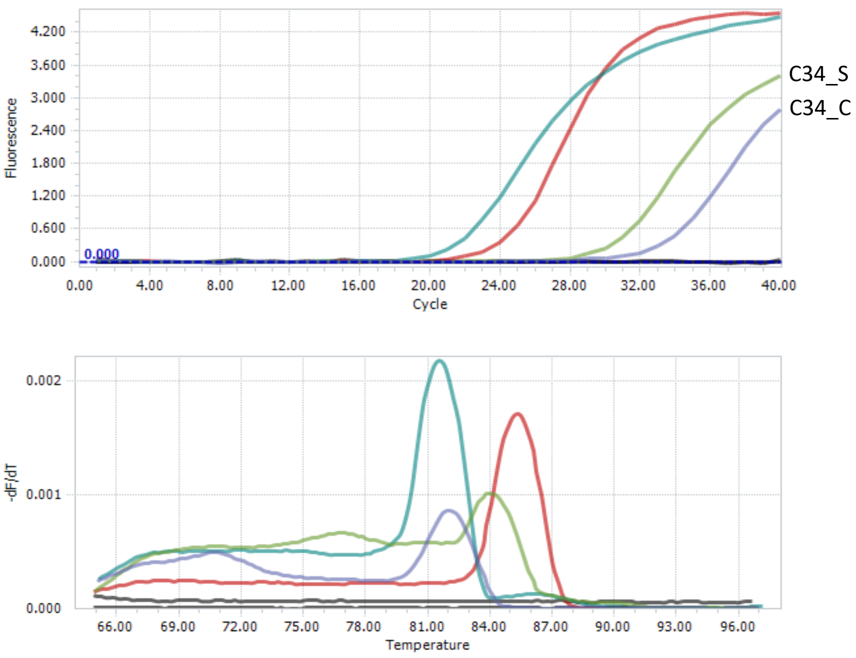 Fluorescence<br>Cycle<br>C34_S<br>C34_C<br>-dF/dT<br>Temperature

32	C35_D0	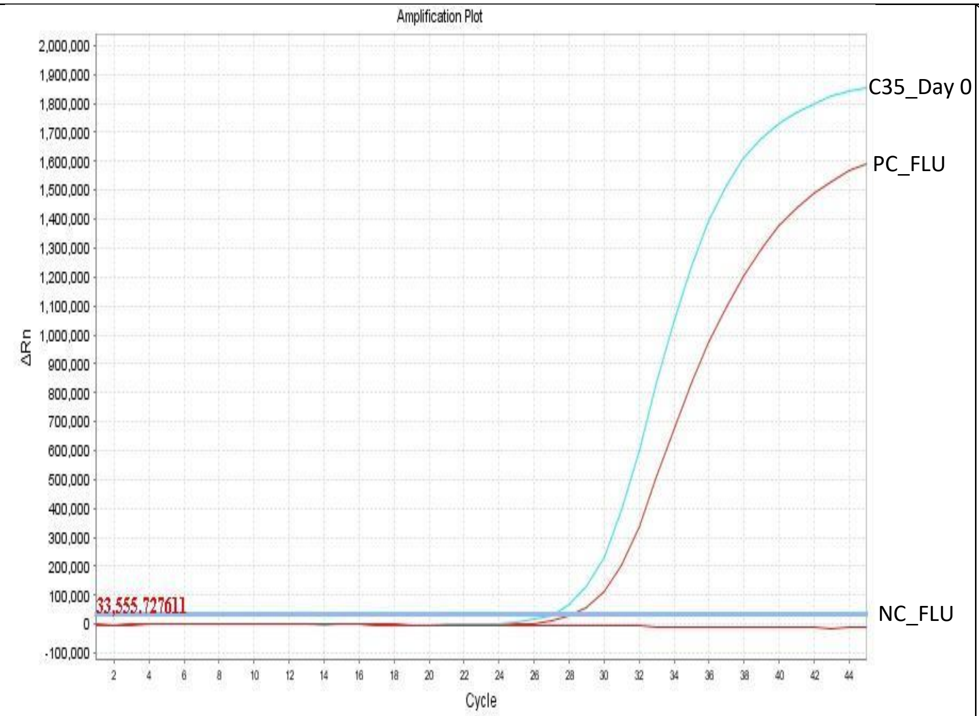Amplification Plot<br>ΔRn<br>Cycle<br>33,555.727611<br>C35_Day 0<br>PC_FLU<br>NC_FLU	

	C35_D2	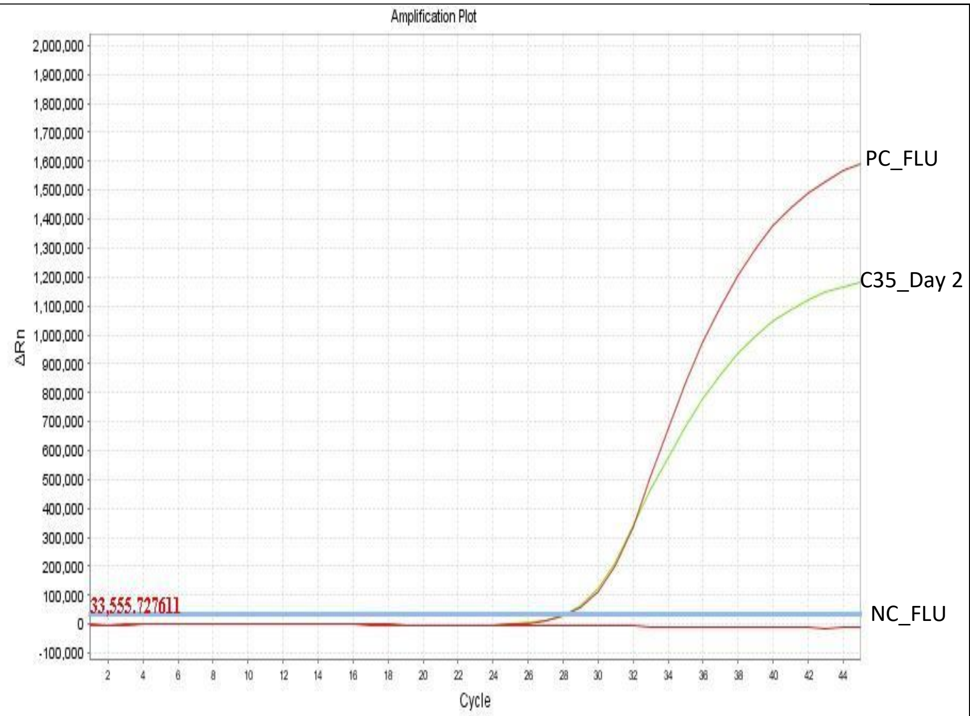 Amplification Plot<br>ΔRn<br>Cycle<br>33,555.727611<br>PC_FLU<br>C35_Day 2<br>NC_FLU	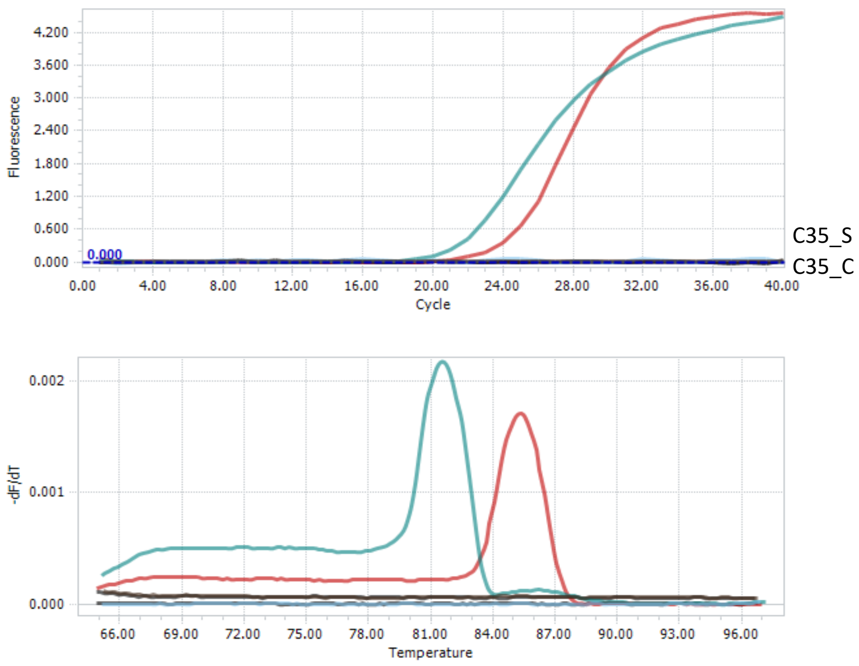 Fluorescence<br>Cycle<br>0.000<br>C35_S<br>C35_C<br>-dF/dT<br>Temperature

33	C36_D0	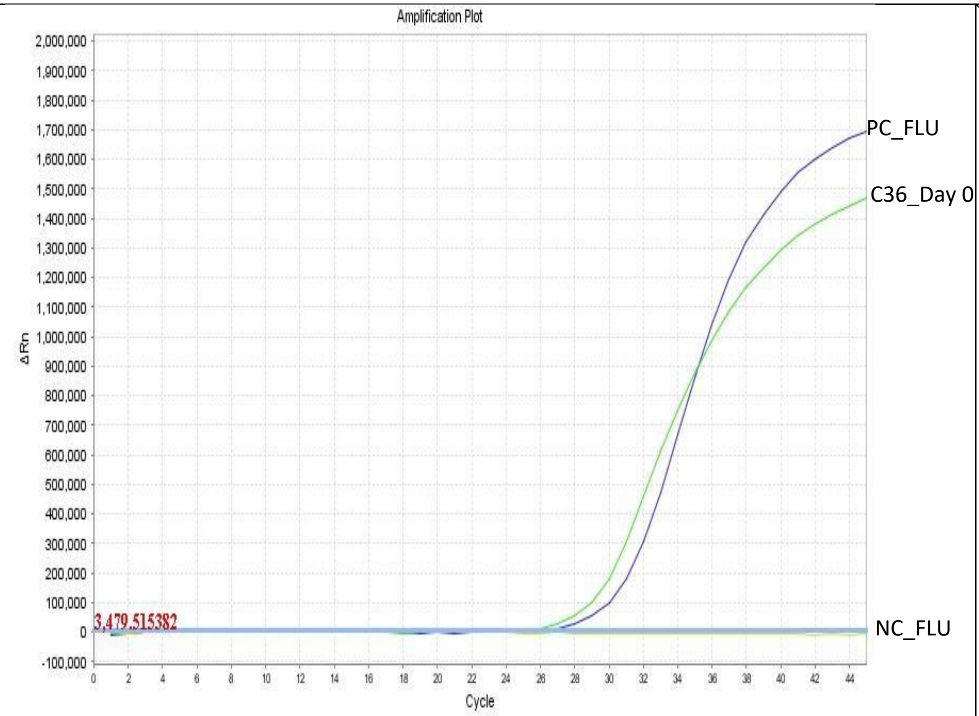Amplification Plot
ΔRn
Cycle
3,479.515382
PC_FLU
C36_Day 0
NC_FLU	

	C36_D2	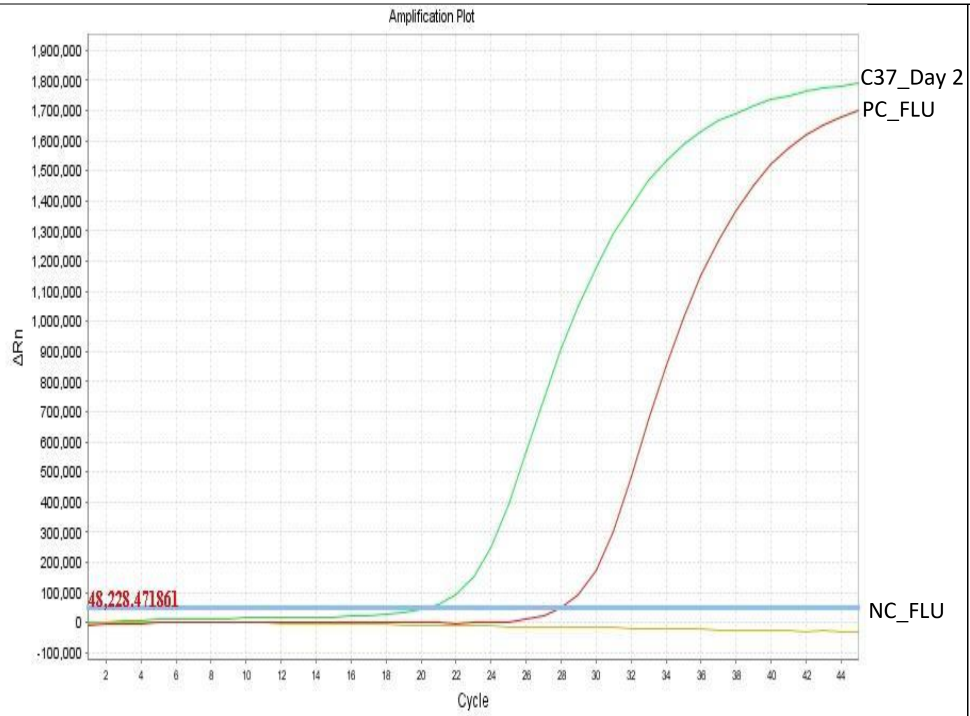	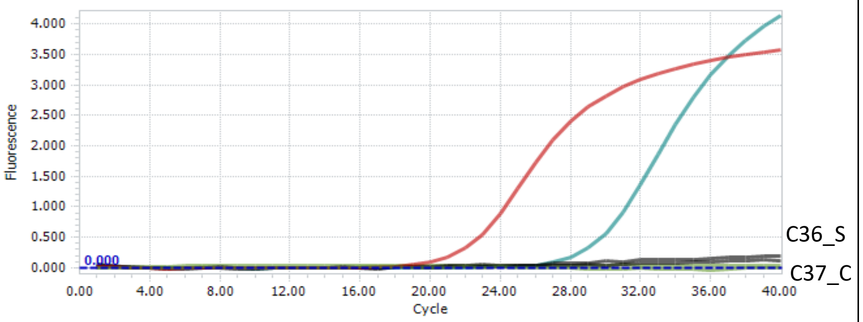 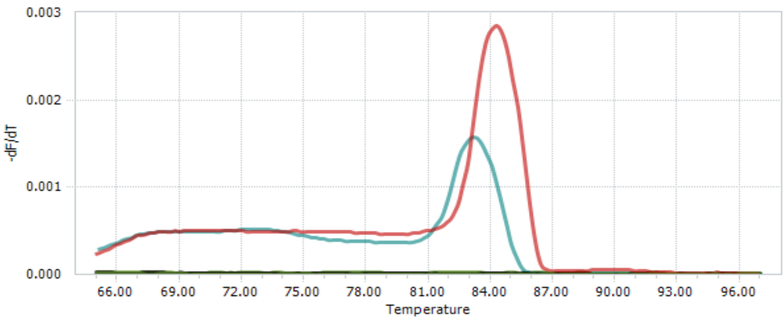

Amplification Plot
ΔRn
Cycle
48,228.471861
C37_Day 2
PC_FLU
NC_FLU

Fluorescence
Cycle
0.000
C36_S
C37_C

-dF/dT
Temperature

34	C37 _D0	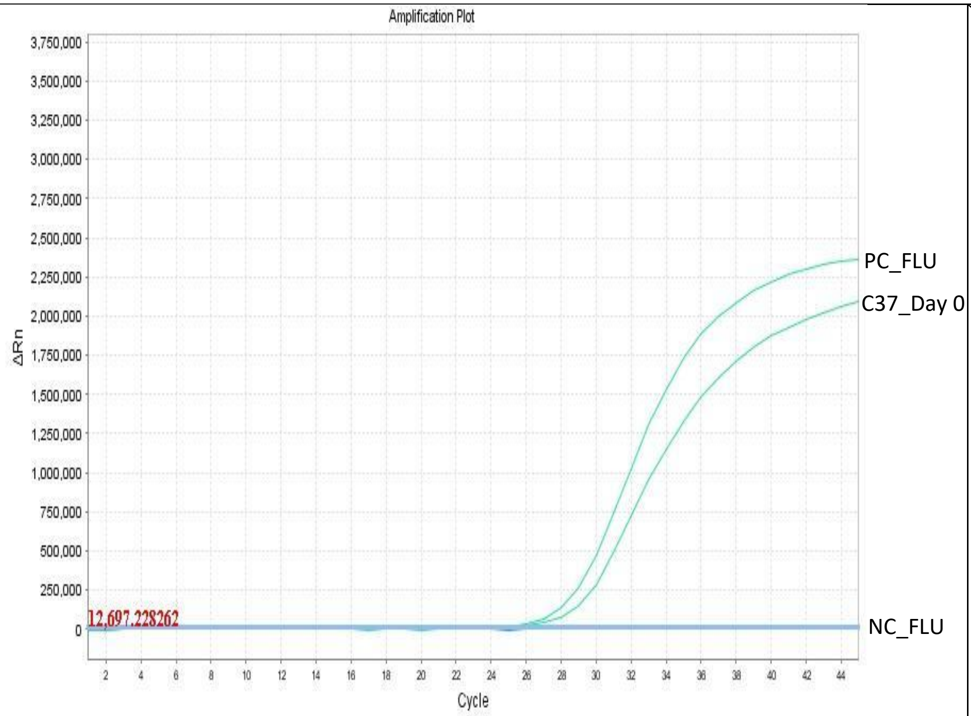Amplification Plot
3,750,000
3,500,000
3,250,000
3,000,000
2,750,000
2,500,000
2,250,000
2,000,000
1,750,000
1,500,000
1,250,000
1,000,000
750,000
500,000
250,000
0
ΔRn
12,697.228262
2
4
6
8
10
12
14
16
18
20
22
24
26
28
30
32
34
36
38
40
42
44
Cycle
PC_FLU
C37_Day 0
NC_FLU	

<table>
<tr>
<td></td>
<td>C37_D2</td>
<td>
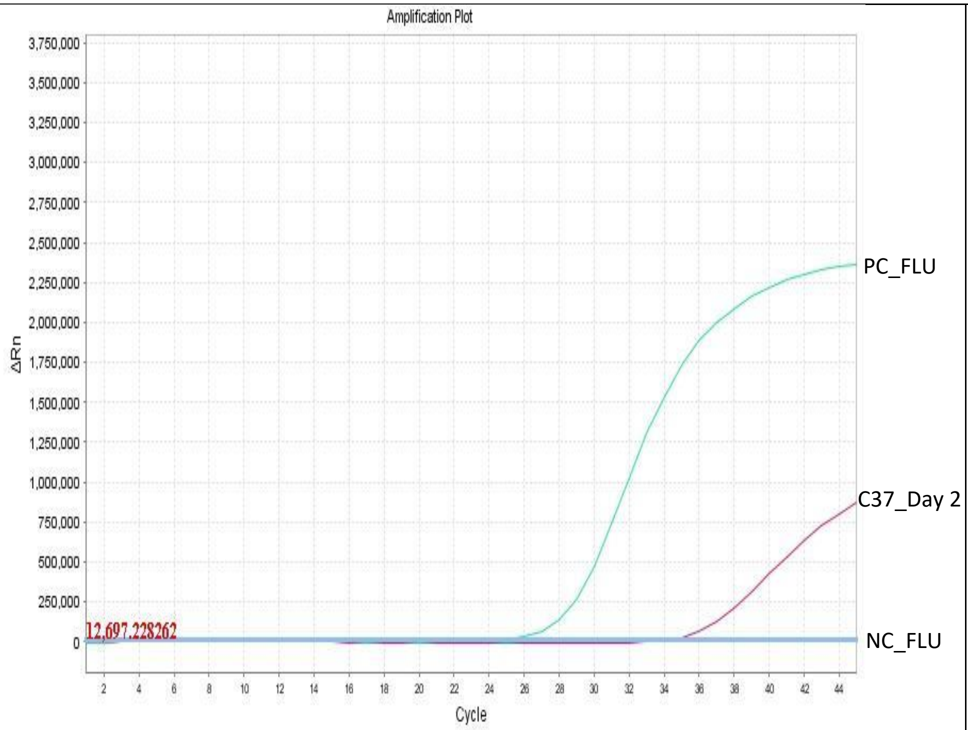

</td>
<td>
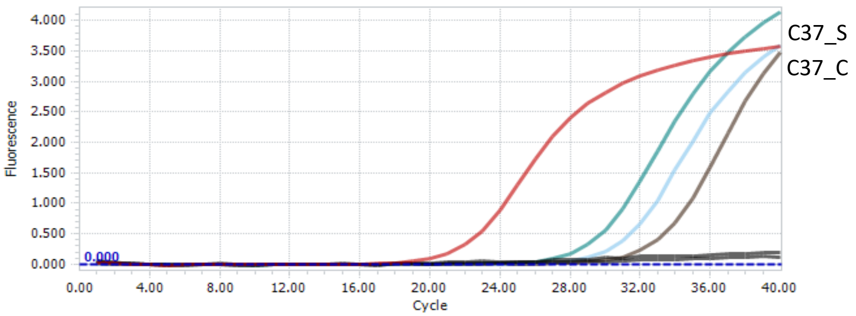

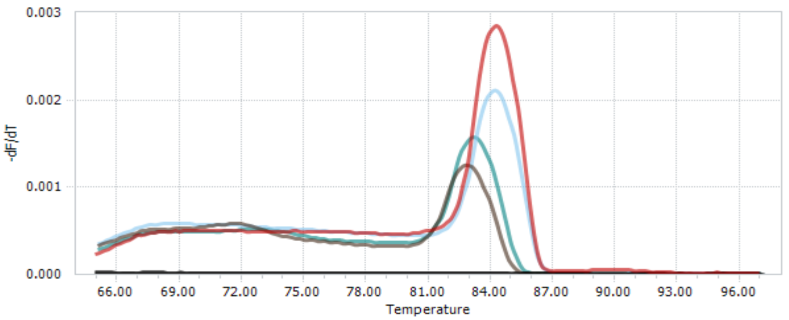

</td>
</tr>
</table>

35	C38_D0	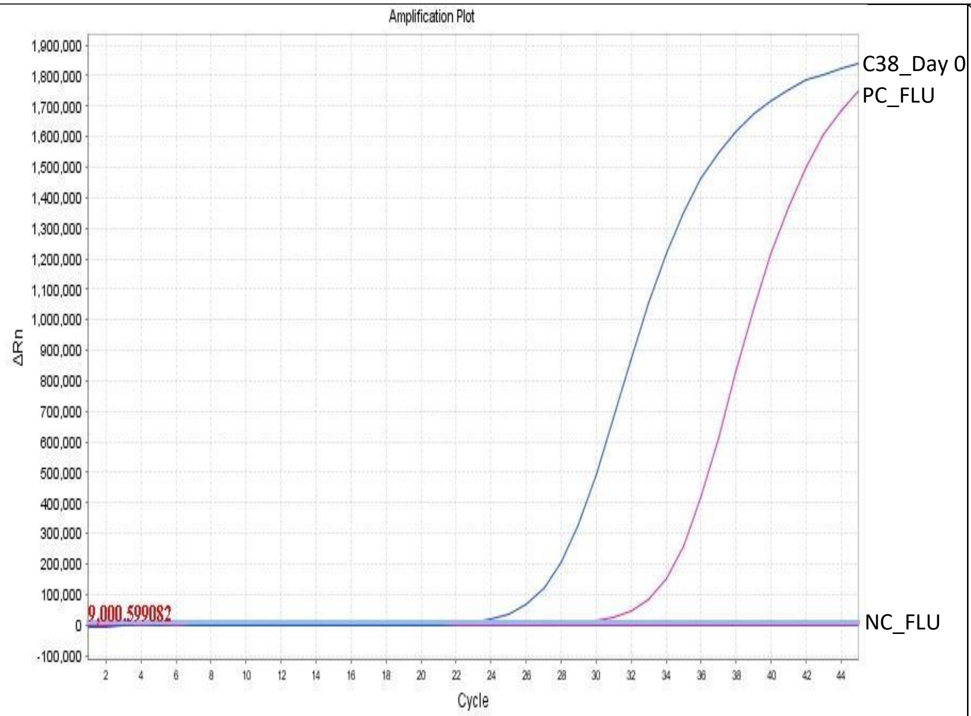Amplification Plot
ΔRn
Cycle
9,000.599082
C38_Day 0
PC_FLU
NC_FLU	

	C38_D2	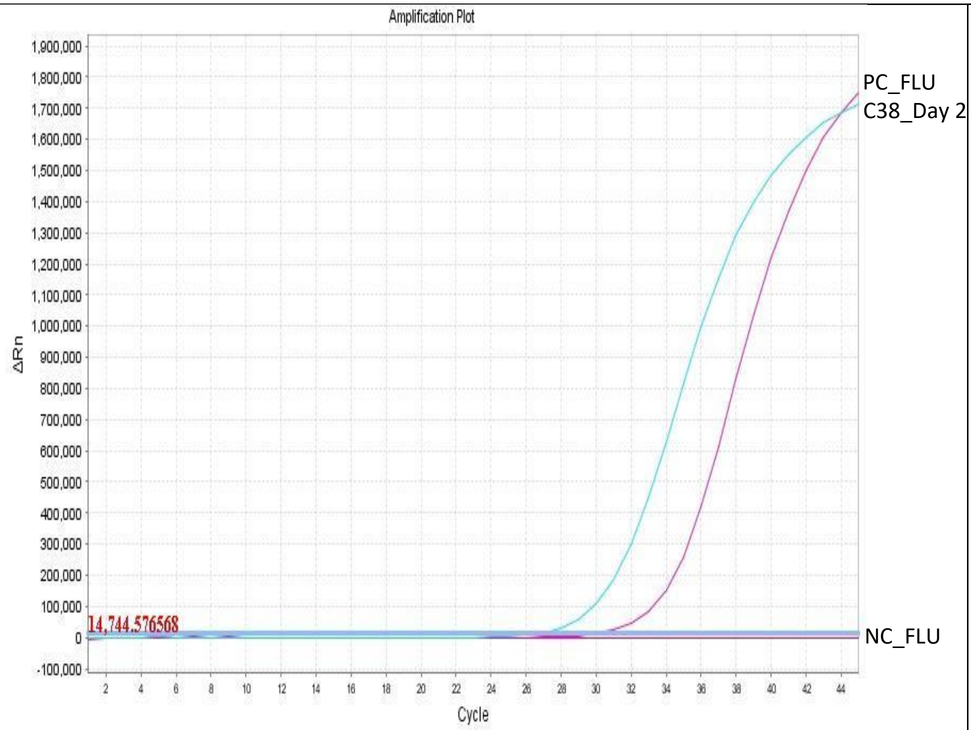 Amplification Plot<br>ΔRn<br>Cycle<br>14,744.576568<br>PC_FLU<br>C38_Day 2<br>NC_FLU	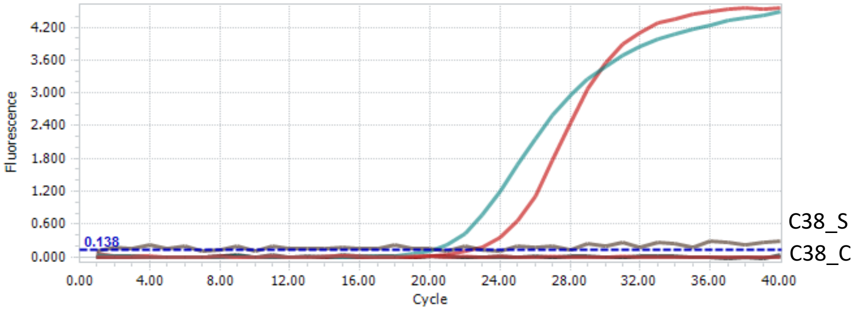 Fluorescence<br>Cycle<br>0.138<br>C38_S<br>C38_C 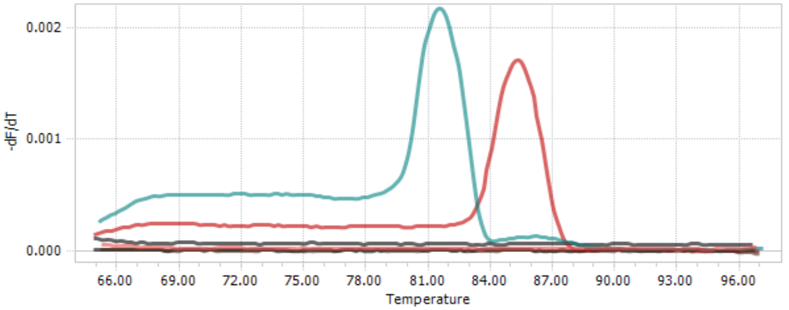 -dF/dT<br>Temperature

36	C39 _D0	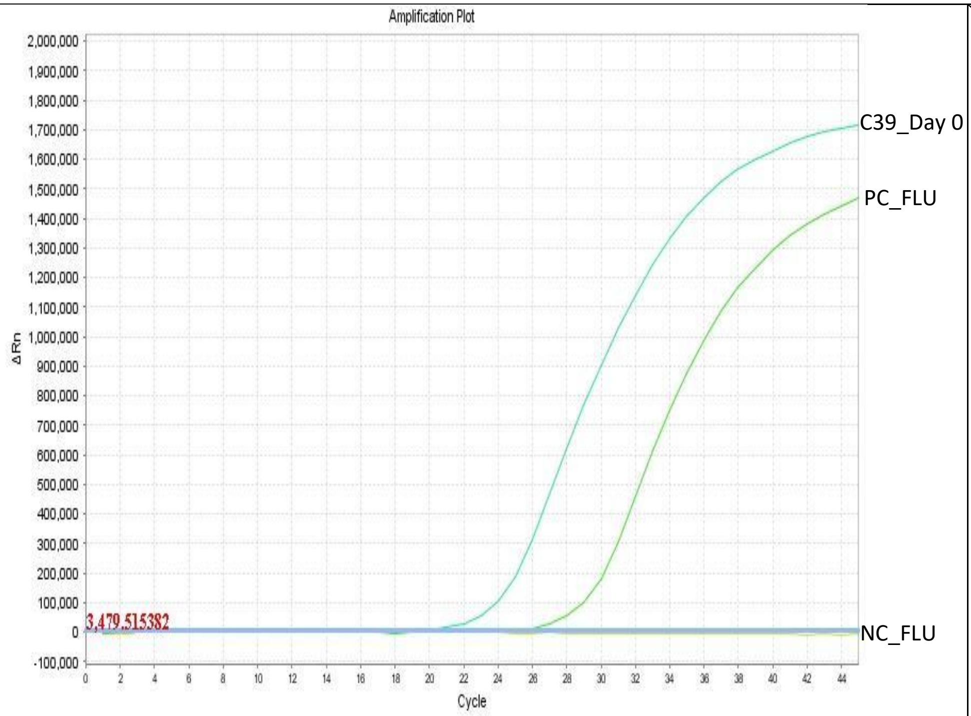Amplification Plot
ΔRn
Cycle
3,479.515382
C39_Day 0
PC_FLU
NC_FLU	

	C39_D2	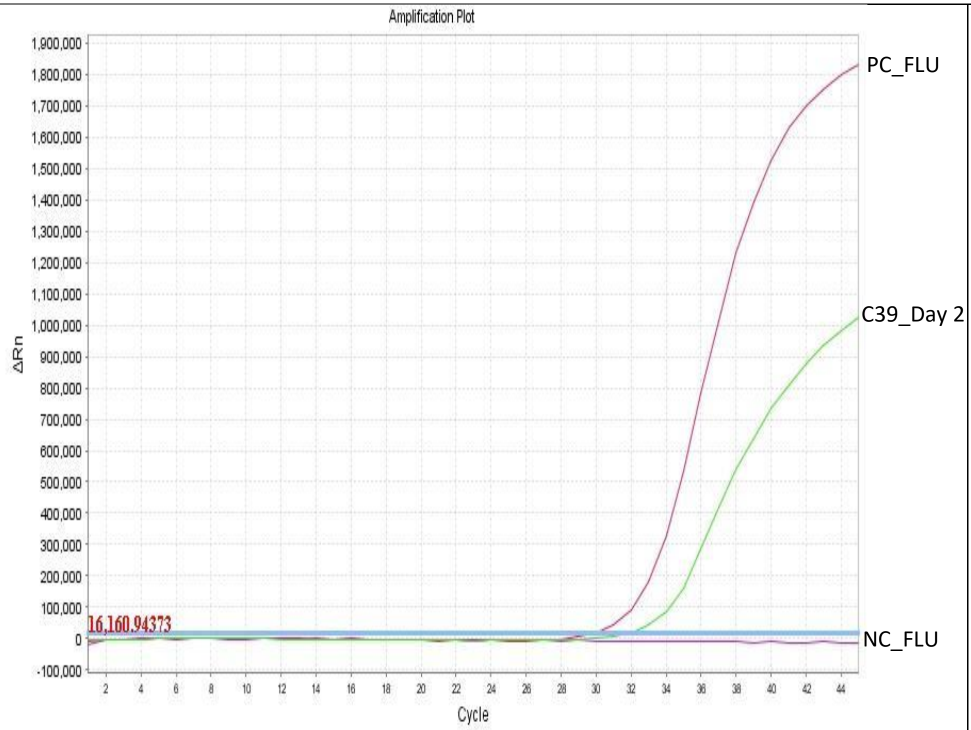	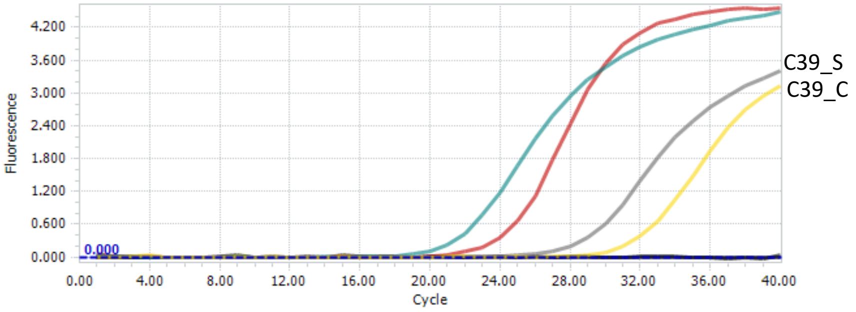 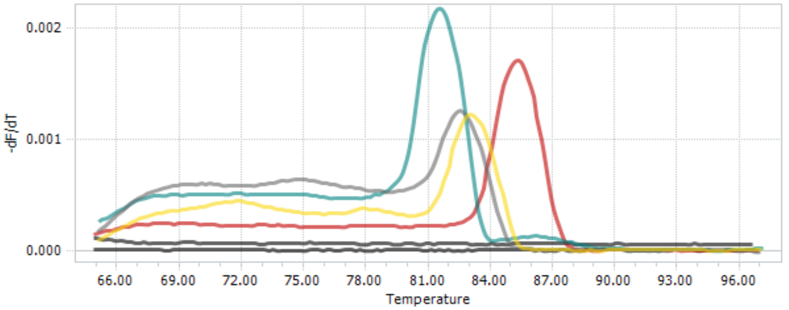

Amplification Plot
ΔRn
Cycle
16,160.94373
PC_FLU
C39_Day 2
NC_FLU

Fluorescence
Cycle
0.000
C39_S
C39_C

-dF/dT
Temperature

37	C41 _D0	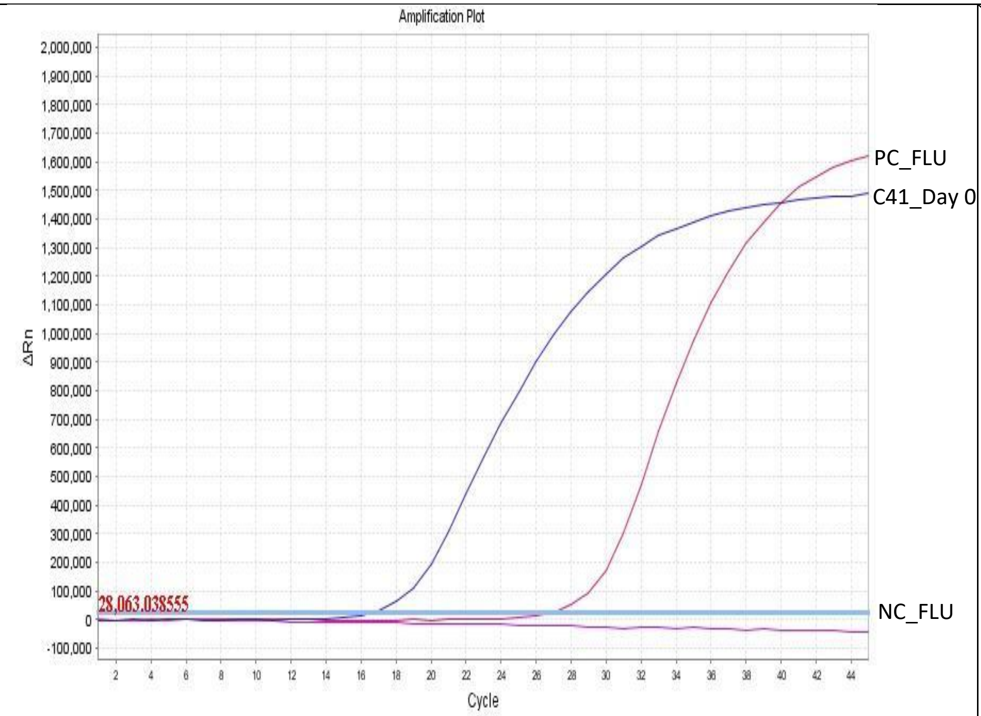Amplification Plot<br>ΔRn<br>Cycle<br>28,063.038555<br>PC_FLU<br>C41_Day 0<br>NC_FLU	

	C41_D2	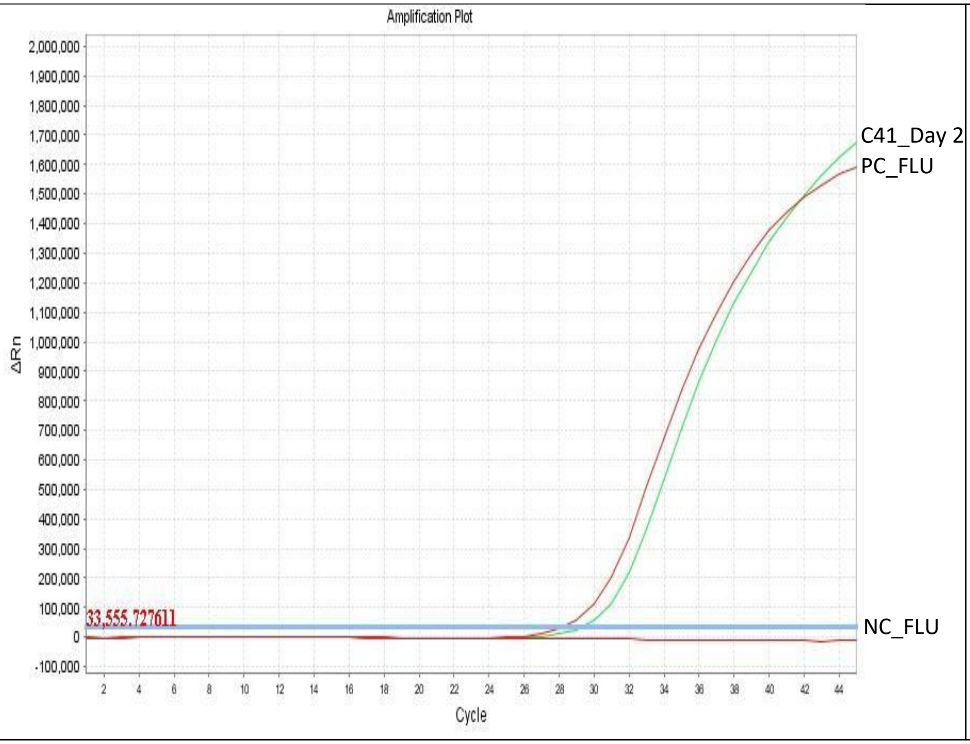 Amplification Plot<br>ΔRn<br>Cycle<br>33,555.727611<br>C41_Day 2<br>PC_FLU<br>NC_FLU	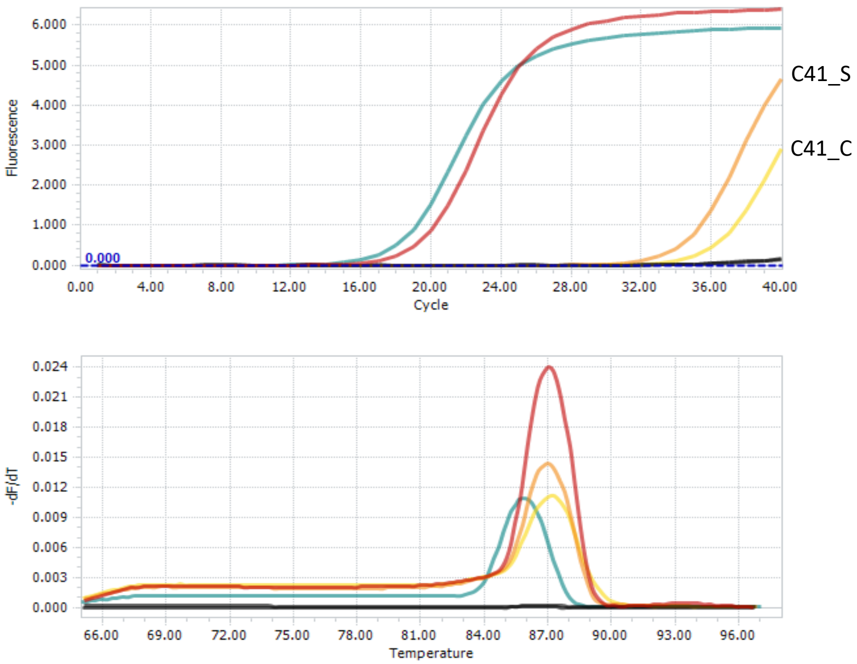 Fluorescence<br>Cycle<br>C41_S<br>C41_C<br>-dF/dT<br>Temperature

38	C42_D0	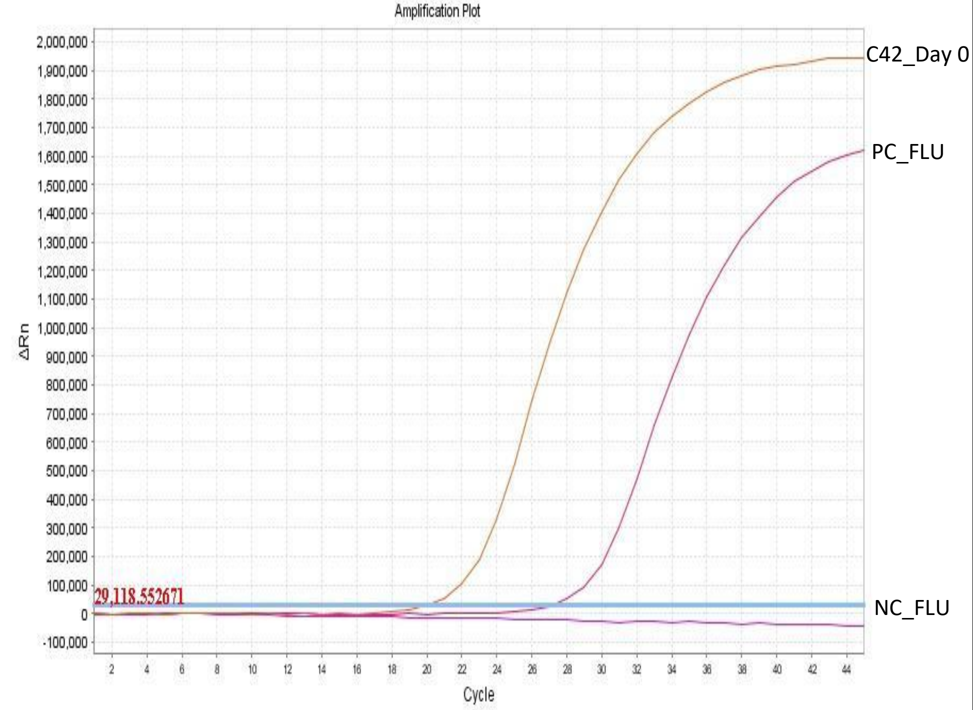Amplification Plot<br>C42_Day 0<br>PC_FLU<br>NC_FLU<br>29,118.552671<br>ΔRn<br>Cycle	

<table>
<tr>
<td></td>
<td>C42_D2</td>
<td>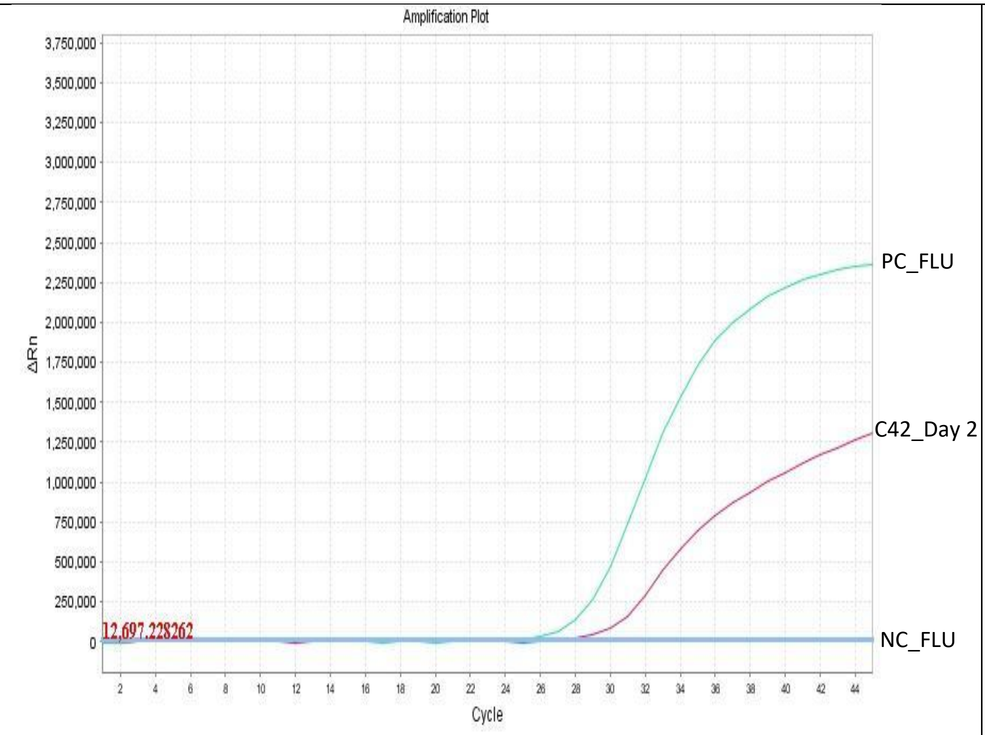
</td>
<td>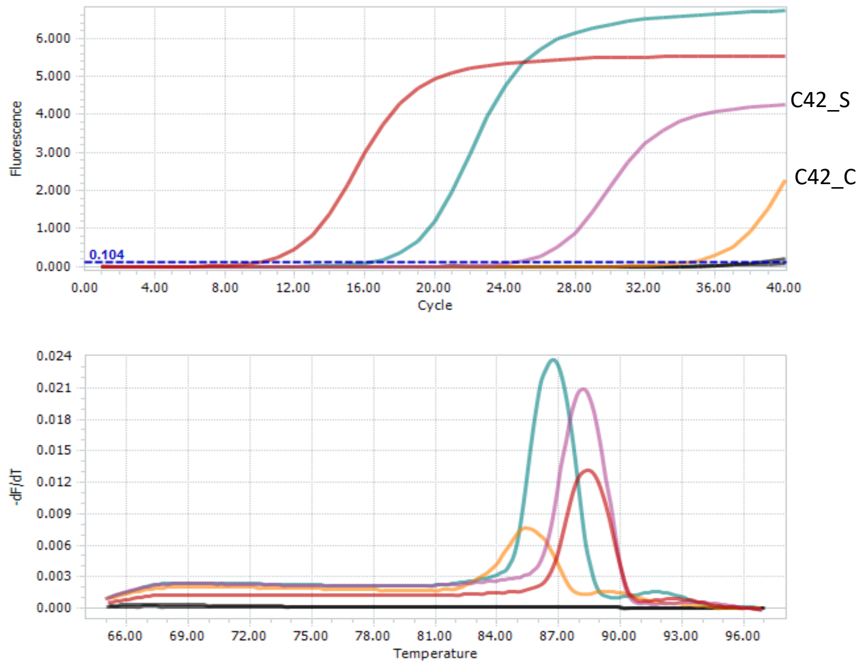
</td>
</tr>
</table>

39	C43_D0	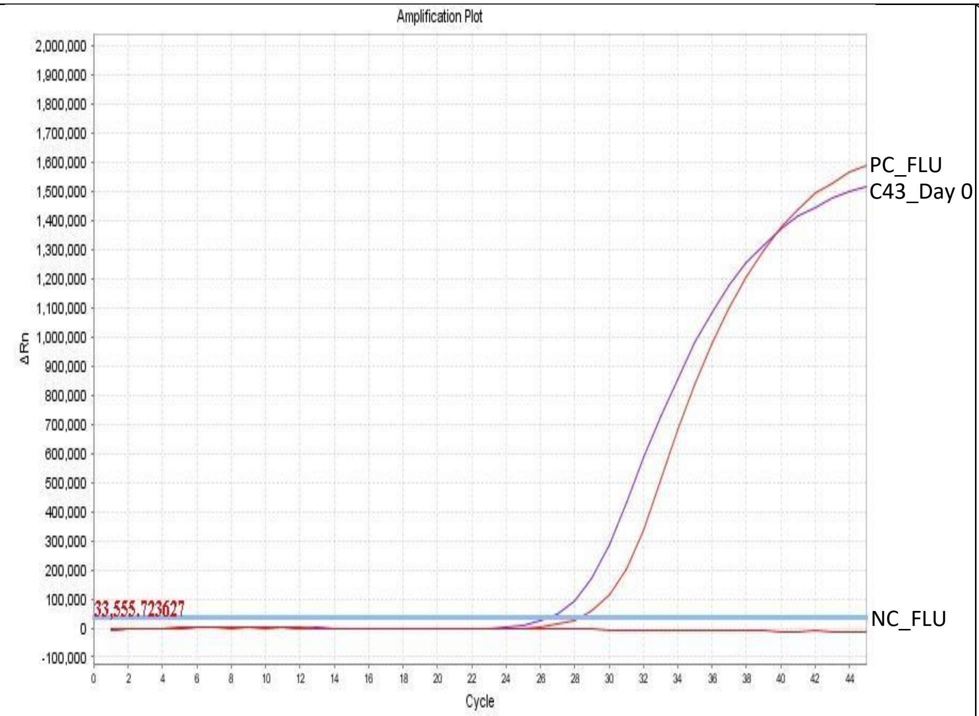Amplification Plot<br>PC_FLU<br>C43_Day 0<br>NC_FLU<br>33,555.723627<br>ΔRn<br>Cycle	

	C43_D2	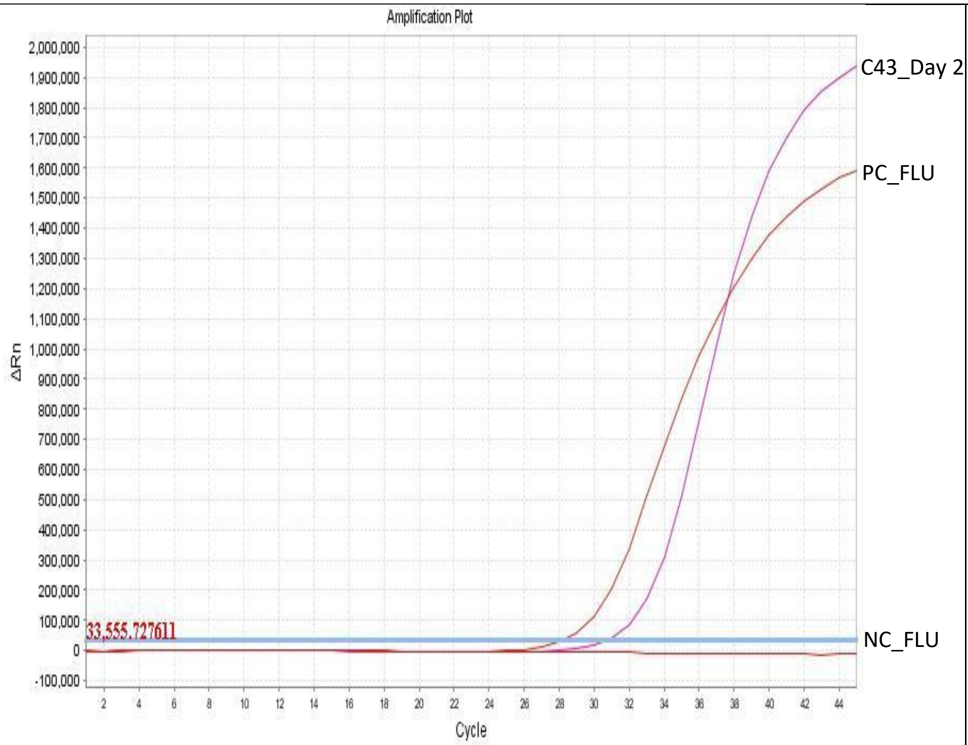	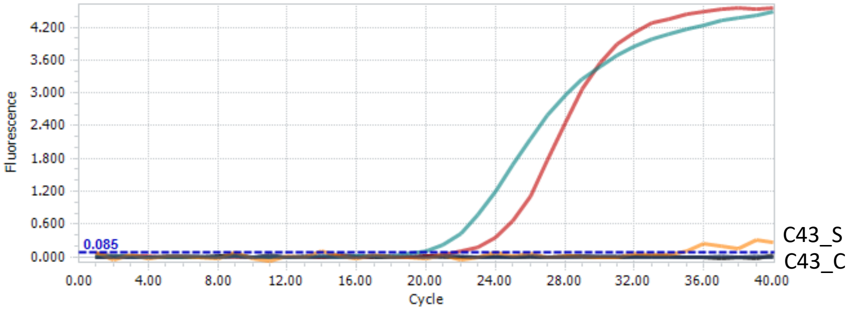 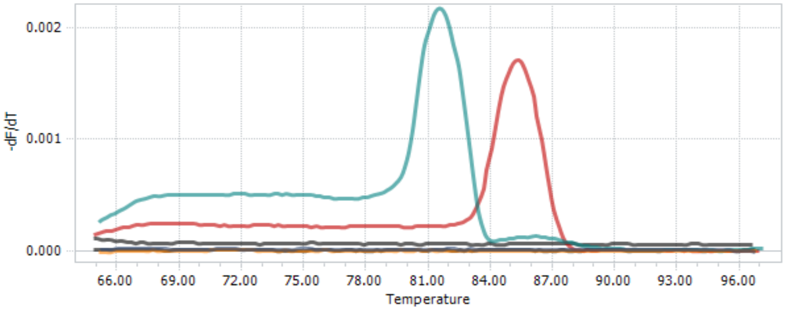

Amplification Plot
ΔRn
Cycle
33,555.727611
C43_Day 2
PC_FLU
NC_FLU

Fluorescence
Cycle
0.085
C43_S
C43_C

-dF/dT
Temperature

40	C44_D0	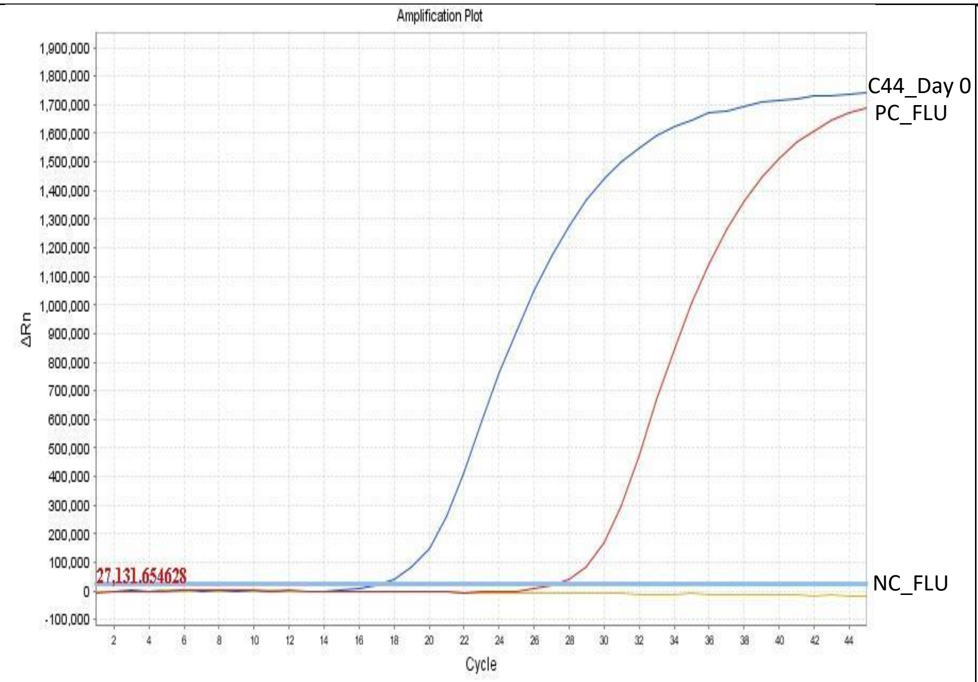Amplification Plot
ΔRn
Cycle
27,131.654628
C44_Day 0
PC_FLU
NC_FLU	

C44_D2

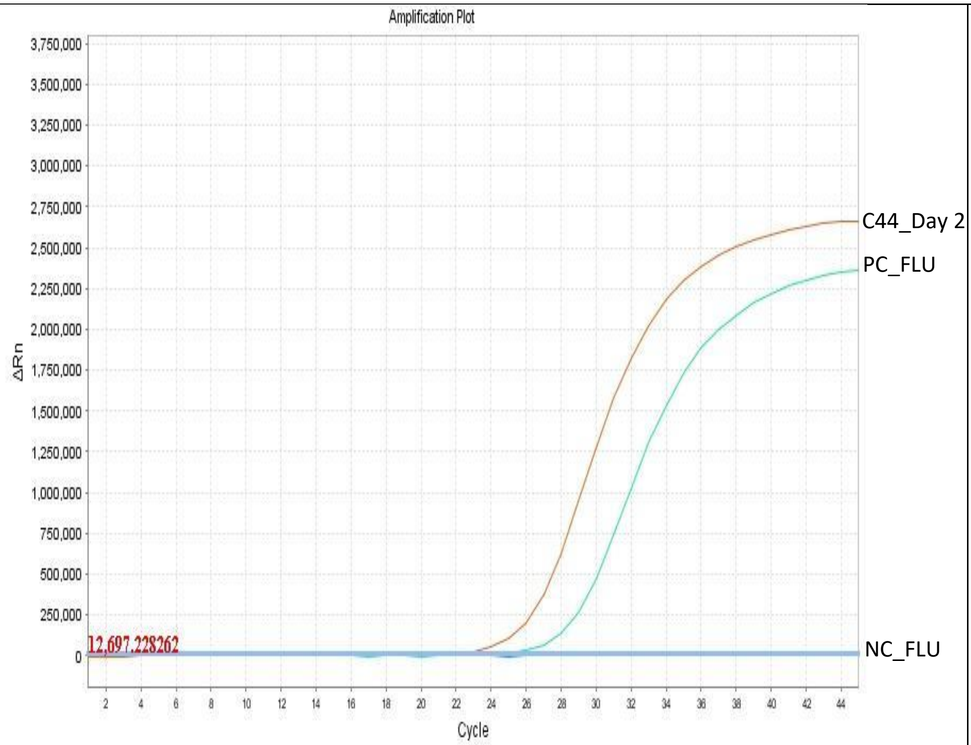

Amplification Plot
ΔRn
Cycle
12,697.228262
C44_Day 2
PC_FLU
NC_FLU

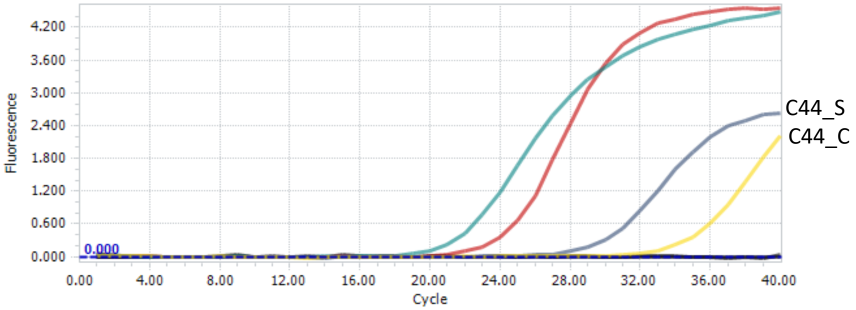

Fluorescence
Cycle
C44_S
C44_C

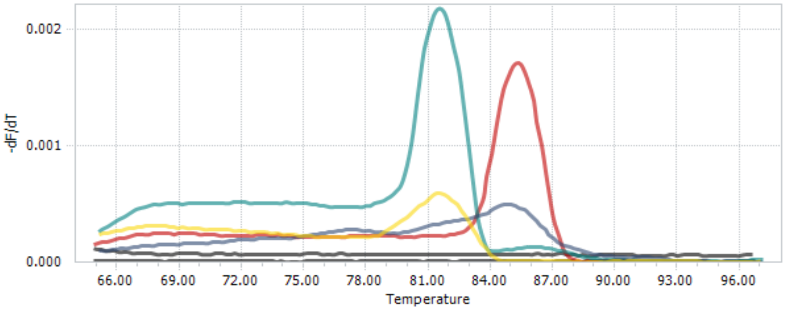

-dF/dT
Temperature

41	C45 _D0	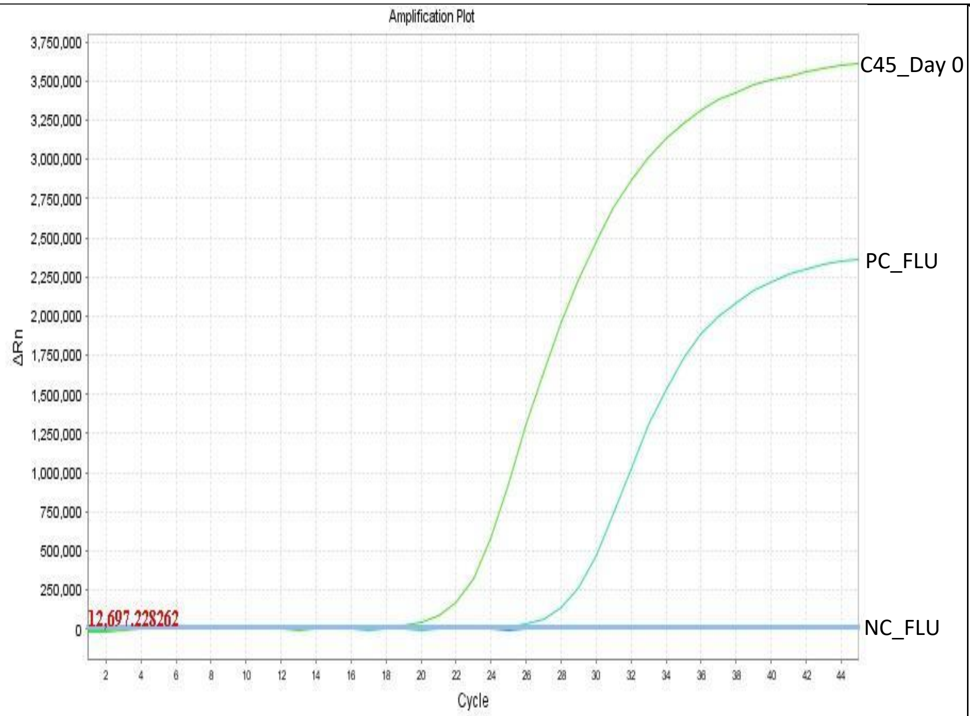Amplification Plot<br>3,750,000<br>3,500,000<br>3,250,000<br>3,000,000<br>2,750,000<br>2,500,000<br>2,250,000<br>2,000,000<br>1,750,000<br>1,500,000<br>1,250,000<br>1,000,000<br>750,000<br>500,000<br>250,000<br>0<br>ΔRn<br>12,697.228262<br>2 4 6 8 10 12 14 16 18 20 22 24 26 28 30 32 34 36 38 40 42 44<br>Cycle<br>C45_Day 0<br>PC_FLU<br>NC_FLU	

	C45_D2	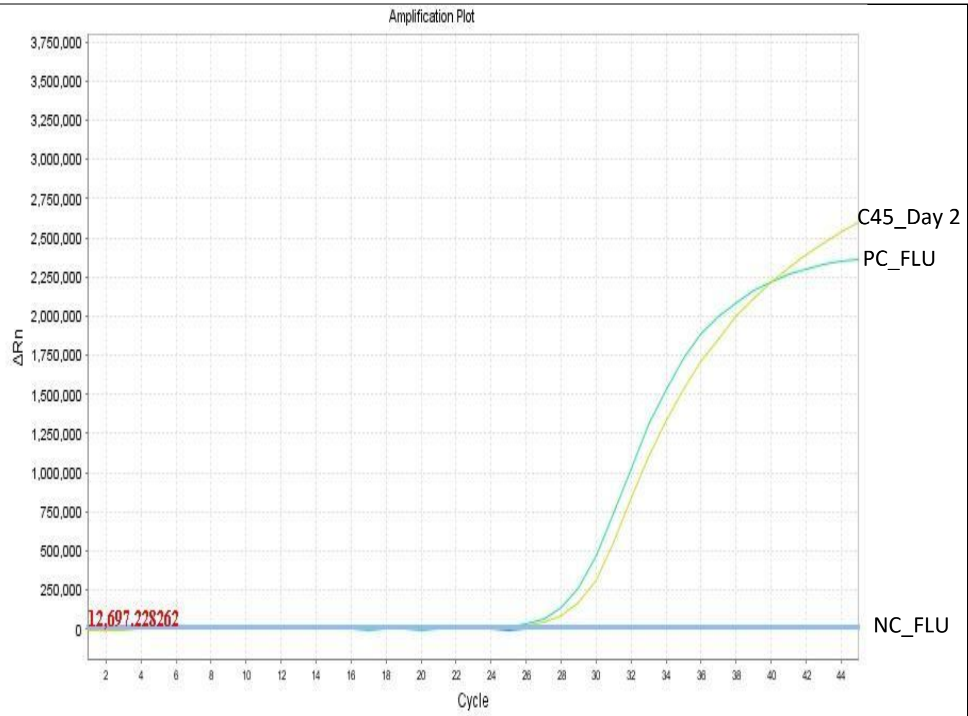 Amplification Plot<br>ΔRn<br>Cycle<br>12,697.228262<br>C45_Day 2<br>PC_FLU<br>NC_FLU	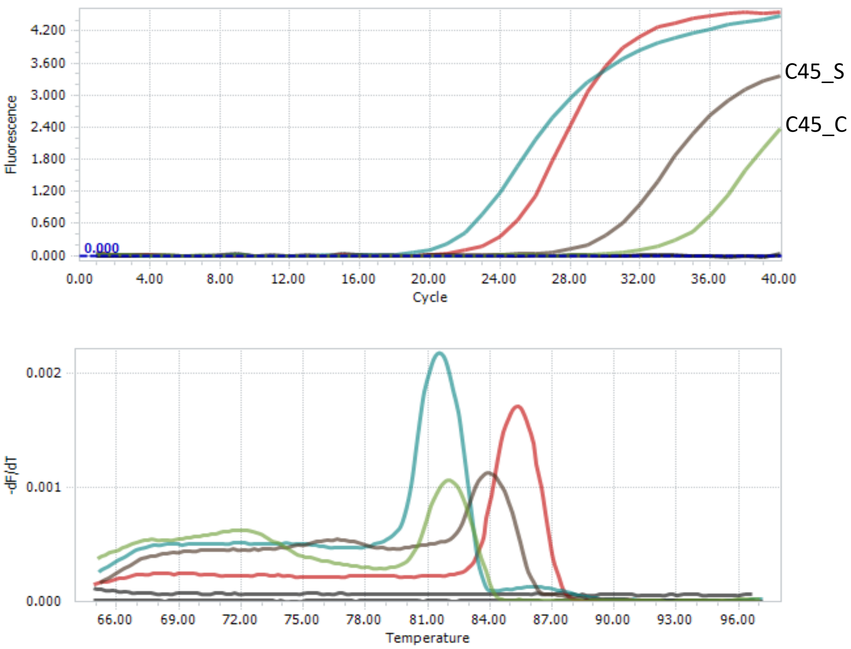 Fluorescence<br>Cycle<br>C45_S<br>C45_C<br>-dF/dT<br>Temperature

42	C47 _D0	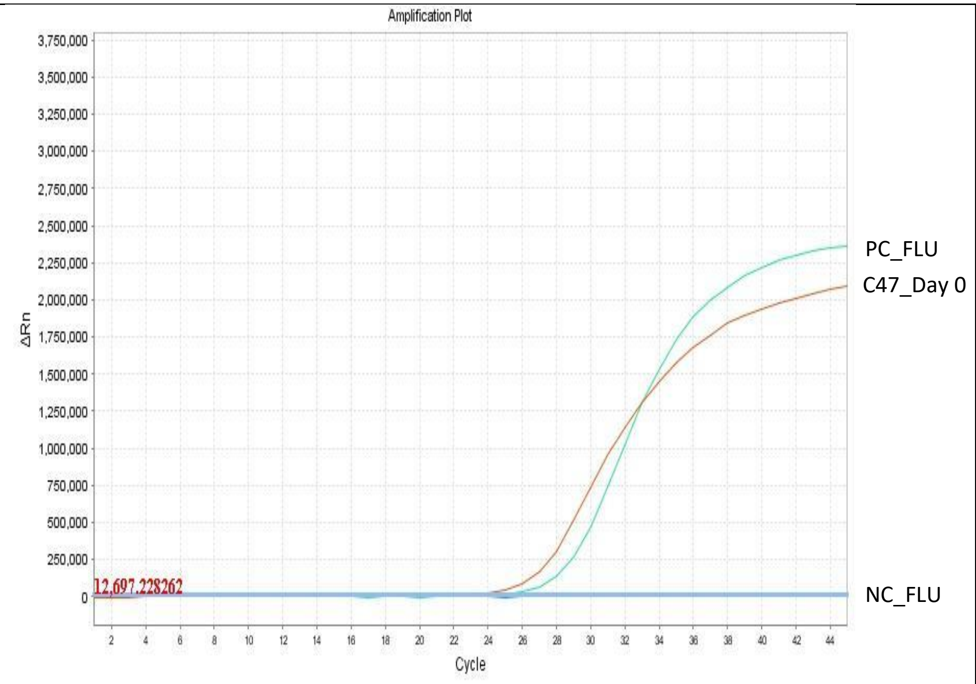Amplification Plot
ΔRn
Cycle
12,697.228262
PC_FLU
C47_Day 0
NC_FLU	

<table>
<tr>
<td></td>
<td>C47_D2</td>
<td>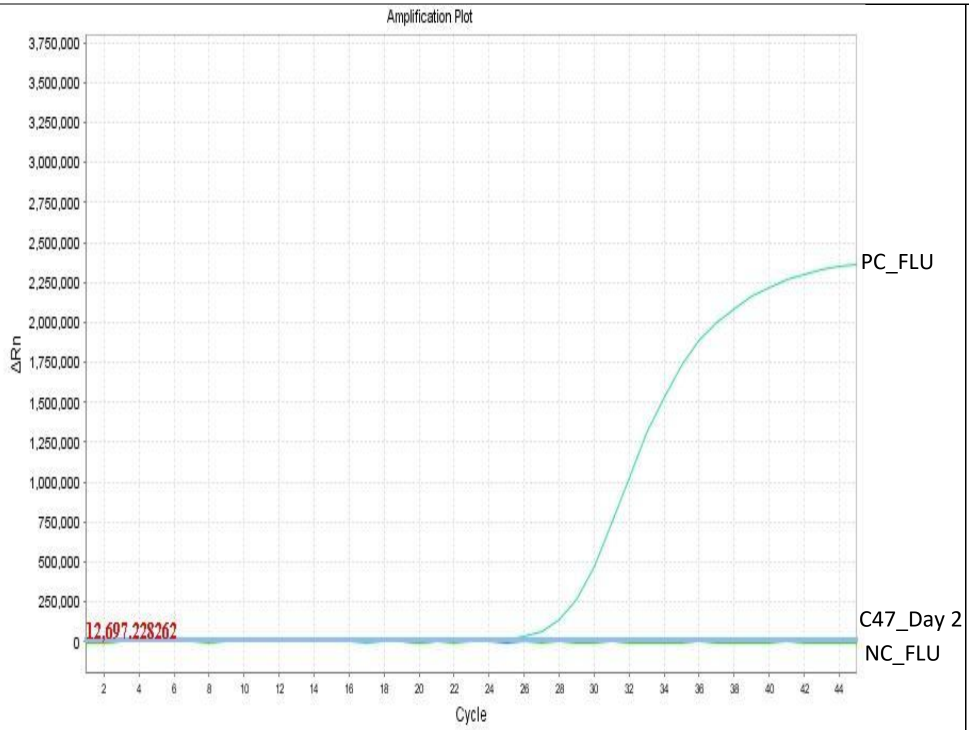
</td>
<td>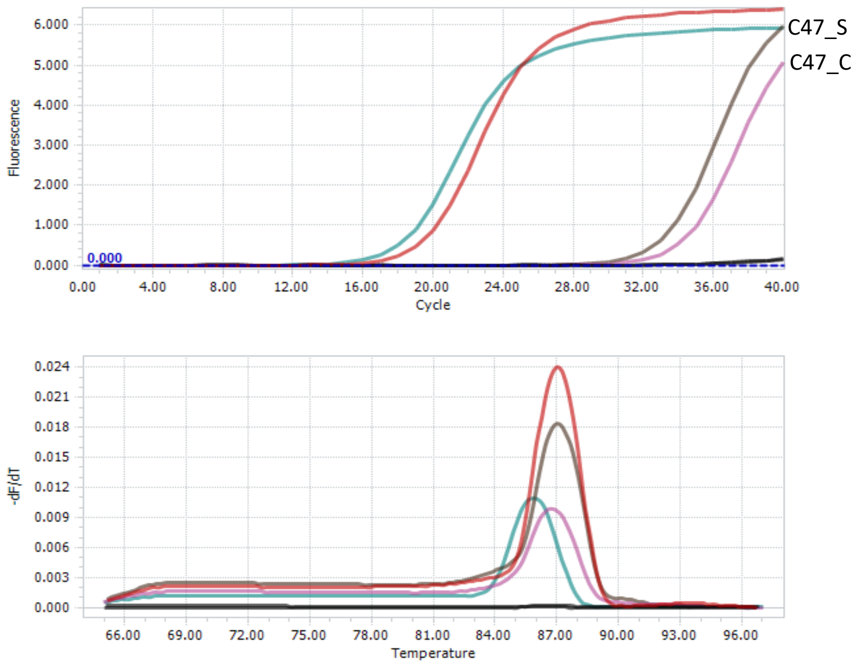
</td>
</tr>
</table>

43	C50 _D0	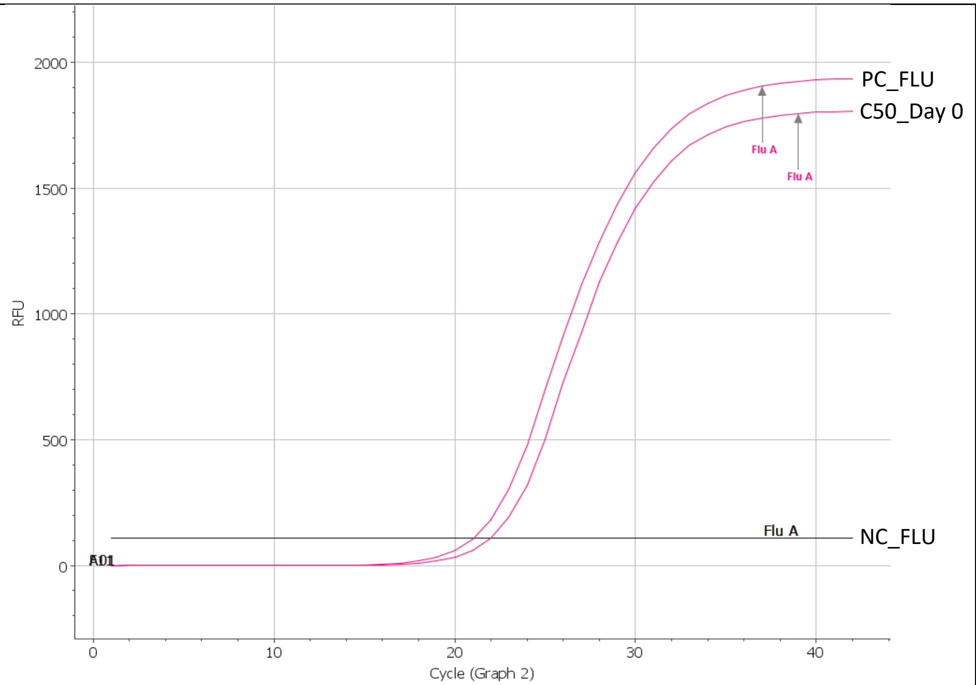2000
1500
1000
500
0
RFU
0
10
20
30
40
Cycle (Graph 2)
PC_FLU
C50_Day 0
Flu A
Flu A
Flu A
NC_FLU
A01	

	C50_D2	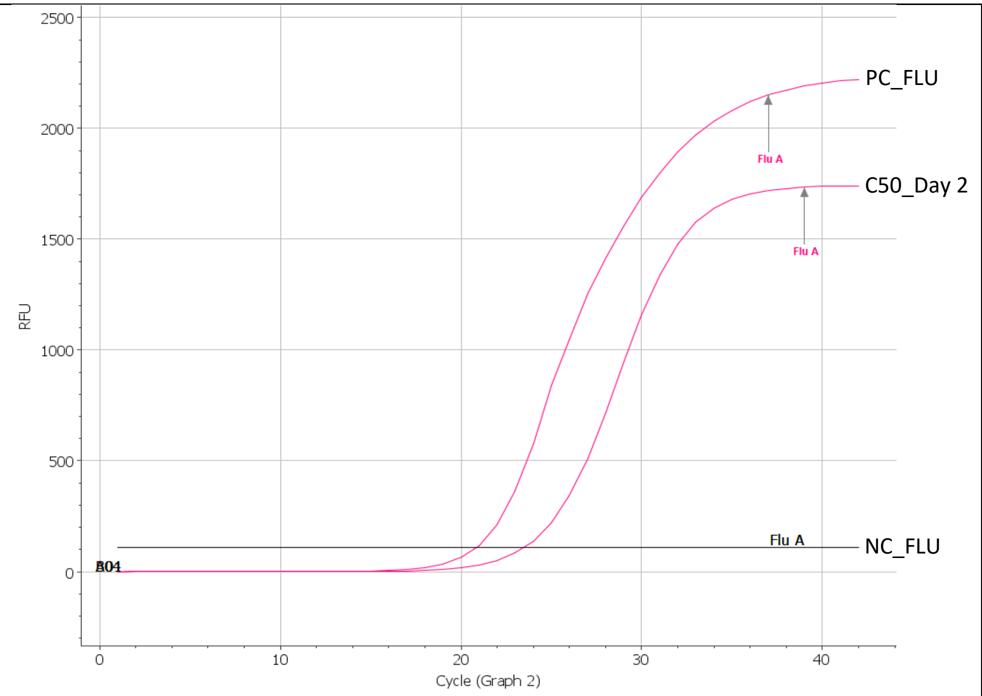 RFU<br>Cycle (Graph 2)<br>PC_FLU<br>C50_Day 2<br>NC_FLU<br>Flu A	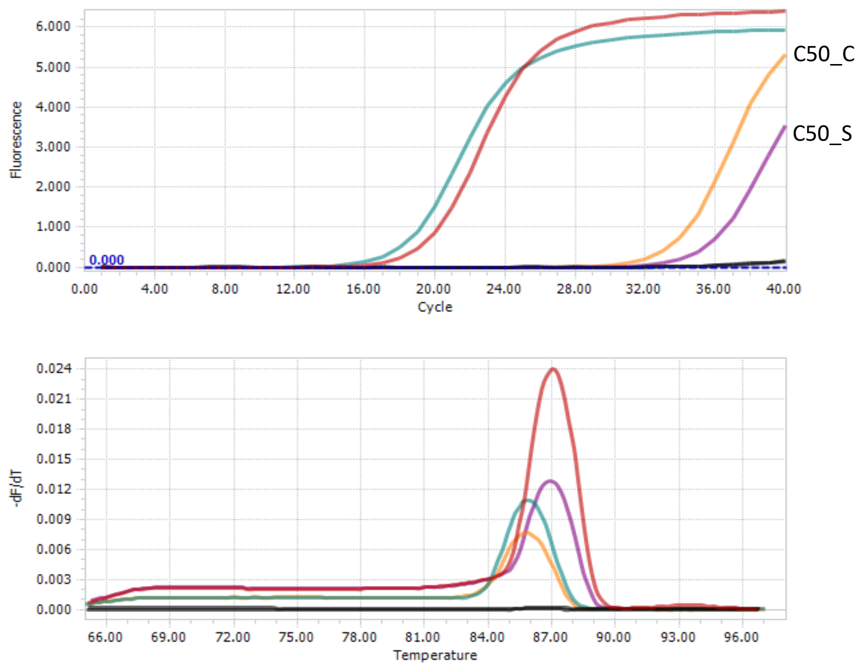 Fluorescence<br>Cycle<br>C50_C<br>C50_S<br>-dF/dT<br>Temperature

44	C51 _D0	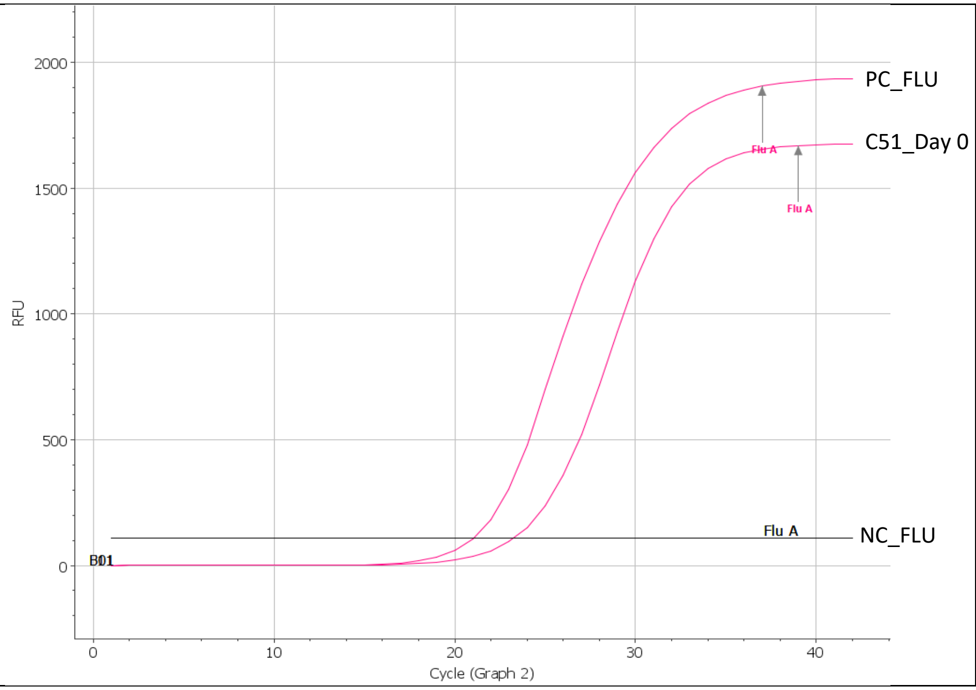PC_FLU<br>C51_Day 0<br>NC_FLU<br>Flu A<br>Flu A<br>Flu A<br>B01<br>RFU<br>Cycle (Graph 2)<br>2000<br>1500<br>1000<br>500<br>0<br>0<br>10<br>20<br>30<br>40	

C51_D2

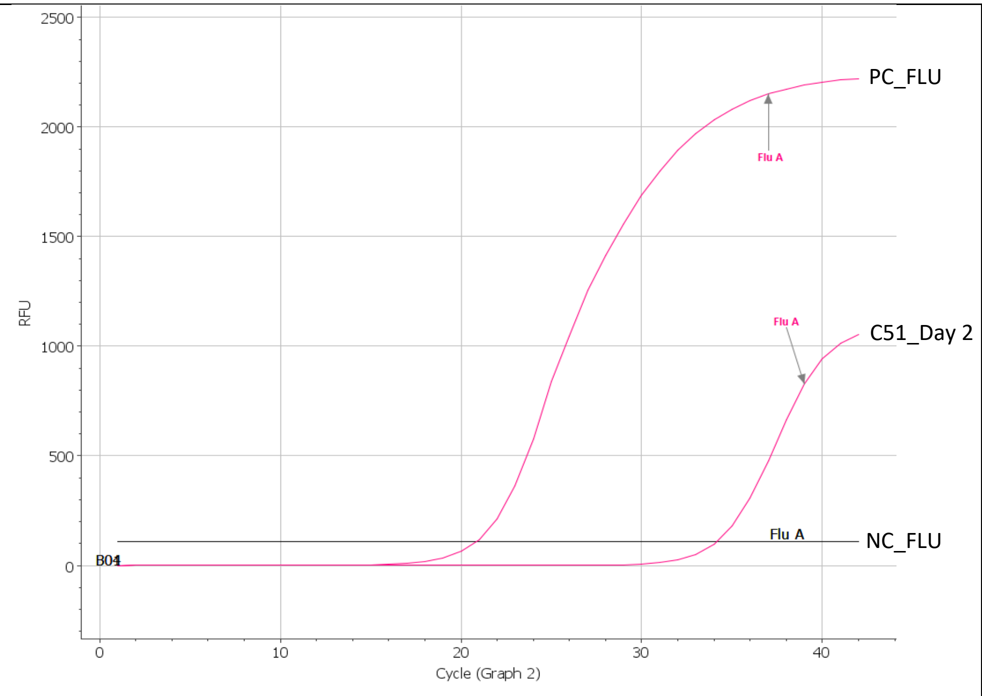
RFU
Cycle (Graph 2)
PC_FLU
C51_Day 2
NC_FLU
Flu A
B04

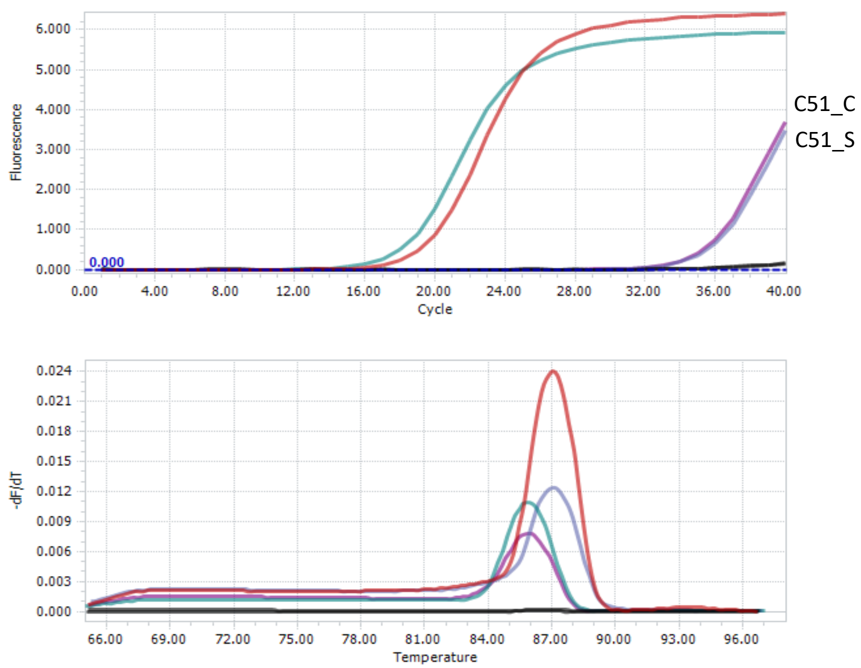
Fluorescence
Cycle
C51_C
C51_S
-dF/dT
Temperature

45	C52<br>_D0	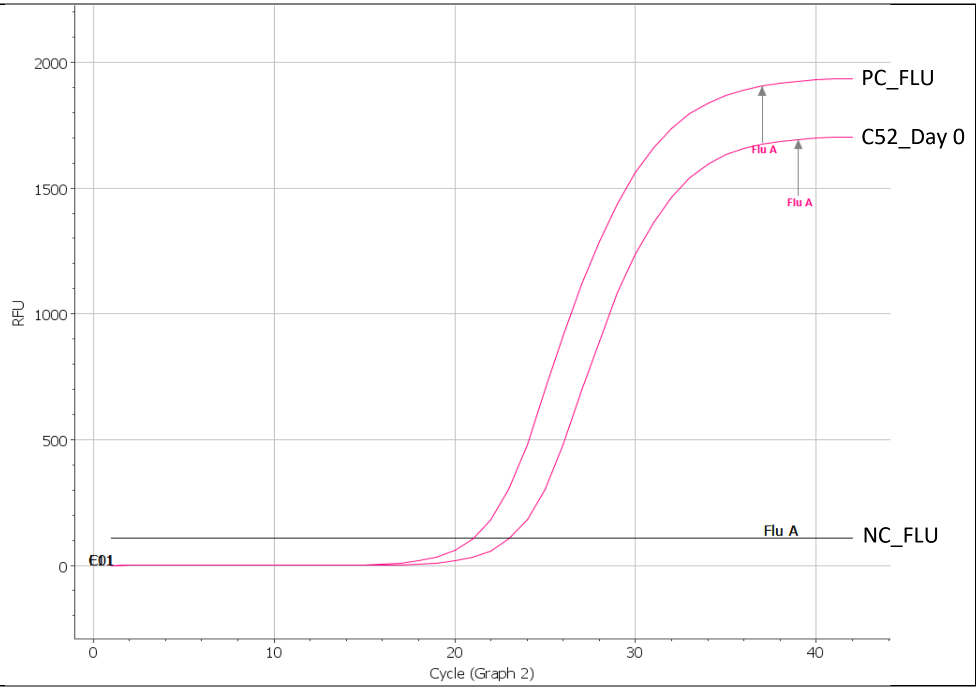2000<br>1500<br>1000<br>500<br>0<br>RFU<br>0<br>10<br>20<br>30<br>40<br>Cycle (Graph 2)<br>PC_FLU<br>C52_Day 0<br>Flu A<br>Flu A<br>Flu A<br>NC_FLU<br>E01	

C52_D2

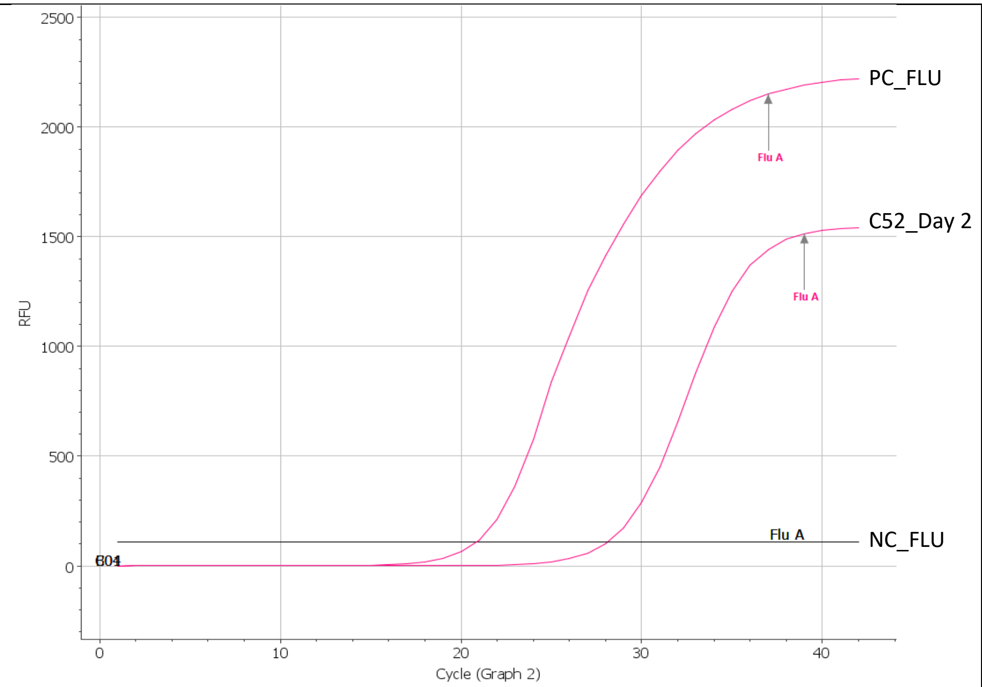
2500
2000
1500
1000
500
0
RFU
0
10
20
30
40
Cycle (Graph 2)
PC_FLU
Flu A
C52_Day 2
Flu A
Flu A
NC_FLU

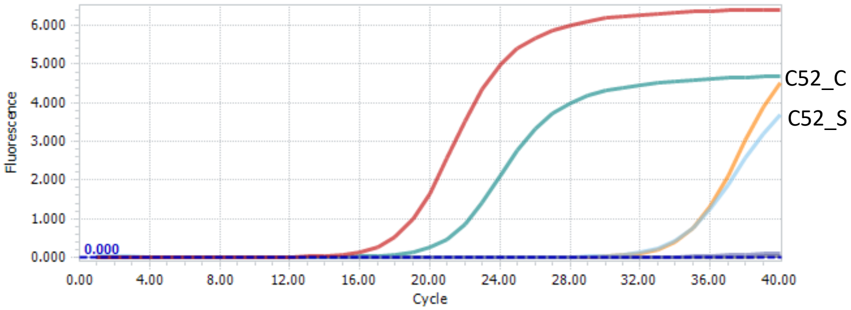
6.000
5.000
4.000
3.000
2.000
1.000
0.000
Fluorescence
0.000
0.00
4.00
8.00
12.00
16.00
20.00
24.00
28.00
32.00
36.00
40.00
Cycle
C52_C
C52_S

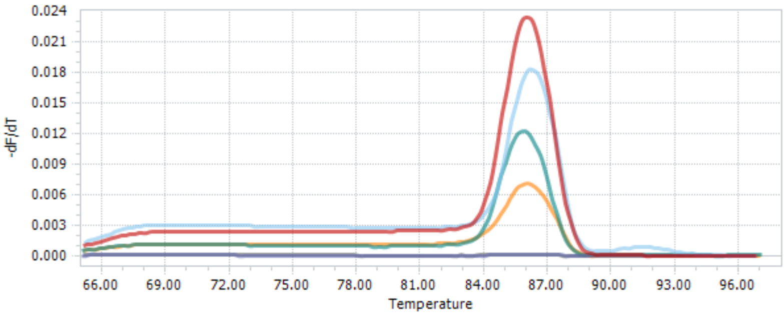
0.024
0.021
0.018
0.015
0.012
0.009
0.006
0.003
0.000
-dF/dT
66.00
69.00
72.00
75.00
78.00
81.00
84.00
87.00
90.00
93.00
96.00
Temperature

46	C53_D0	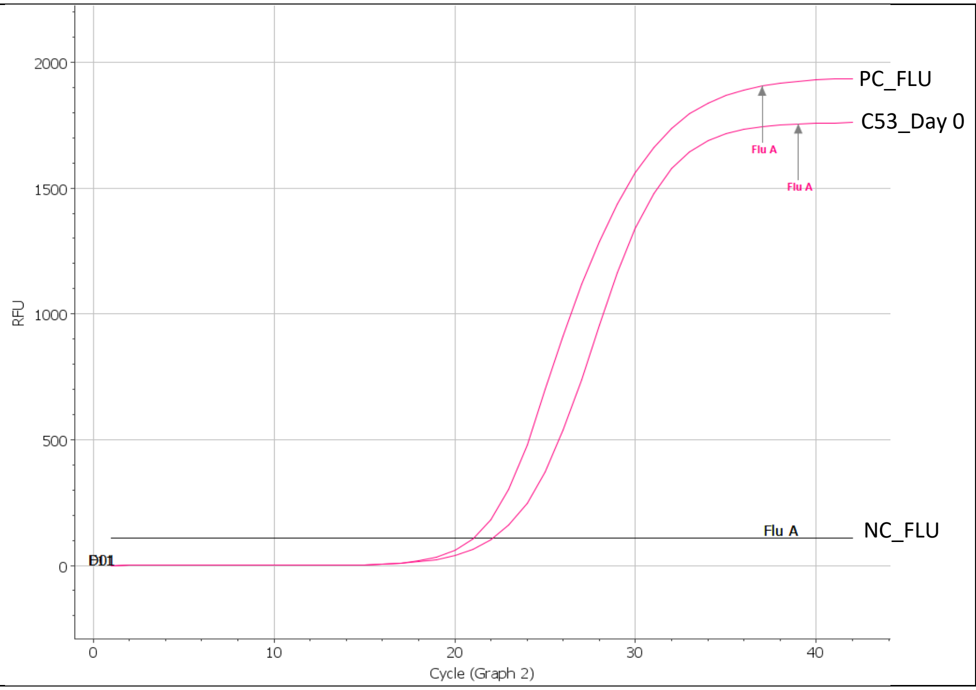 RFU<br>2000<br>1500<br>1000<br>500<br>0<br>0<br>10<br>20<br>30<br>40<br>Cycle (Graph 2)<br>Flu A<br>Flu A<br>Flu A<br>D01<br>PC_FLU<br>C53_Day 0<br>NC_FLU	

	C53_D2	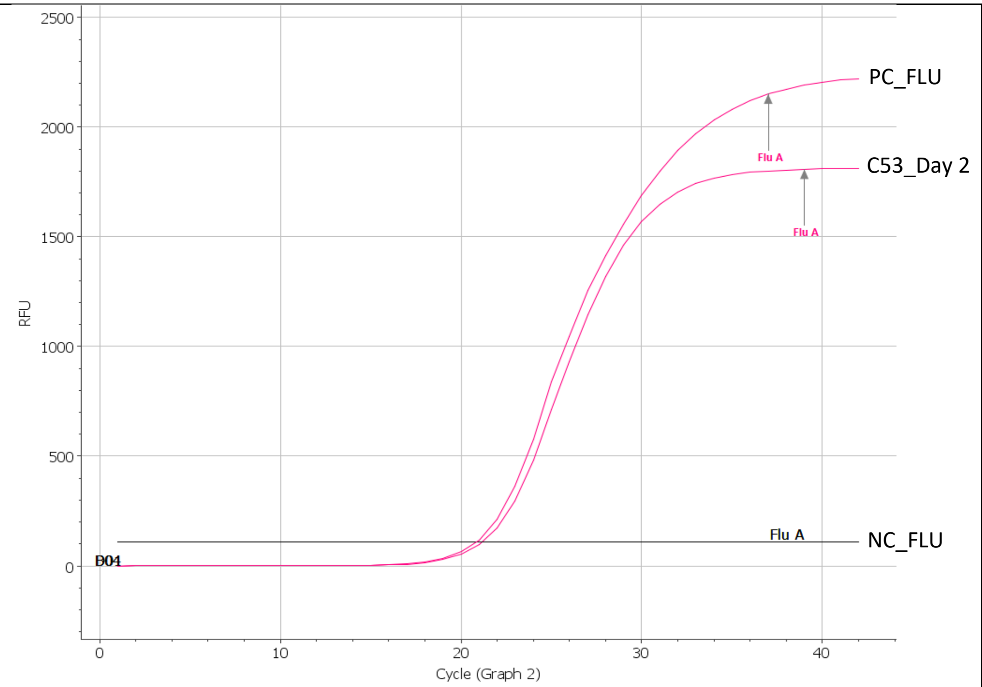	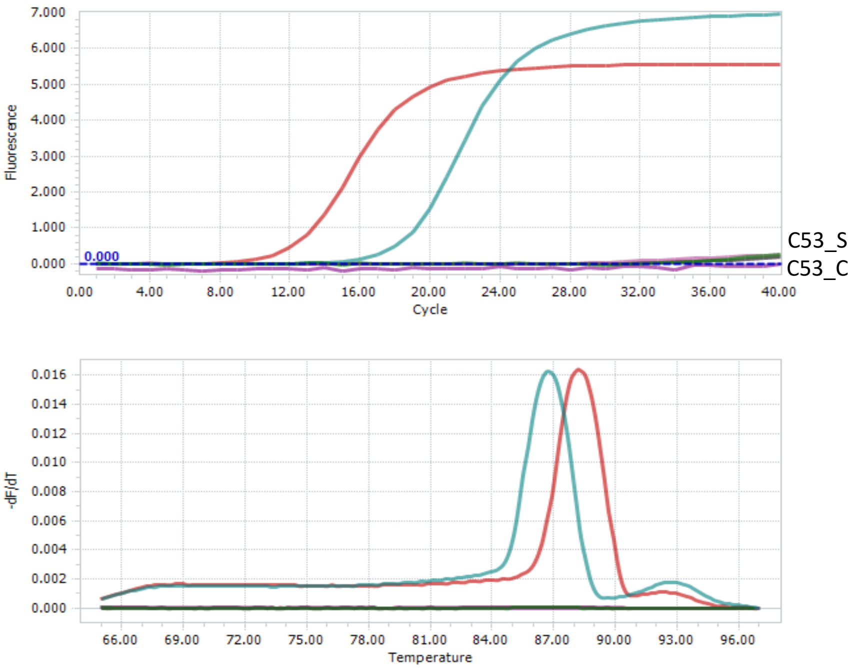

2500
2000
1500
1000
500
0
RFU
0
10
20
30
40
Cycle (Graph 2)
PC_FLU
Flu A
C53_Day 2
Flu A
Flu A
NC_FLU
D04

7.000
6.000
5.000
4.000
3.000
2.000
1.000
0.000
Fluorescence
0.000
0.00
4.00
8.00
12.00
16.00
20.00
24.00
28.00
32.00
36.00
40.00
Cycle
C53_S
C53_C
0.016
0.014
0.012
0.010
0.008
0.006
0.004
0.002
0.000
-dF/dT
66.00
69.00
72.00
75.00
78.00
81.00
84.00
87.00
90.00
93.00
96.00
Temperature

47	C54_D0	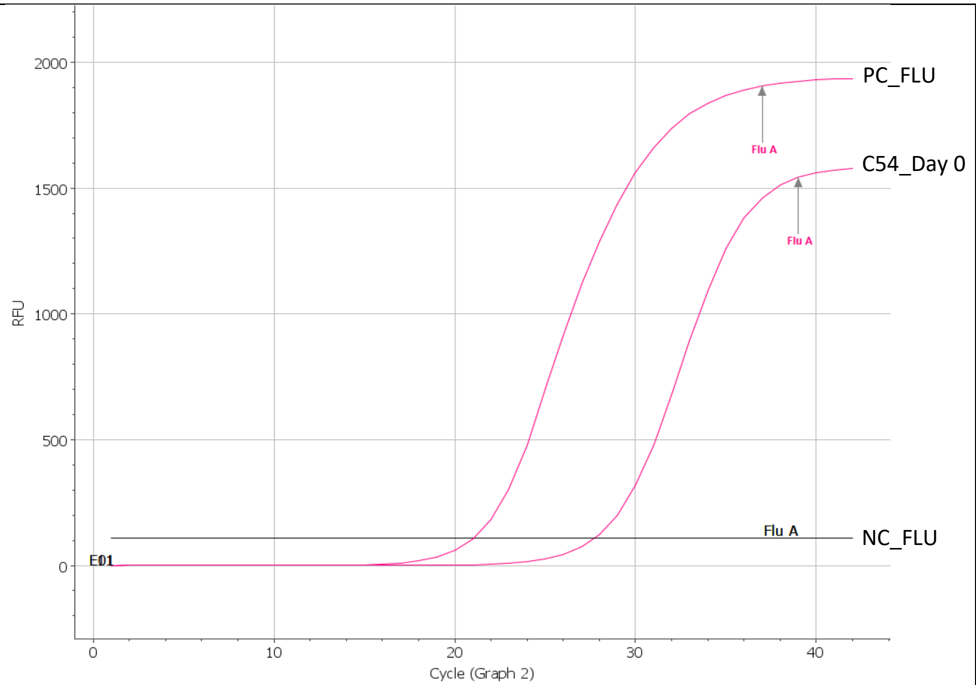	

PC_FLU
C54_Day 0
NC_FLU
Flu A
Flu A
Flu A
E01
RFU
2000
1500
1000
500
0
0
10
20
30
40
Cycle (Graph 2)

	C54_D2	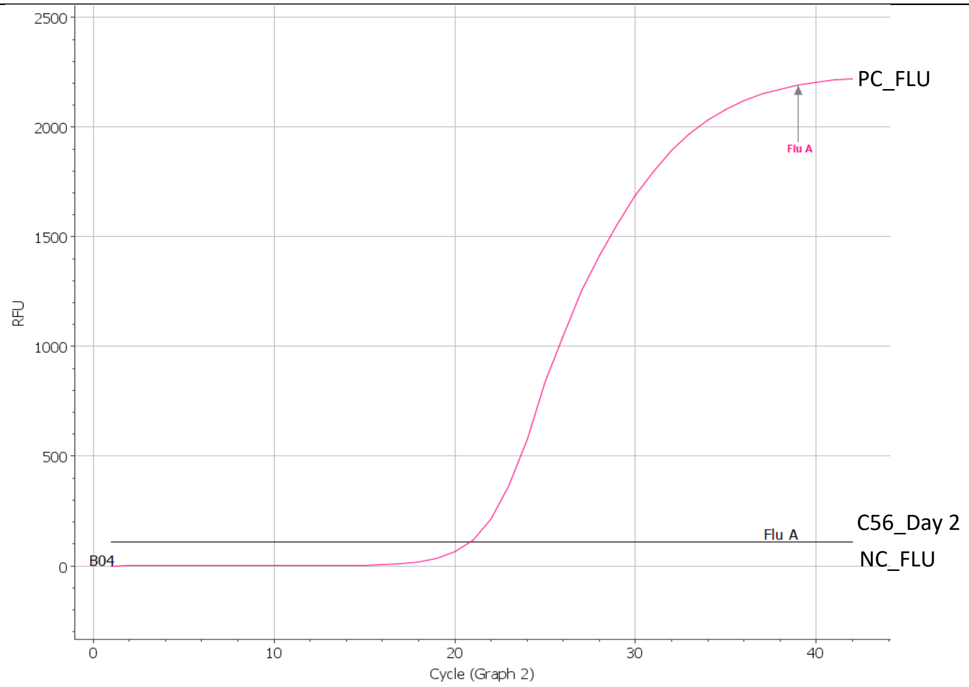 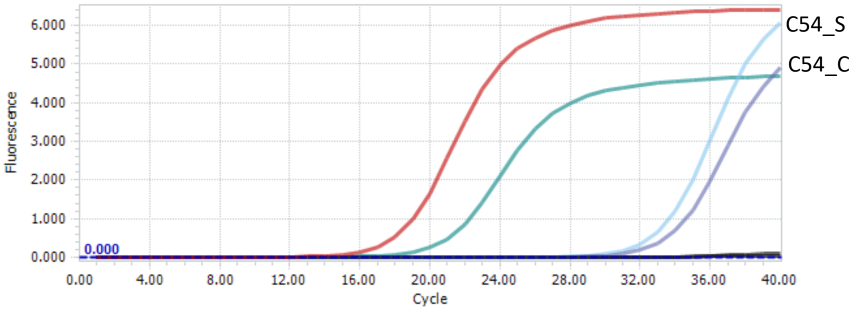 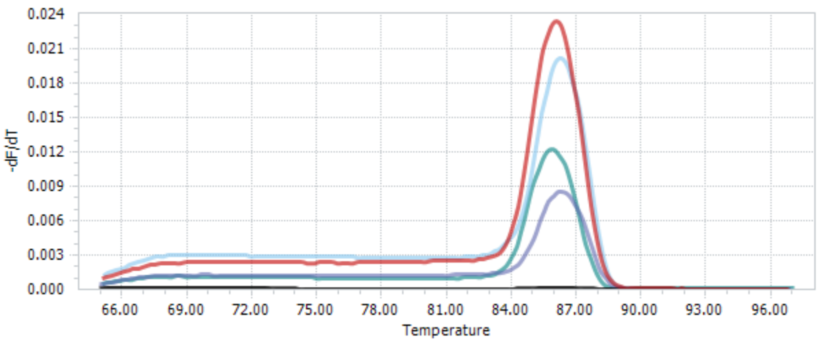

PC_FLU
Flu A
C56_Day 2
NC_FLU
B04
RFU
Cycle (Graph 2)

C54_S
C54_C
Fluorescence
Cycle
0.000

-dF/dT
Temperature

48	C55_D0	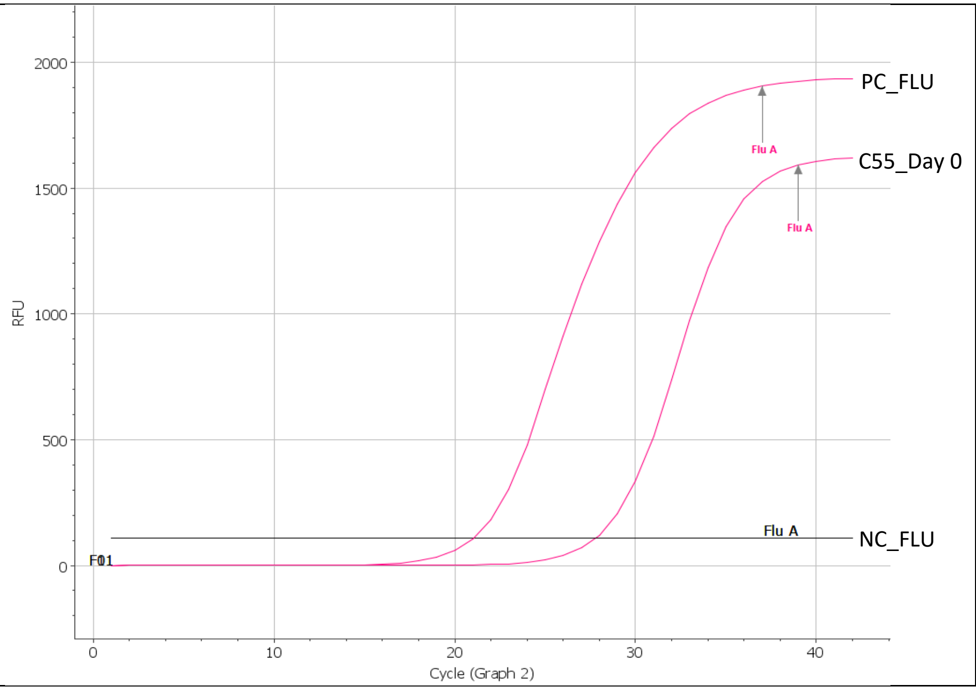2000
1500
1000
500
0
RFU
0
10
20
30
40
Cycle (Graph 2)
Flu A
Flu A
Flu A
F01
PC_FLU
C55_Day 0
NC_FLU	

	C55_D2	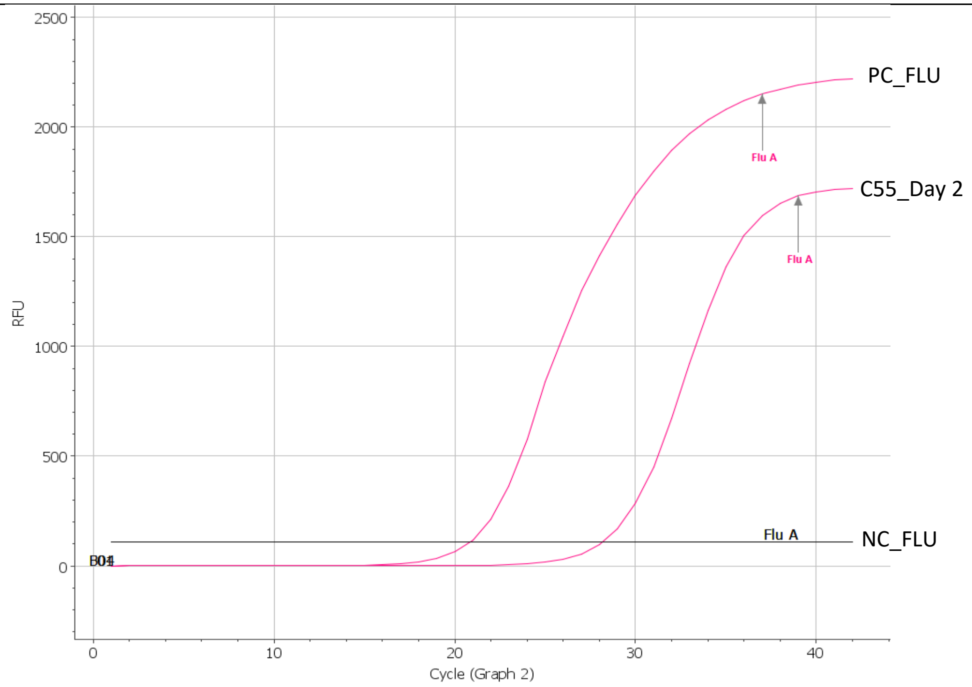	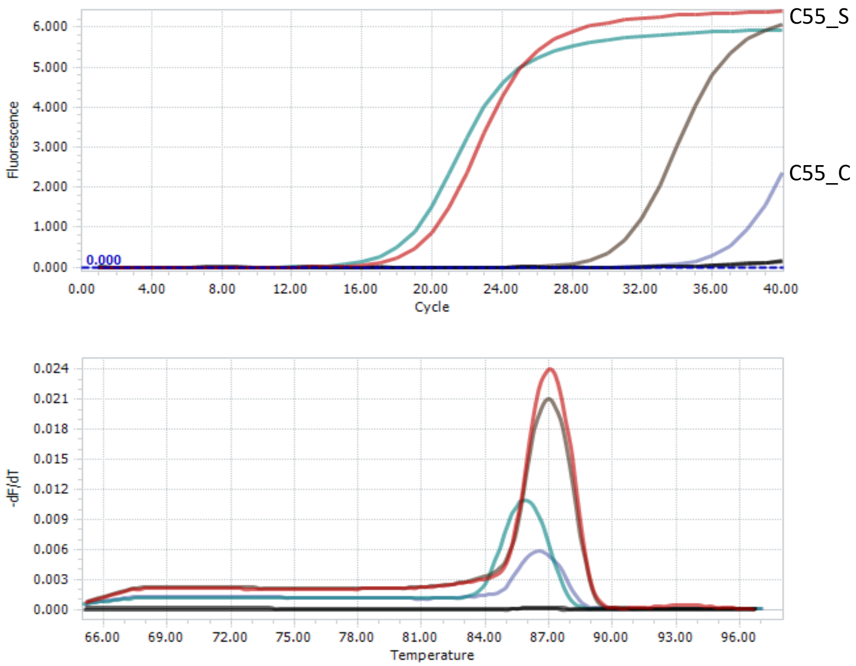

2500
2000
1500
1000
500
0
RFU
0
10
20
30
40
Cycle (Graph 2)
PC_FLU
Flu A
C55_Day 2
Flu A
Flu A
NC_FLU

6.000
5.000
4.000
3.000
2.000
1.000
0.000
Fluorescence
0.000
0.00
4.00
8.00
12.00
16.00
20.00
24.00
28.00
32.00
36.00
40.00
Cycle
C55_S
C55_C
0.024
0.021
0.018
0.015
0.012
0.009
0.006
0.003
0.000
-dF/dT
66.00
69.00
72.00
75.00
78.00
81.00
84.00
87.00
90.00
93.00
96.00
Temperature

49	C59_D0	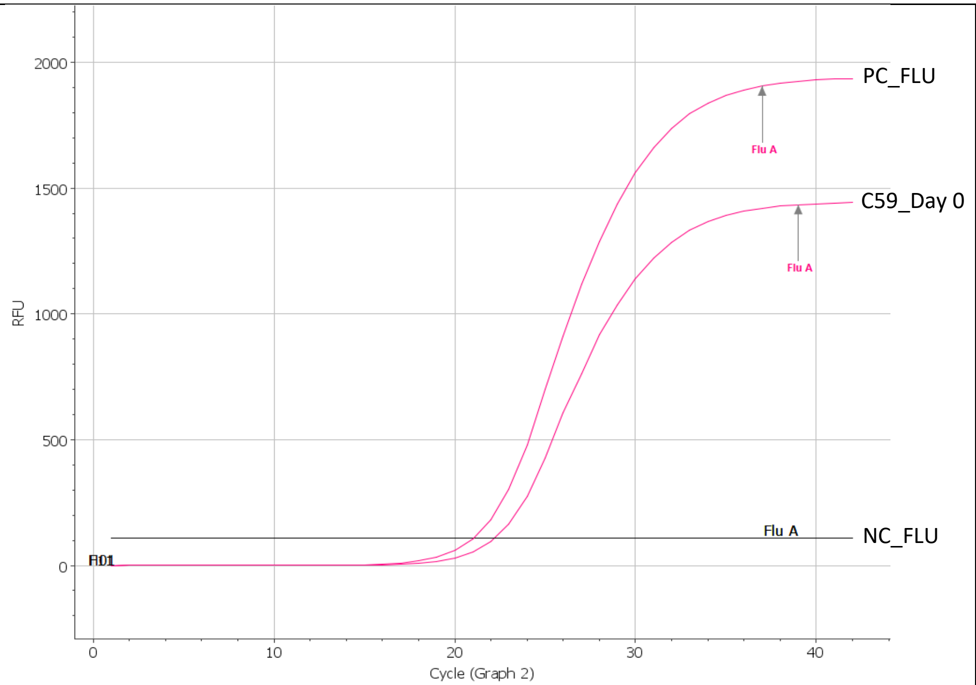2000<br>1500<br>1000<br>500<br>0<br>RFU<br>0<br>10<br>20<br>30<br>40<br>Cycle (Graph 2)<br>PC_FLU<br>Flu A<br>C59_Day 0<br>Flu A<br>Flu A<br>NC_FLU	

	C59_D2	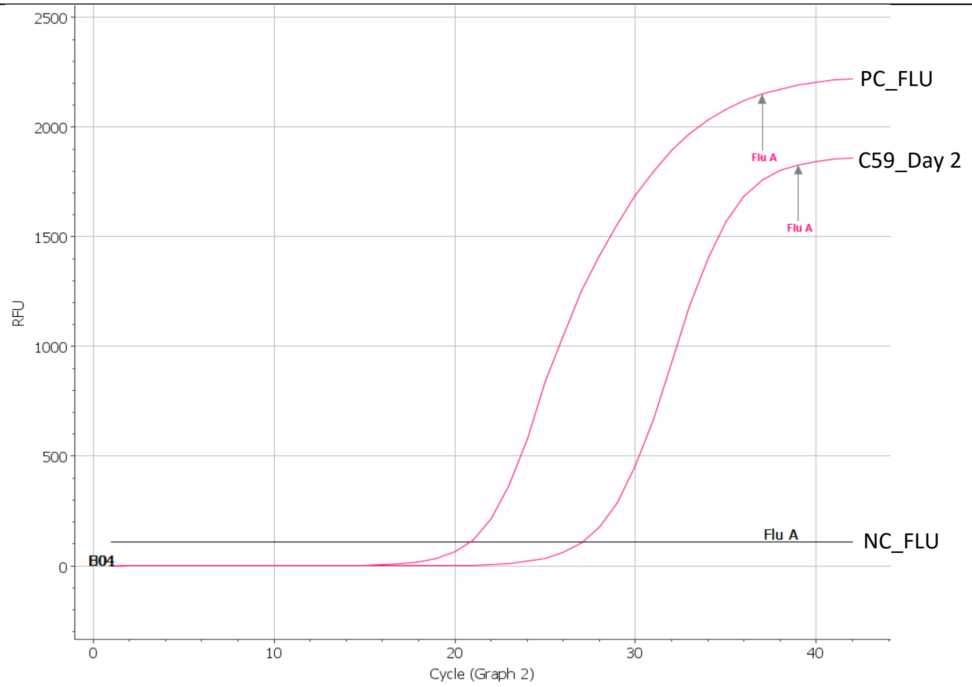	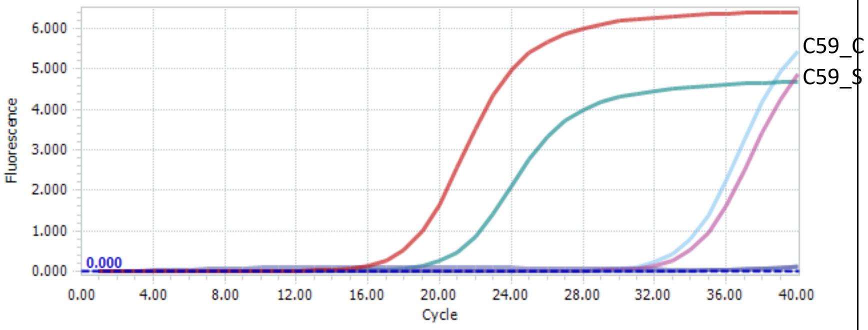 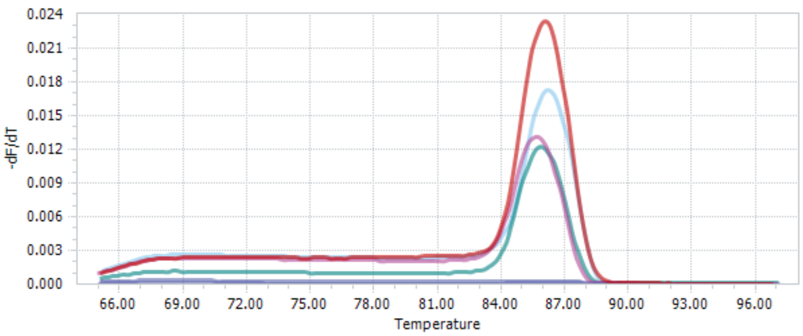

PC_FLU
C59_Day 2
NC_FLU
Flu A
B04
RFU
Cycle (Graph 2)

C59_C
C59_S
Fluorescence
Cycle
0.000

-dF/dT
Temperature

50	C61_D0	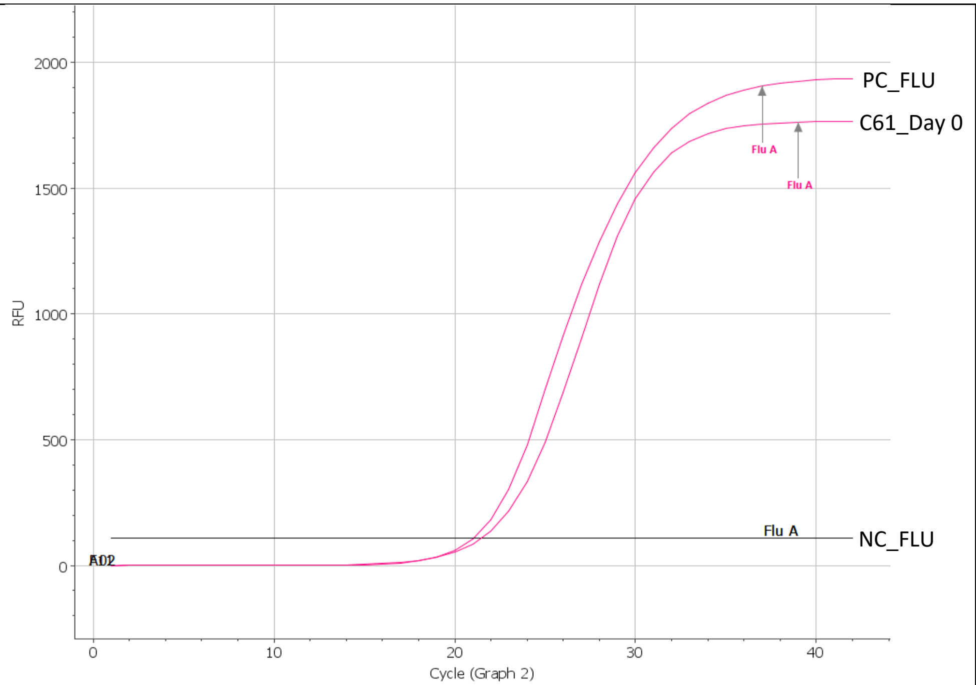	

PC_FLU
C61_Day 0
NC_FLU
Flu A
Flu A
Flu A
RFU
2000
1500
1000
500
0
0
10
20
30
40
Cycle (Graph 2)

	C61_D2	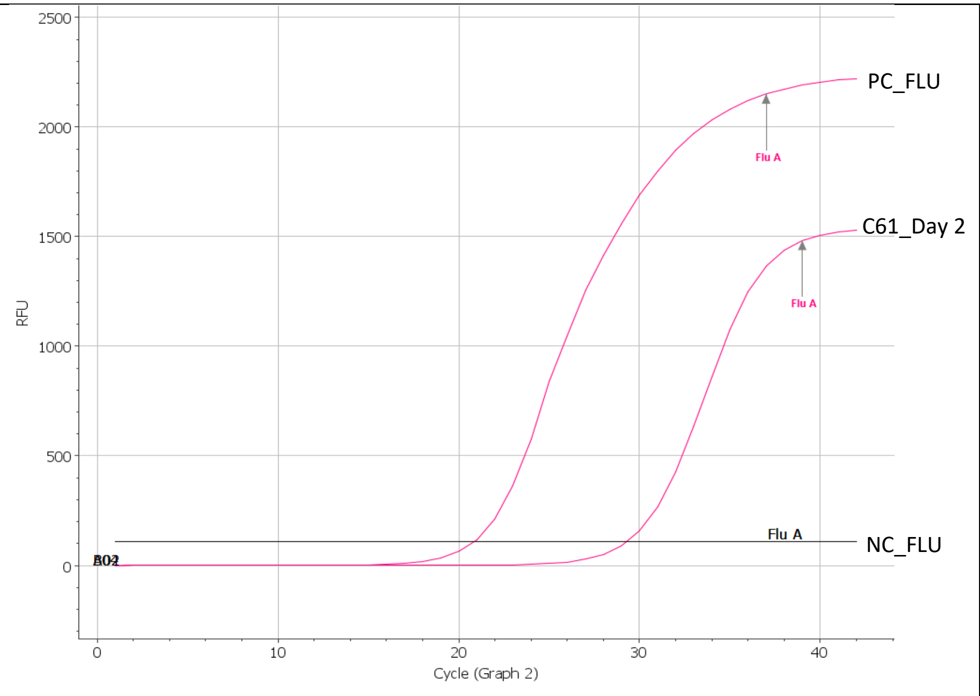RFU<br>2500<br>2000<br>1500<br>1000<br>500<br>0<br>0 10 20 30 40<br>Cycle (Graph 2)<br>PC_FLU<br>C61_Day 2<br>NC_FLU<br>Flu A<br>Flu A<br>Flu A	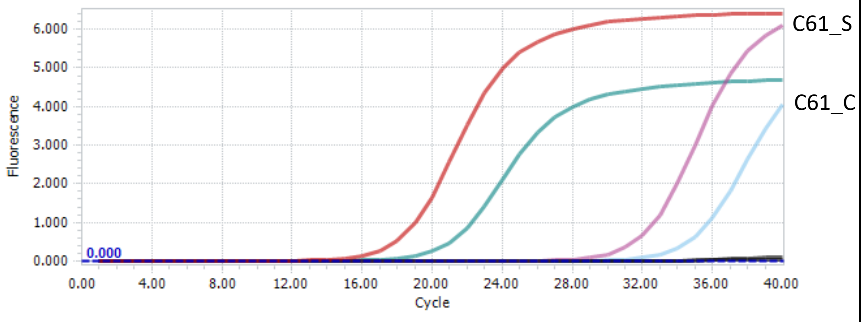Fluorescence<br>6.000<br>5.000<br>4.000<br>3.000<br>2.000<br>1.000<br>0.000<br>0.000<br>0.00 4.00 8.00 12.00 16.00 20.00 24.00 28.00 32.00 36.00 40.00<br>Cycle<br>C61_S<br>C61_C<br>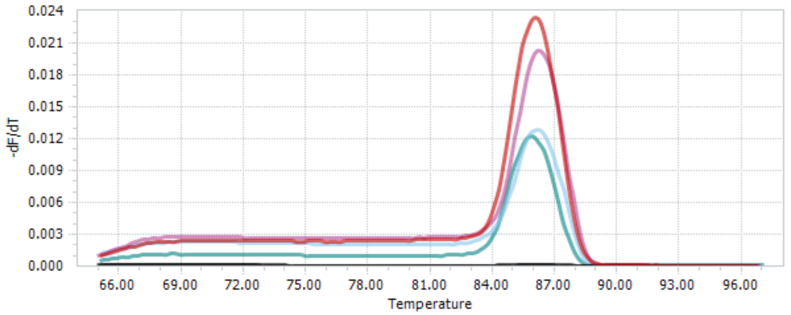-dF/dT<br>0.024<br>0.021<br>0.018<br>0.015<br>0.012<br>0.009<br>0.006<br>0.003<br>0.000<br>66.00 69.00 72.00 75.00 78.00 81.00 84.00 87.00 90.00 93.00 96.00<br>Temperature

51	C62_D0	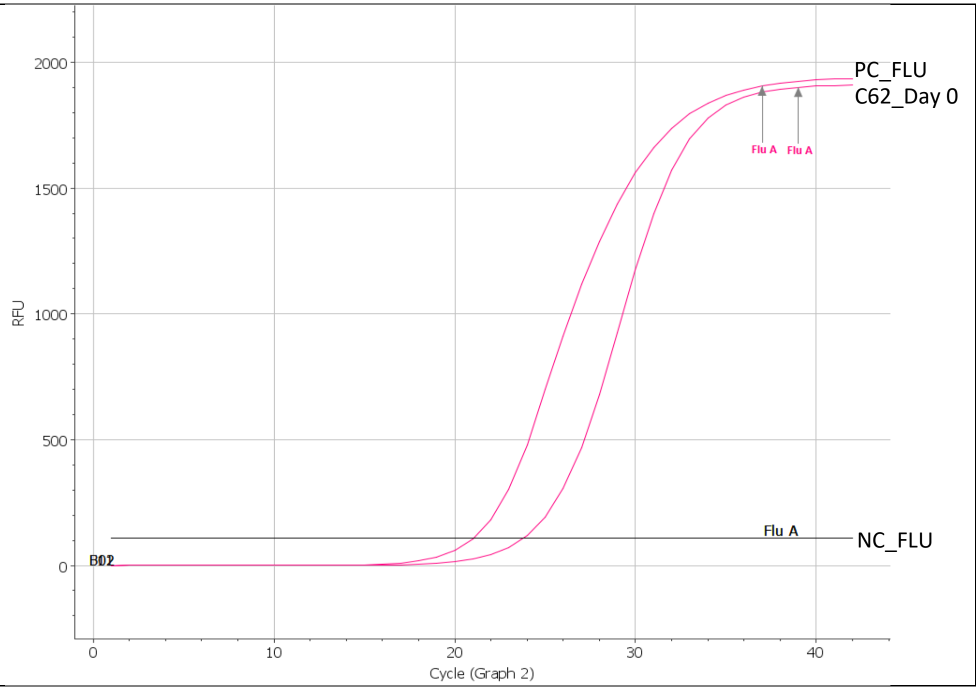2000
1500
1000
500
0
RFU
0
10
20
30
40
Cycle (Graph 2)
PC_FLU
C62_Day 0
Flu A
Flu A
Flu A
NC_FLU	

	C62_D2	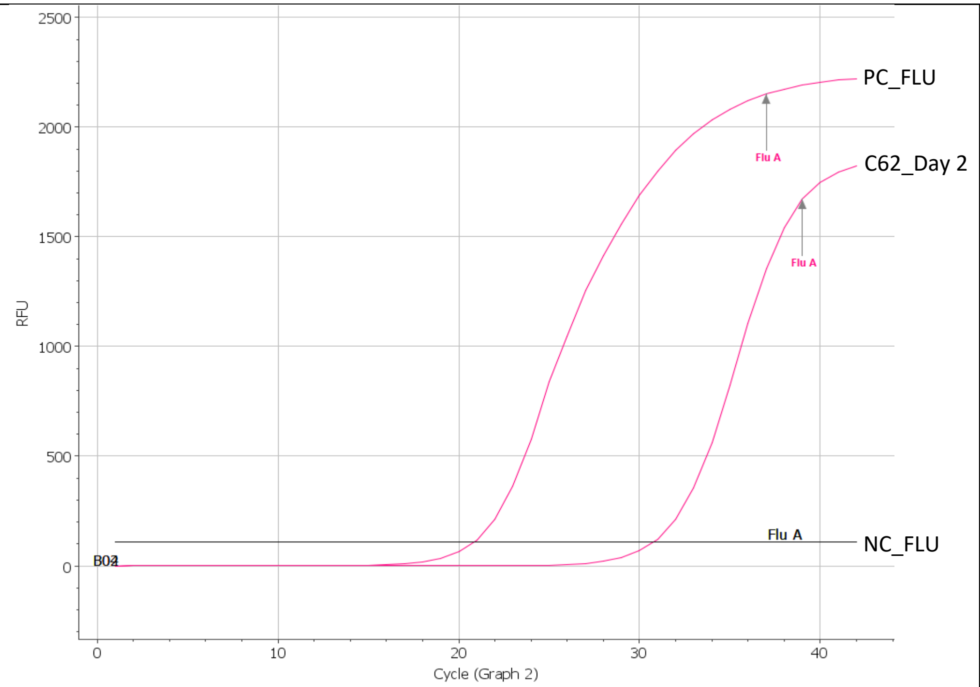 PC_FLU<br>C62_Day 2<br>NC_FLU<br>Flu A<br>Flu A<br>Flu A<br>B04<br>RFU<br>Cycle (Graph 2)	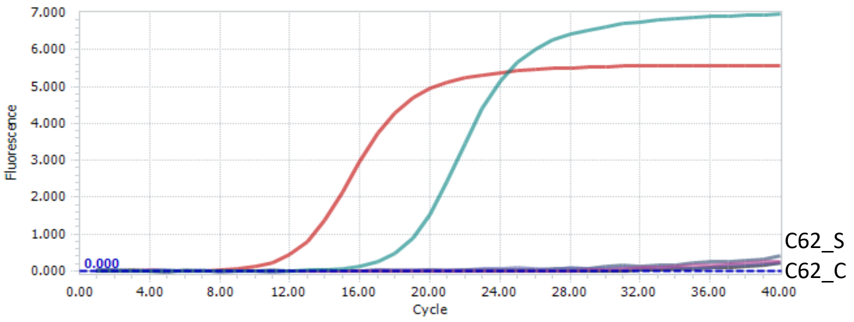 Fluorescence<br>Cycle<br>0.000<br>C62_S<br>C62_C 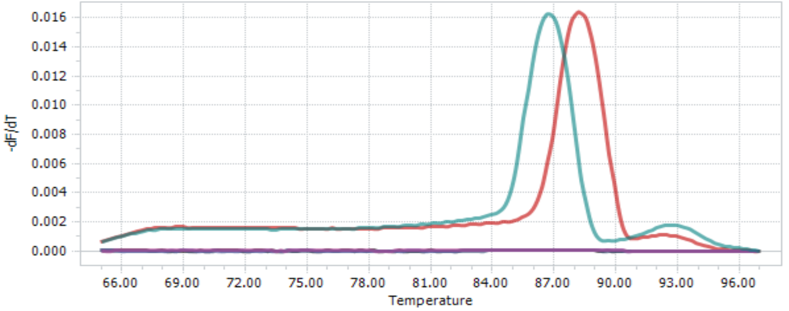 -dF/dT<br>Temperature

52	C66_D0	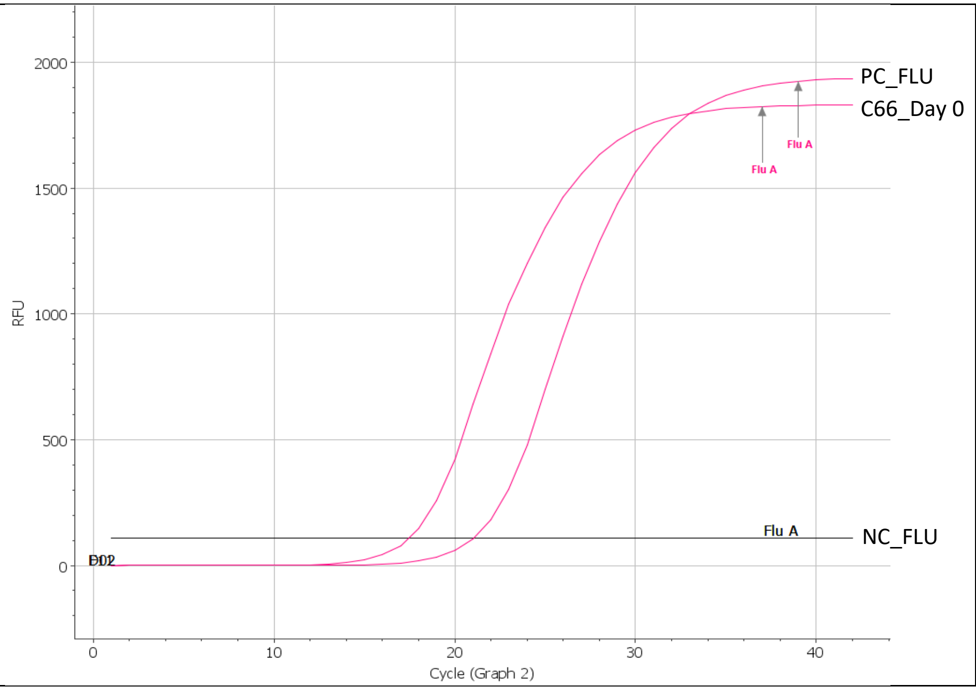2000<br>1500<br>1000<br>500<br>0<br>RFU<br>0<br>10<br>20<br>30<br>40<br>Cycle (Graph 2)<br>Flu A<br>Flu A<br>Flu A<br>PC_FLU<br>C66_Day 0<br>NC_FLU	

	C66_D2	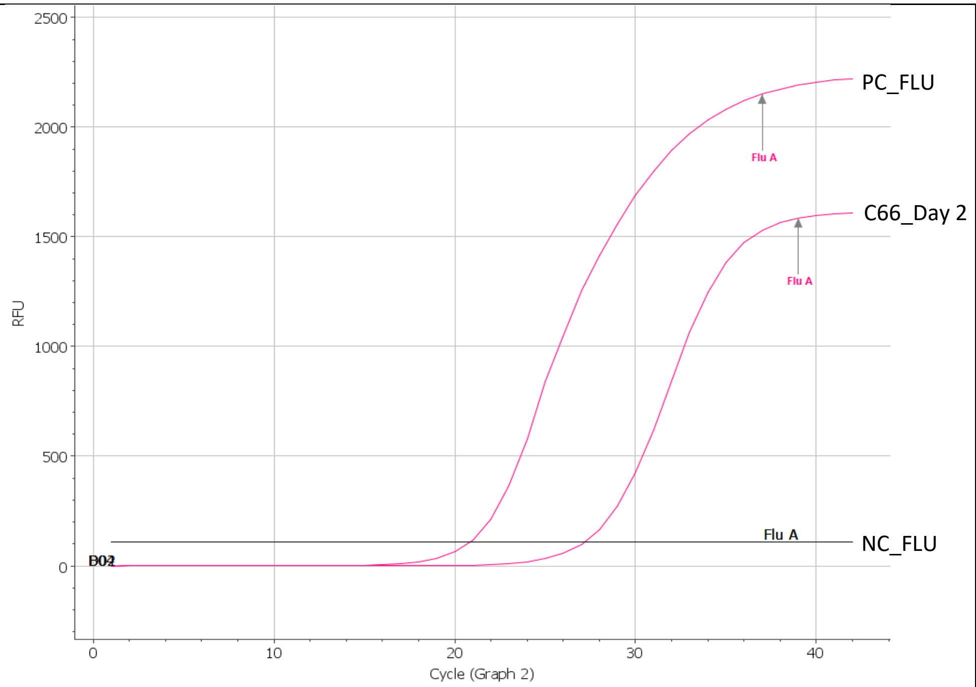PC_FLU<br>C66_Day 2<br>NC_FLU<br>Flu A<br>Flu A<br>Flu A<br>D02<br>RFU<br>Cycle (Graph 2)<br>2500<br>2000<br>1500<br>1000<br>500<br>0<br>0<br>10<br>20<br>30<br>40	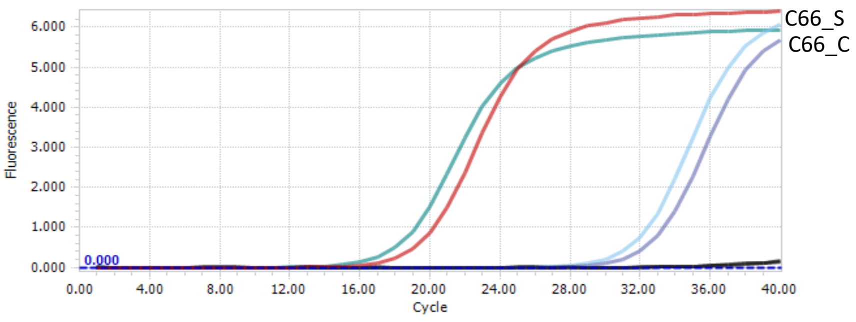C66_S<br>C66_C<br>Fluorescence<br>Cycle<br>0.000<br>6.000<br>5.000<br>4.000<br>3.000<br>2.000<br>1.000<br>0.000<br>0.00<br>4.00<br>8.00<br>12.00<br>16.00<br>20.00<br>24.00<br>28.00<br>32.00<br>36.00<br>40.00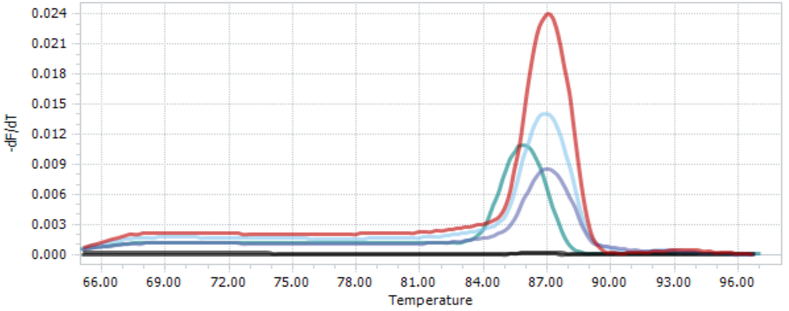-dF/dT<br>Temperature<br>0.024<br>0.021<br>0.018<br>0.015<br>0.012<br>0.009<br>0.006<br>0.003<br>0.000<br>66.00<br>69.00<br>72.00<br>75.00<br>78.00<br>81.00<br>84.00<br>87.00<br>90.00<br>93.00<br>96.00

53	C67_D0	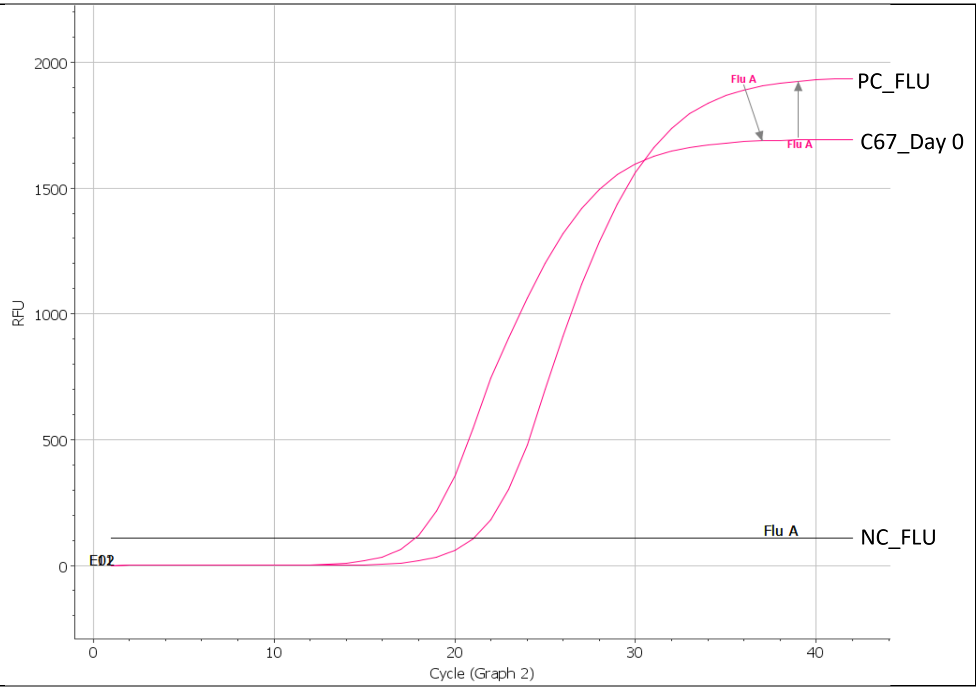2000<br>1500<br>1000<br>500<br>0<br>RFU<br>0<br>10<br>20<br>30<br>40<br>Cycle (Graph 2)<br>Flu A<br>Flu A<br>Flu A<br>PC_FLU<br>C67_Day 0<br>NC_FLU	

C67_D2

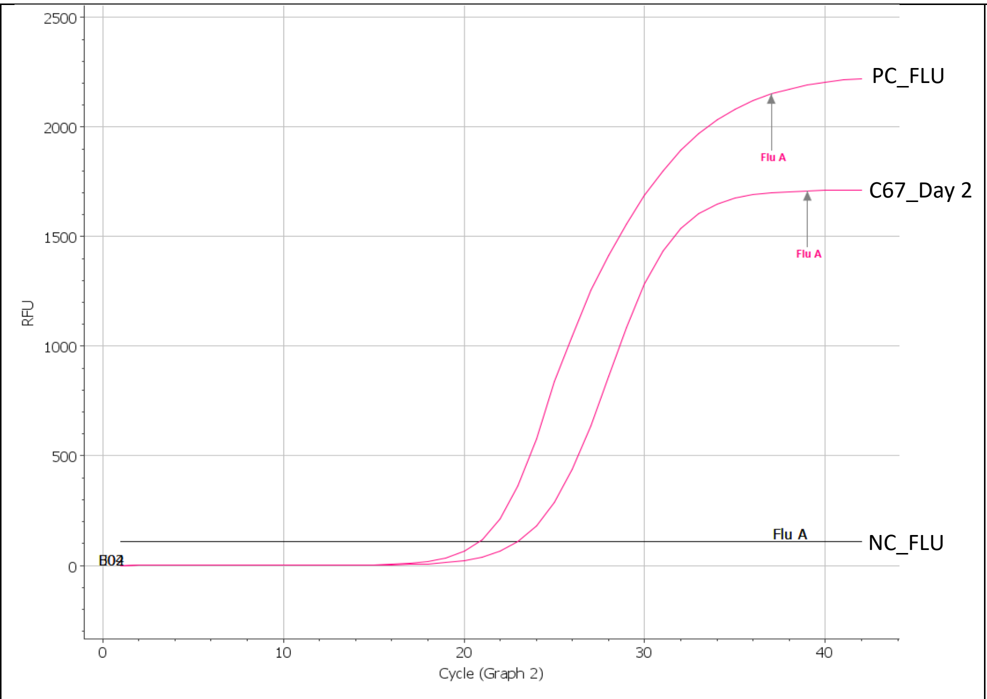
PC_FLU
C67_Day 2
NC_FLU
Flu A
Flu A
Flu A
B02
RFU
2500
2000
1500
1000
500
0
0
10
20
30
40
Cycle (Graph 2)

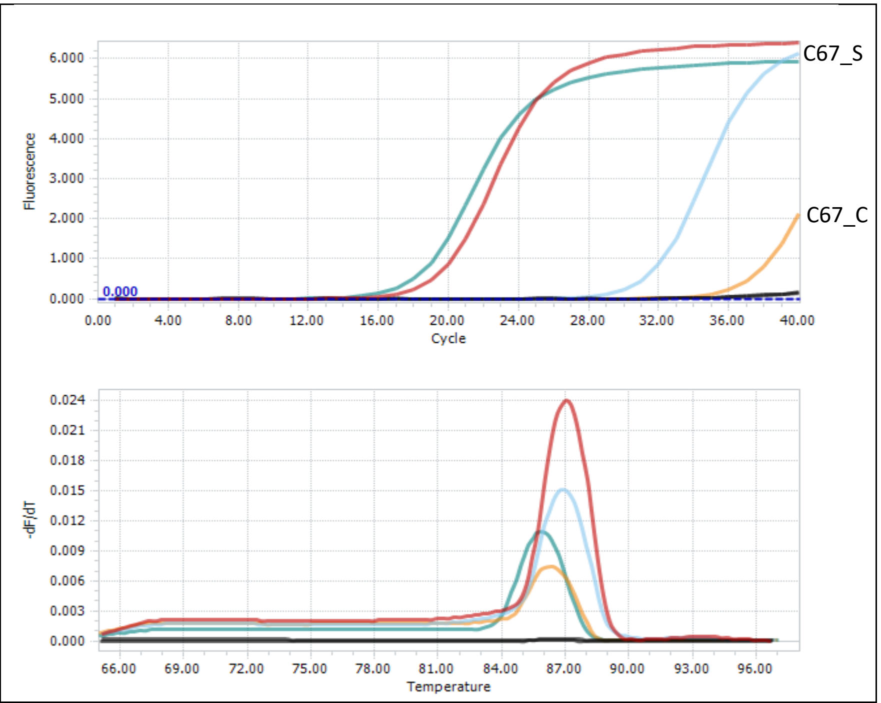
C67_S
C67_C
Fluorescence
6.000
5.000
4.000
3.000
2.000
1.000
0.000
0.000
0.00
4.00
8.00
12.00
16.00
20.00
24.00
28.00
32.00
36.00
40.00
Cycle
-dF/dT
0.024
0.021
0.018
0.015
0.012
0.009
0.006
0.003
0.000
66.00
69.00
72.00
75.00
78.00
81.00
84.00
87.00
90.00
93.00
96.00
Temperature

54	C68_D0	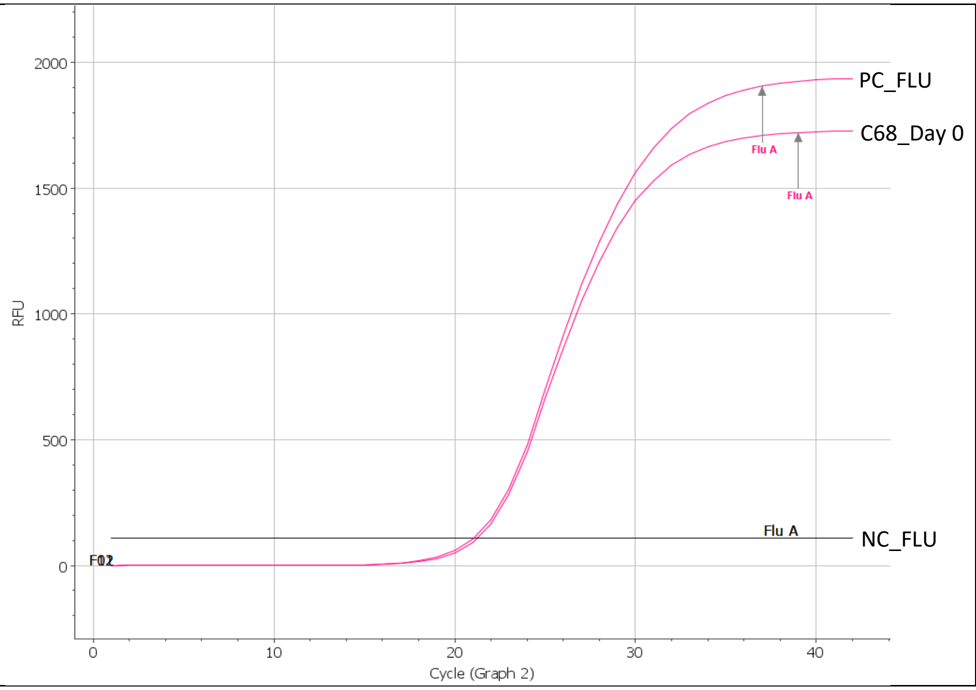 2000<br>1500<br>1000<br>500<br>0<br>RFU<br>0<br>10<br>20<br>30<br>40<br>Cycle (Graph 2)<br>PC_FLU<br>C68_Day 0<br>Flu A<br>Flu A<br>Flu A<br>NC_FLU	

C68_D2

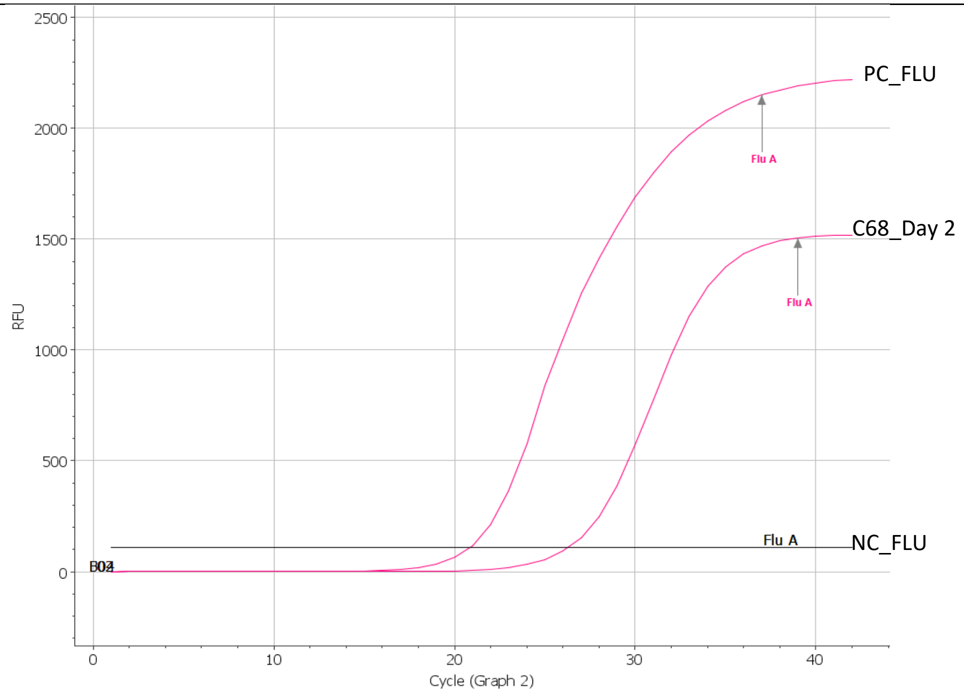
PC_FLU
C68_Day 2
NC_FLU
Flu A
RFU
Cycle (Graph 2)
2500
2000
1500
1000
500
0
0
10
20
30
40

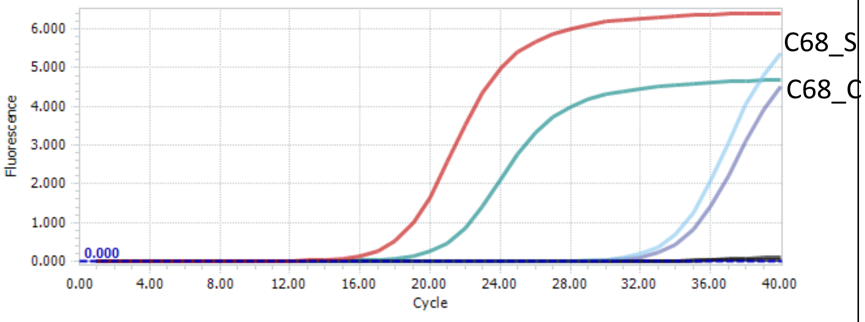
C68_S
C68_C
Fluorescence
Cycle
0.000
6.000
5.000
4.000
3.000
2.000
1.000
0.000
0.00
4.00
8.00
12.00
16.00
20.00
24.00
28.00
32.00
36.00
40.00

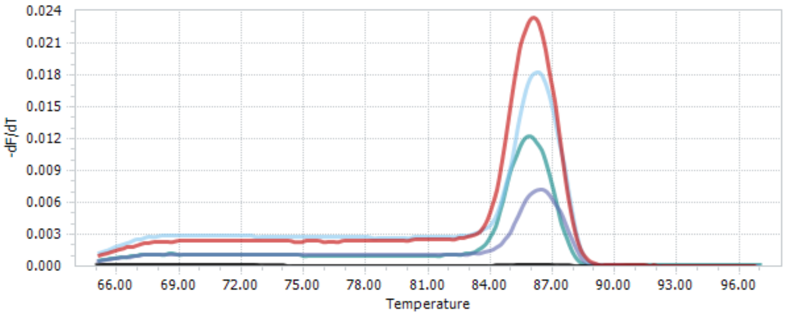
-dF/dT
Temperature
0.024
0.021
0.018
0.015
0.012
0.009
0.006
0.003
0.000
66.00
69.00
72.00
75.00
78.00
81.00
84.00
87.00
90.00
93.00
96.00

55	C70_D0	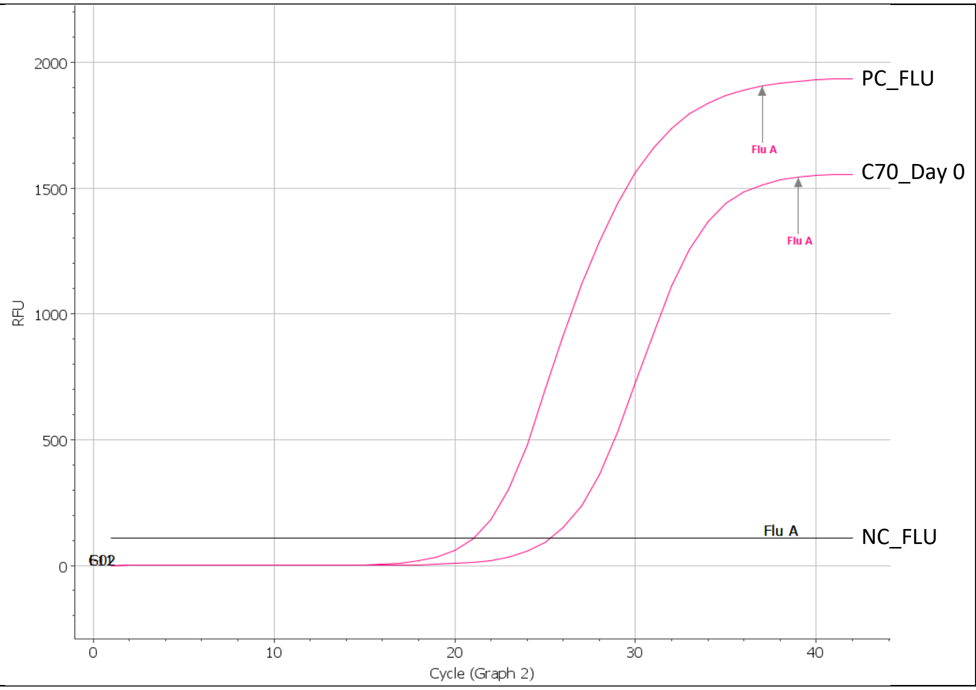2000<br>1500<br>1000<br>500<br>0<br>RFU<br>0<br>10<br>20<br>30<br>40<br>Cycle (Graph 2)<br>Flu A<br>Flu A<br>Flu A<br>PC_FLU<br>C70_Day 0<br>NC_FLU	

	C70_D2	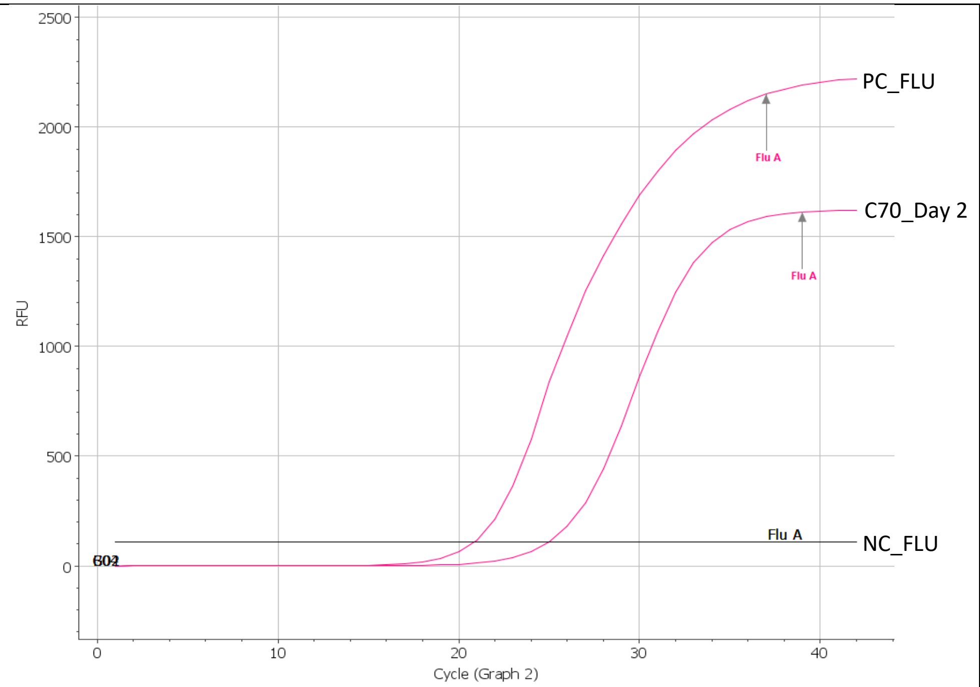 PC_FLU<br>C70_Day 2<br>NC_FLU<br>Flu A<br>Flu A<br>Flu A<br>RFU<br>Cycle (Graph 2)	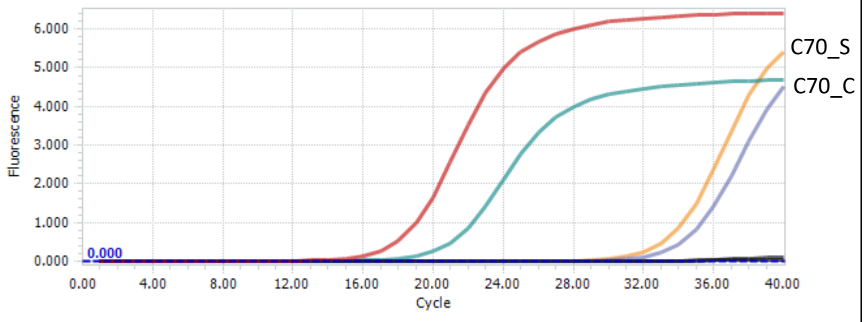 C70_S<br>C70_C<br>Fluorescence<br>Cycle 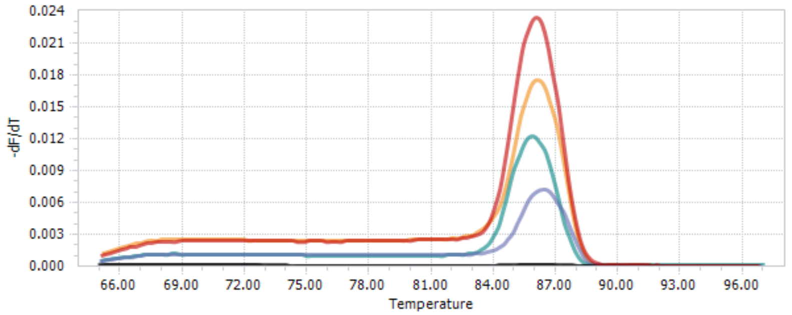 -dF/dT<br>Temperature

<table>
<tr><td>56</td><td>C71_D0</td><td>
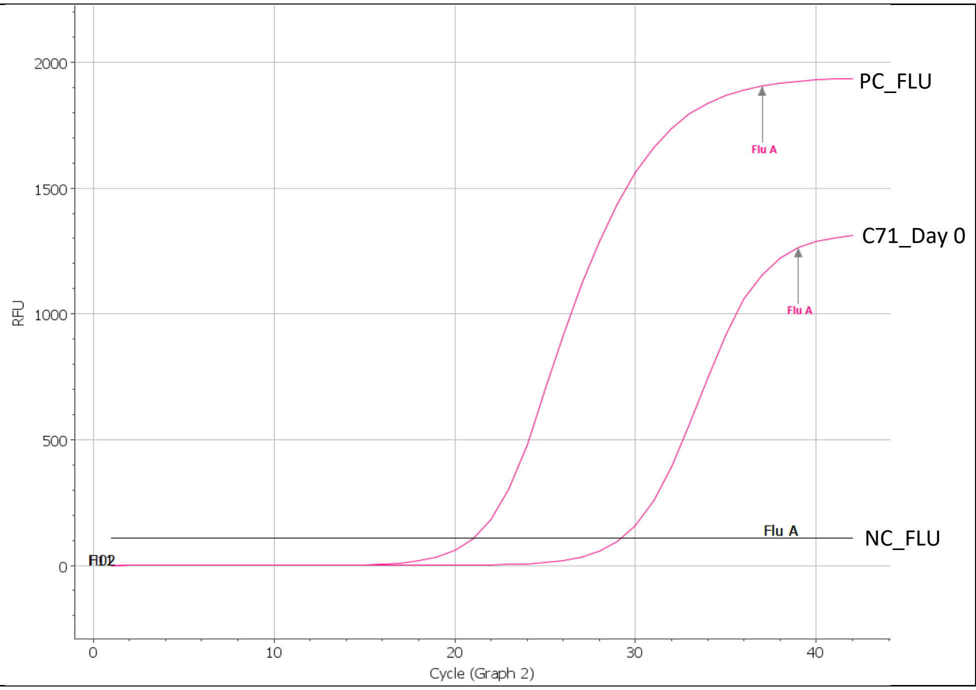

</td><td></td></tr>
</table>

	C71_D2	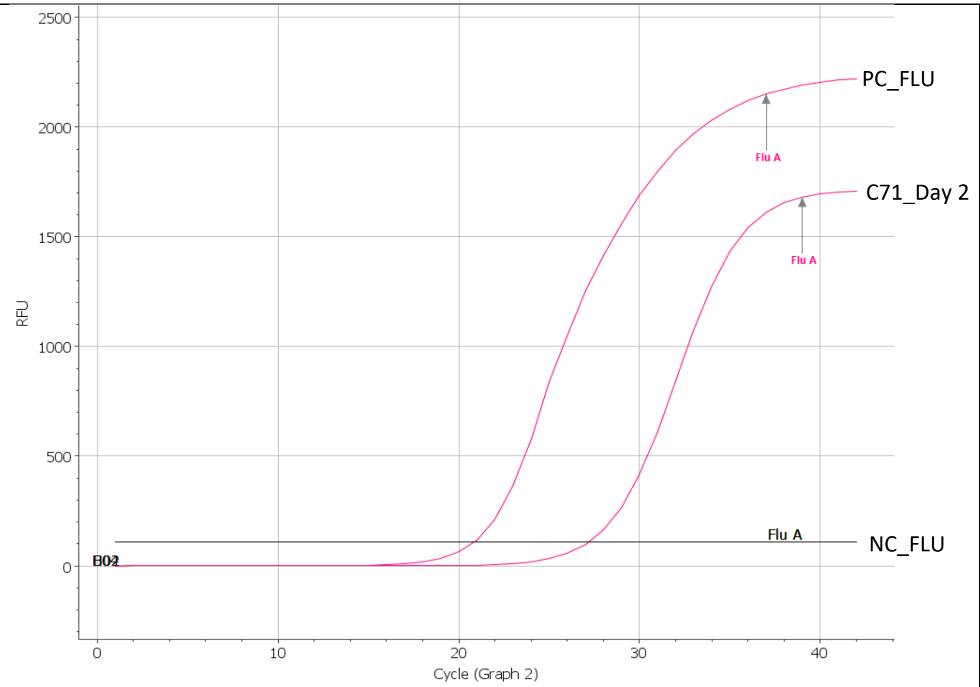 PC_FLU<br>C71_Day 2<br>NC_FLU<br>Flu A<br>Flu A<br>Flu A<br>RFU<br>Cycle (Graph 2)<br>2500<br>2000<br>1500<br>1000<br>500<br>0<br>0<br>10<br>20<br>30<br>40	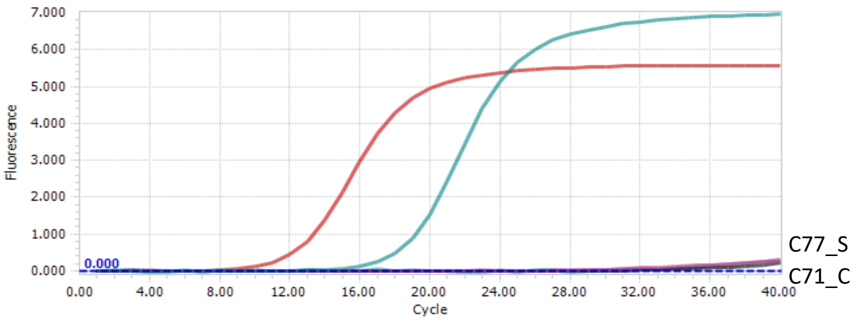 Fluorescence<br>Cycle<br>C77_S<br>C71_C<br>0.000 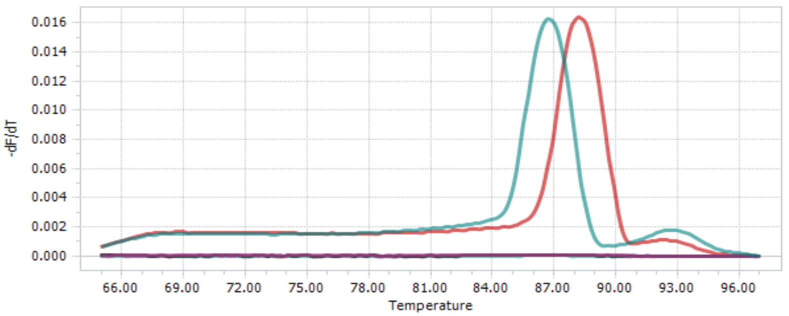 -dF/dT<br>Temperature

57	C72_D0	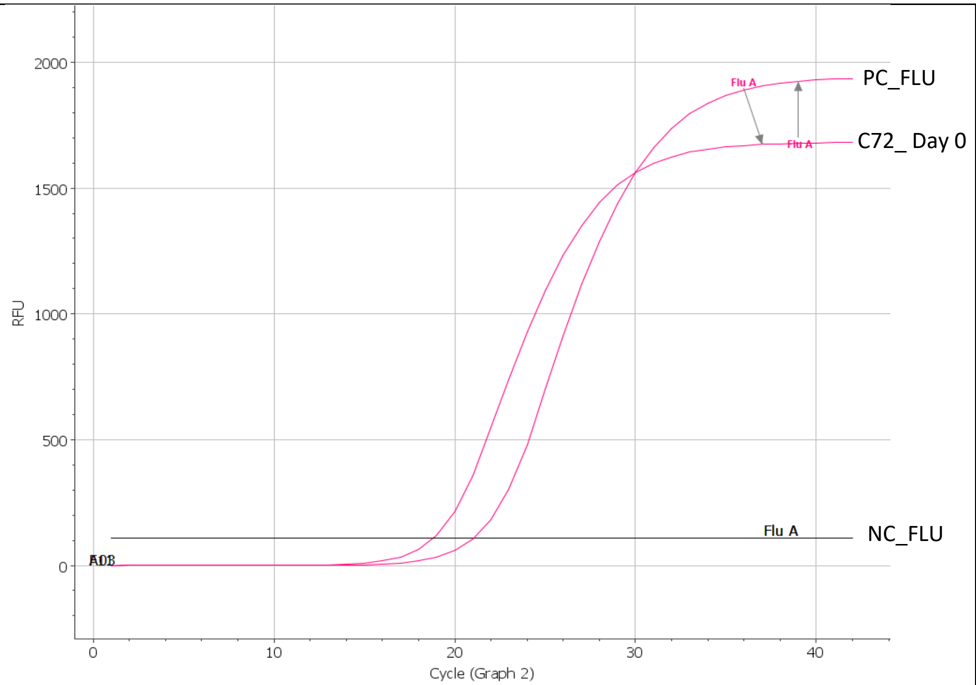	

PC_FLU
C72_ Day 0
NC_FLU
Flu A
Flu A
Flu A
RFU
2000
1500
1000
500
0
0
10
20
30
40
Cycle (Graph 2)

C72_D2

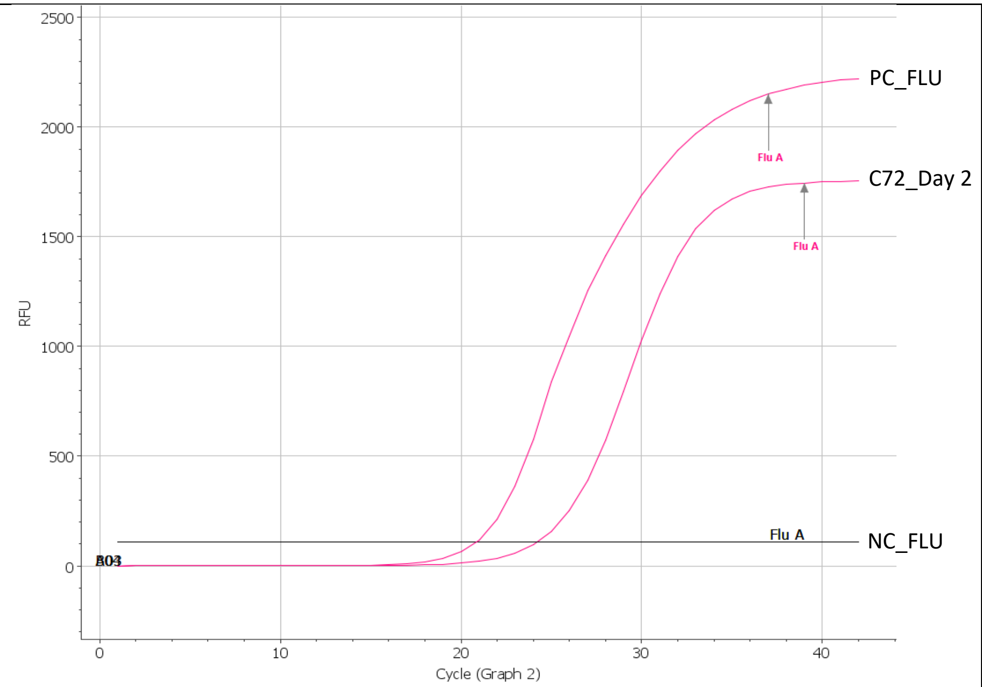

PC_FLU
C72_Day 2
NC_FLU
Flu A
RFU
2500
2000
1500
1000
500
0
10
20
30
40
Cycle (Graph 2)

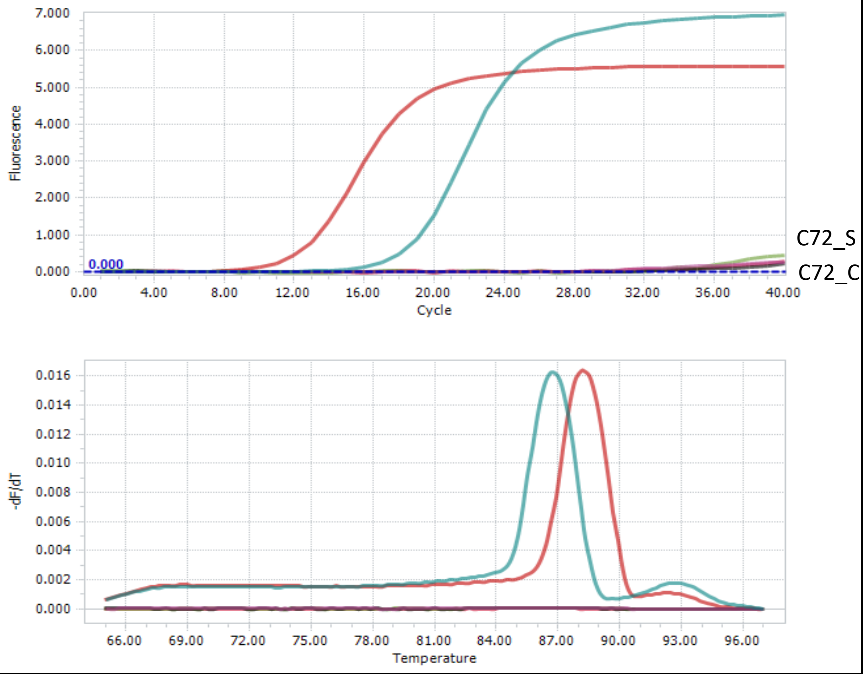

Fluorescence
7.000
6.000
5.000
4.000
3.000
2.000
1.000
0.000
0.00
4.00
8.00
12.00
16.00
20.00
24.00
28.00
32.00
36.00
40.00
Cycle
C72_S
C72_C
-dF/dT
0.016
0.014
0.012
0.010
0.008
0.006
0.004
0.002
0.000
66.00
69.00
72.00
75.00
78.00
81.00
84.00
87.00
90.00
93.00
96.00
Temperature

58	C73_D0	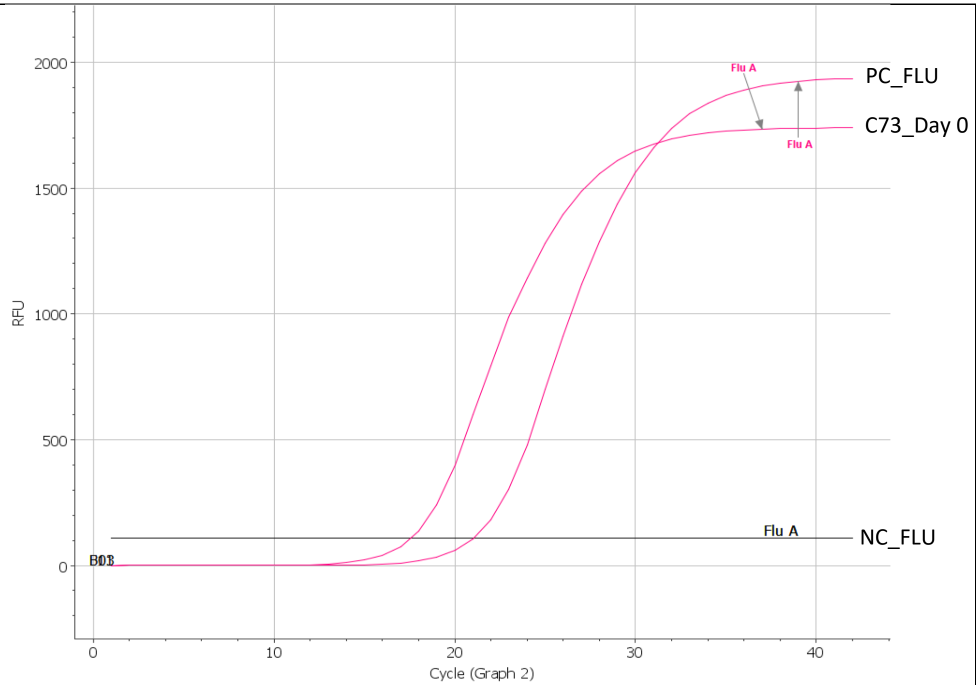 RFU<br>2000<br>1500<br>1000<br>500<br>0<br>0<br>10<br>20<br>30<br>40<br>Cycle (Graph 2)<br>Flu A<br>Flu A<br>Flu A<br>PC_FLU<br>C73_Day 0<br>NC_FLU	

C73_D2

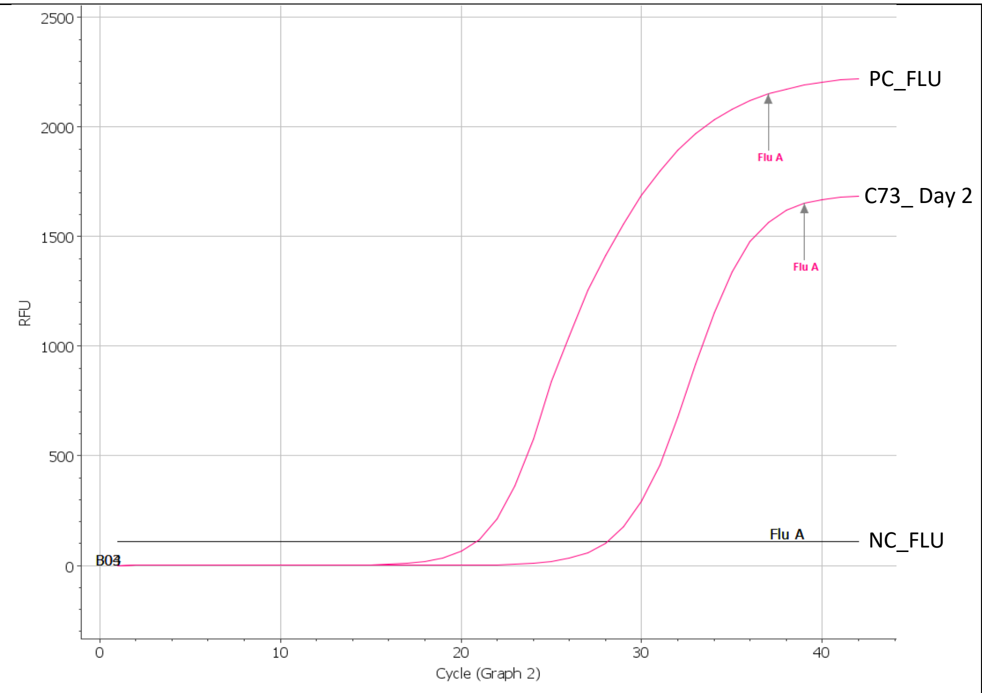
2500
2000
1500
1000
500
0
RFU
0
10
20
30
40
Cycle (Graph 2)
PC_FLU
C73_ Day 2
NC_FLU
Flu A
Flu A
Flu A
B03

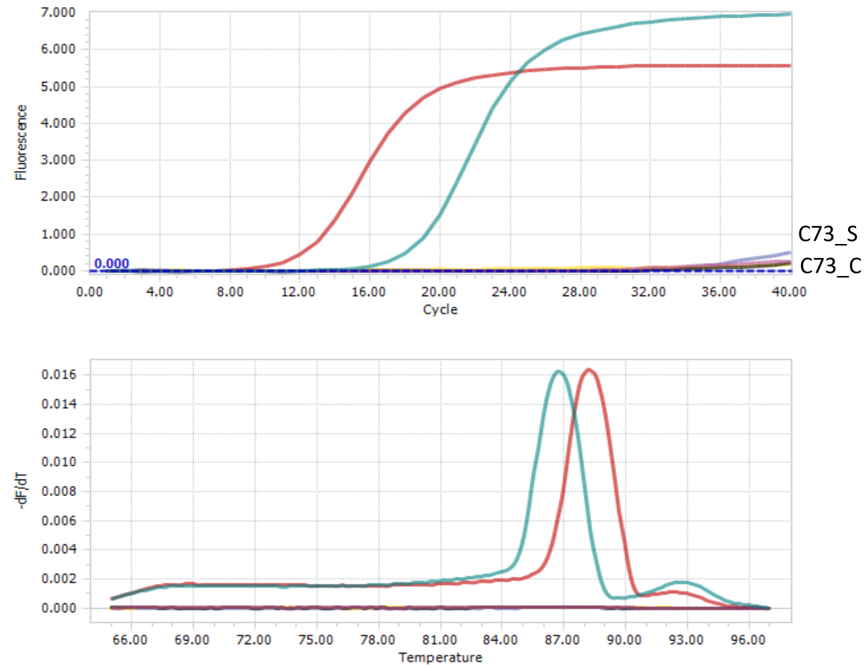
7.000
6.000
5.000
4.000
3.000
2.000
1.000
0.000
Fluorescence
0.000
0.00
4.00
8.00
12.00
16.00
20.00
24.00
28.00
32.00
36.00
40.00
Cycle
C73_S
C73_C
0.016
0.014
0.012
0.010
0.008
0.006
0.004
0.002
0.000
-dF/dT
66.00
69.00
72.00
75.00
78.00
81.00
84.00
87.00
90.00
93.00
96.00
Temperature

59	C74_D0	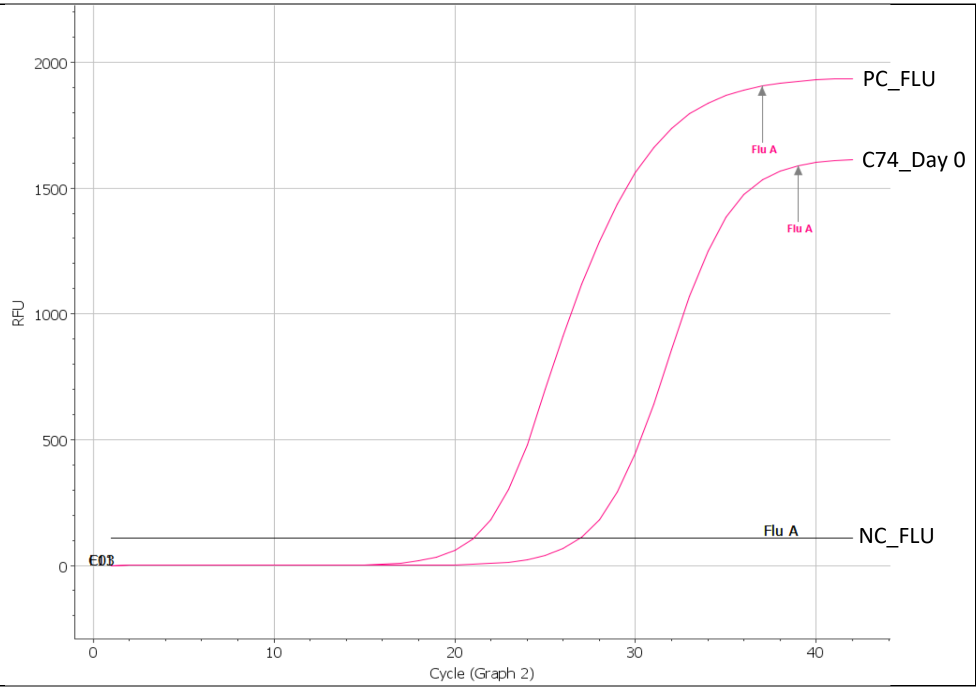PC_FLU<br>C74_Day 0<br>NC_FLU<br>Flu A<br>Flu A<br>Flu A<br>RFU<br>2000<br>1500<br>1000<br>500<br>0<br>0<br>10<br>20<br>30<br>40<br>Cycle (Graph 2)	

	C74_D2	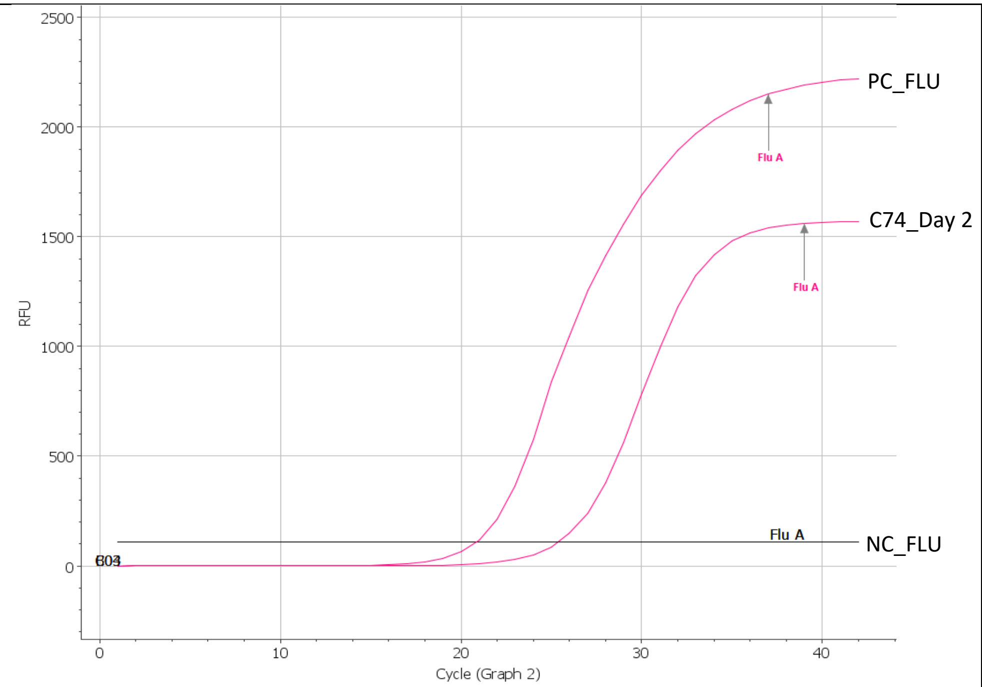	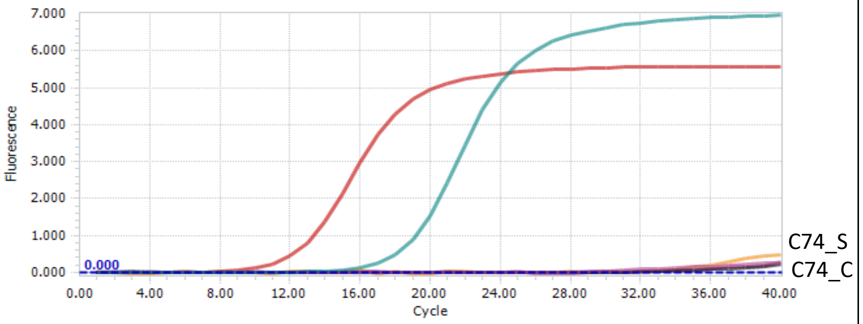 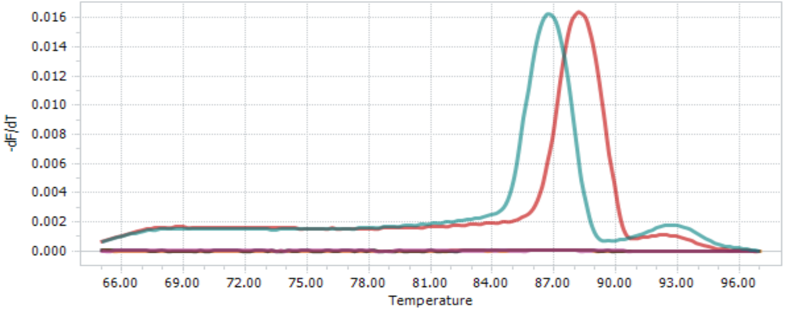

2500
2000
1500
1000
500
0
RFU
0
10
20
30
40
Cycle (Graph 2)
PC_FLU
Flu A
C74_Day 2
Flu A
Flu A
NC_FLU

7.000
6.000
5.000
4.000
3.000
2.000
1.000
0.000
Fluorescence
0.000
0.00
4.00
8.00
12.00
16.00
20.00
24.00
28.00
32.00
36.00
40.00
Cycle
C74_S
C74_C

0.016
0.014
0.012
0.010
0.008
0.006
0.004
0.002
0.000
-dF/dT
66.00
69.00
72.00
75.00
78.00
81.00
84.00
87.00
90.00
93.00
96.00
Temperature

60	C75_D0	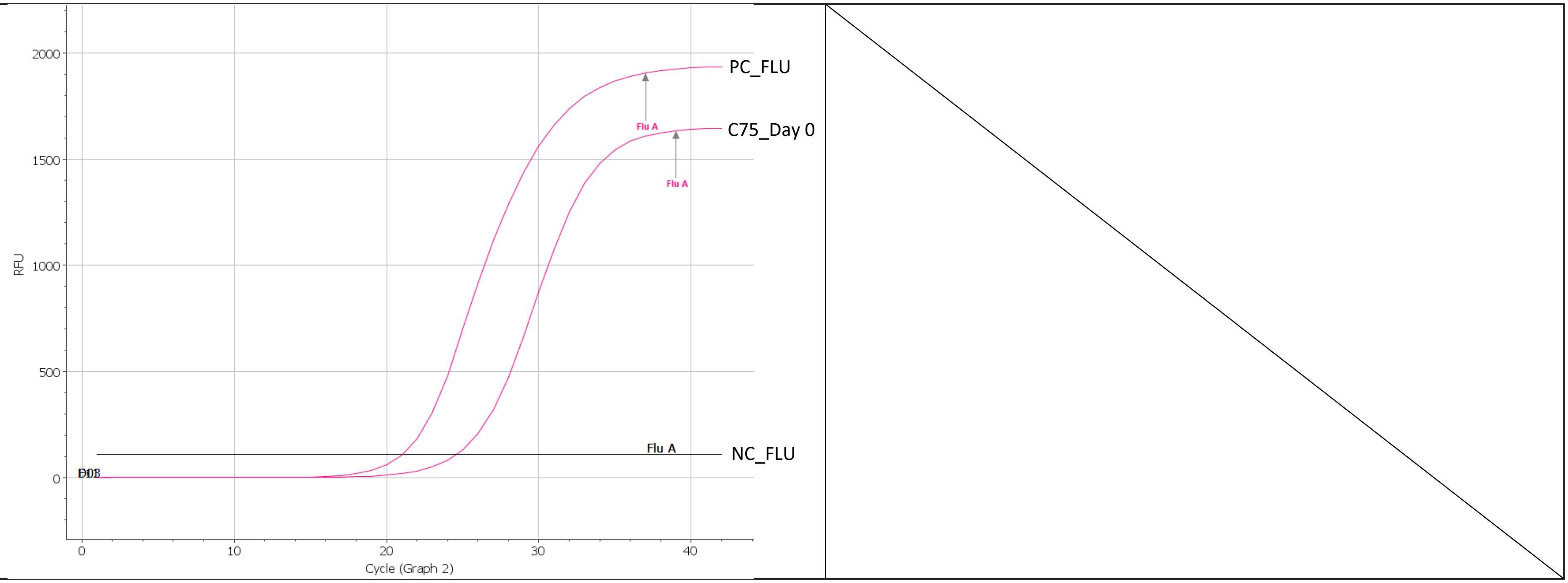2000<br>1500<br>1000<br>500<br>0<br>RFU<br>0<br>10<br>20<br>30<br>40<br>Cycle (Graph 2)<br>Flu A<br>Flu A<br>Flu A<br>PC_FLU<br>C75_Day 0<br>NC_FLU	

	C75_D2	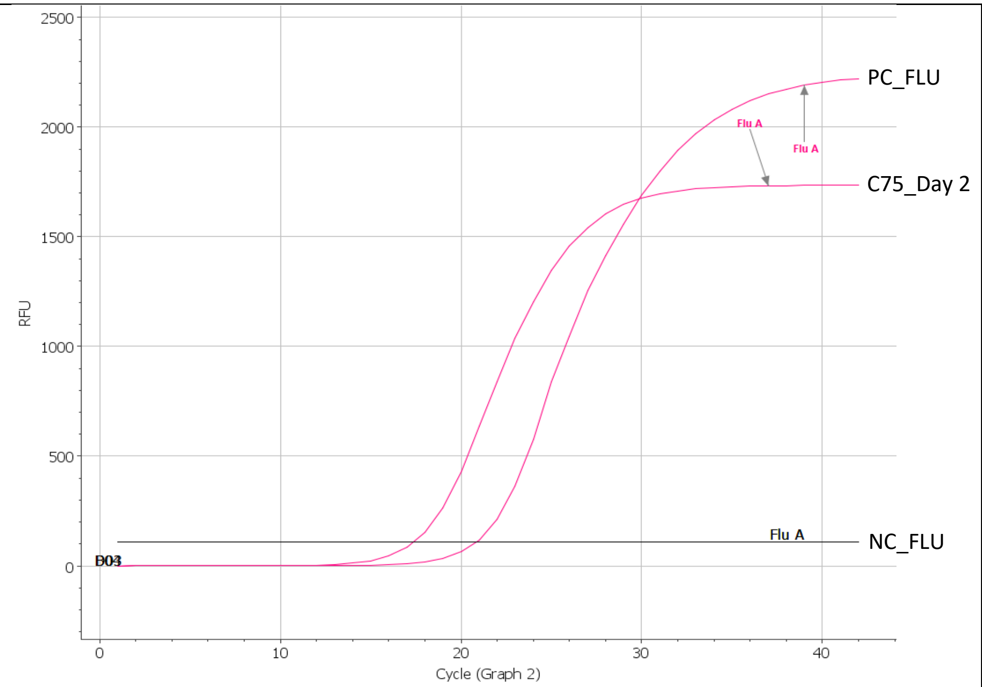	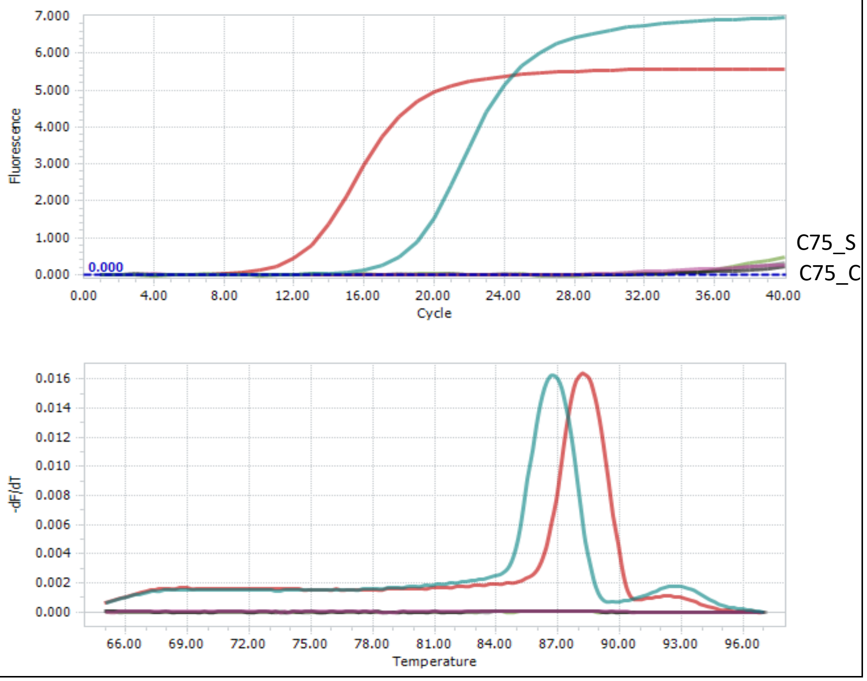

PC_FLU
C75_Day 2
NC_FLU
Flu A
Flu A
Flu A
RFU
Cycle (Graph 2)

Fluorescence
Cycle
0.000
C75_S
C75_C
-dF/dT
Temperature

61	C76_D0	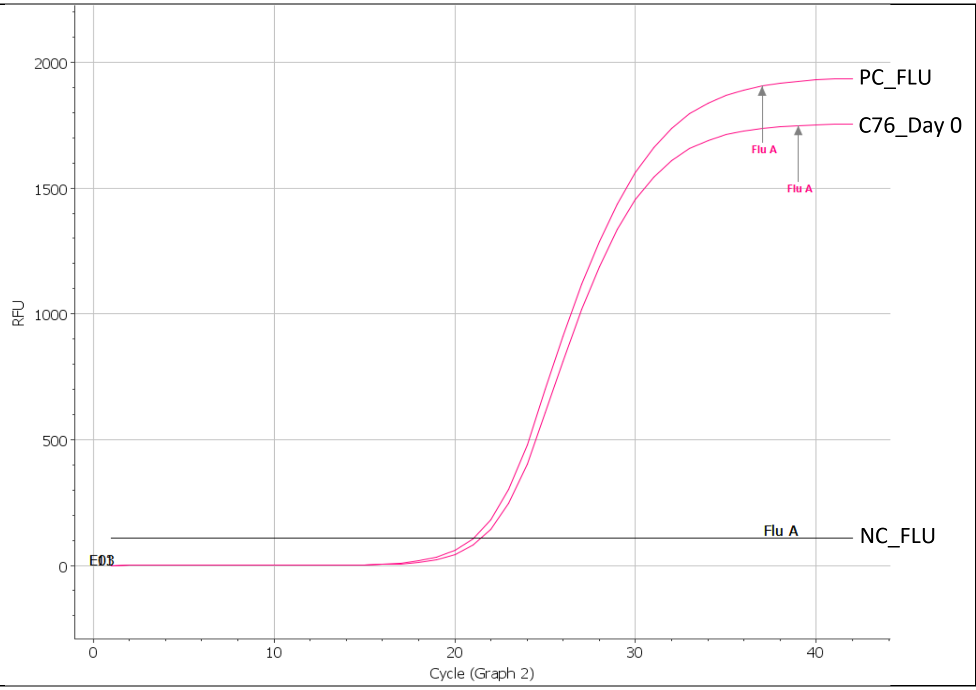2000<br>1500<br>1000<br>500<br>0<br>RFU<br>0<br>10<br>20<br>30<br>40<br>Cycle (Graph 2)<br>PC_FLU<br>C76_Day 0<br>Flu A<br>Flu A<br>Flu A<br>NC_FLU	

	C76_D2	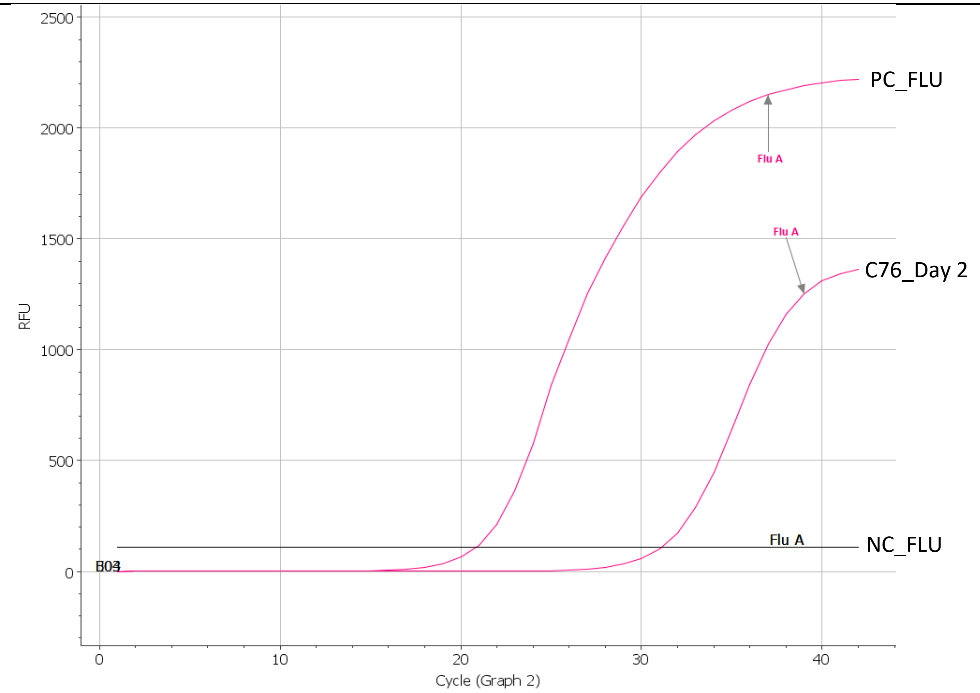	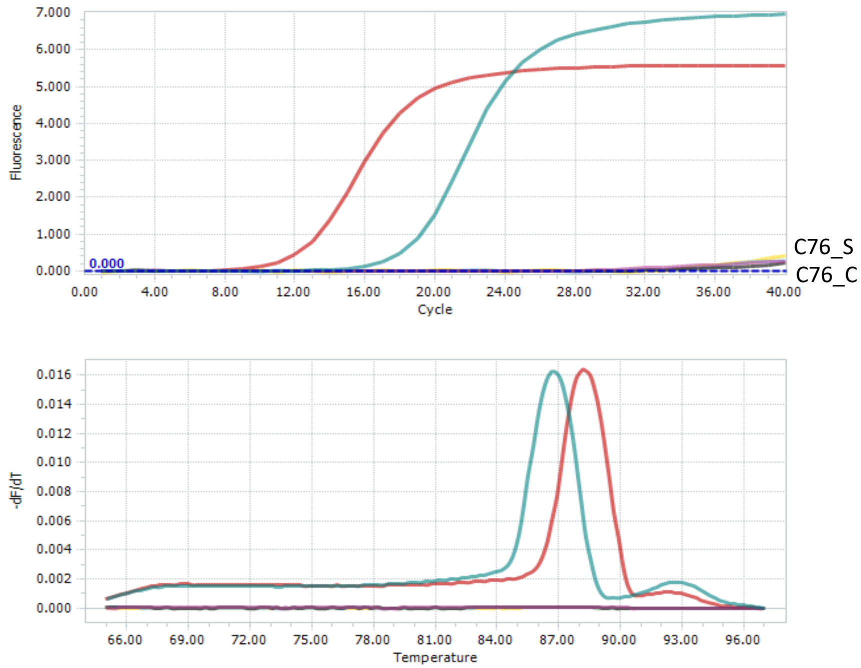

RFU
Cycle (Graph 2)
PC_FLU
C76_Day 2
NC_FLU
Flu A

Fluorescence
Cycle
C76_S
C76_C
-dF/dT
Temperature

62	C77_D0	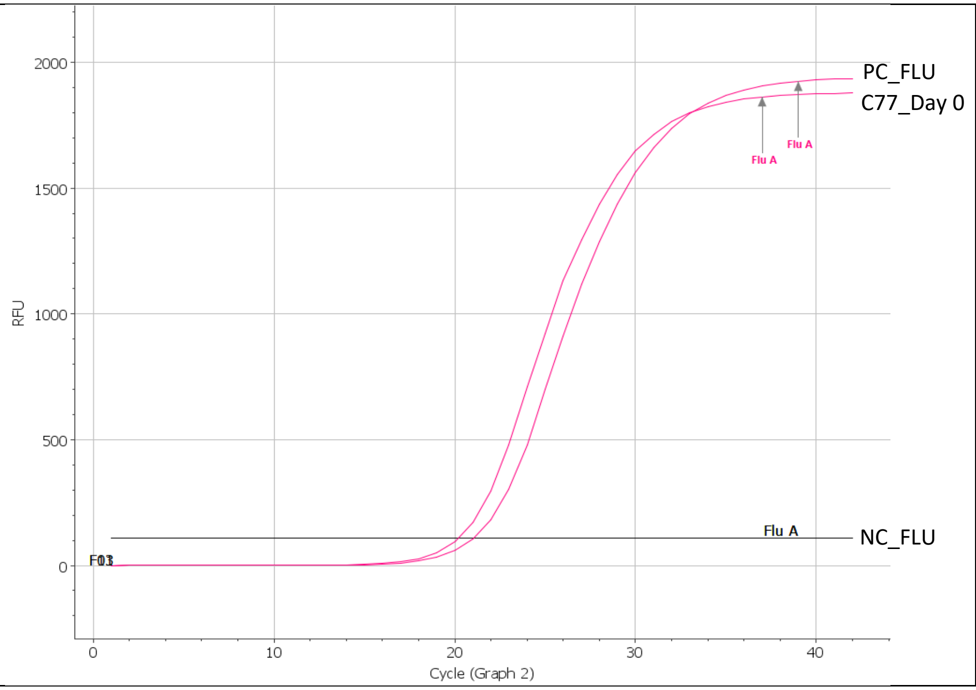2000
1500
1000
500
0
RFU
0
10
20
30
40
Cycle (Graph 2)
PC_FLU
C77_Day 0
Flu A
Flu A
Flu A
NC_FLU
F03	

	C77_D2	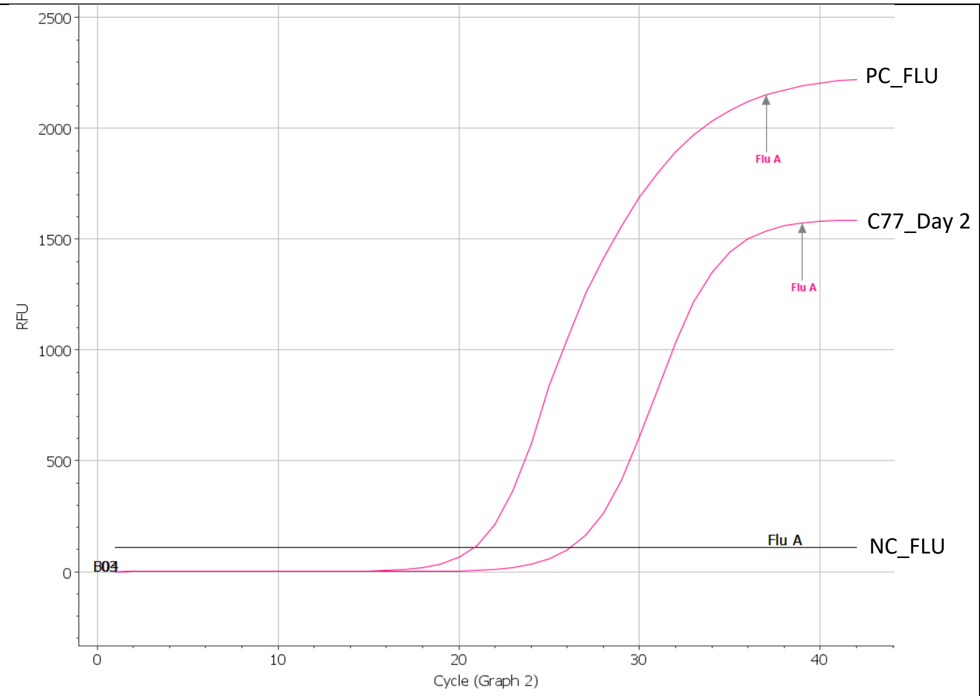 PC_FLU<br>C77_Day 2<br>NC_FLU<br>Flu A<br>RFU<br>Cycle (Graph 2)	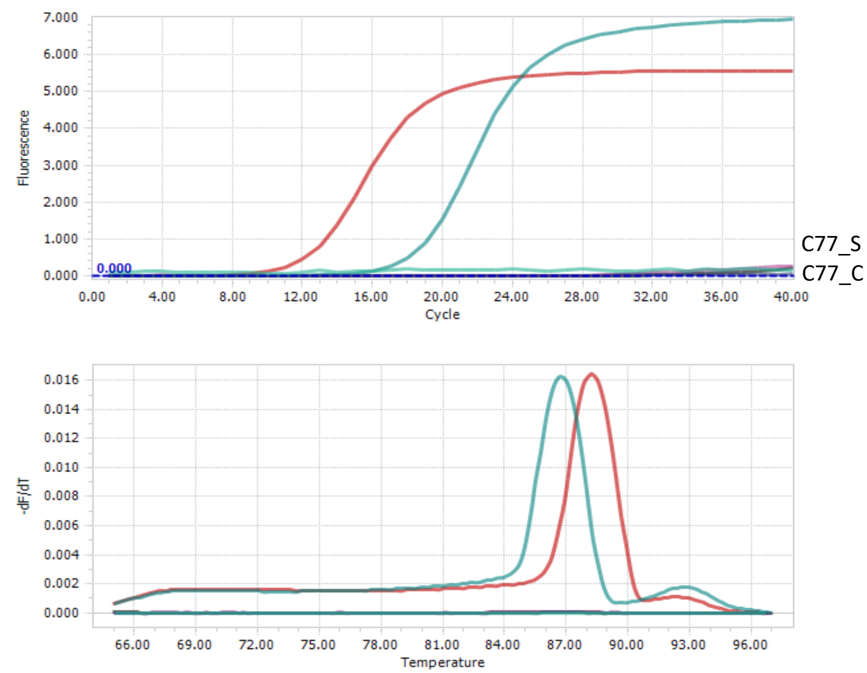 Fluorescence<br>Cycle<br>C77_S<br>C77_C<br>-dF/dT<br>Temperature

63	C78_D0	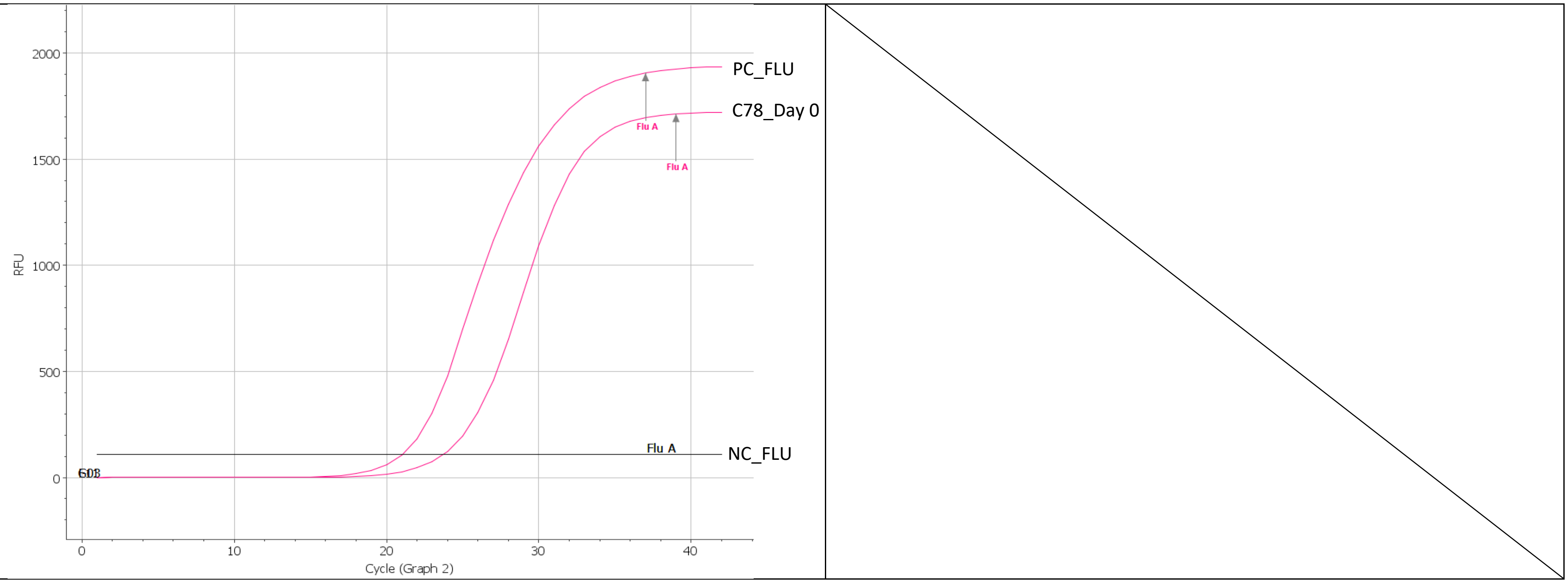	

RFU
2000
1500
1000
500
0
Cycle (Graph 2)
0
10
20
30
40
PC_FLU
C78_Day 0
NC_FLU
Flu A
Flu A
Flu A

C78_D2

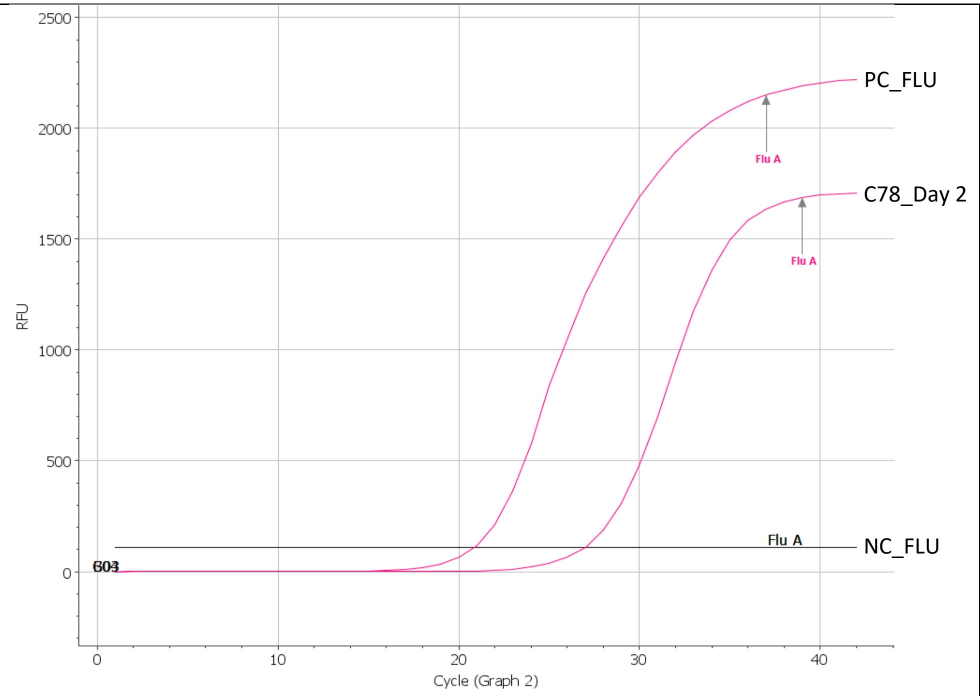

2500
2000
1500
1000
500
0
RFU
PC_FLU
C78_Day 2
NC_FLU
Flu A
Flu A
Flu A
603
0
10
20
30
40
Cycle (Graph 2)

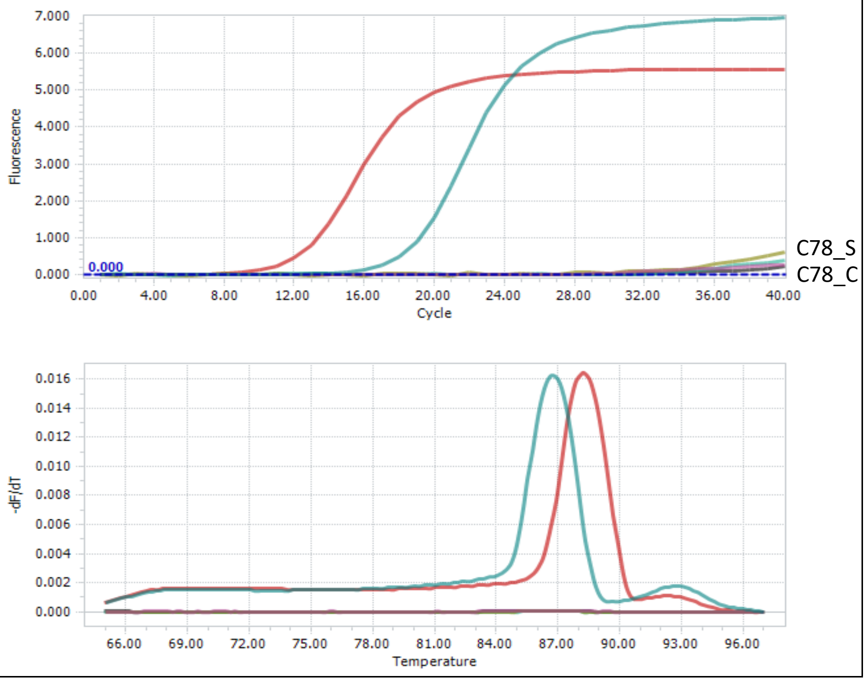

Fluorescence
7.000
6.000
5.000
4.000
3.000
2.000
1.000
0.000
0.000
0.00
4.00
8.00
12.00
16.00
20.00
24.00
28.00
32.00
36.00
40.00
Cycle
C78_S
C78_C
-dF/dT
0.016
0.014
0.012
0.010
0.008
0.006
0.004
0.002
0.000
66.00
69.00
72.00
75.00
78.00
81.00
84.00
87.00
90.00
93.00
96.00
Temperature

64	C79_D0	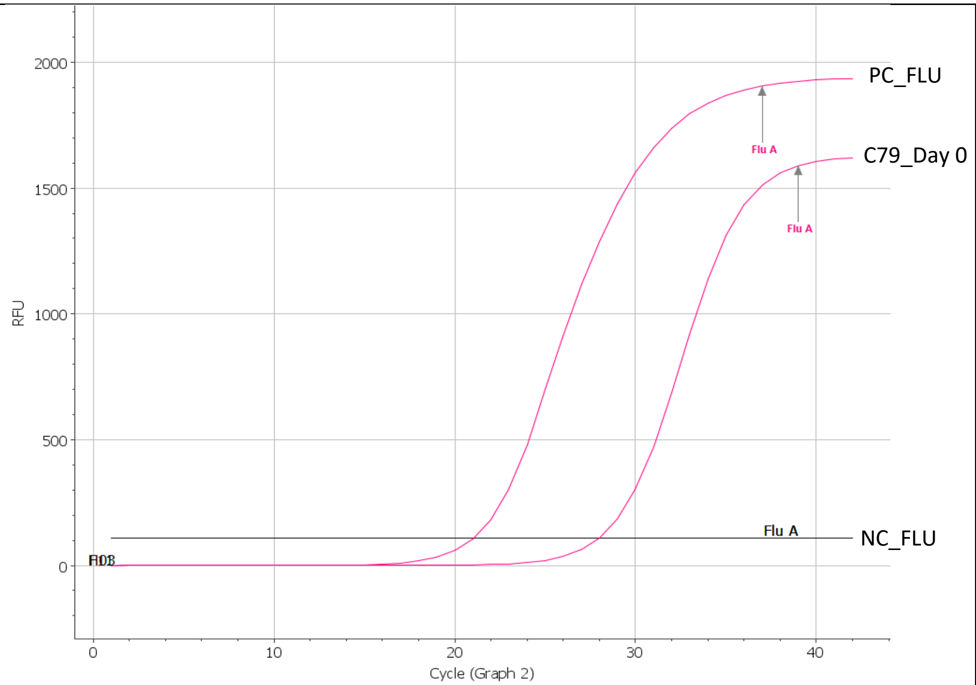	

2000
1500
1000
500
0
-500
RFU
0
10
20
30
40
Cycle (Graph 2)
PC_FLU
C79_Day 0
NC_FLU
Flu A
Flu A
Flu A

	C79_D2	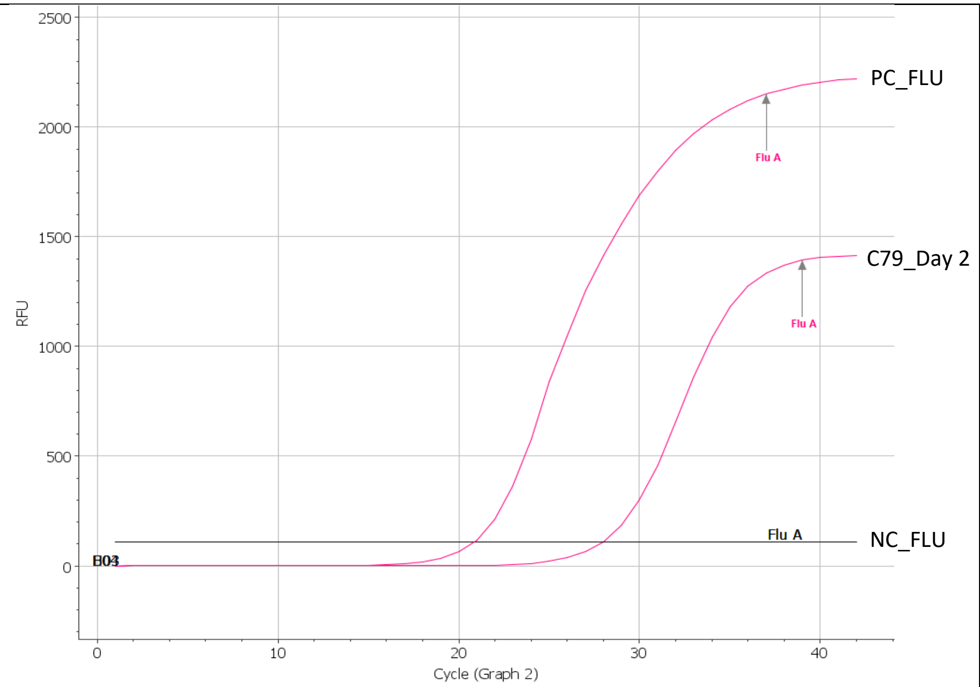 PC_FLU<br>C79_Day 2<br>NC_FLU<br>Flu A<br>RFU<br>Cycle (Graph 2) 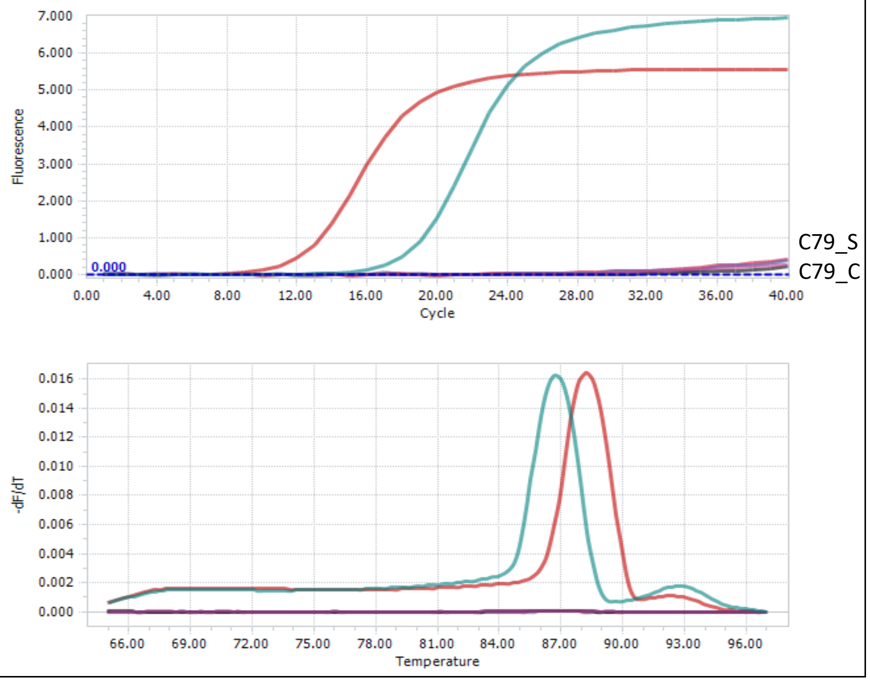 Fluorescence<br>Cycle<br>C79_S<br>C79_C<br>-dF/dT<br>Temperature

65	C81_D0	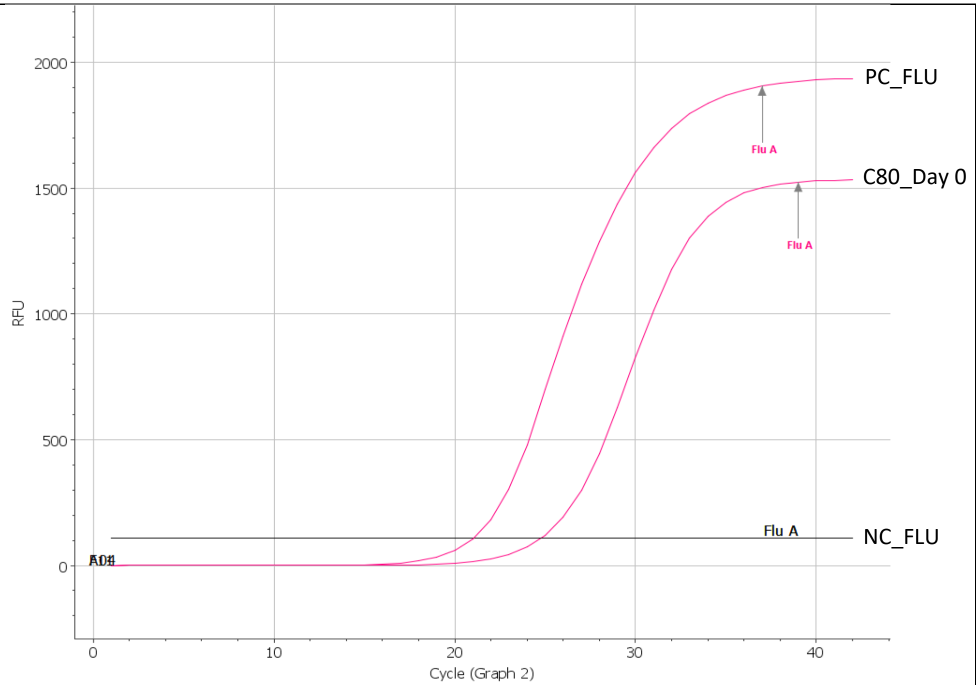	

RFU
2000
1500
1000
500
0
0
10
20
30
40
Cycle (Graph 2)
Flu A
Flu A
Flu A
PC_FLU
C80_Day 0
NC_FLU

	C81 _D2	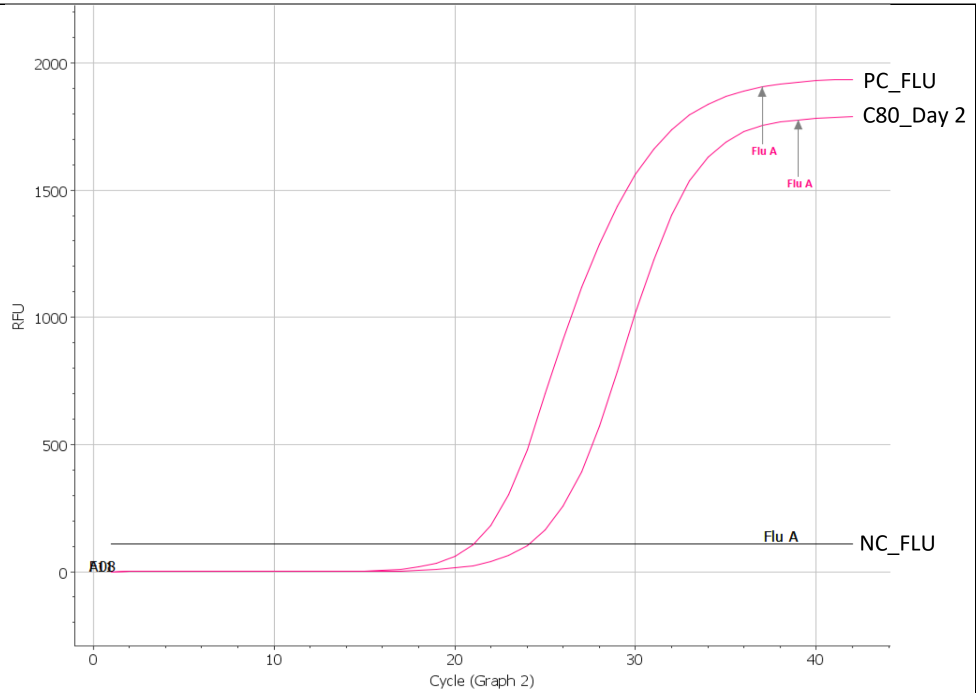	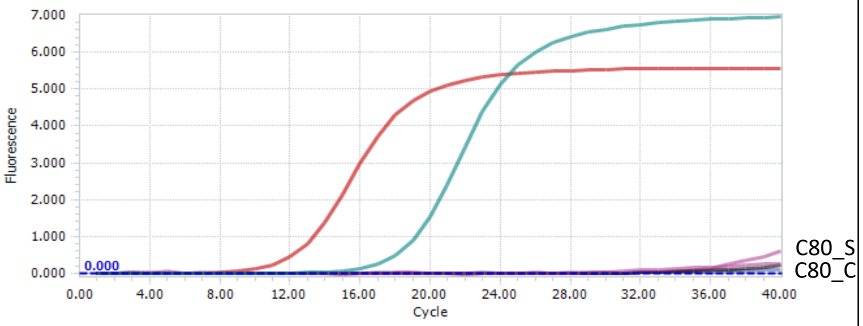 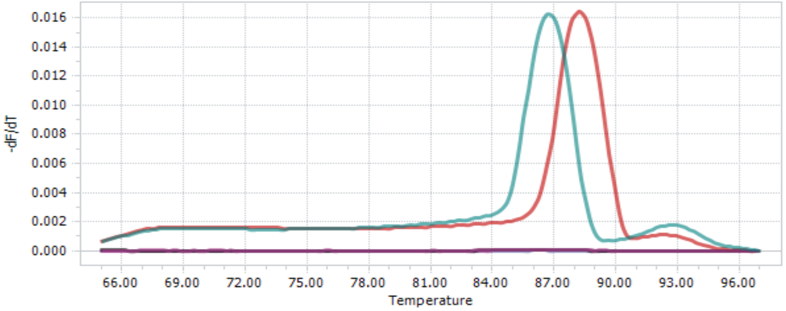

RFU
Cycle (Graph 2)
2000
1500
1000
500
0
0
10
20
30
40
Flu A
Flu A
Flu A
PC_FLU
C80_Day 2
NC_FLU

Fluorescence
Cycle
7.000
6.000
5.000
4.000
3.000
2.000
1.000
0.000
0.00
4.00
8.00
12.00
16.00
20.00
24.00
28.00
32.00
36.00
40.00
C80_S
C80_C

-dF/dT
Temperature
0.016
0.014
0.012
0.010
0.008
0.006
0.004
0.002
0.000
66.00
69.00
72.00
75.00
78.00
81.00
84.00
87.00
90.00
93.00
96.00

66	C82_D0	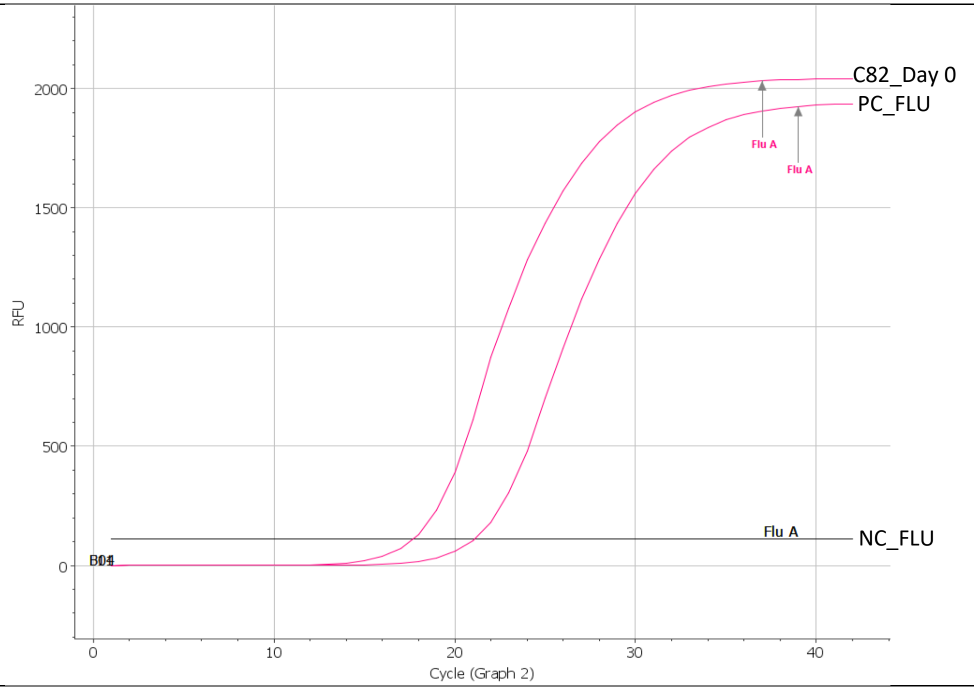2000<br>1500<br>1000<br>500<br>0<br>RFU<br>0<br>10<br>20<br>30<br>40<br>Cycle (Graph 2)<br>C82_Day 0<br>PC_FLU<br>Flu A<br>Flu A<br>Flu A<br>NC_FLU	

C82_D2

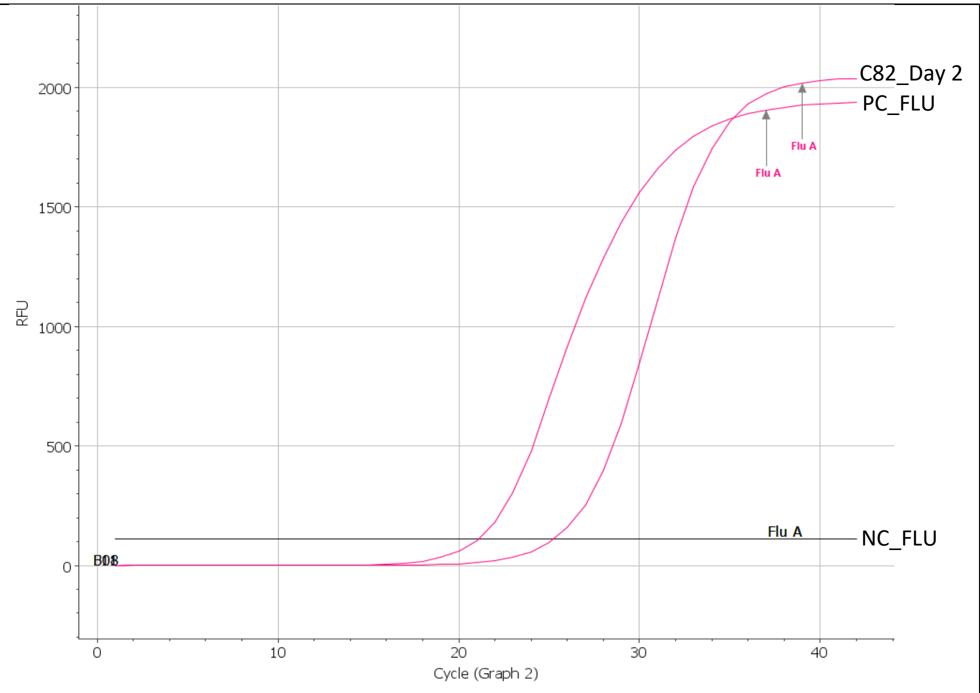
C82_Day 2
PC_FLU
NC_FLU
Flu A
Flu A
Flu A
RFU
2000
1500
1000
500
0
0
10
20
30
40
Cycle (Graph 2)

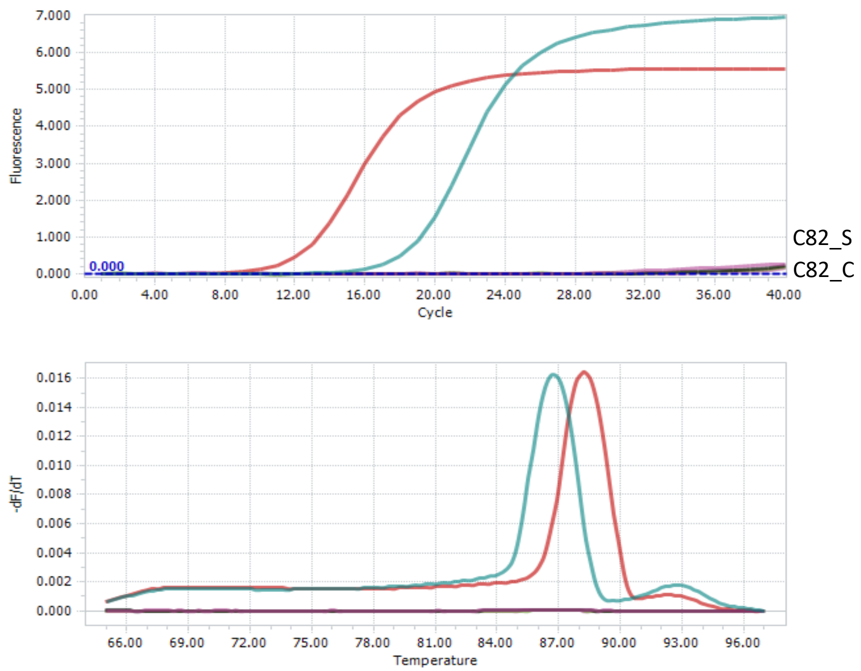
Fluorescence
7.000
6.000
5.000
4.000
3.000
2.000
1.000
0.000
0.000
0.00
4.00
8.00
12.00
16.00
20.00
24.00
28.00
32.00
36.00
40.00
Cycle
C82_S
C82_C
-dF/dT
0.016
0.014
0.012
0.010
0.008
0.006
0.004
0.002
0.000
66.00
69.00
72.00
75.00
78.00
81.00
84.00
87.00
90.00
93.00
96.00
Temperature

67	C83_D0	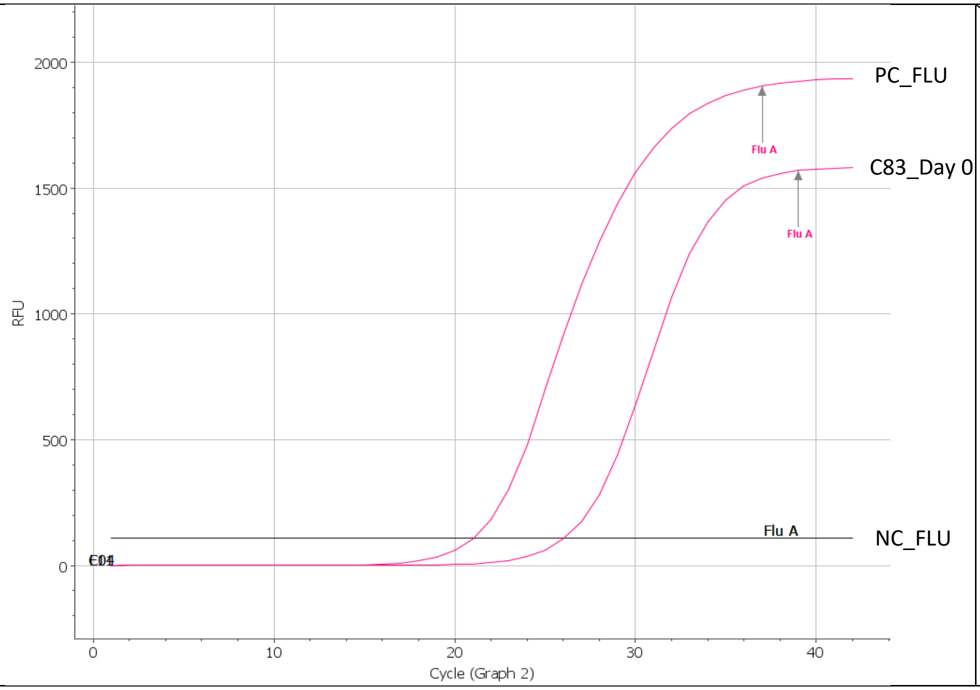	

PC_FLU
C83_Day 0
NC_FLU
Flu A
Flu A
Flu A
RFU
2000
1500
1000
500
0
0
10
20
30
40
Cycle (Graph 2)

	C83_D2	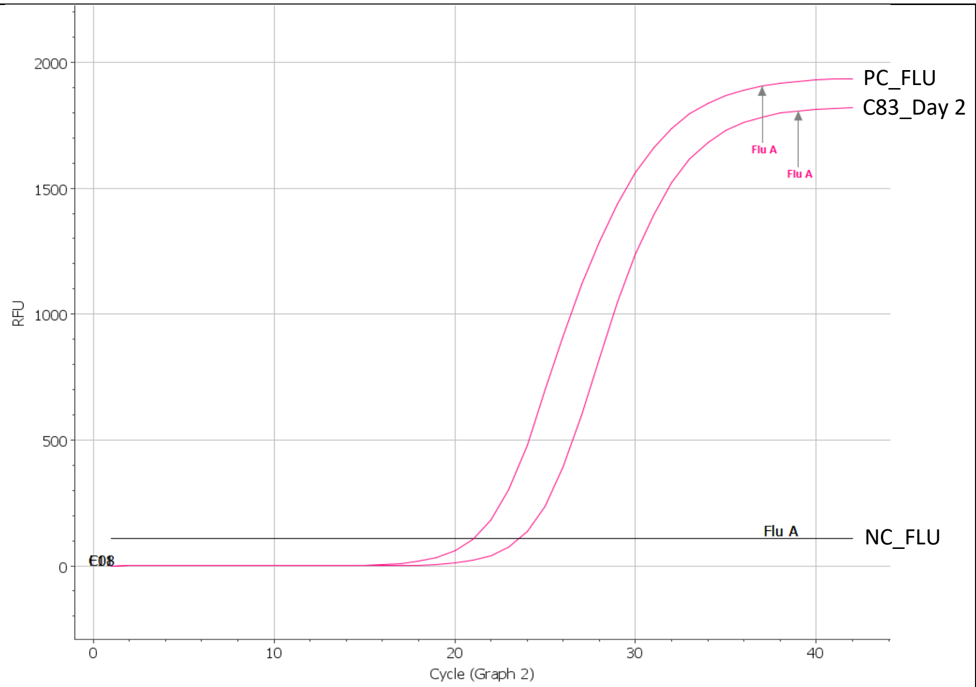PC_FLU<br>C83_Day 2<br>NC_FLU<br>Flu A<br>RFU<br>Cycle (Graph 2)	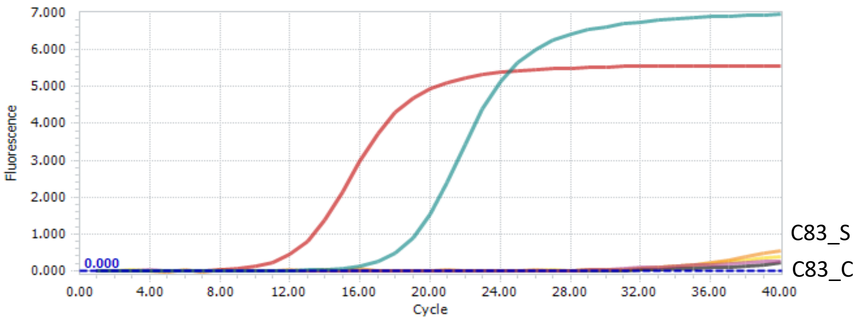Fluorescence<br>Cycle<br>C83_S<br>C83_C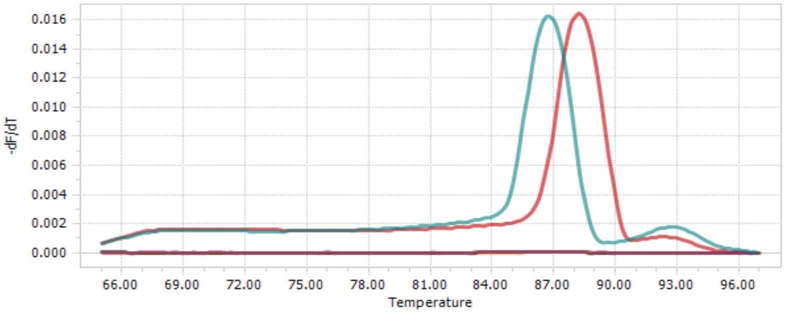-dF/dT<br>Temperature

68	C85_D0	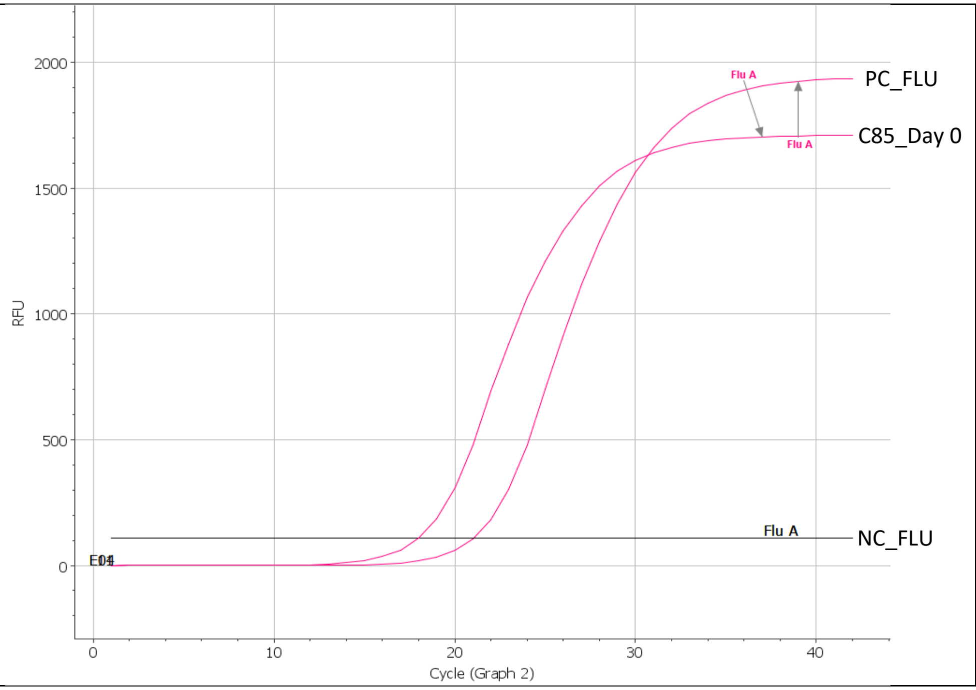PC_FLU<br>C85_Day 0<br>NC_FLU<br>Flu A<br>Flu A<br>Flu A<br>E04<br>RFU<br>Cycle (Graph 2)<br>2000<br>1500<br>1000<br>500<br>0<br>0<br>10<br>20<br>30<br>40	

	C85_D2	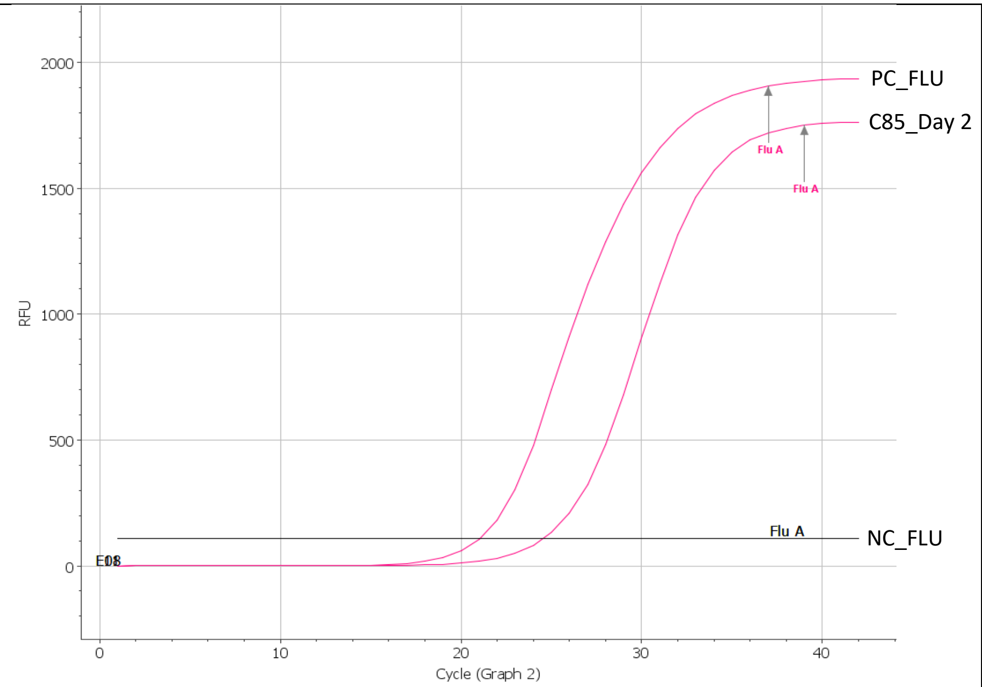	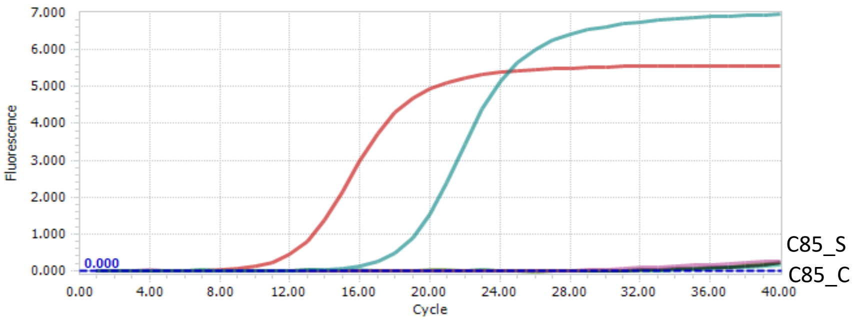 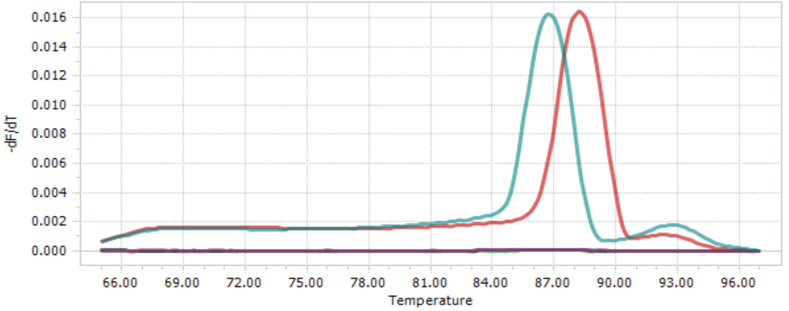

RFU
2000
1500
1000
500
0
0
10
20
30
40
Cycle (Graph 2)
PC_FLU
C85_Day 2
NC_FLU
Flu A
Flu A
Flu A

Fluorescence
7.000
6.000
5.000
4.000
3.000
2.000
1.000
0.000
0.000
0.00
4.00
8.00
12.00
16.00
20.00
24.00
28.00
32.00
36.00
40.00
Cycle
C85_S
C85_C

-dF/dT
0.016
0.014
0.012
0.010
0.008
0.006
0.004
0.002
0.000
66.00
69.00
72.00
75.00
78.00
81.00
84.00
87.00
90.00
93.00
96.00
Temperature

69	C86_D0	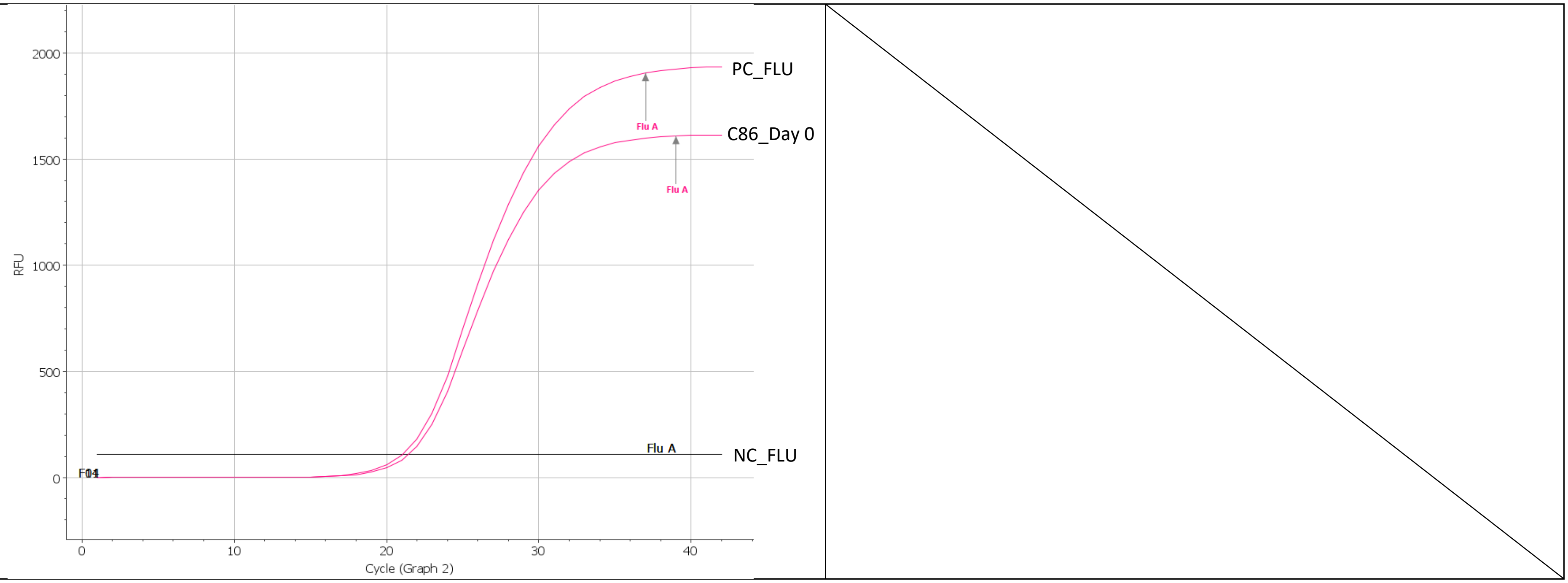	

2000
1500
1000
500
0
RFU
0
10
20
30
40
Cycle (Graph 2)
PC_FLU
C86_Day 0
NC_FLU
Flu A
Flu A
Flu A
F04

	C86 _D2	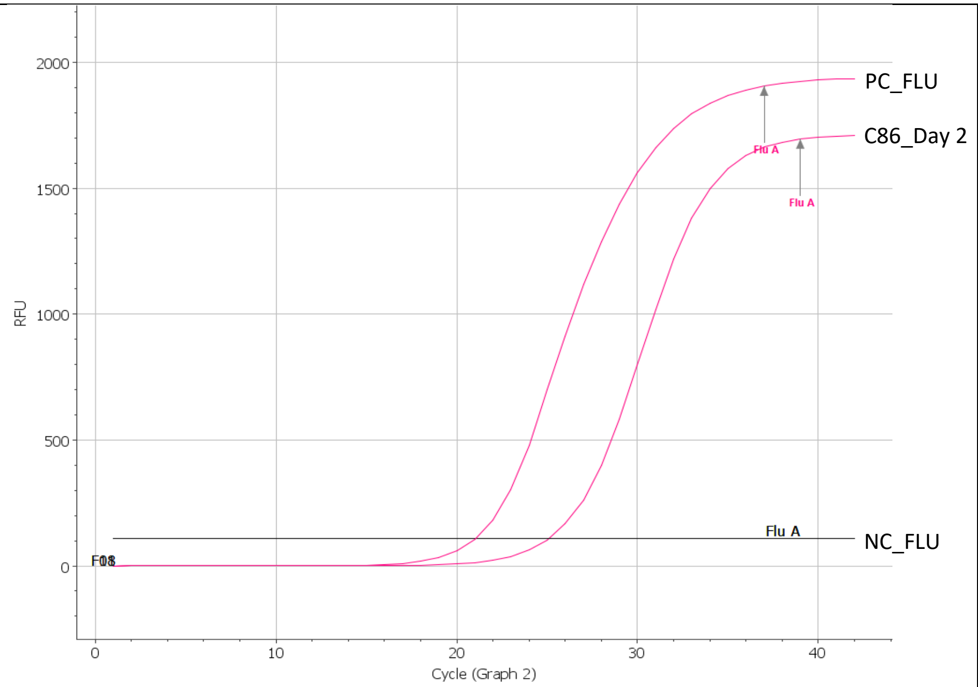PC_FLU<br>C86_Day 2<br>NC_FLU<br>Flu A<br>RFU<br>Cycle (Graph 2)	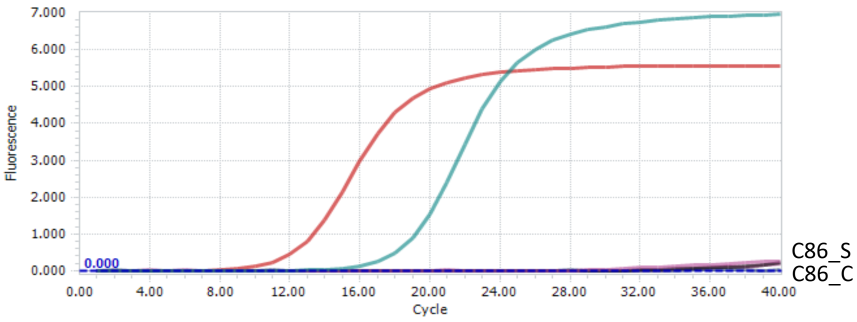Fluorescence<br>Cycle<br>C86_S<br>C86_C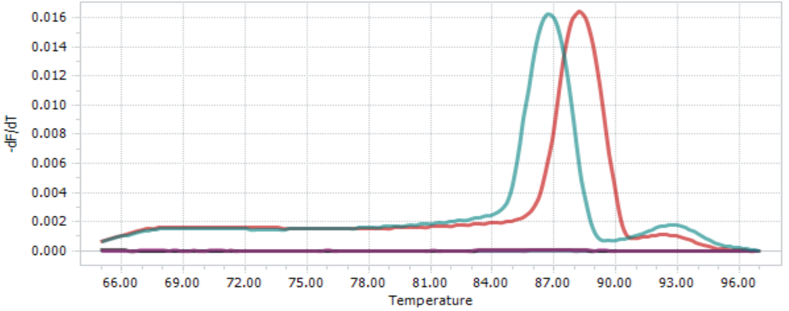-dF/dT<br>Temperature

70	C87_D0	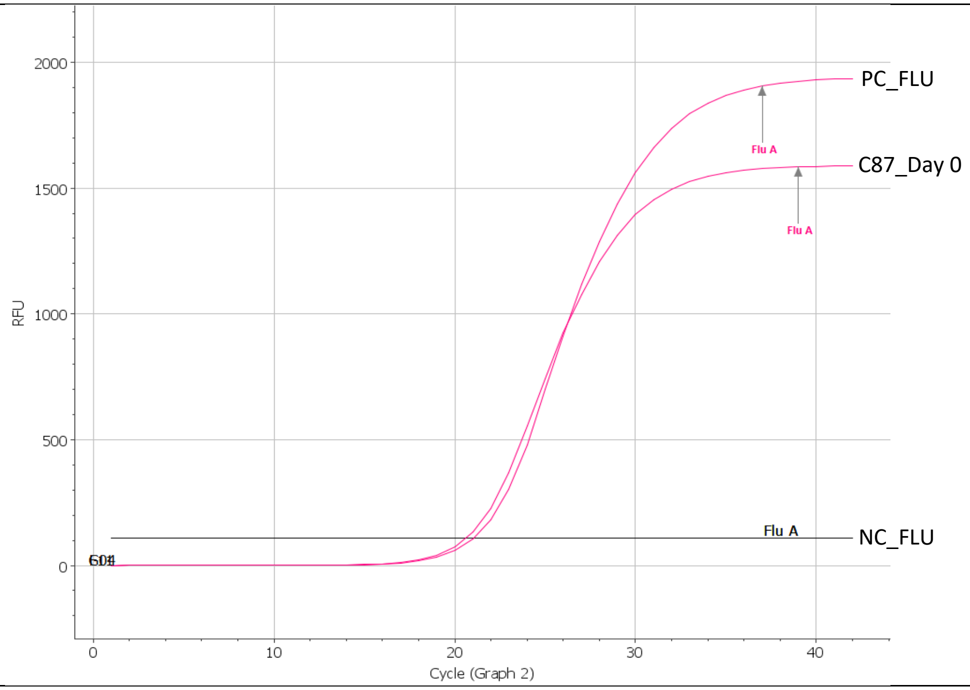2000
1500
1000
500
0
RFU
0
10
20
30
40
Cycle (Graph 2)
Flu A
Flu A
Flu A
PC_FLU
C87_Day 0
NC_FLU	

C87_D2

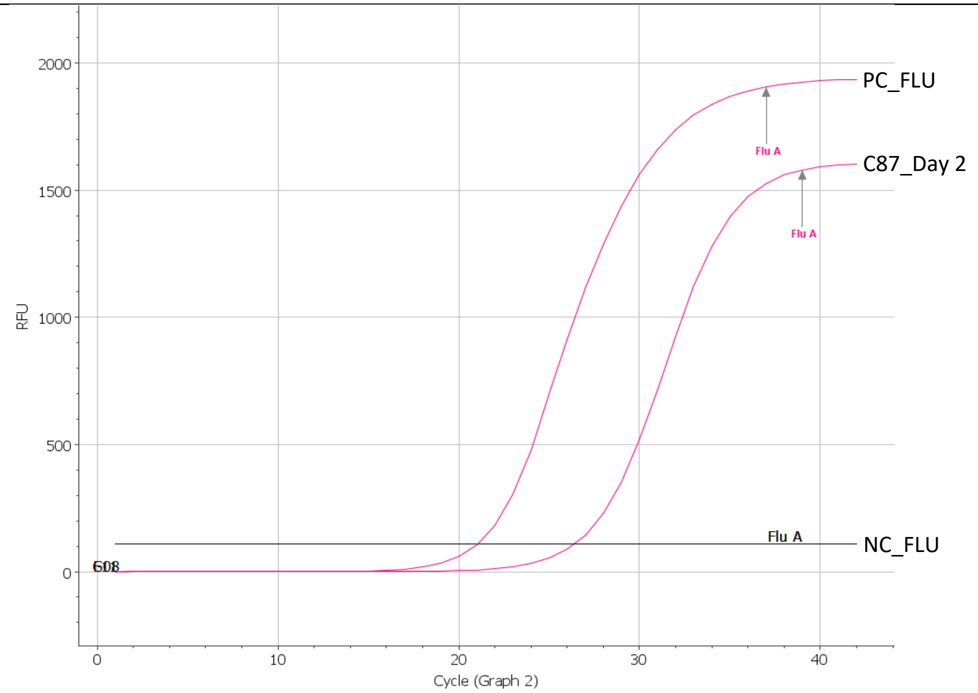
PC_FLU
C87_Day 2
NC_FLU
Flu A
Flu A
Flu A
608
RFU
2000
1500
1000
500
0
0
10
20
30
40
Cycle (Graph 2)

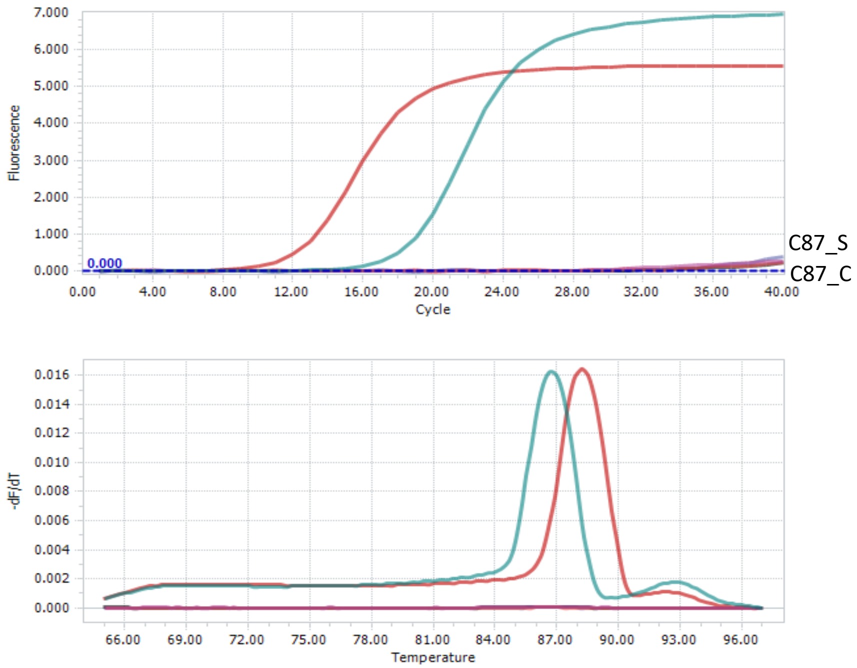
Fluorescence
Cycle
0.000
C87_S
C87_C
-dF/dT
Temperature

71	C88_D0	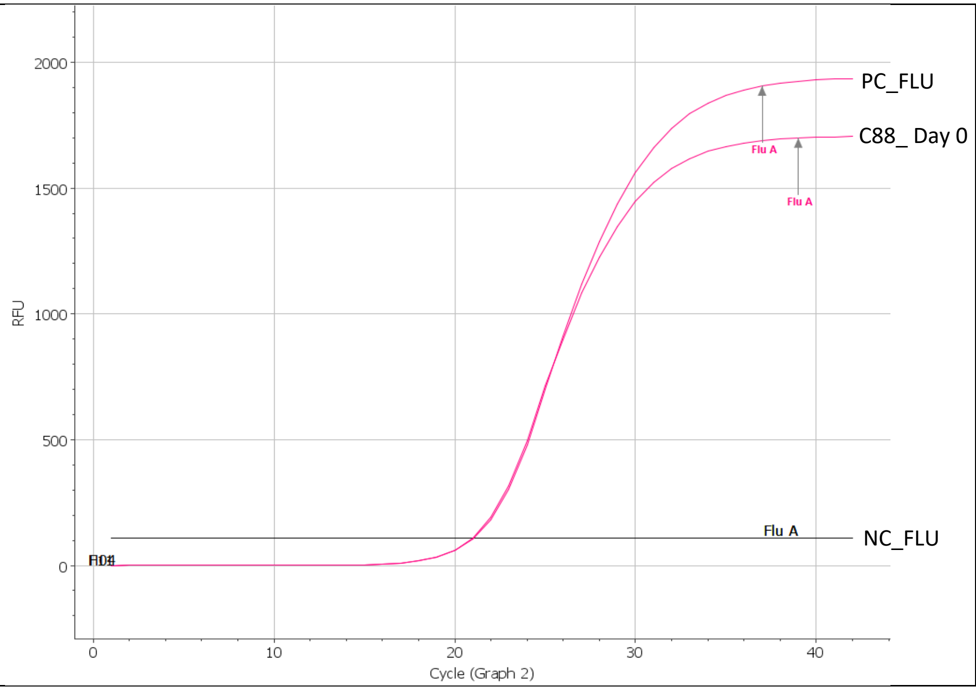2000
1500
1000
500
0
RFU
0
10
20
30
40
Cycle (Graph 2)
PC_FLU
C88_ Day 0
Flu A
Flu A
Flu A
NC_FLU	

C88_D2

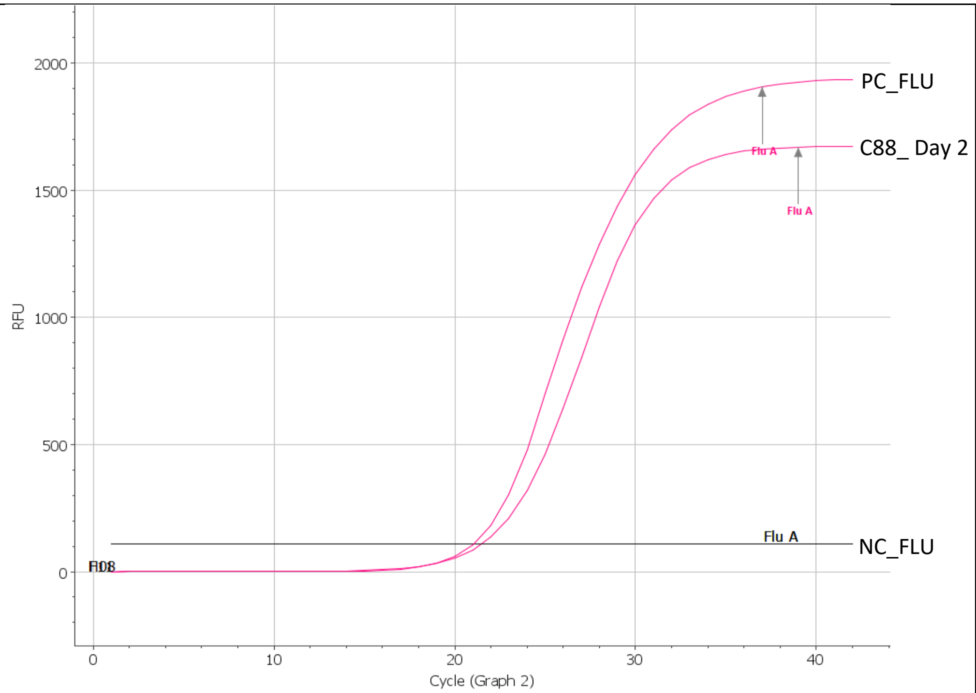

2000
1500
1000
500
0
RFU
0
10
20
30
40
Cycle (Graph 2)
PC_FLU
C88_ Day 2
NC_FLU
Flu A
Flu A
Flu A

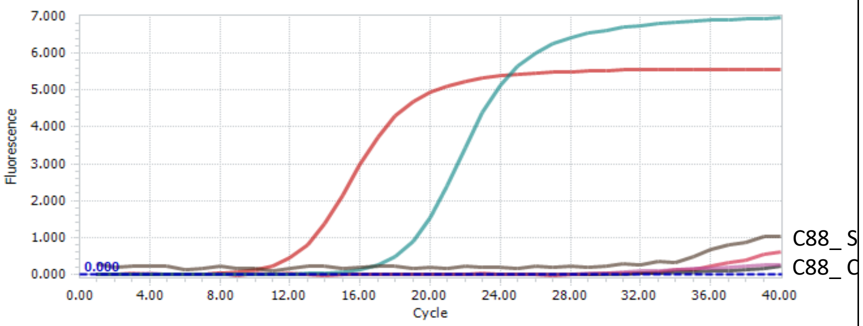

7.000
6.000
5.000
4.000
3.000
2.000
1.000
0.000
Fluorescence
0.000
0.00
4.00
8.00
12.00
16.00
20.00
24.00
28.00
32.00
36.00
40.00
Cycle
C88_ S
C88_ C

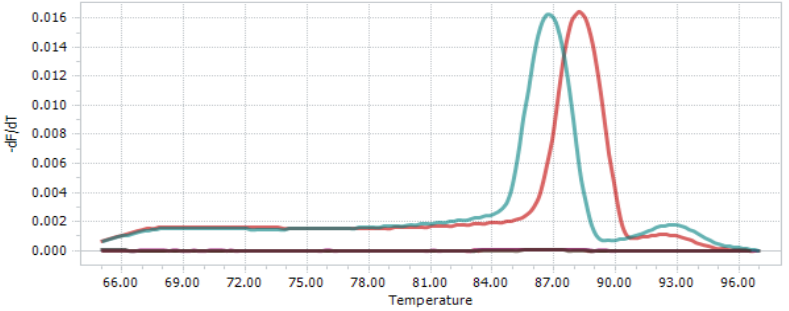

0.016
0.014
0.012
0.010
0.008
0.006
0.004
0.002
0.000
-dF/dT
66.00
69.00
72.00
75.00
78.00
81.00
84.00
87.00
90.00
93.00
96.00
Temperature

72	C89_D0	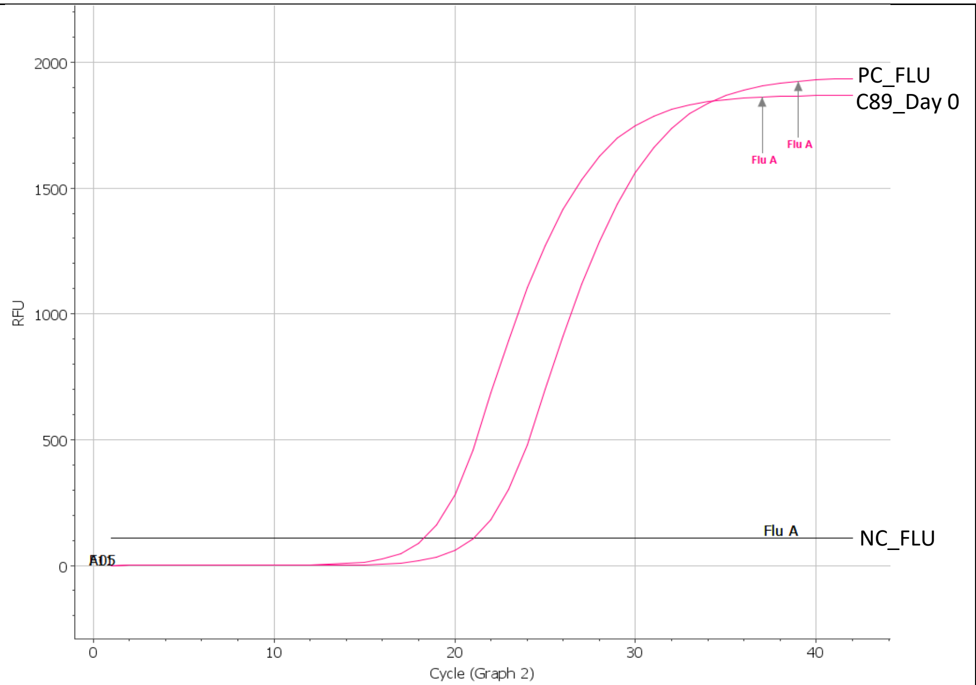	

PC_FLU
C89_Day 0
NC_FLU
Flu A
Flu A
Flu A
RFU
2000
1500
1000
500
0
0
10
20
30
40
Cycle (Graph 2)

	C89_D2	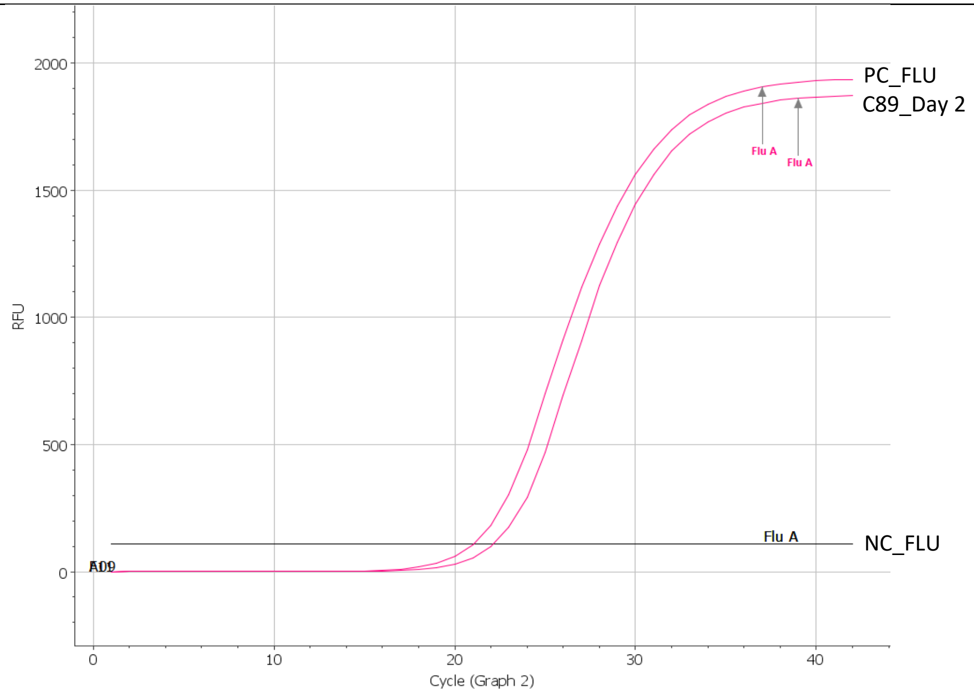 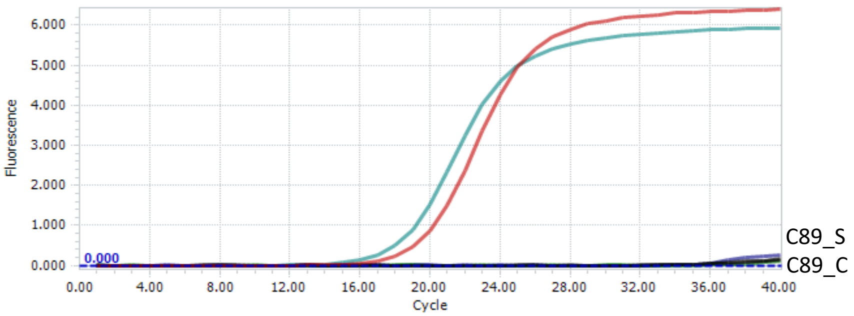 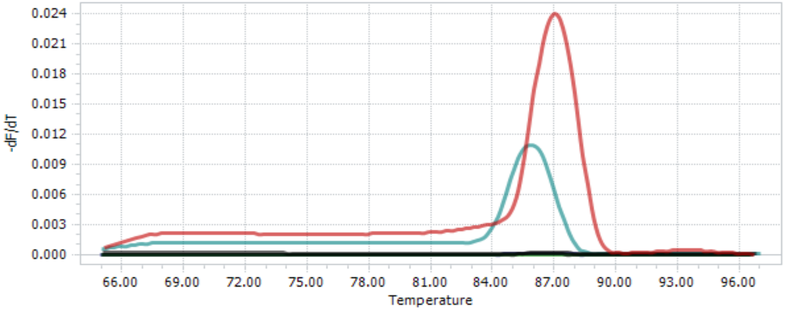

PC_FLU
C89_Day 2
NC_FLU
Flu A
Flu A
Flu A
RFU
Cycle (Graph 2)

Fluorescence
Cycle
C89_S
C89_C

-dF/dT
Temperature

73	C90_D0	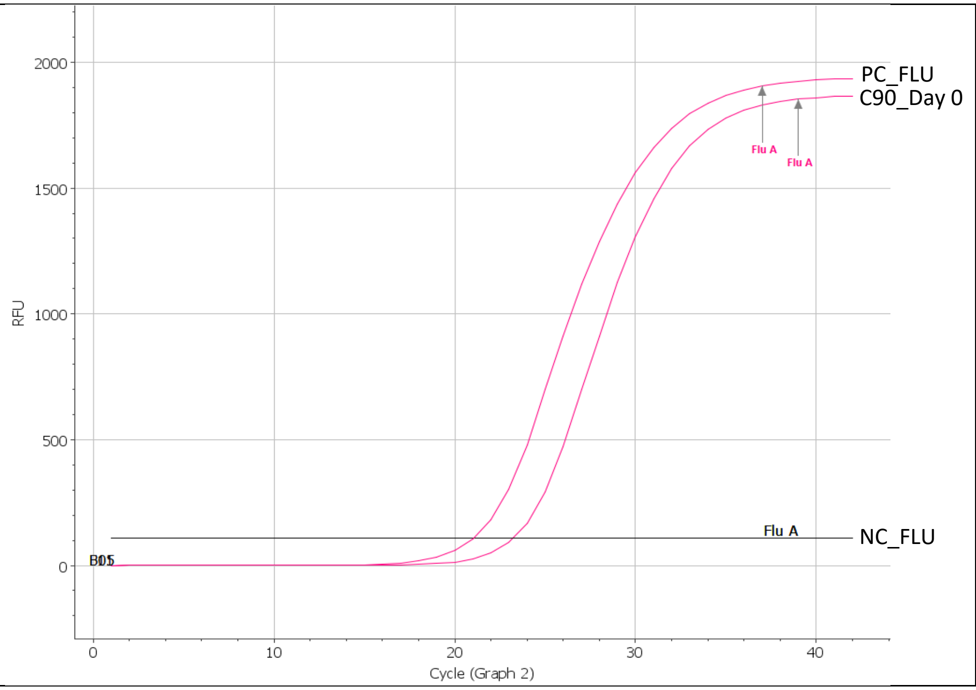RFU
2000
1500
1000
500
0
0
10
20
30
40
Cycle (Graph 2)
PC_FLU
C90_Day 0
Flu A
Flu A
Flu A
NC_FLU	

	C90_D2	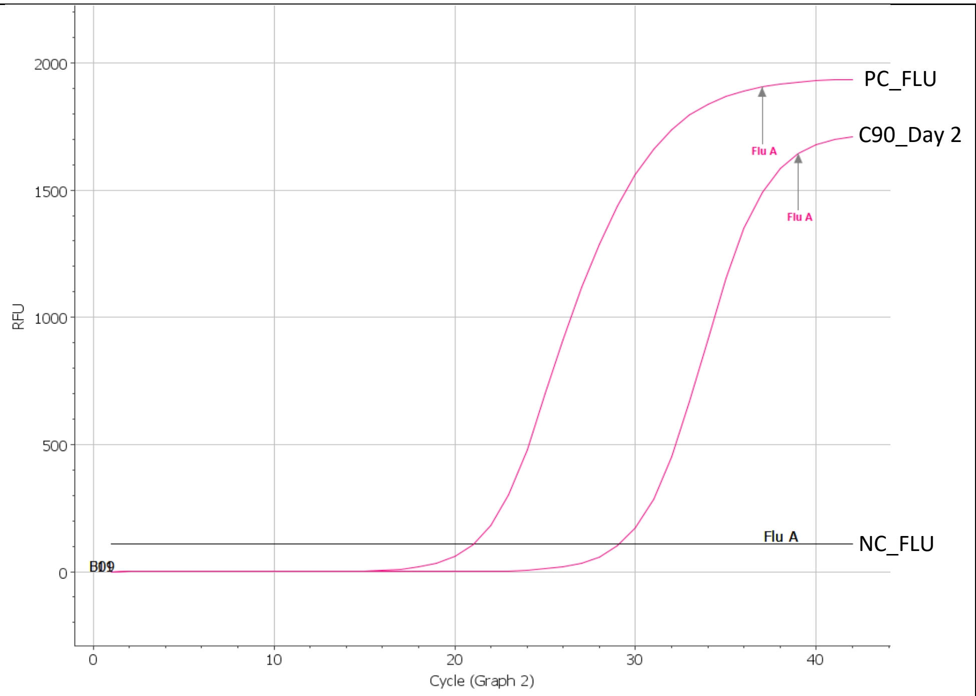	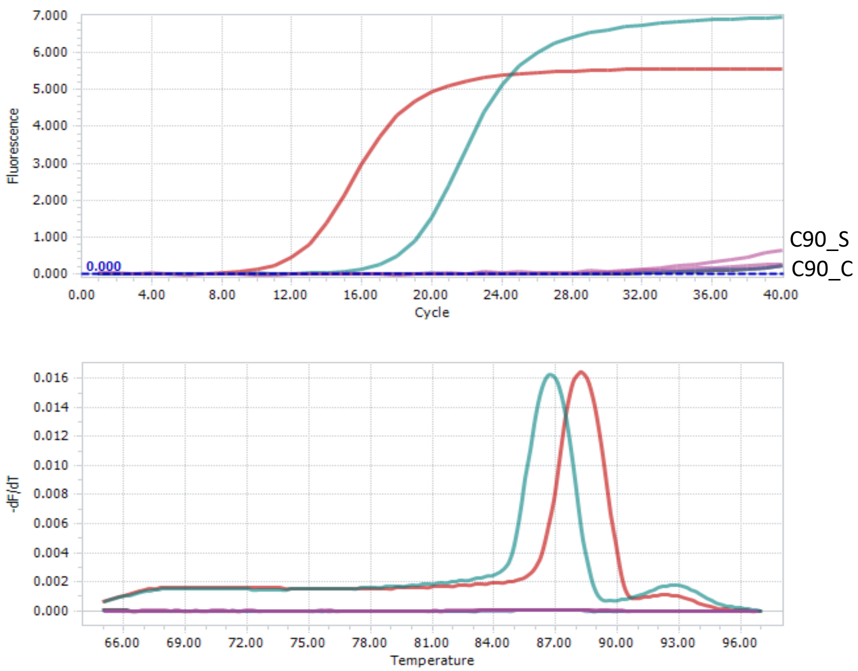

2000
1500
1000
500
0
RFU
0
10
20
30
40
Cycle (Graph 2)
PC_FLU
C90_Day 2
NC_FLU
Flu A
Flu A
Flu A

7.000
6.000
5.000
4.000
3.000
2.000
1.000
0.000
Fluorescence
0.000
0.00
4.00
8.00
12.00
16.00
20.00
24.00
28.00
32.00
36.00
40.00
Cycle
C90_S
C90_C
0.016
0.014
0.012
0.010
0.008
0.006
0.004
0.002
0.000
-dF/dT
66.00
69.00
72.00
75.00
78.00
81.00
84.00
87.00
90.00
93.00
96.00
Temperature

74	C91_D0	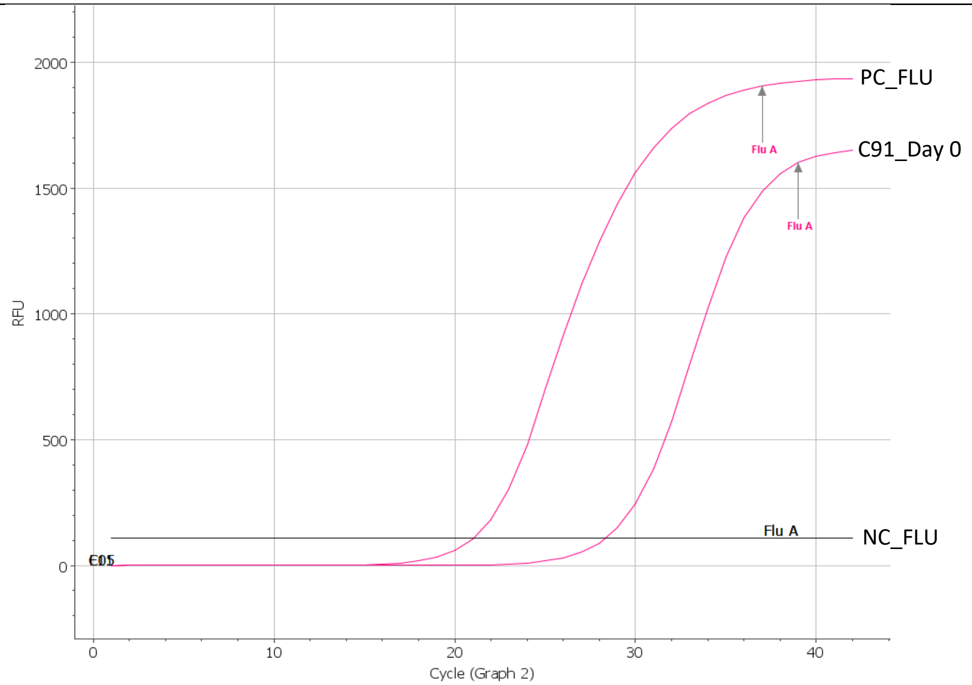PC_FLU<br>C91_Day 0<br>NC_FLU<br>Flu A<br>Flu A<br>Flu A<br>RFU<br>2000<br>1500<br>1000<br>500<br>0<br>0<br>10<br>20<br>30<br>40<br>Cycle (Graph 2)	

	C91_D2	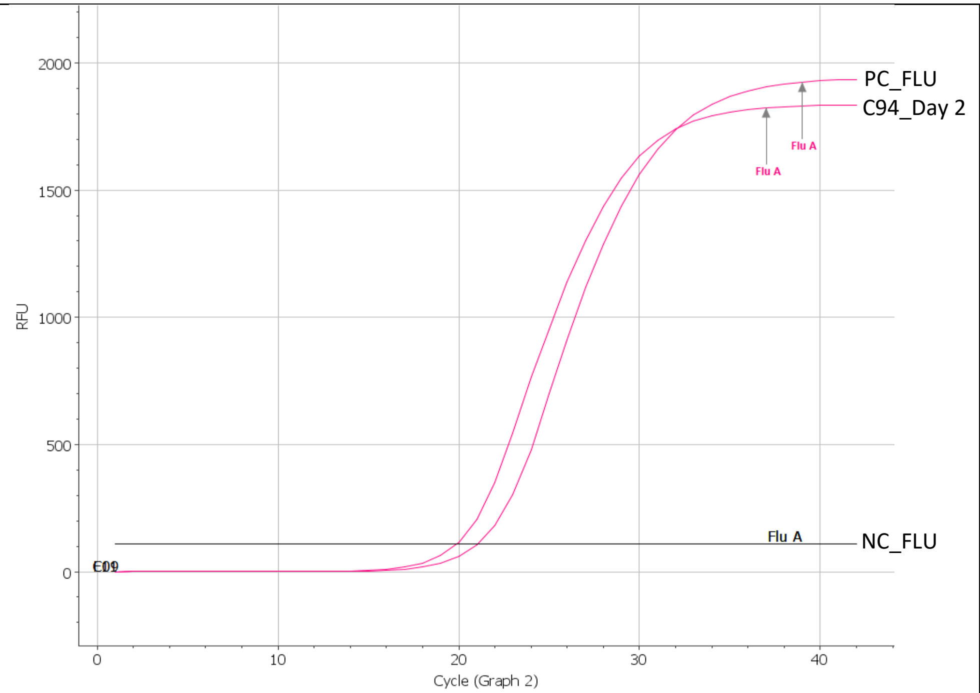 PC_FLU<br>C94_Day 2<br>NC_FLU<br>Flu A<br>Flu A<br>Flu A<br>RFU<br>Cycle (Graph 2) 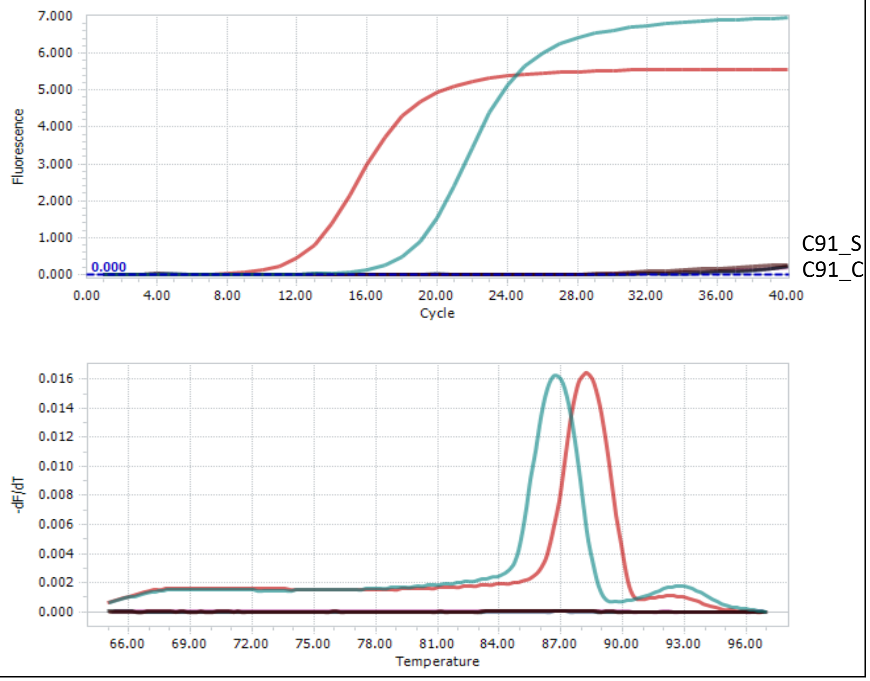 Fluorescence<br>Cycle<br>0.000<br>C91_S<br>C91_C<br>-dF/dT<br>Temperature

75	C92_D0	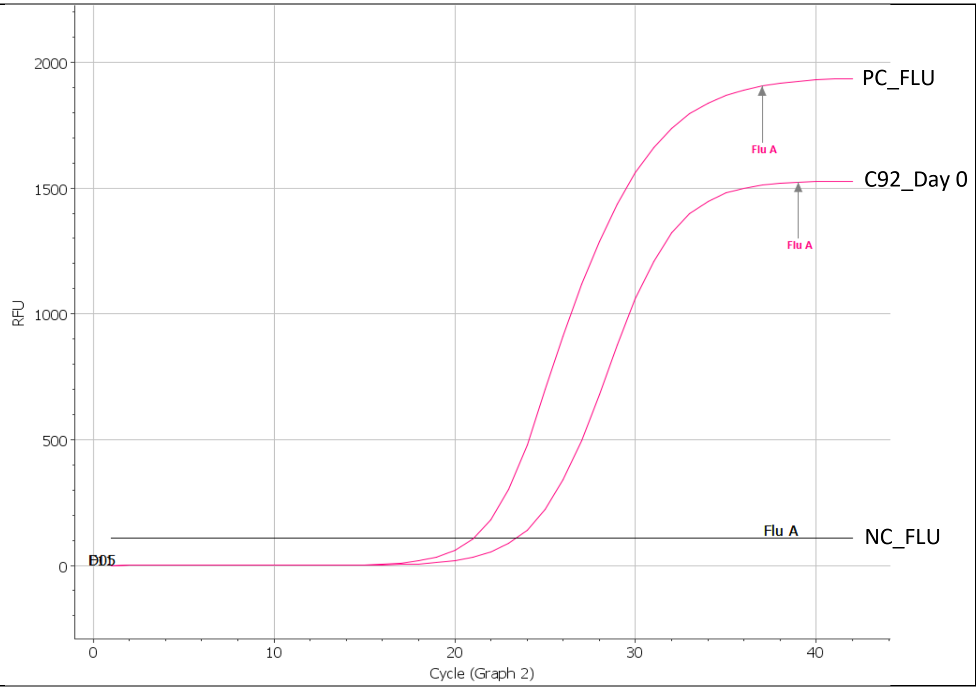 2000<br>1500<br>1000<br>500<br>0<br>RFU<br>0<br>10<br>20<br>30<br>40<br>Cycle (Graph 2)<br>PC_FLU<br>C92_Day 0<br>NC_FLU<br>Flu A<br>Flu A<br>Flu A	

C92 _D2

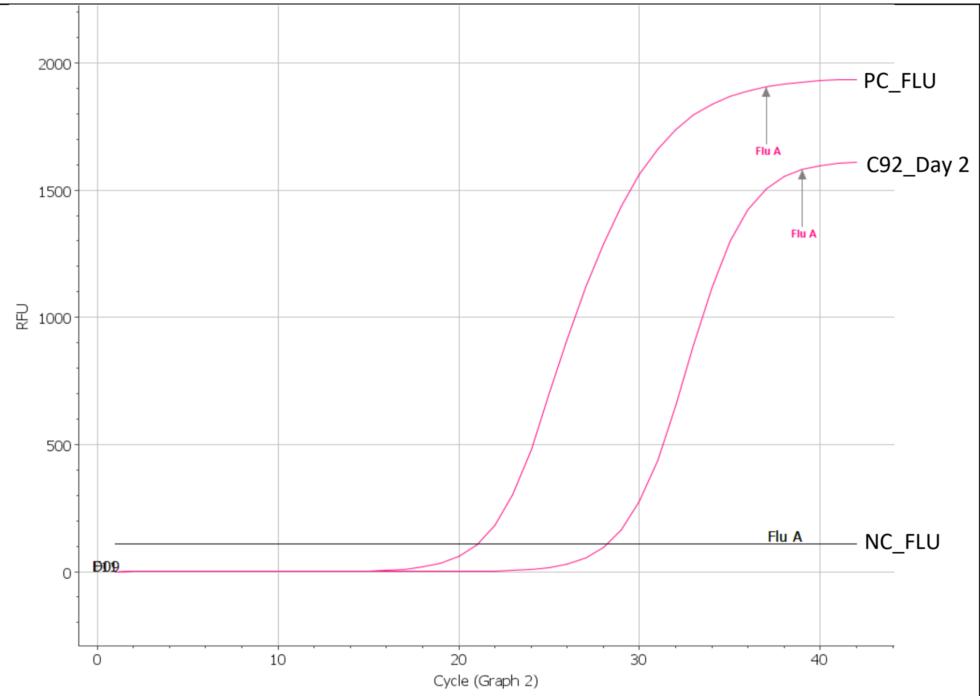
2000
1500
1000
500
0
RFU
0
10
20
30
40
Cycle (Graph 2)
PC_FLU
C92_Day 2
NC_FLU
Flu A
Flu A
Flu A
D09

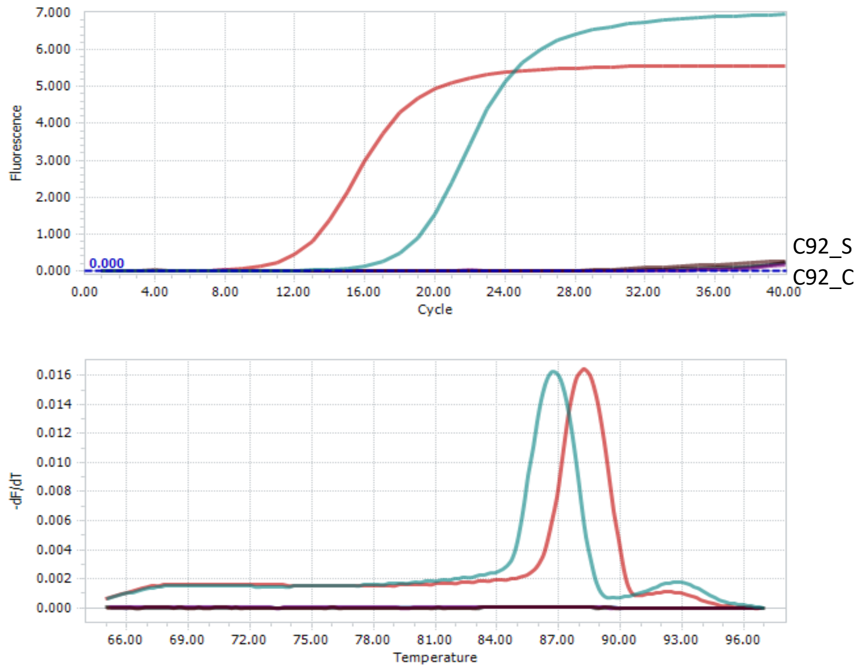
7.000
6.000
5.000
4.000
3.000
2.000
1.000
0.000
Fluorescence
0.000
0.00
4.00
8.00
12.00
16.00
20.00
24.00
28.00
32.00
36.00
40.00
Cycle
C92_S
C92_C
0.016
0.014
0.012
0.010
0.008
0.006
0.004
0.002
0.000
-dF/dT
66.00
69.00
72.00
75.00
78.00
81.00
84.00
87.00
90.00
93.00
96.00
Temperature

76	C93_D0	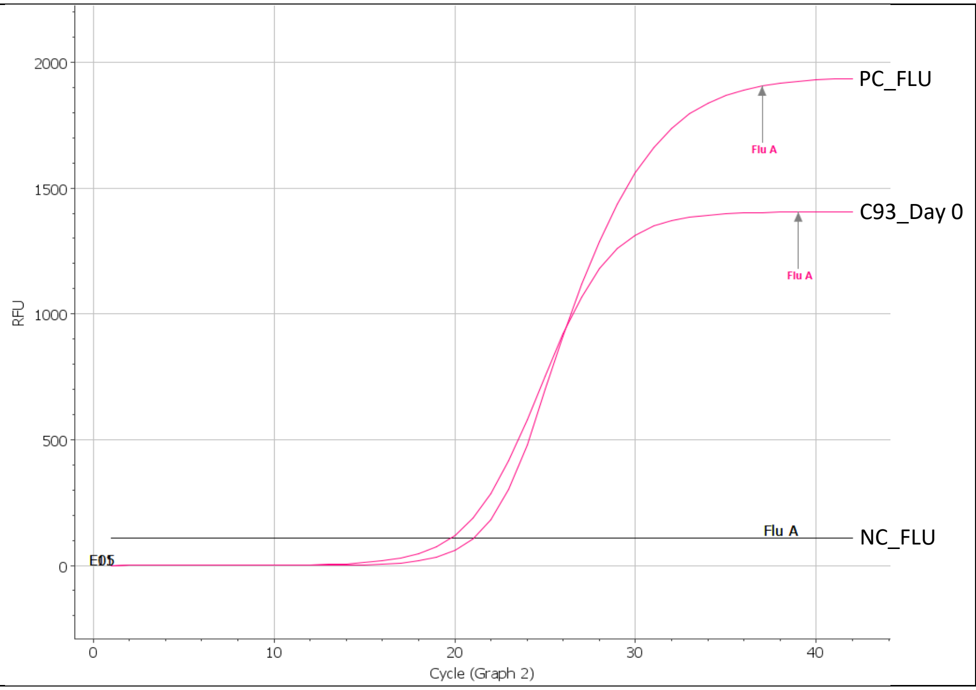2000<br>1500<br>1000<br>500<br>0<br>RFU<br>0<br>10<br>20<br>30<br>40<br>Cycle (Graph 2)<br>Flu A<br>Flu A<br>Flu A<br>E05<br>PC_FLU<br>C93_Day 0<br>NC_FLU	

	C93 _D2	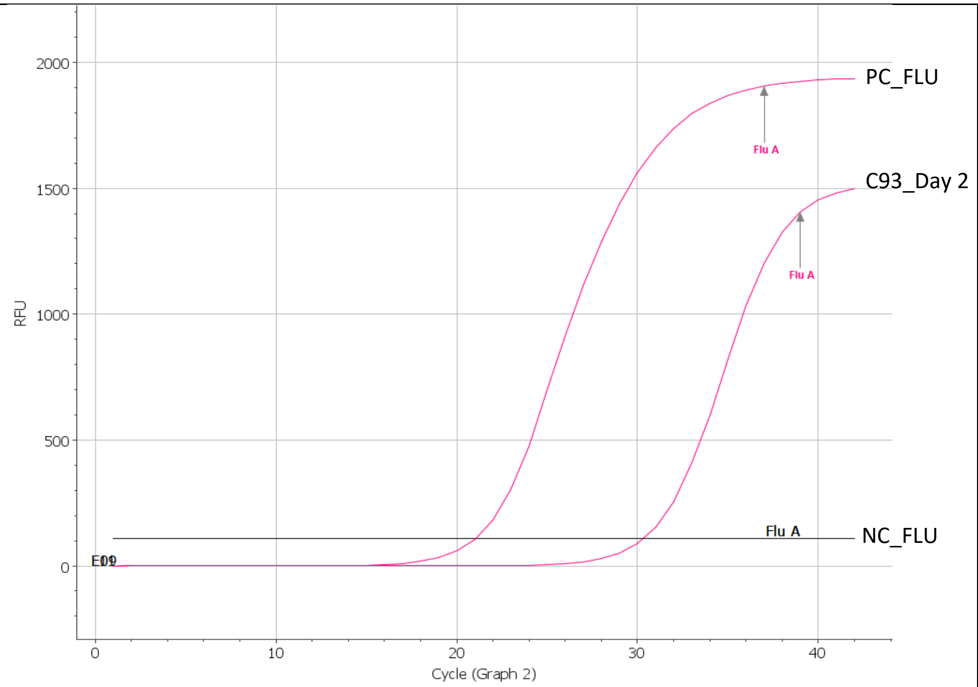2000<br>1500<br>1000<br>500<br>0<br>RFU<br>0<br>10<br>20<br>30<br>40<br>Cycle (Graph 2)<br>Flu A<br>Flu A<br>Flu A<br>E09<br>PC_FLU<br>C93_Day 2<br>NC_FLU	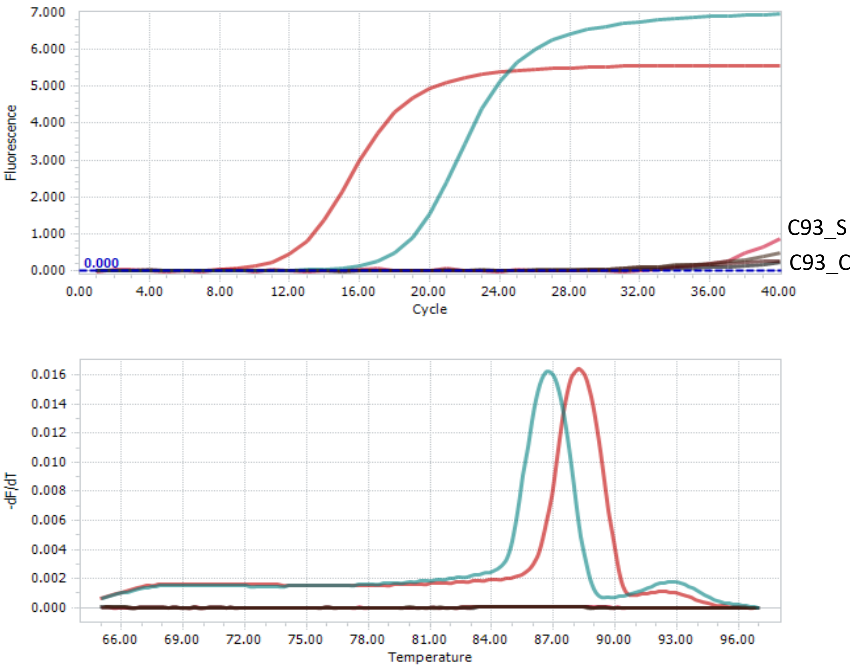7.000<br>6.000<br>5.000<br>4.000<br>3.000<br>2.000<br>1.000<br>0.000<br>0.000<br>Fluorescence<br>0.00<br>4.00<br>8.00<br>12.00<br>16.00<br>20.00<br>24.00<br>28.00<br>32.00<br>36.00<br>40.00<br>Cycle<br>C93_S<br>C93_C<br>0.016<br>0.014<br>0.012<br>0.010<br>0.008<br>0.006<br>0.004<br>0.002<br>0.000<br>-dF/dT<br>66.00<br>69.00<br>72.00<br>75.00<br>78.00<br>81.00<br>84.00<br>87.00<br>90.00<br>93.00<br>96.00<br>Temperature

77	C94_D0	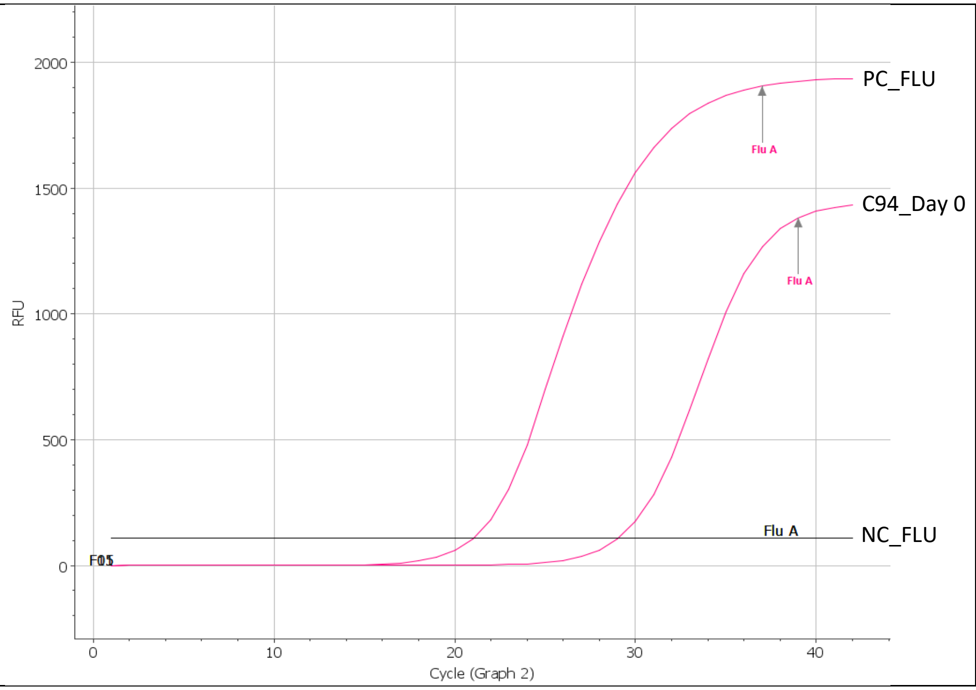	

2000
1500
1000
500
0
RFU
0
10
20
30
40
Cycle (Graph 2)
PC_FLU
C94_Day 0
NC_FLU
Flu A
Flu A
Flu A

	C94_D2	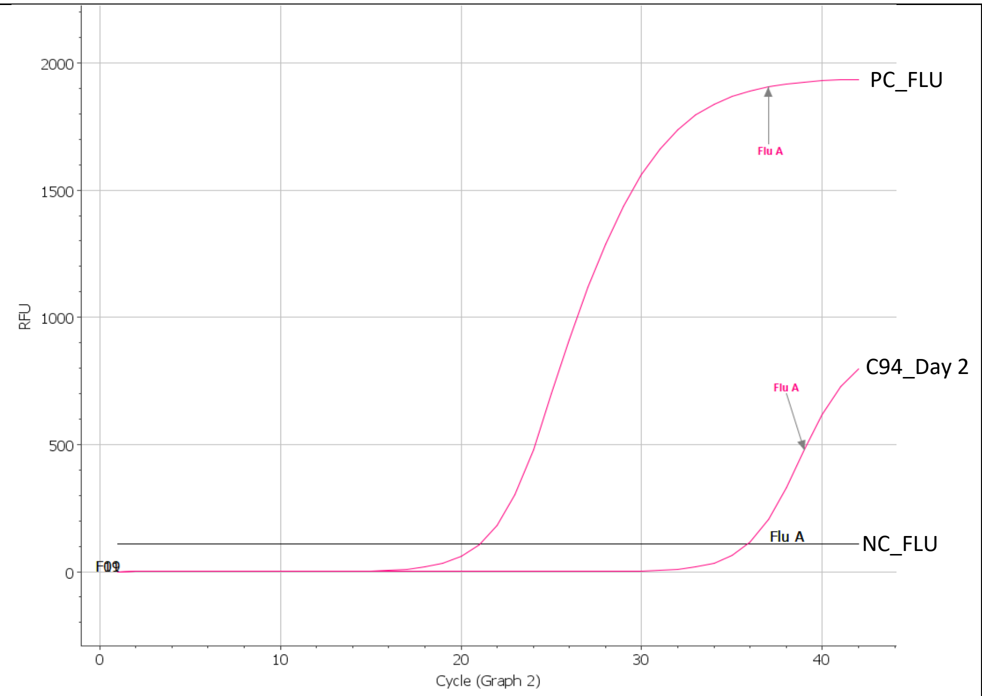 2000 1500 1000 500 0 RFU 0 10 20 30 40 Cycle (Graph 2) PC_FLU Flu A C94_Day 2 Flu A NC_FLU Flu A	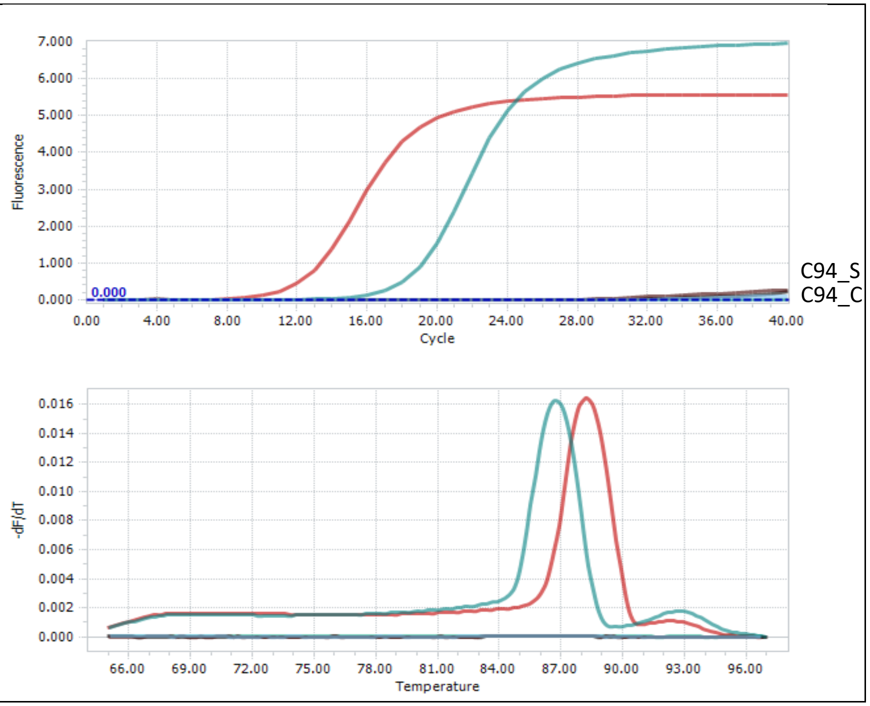 7.000 6.000 5.000 4.000 3.000 2.000 1.000 0.000 Fluorescence 0.000 0.00 4.00 8.00 12.00 16.00 20.00 24.00 28.00 32.00 36.00 40.00 Cycle C94_S C94_C 0.016 0.014 0.012 0.010 0.008 0.006 0.004 0.002 0.000 -dF/dT 66.00 69.00 72.00 75.00 78.00 81.00 84.00 87.00 90.00 93.00 96.00 Temperature

78	C95_D0	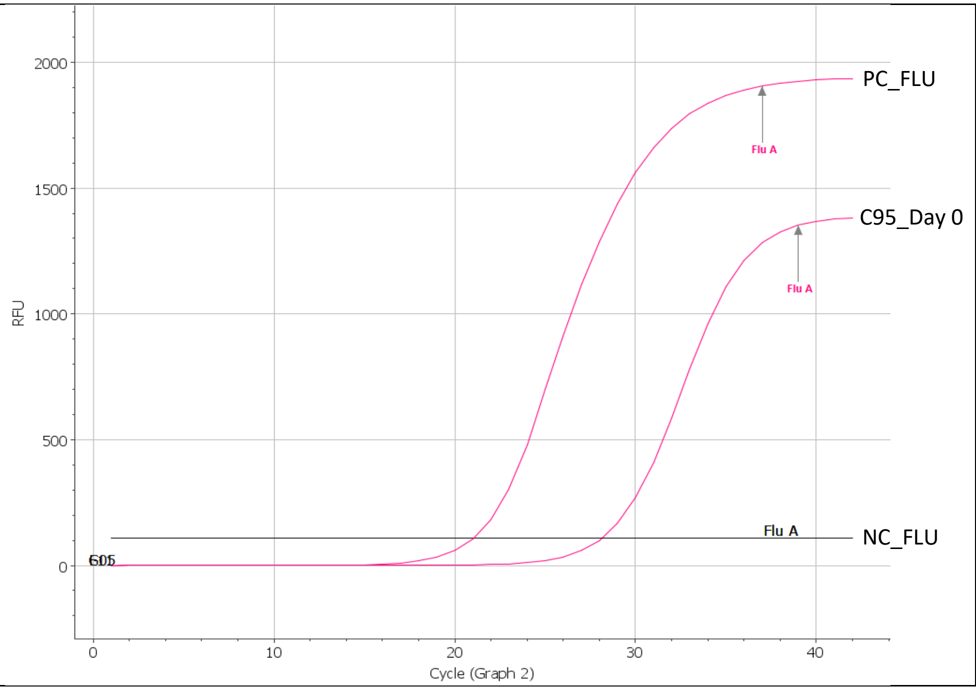	

PC_FLU
C95_Day 0
NC_FLU
Flu A
Flu A
Flu A
RFU
2000
1500
1000
500
0
0
10
20
30
40
Cycle (Graph 2)

	C95_D2	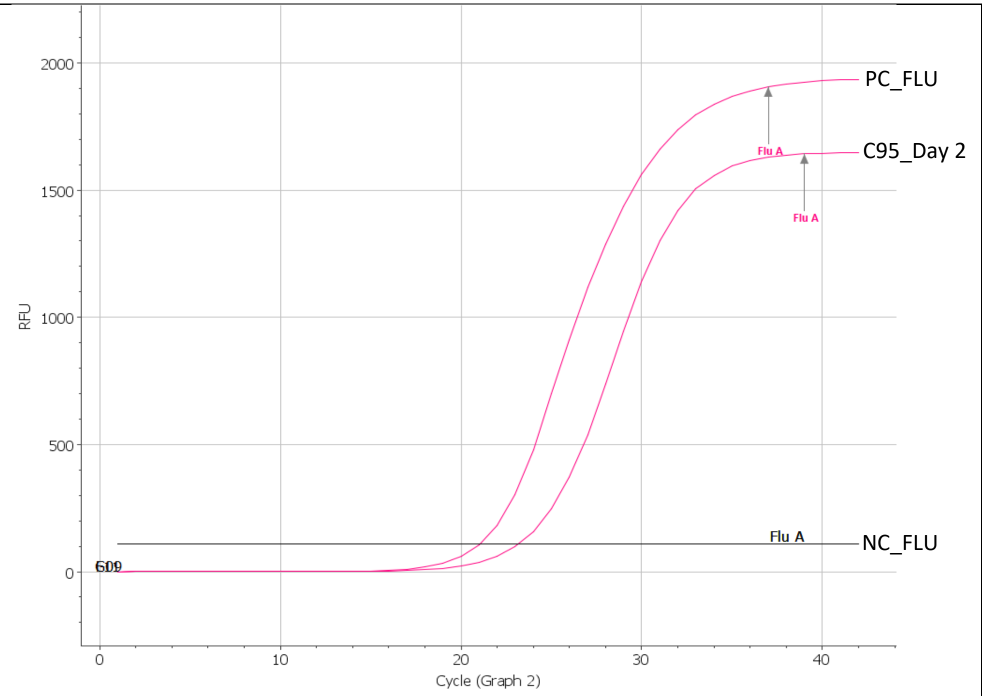 PC_FLU<br>C95_Day 2<br>NC_FLU<br>Flu A<br>Flu A<br>Flu A<br>RFU<br>2000<br>1500<br>1000<br>500<br>0<br>0 10 20 30 40<br>Cycle (Graph 2)	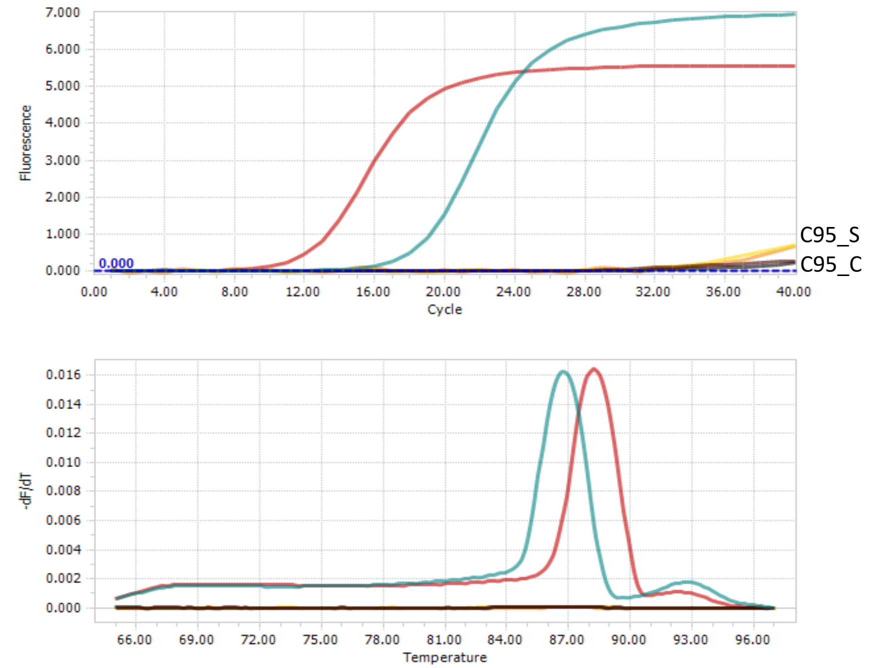 Fluorescence<br>7.000<br>6.000<br>5.000<br>4.000<br>3.000<br>2.000<br>1.000<br>0.000<br>0.00 4.00 8.00 12.00 16.00 20.00 24.00 28.00 32.00 36.00 40.00<br>Cycle<br>C95_S<br>C95_C<br>-dF/dT<br>0.016<br>0.014<br>0.012<br>0.010<br>0.008<br>0.006<br>0.004<br>0.002<br>0.000<br>66.00 69.00 72.00 75.00 78.00 81.00 84.00 87.00 90.00 93.00 96.00<br>Temperature

79	C96_D0	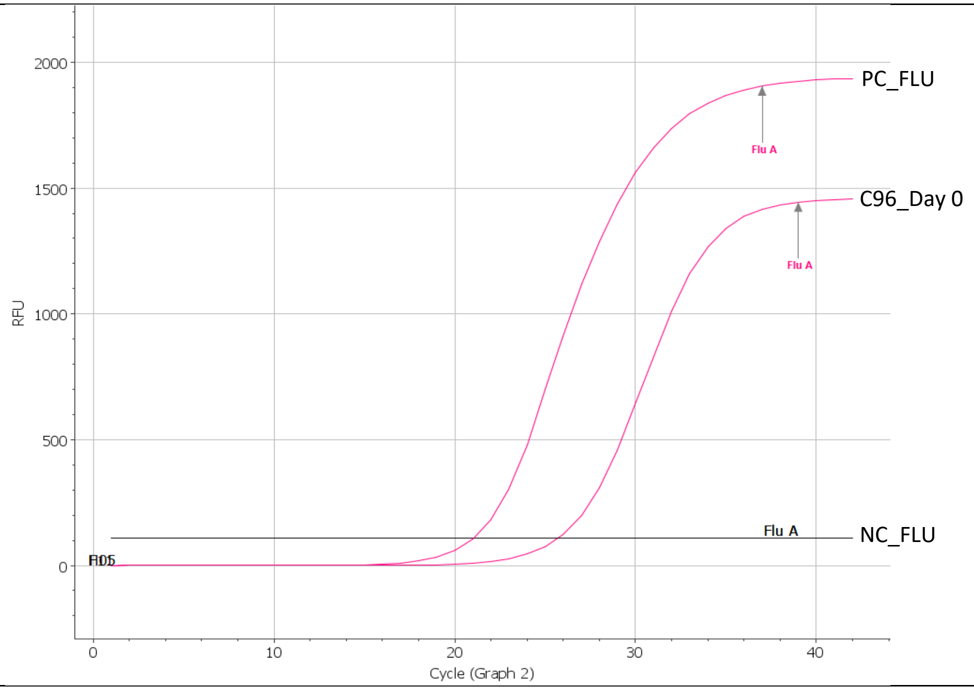PC_FLU
C96_Day 0
NC_FLU
Flu A
Flu A
Flu A
RFU
2000
1500
1000
500
0
0
10
20
30
40
Cycle (Graph 2)	

	C96_D2	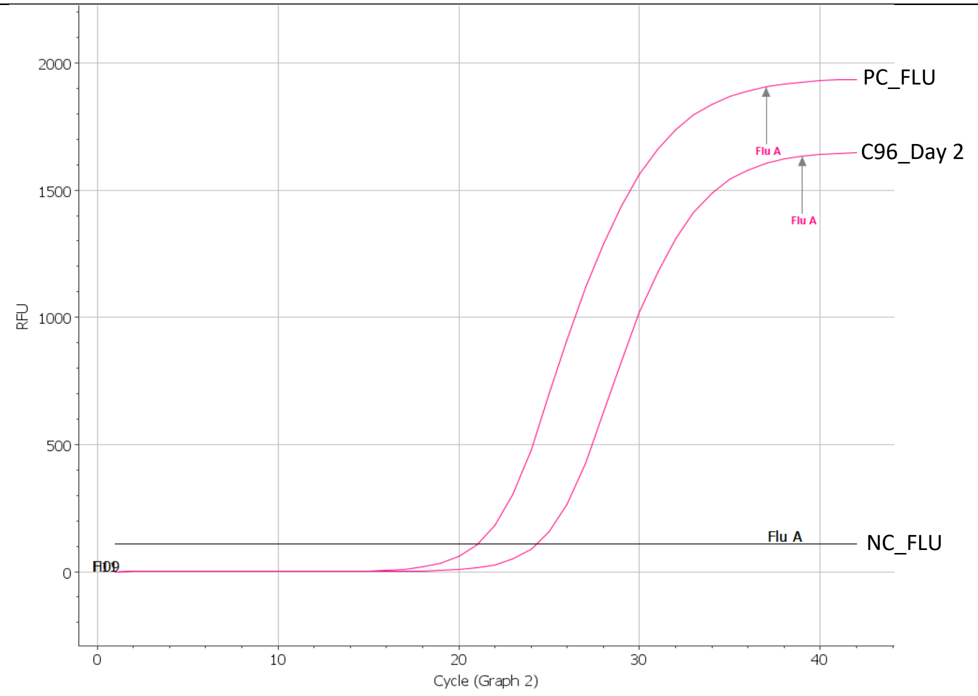2000<br>1500<br>1000<br>500<br>0<br>RFU<br>PC_FLU<br>Flu A<br>C96_Day 2<br>Flu A<br>Flu A<br>NC_FLU<br>0<br>10<br>20<br>30<br>40<br>Cycle (Graph 2)	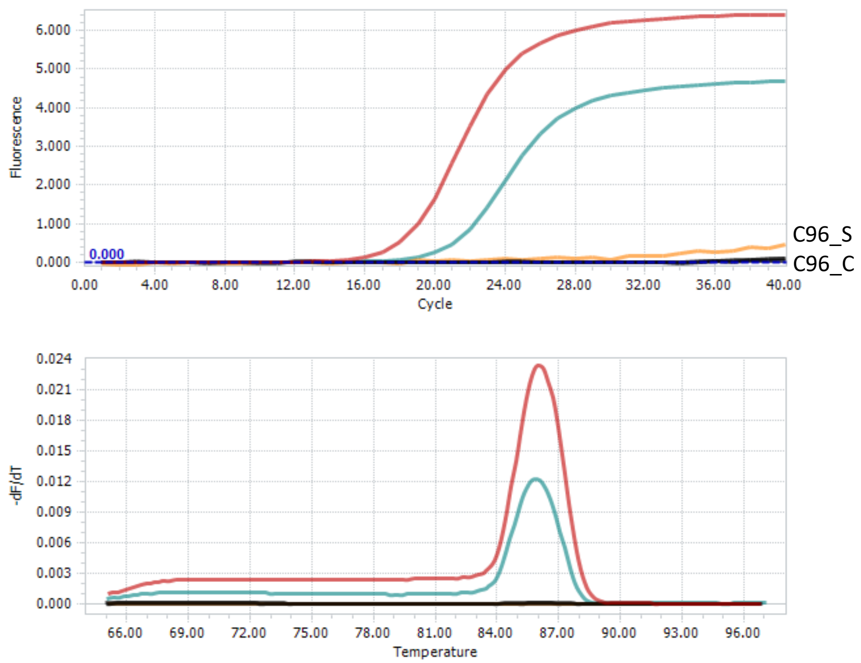6.000<br>5.000<br>4.000<br>3.000<br>2.000<br>1.000<br>0.000<br>Fluorescence<br>0.000<br>C96_S<br>C96_C<br>0.00<br>4.00<br>8.00<br>12.00<br>16.00<br>20.00<br>24.00<br>28.00<br>32.00<br>36.00<br>40.00<br>Cycle<br>0.024<br>0.021<br>0.018<br>0.015<br>0.012<br>0.009<br>0.006<br>0.003<br>0.000<br>-dF/dT<br>66.00<br>69.00<br>72.00<br>75.00<br>78.00<br>81.00<br>84.00<br>87.00<br>90.00<br>93.00<br>96.00<br>Temperature

80	C97_D0	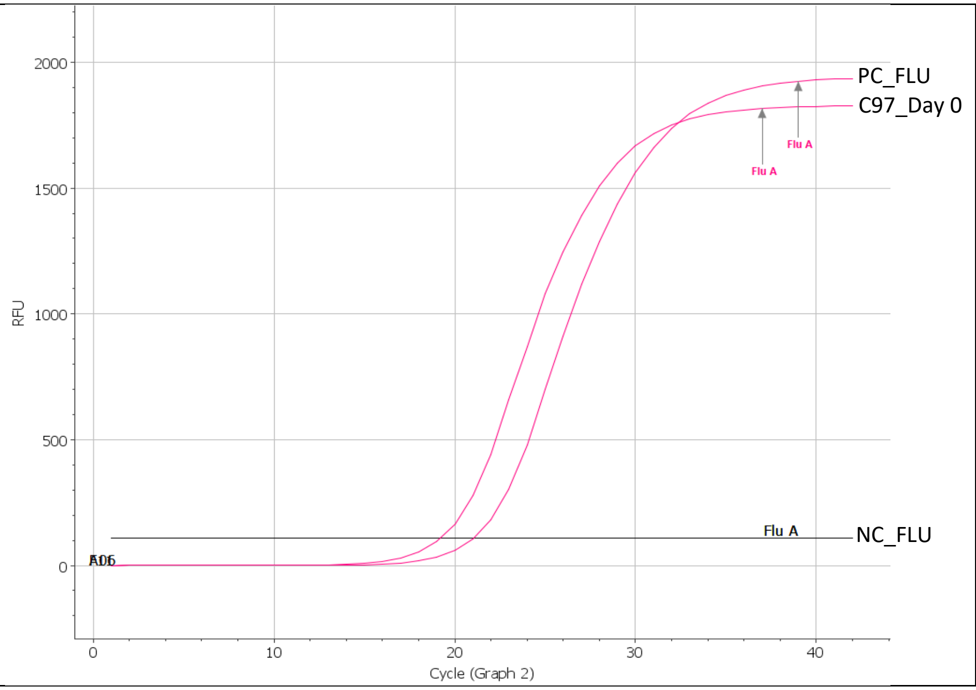2000<br>1500<br>1000<br>500<br>0<br>RFU<br>0<br>10<br>20<br>30<br>40<br>Cycle (Graph 2)<br>Flu A<br>Flu A<br>Flu A<br>PC_FLU<br>C97_Day 0<br>NC_FLU	

	C97_D2	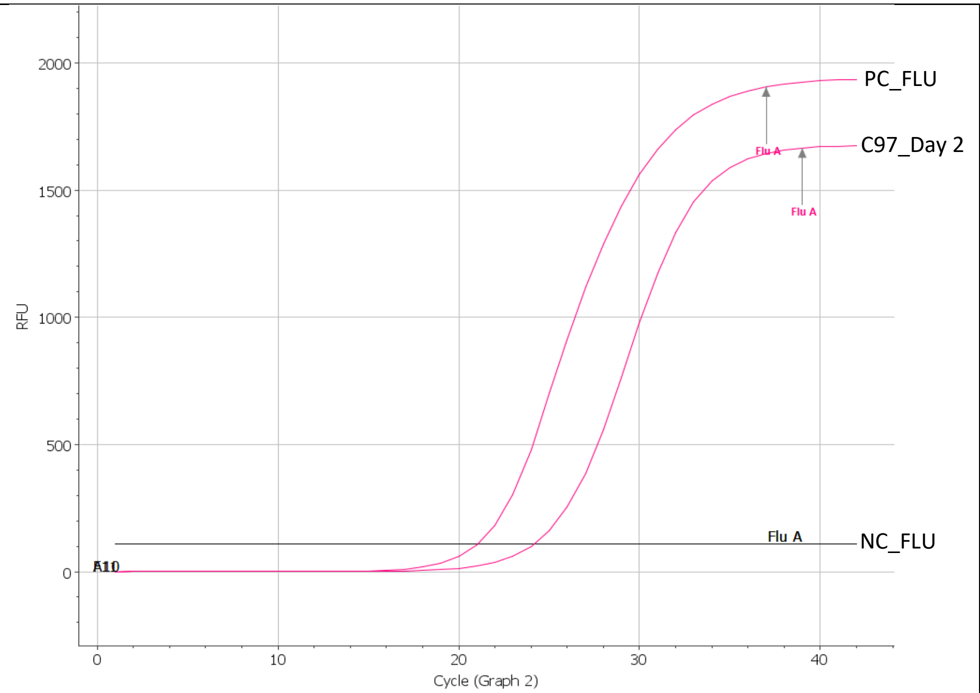	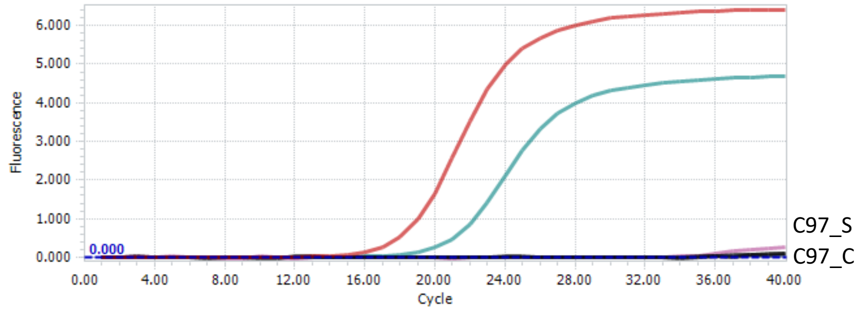 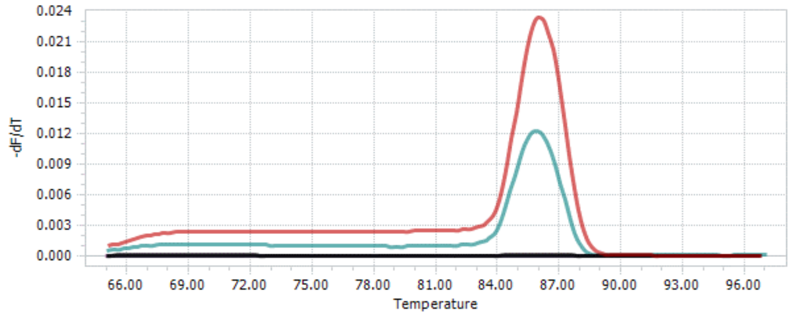

PC_FLU
C97_Day 2
NC_FLU
Flu A
Flu A
Flu A
A10
RFU
2000
1500
1000
500
0
0
10
20
30
40
Cycle (Graph 2)

Fluorescence
6.000
5.000
4.000
3.000
2.000
1.000
0.000
0.000
0.00
4.00
8.00
12.00
16.00
20.00
24.00
28.00
32.00
36.00
40.00
Cycle
C97_S
C97_C

-dF/dT
0.024
0.021
0.018
0.015
0.012
0.009
0.006
0.003
0.000
66.00
69.00
72.00
75.00
78.00
81.00
84.00
87.00
90.00
93.00
96.00
Temperature

81	C98_D0	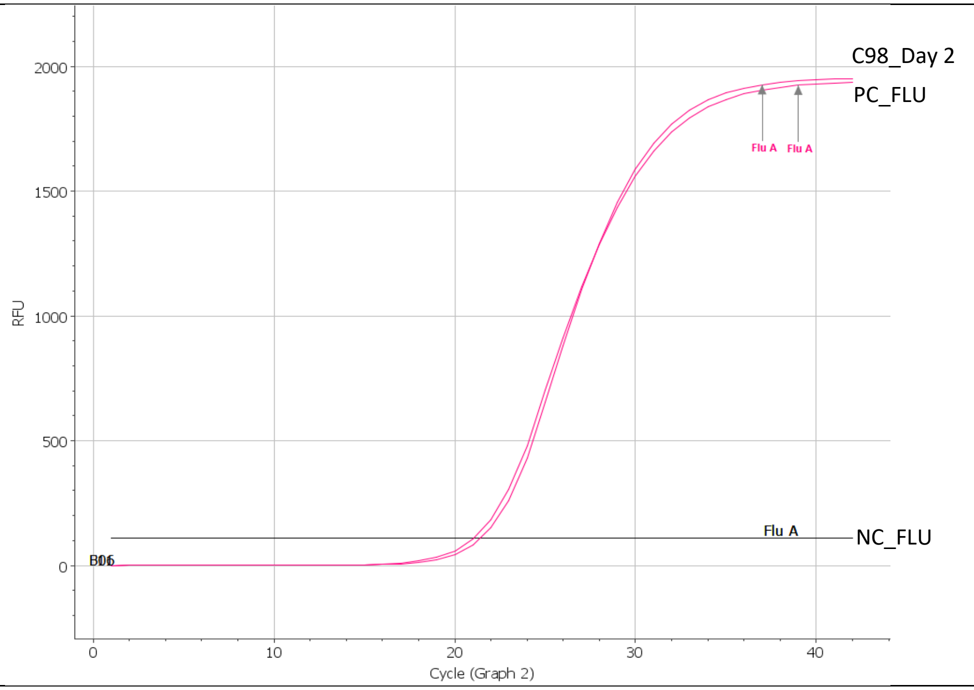C98_Day 2
PC_FLU
Flu A
Flu A
Flu A
NC_FLU
B06
RFU
2000
1500
1000
500
0
0
10
20
30
40
Cycle (Graph 2)	

C98_D2

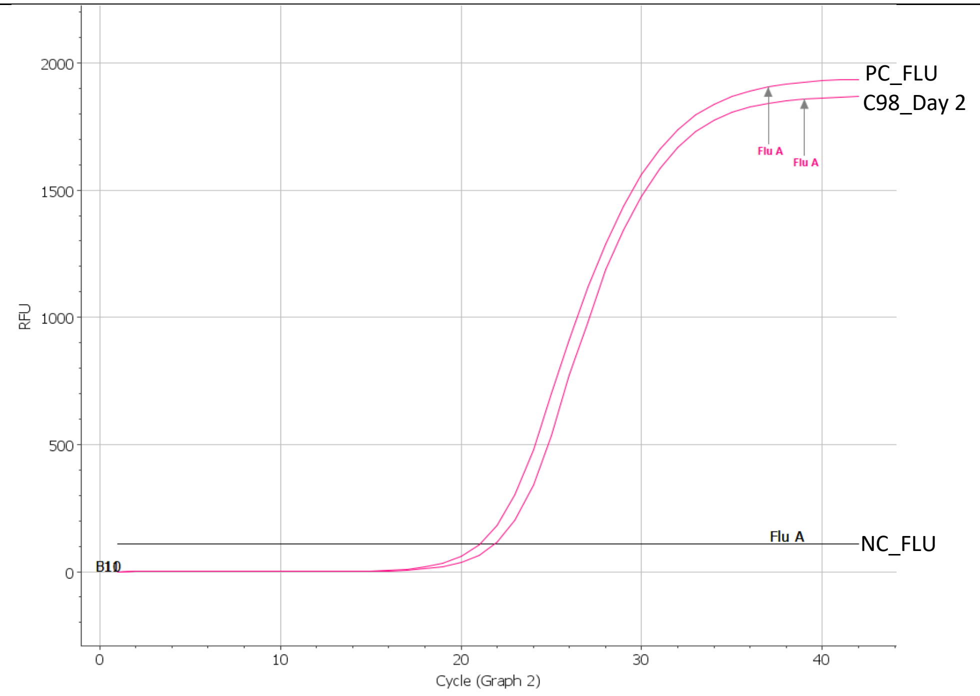
PC_FLU
C98_Day 2
NC_FLU
Flu A
Flu A
Flu A
B10
RFU
Cycle (Graph 2)

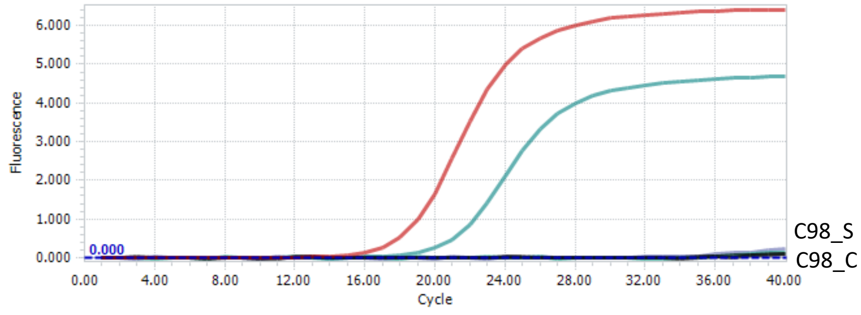
Fluorescence
Cycle
0.000
C98_S
C98_C

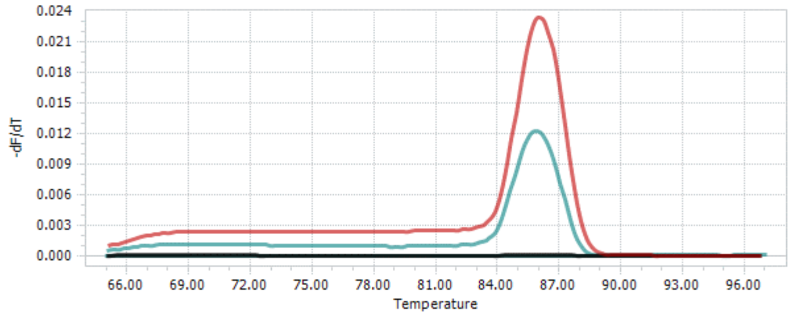
-dF/dT
Temperature

82	C99_D0	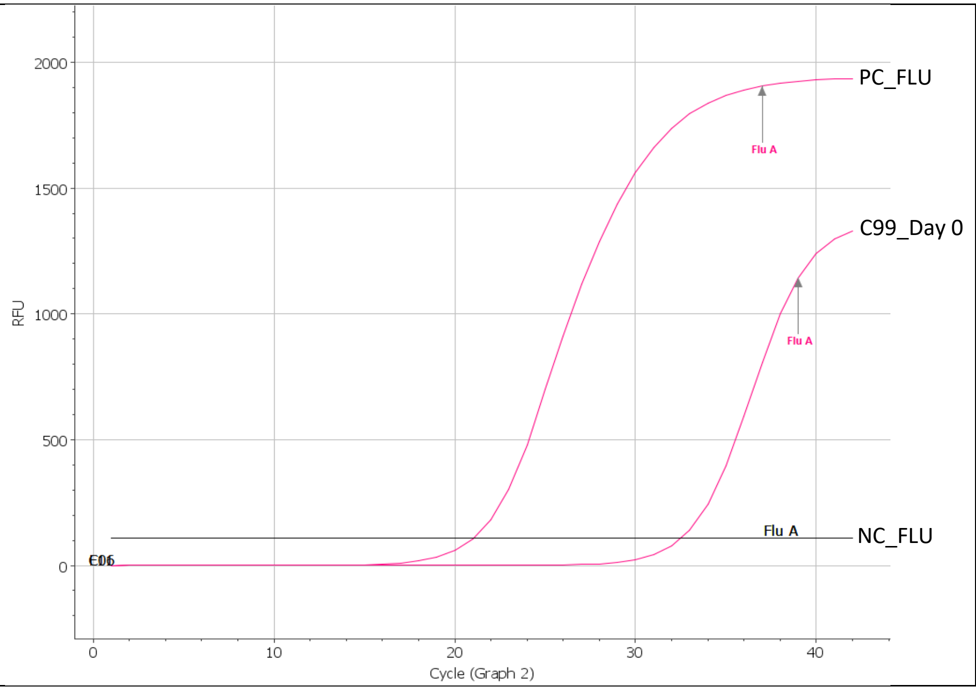2000<br>1500<br>1000<br>500<br>0<br>RFU<br>0<br>10<br>20<br>30<br>40<br>Cycle (Graph 2)<br>PC_FLU<br>Flu A<br>C99_Day 0<br>Flu A<br>Flu A<br>NC_FLU	

	C99_D2	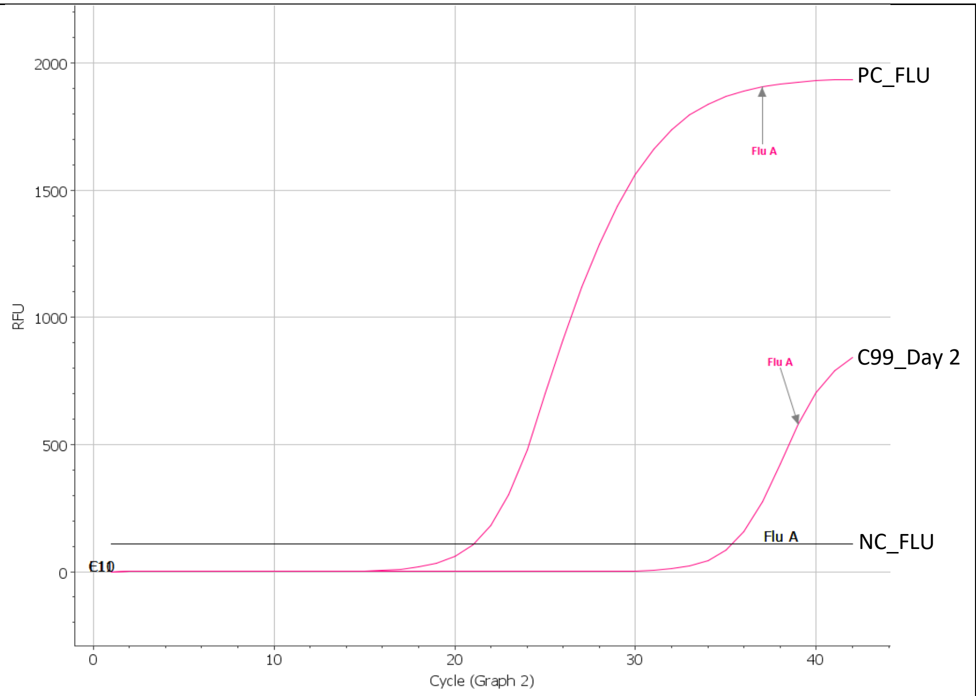	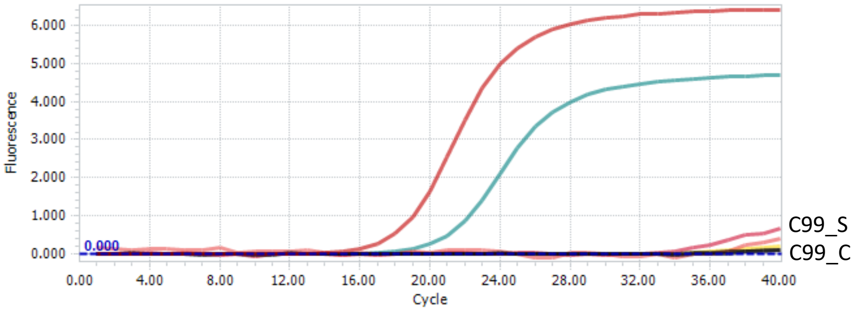<br>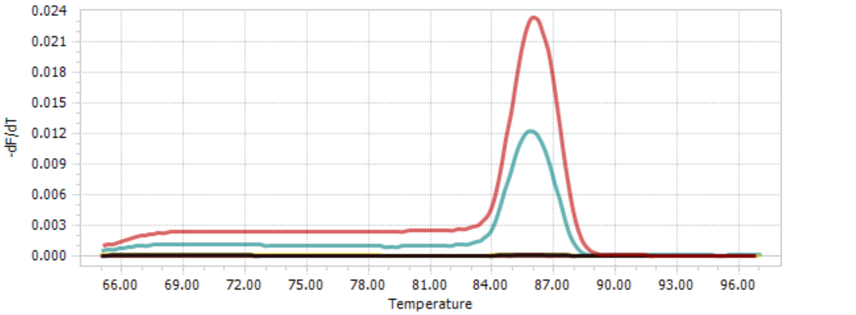

PC_FLU
Flu A
C99_Day 2
Flu A
NC_FLU
Flu A
RFU
Cycle (Graph 2)

Fluorescence
Cycle
C99_S
C99_C

-dF/dT
Temperature

83	C100_D 0	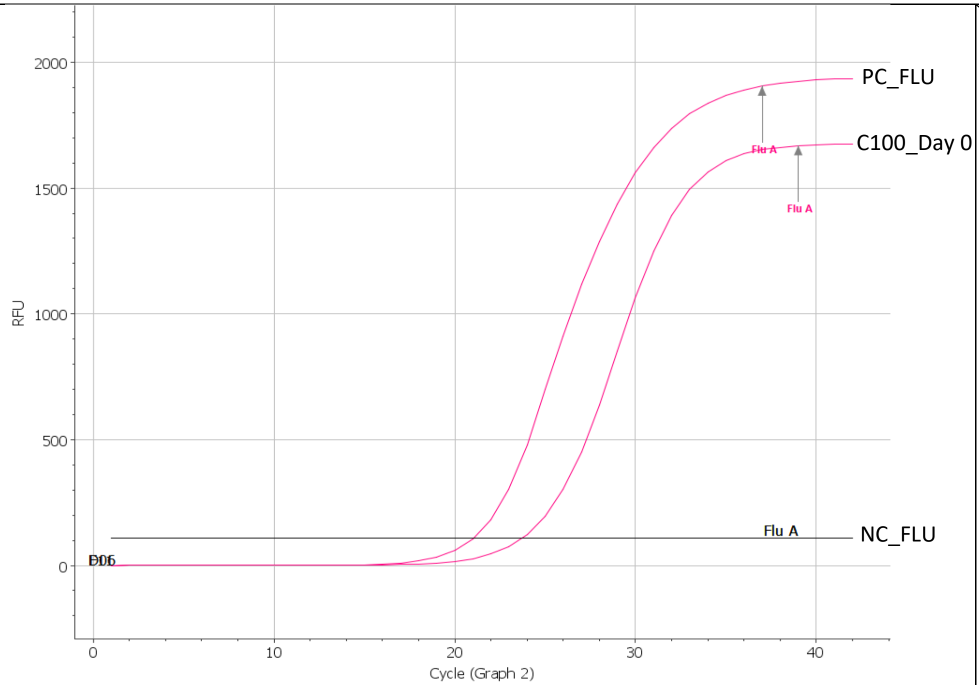	

PC_FLU
C100_Day 0
NC_FLU
Flu A
RFU
2000
1500
1000
500
0
10
20
30
40
Cycle (Graph 2)

<table>
<tr>
<td></td>
<td>C100_D
2</td>
<td>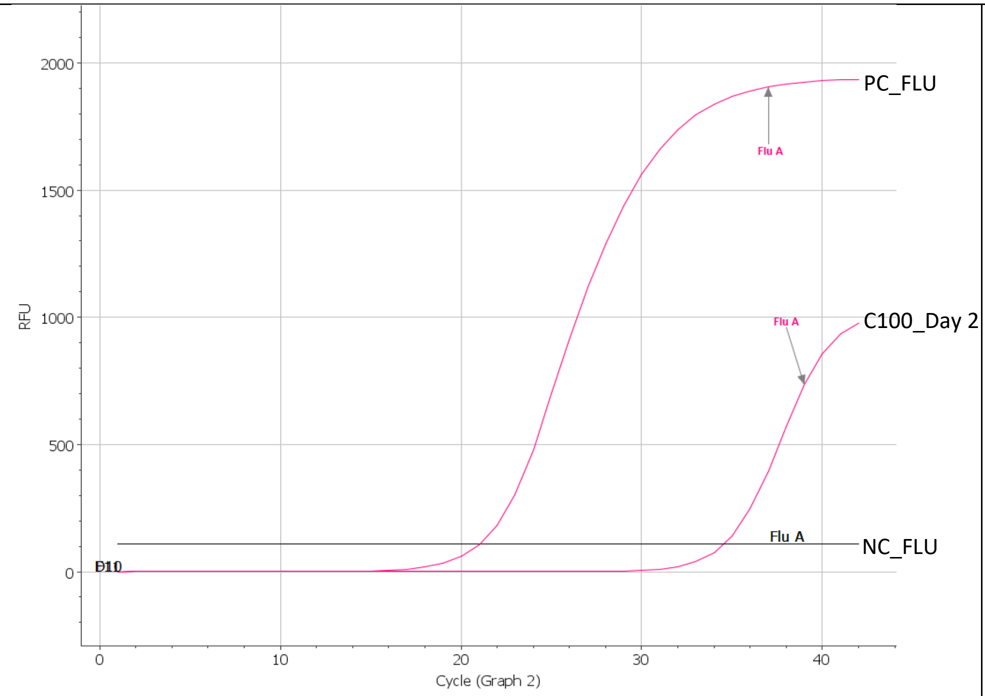
</td>
<td>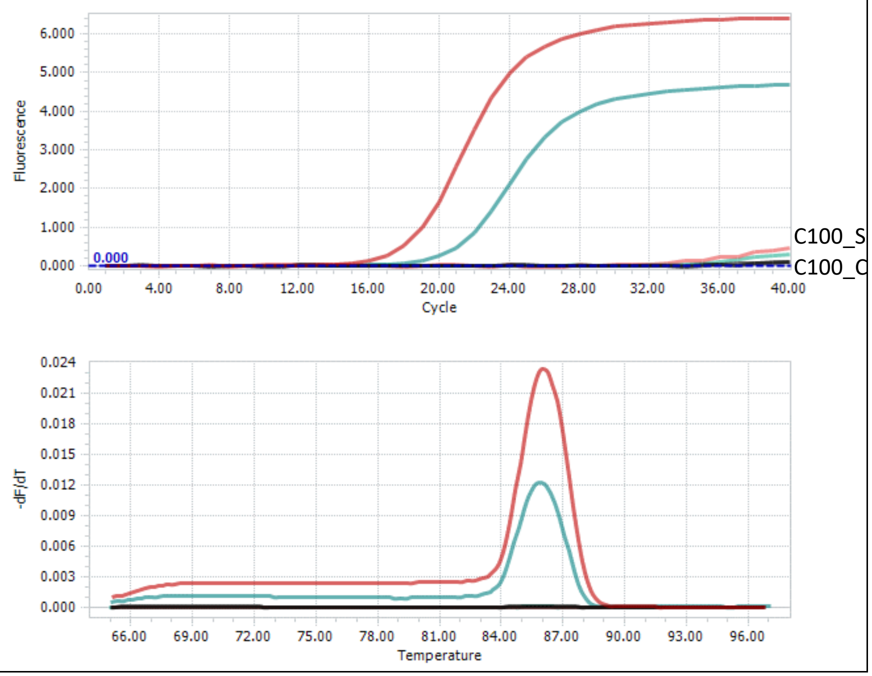
</td>
</tr>
</table>

84	C101_D 0	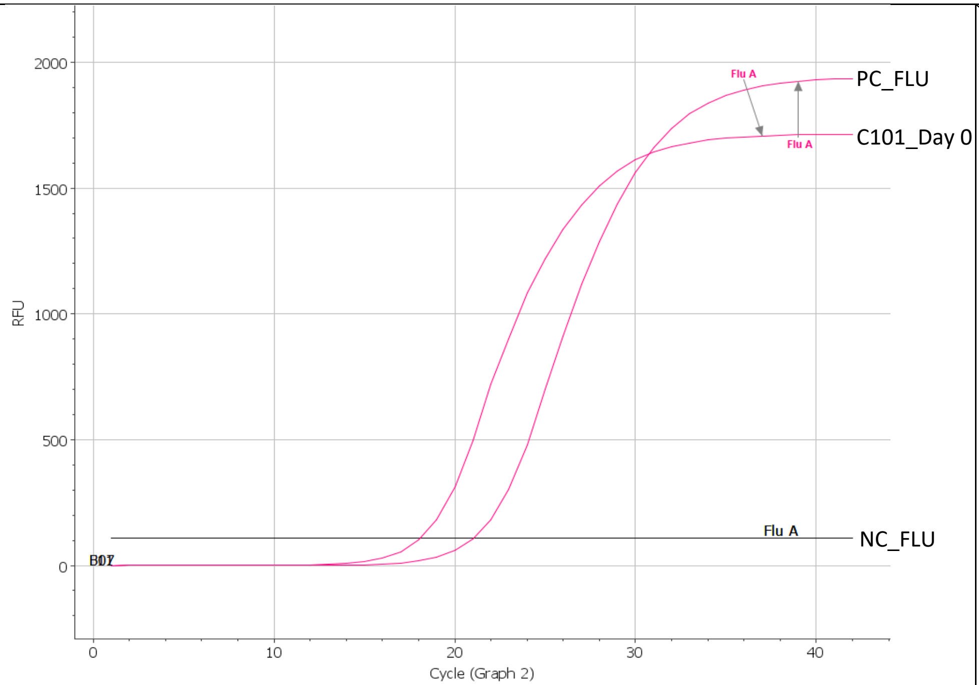PC_FLU<br>C101_Day 0<br>NC_FLU<br>Flu A<br>Flu A<br>Flu A<br>RFU<br>Cycle (Graph 2)<br>2000<br>1500<br>1000<br>500<br>0<br>0<br>10<br>20<br>30<br>40	

<table>
<tr>
<td></td>
<td>C101_D
2</td>
<td>
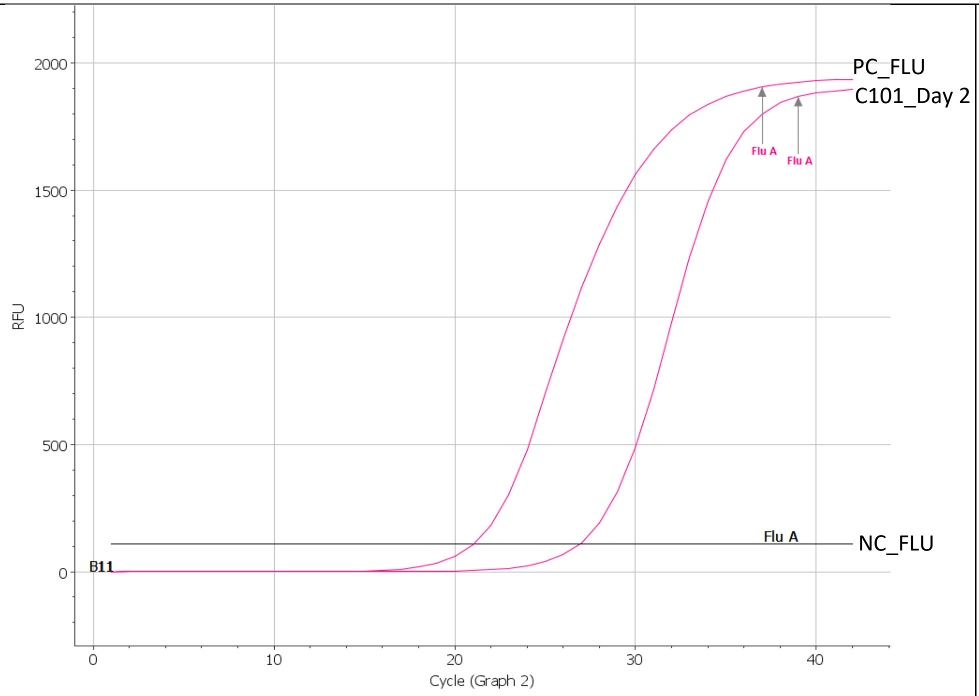

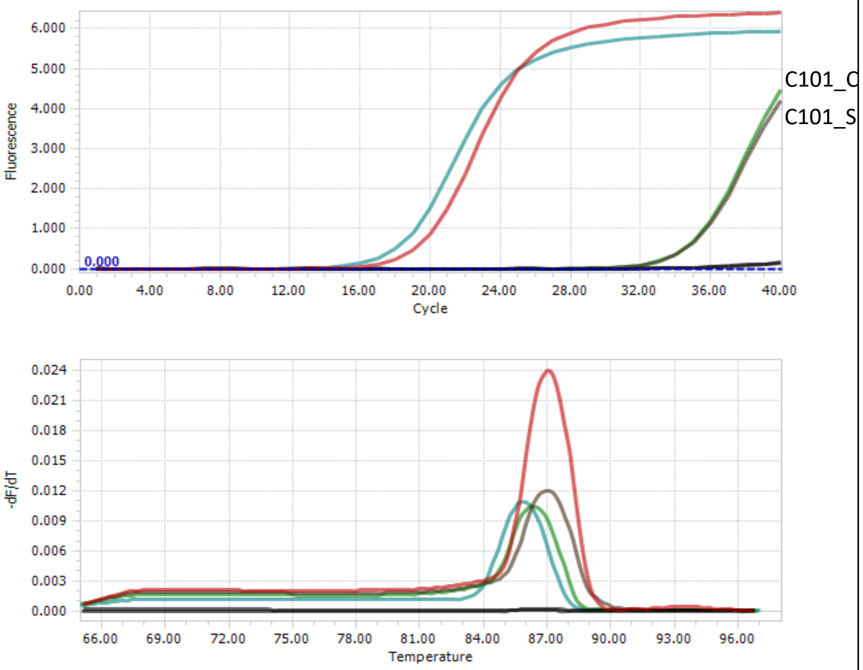

</td>
</tr>
</table>

85	C102_D0	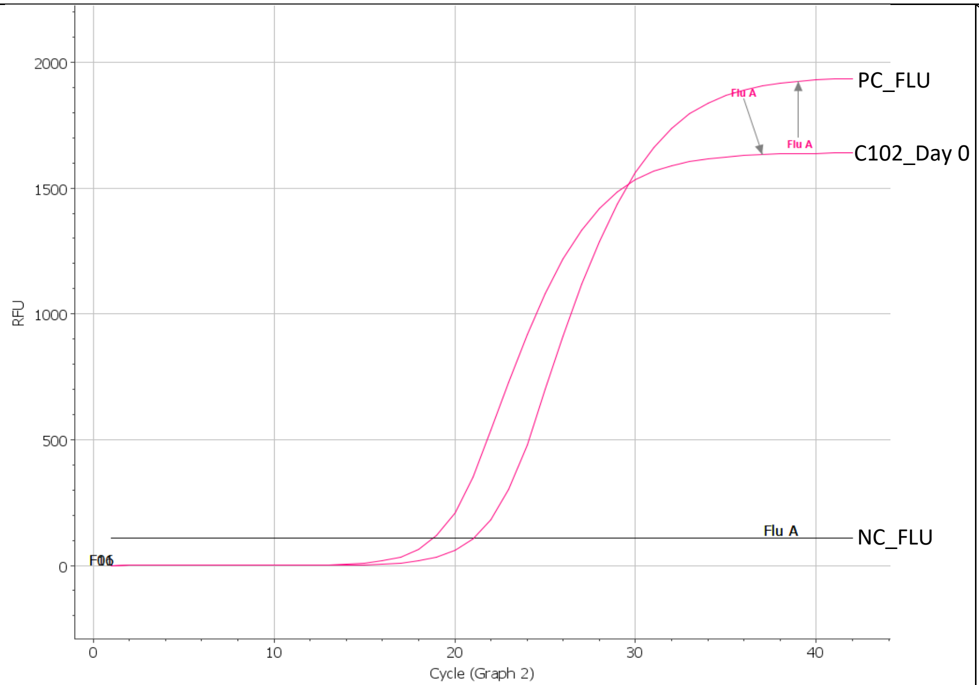	

PC_FLU
C102_Day 0
NC_FLU
Flu A
RFU
2000
1500
1000
500
0
10
20
30
40
Cycle (Graph 2)

<table>
<tr>
<td></td>
<td>C102_D
2</td>
<td>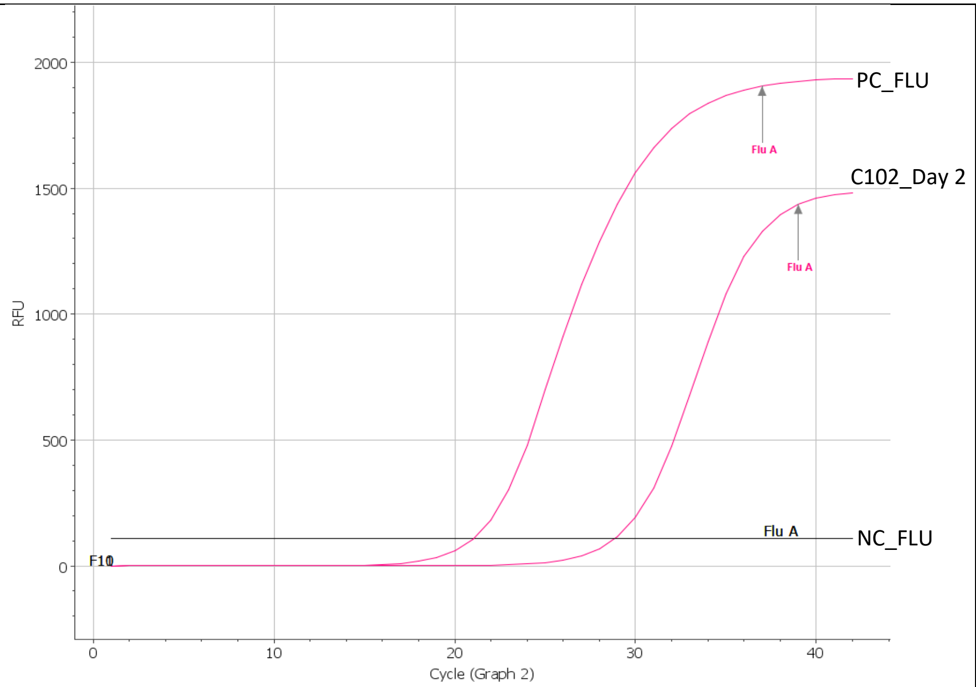
</td>
<td>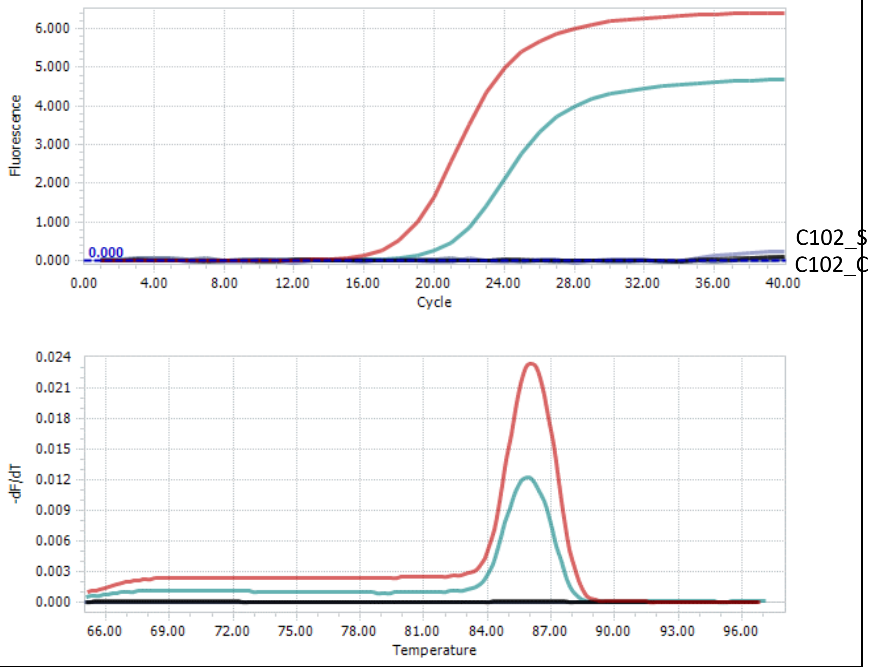
</td>
</tr>
</table>

86	C103_D0	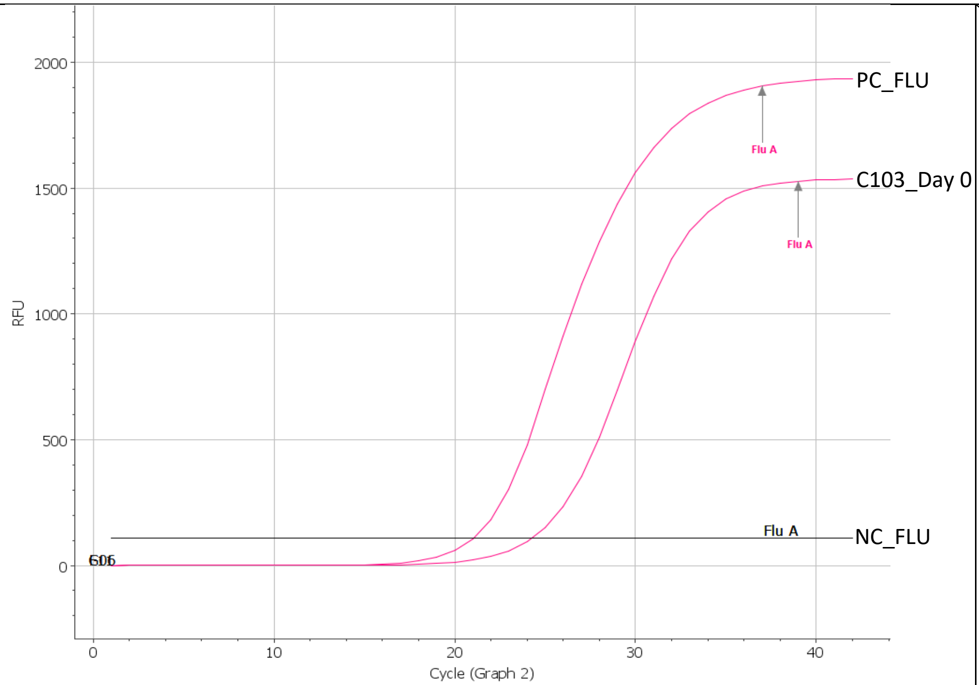RFU<br>2000<br>1500<br>1000<br>500<br>0<br>0<br>10<br>20<br>30<br>40<br>Cycle (Graph 2)<br>PC_FLU<br>Flu A<br>C103_Day 0<br>Flu A<br>Flu A<br>NC_FLU	

	C103_D 2	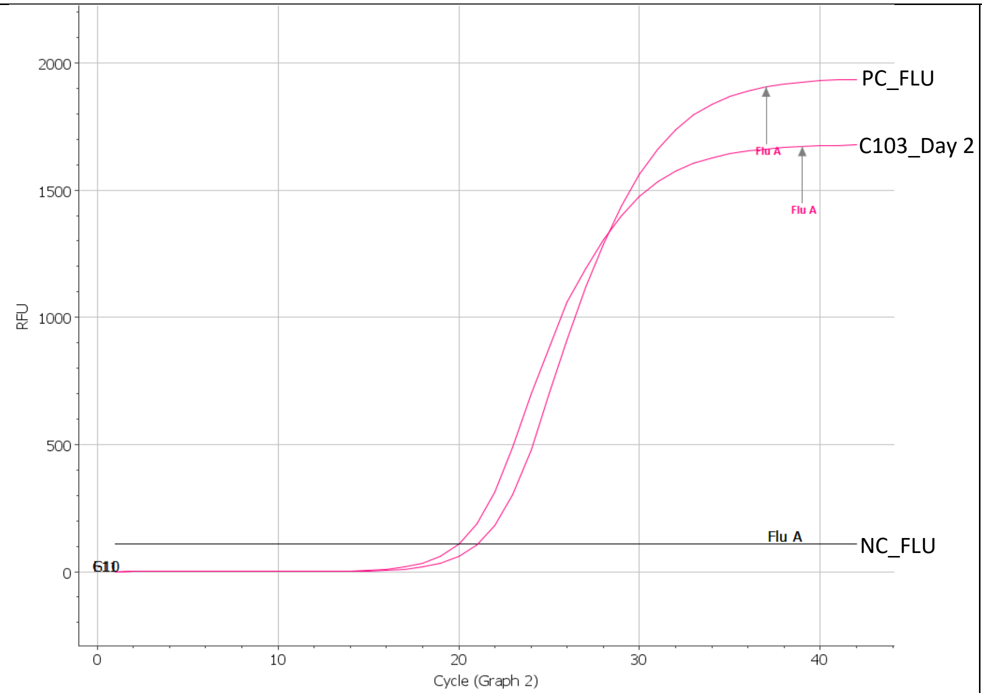	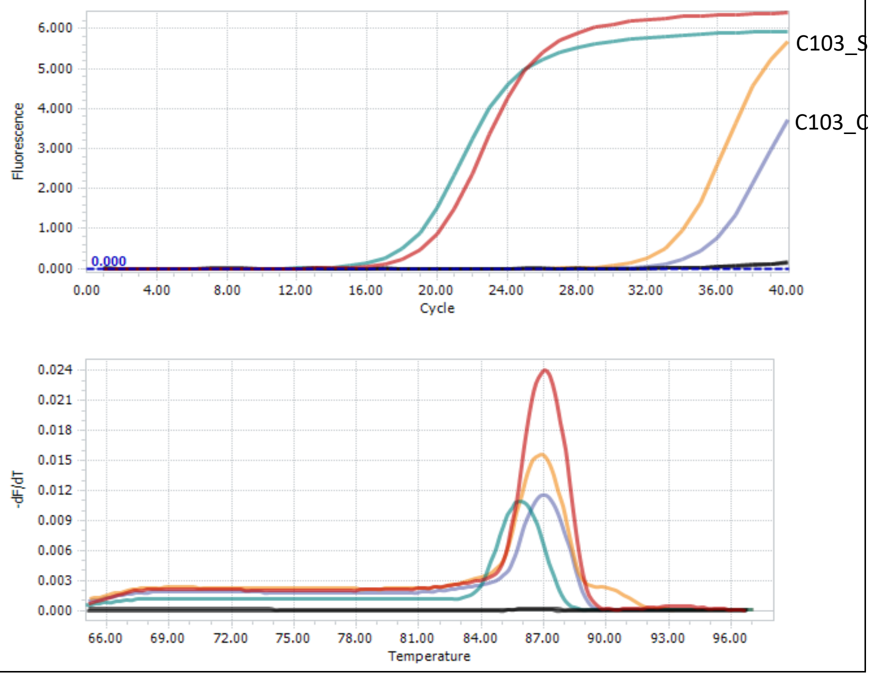

PC_FLU
C103_Day 2
NC_FLU
Flu A
RFU
Cycle (Graph 2)

C103_S
C103_C
Fluorescence
Cycle
-dF/dT
Temperature

87	C104_D0	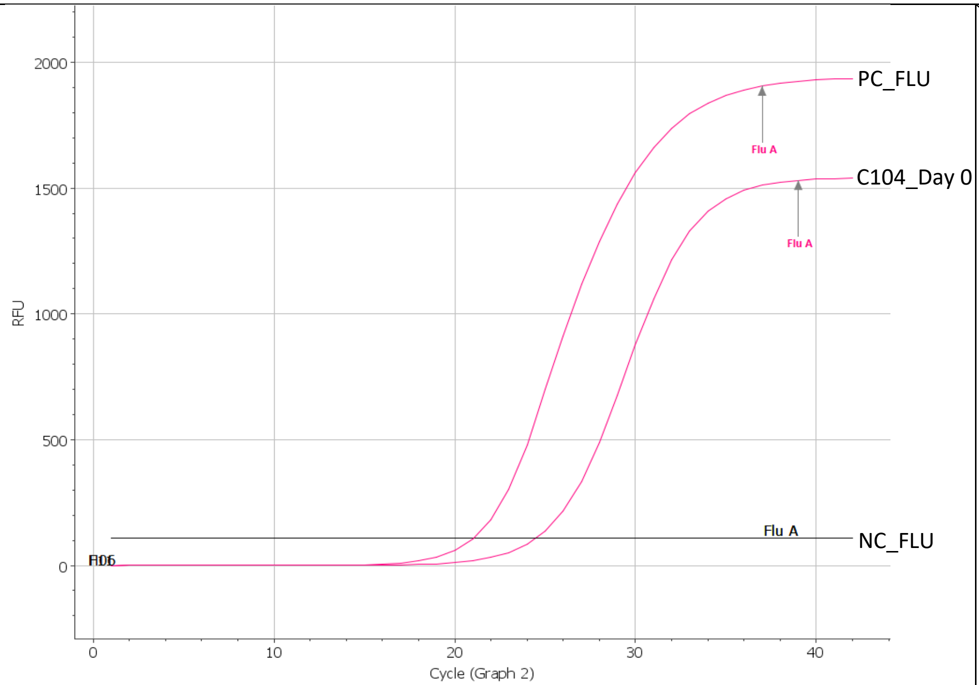PC_FLU<br>C104_Day 0<br>NC_FLU<br>Flu A<br>Flu A<br>Flu A<br>RFU<br>Cycle (Graph 2)<br>2000<br>1500<br>1000<br>500<br>0<br>0<br>10<br>20<br>30<br>40	

<table>
<tr>
<td></td>
<td>C104_D
2</td>
<td>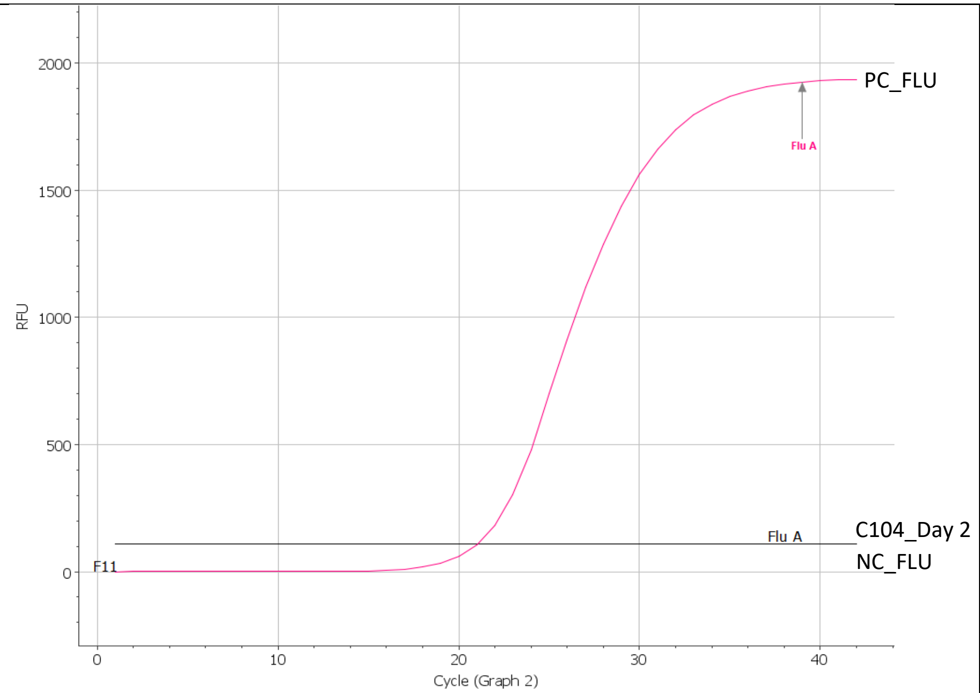
</td>
<td>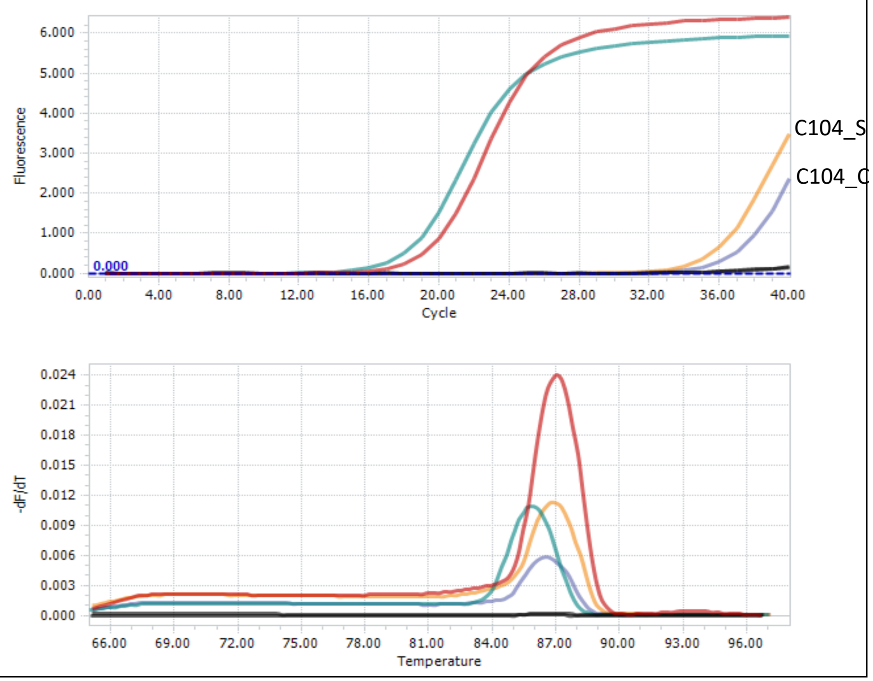
</td>
</tr>
</table>

88	C105_D0	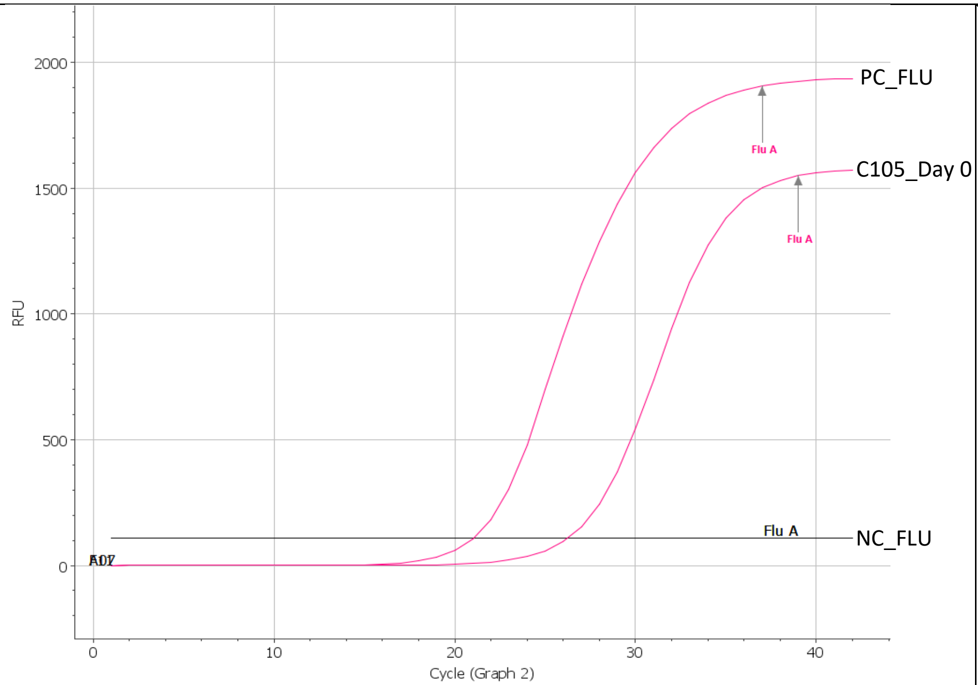2000<br>1500<br>1000<br>500<br>0<br>RFU<br>0<br>10<br>20<br>30<br>40<br>Cycle (Graph 2)<br>Flu A<br>Flu A<br>Flu A<br>PC_FLU<br>C105_Day 0<br>NC_FLU	

	C105_D<br>2	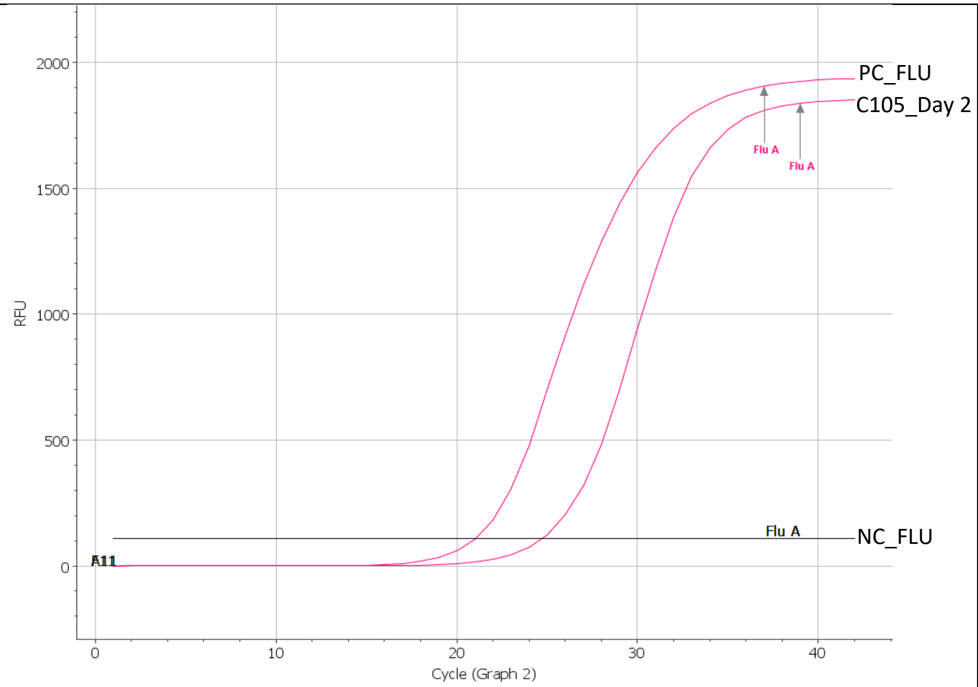RFU<br>2000<br>1500<br>1000<br>500<br>0<br>0 10 20 30 40<br>Cycle (Graph 2)<br>PC_FLU<br>C105_Day 2<br>NC_FLU<br>Flu A<br>Flu A<br>Flu A<br>A11	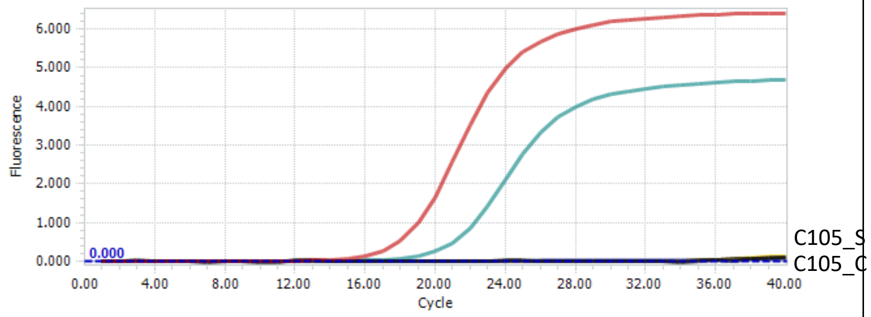Fluorescence<br>6.000<br>5.000<br>4.000<br>3.000<br>2.000<br>1.000<br>0.000<br>0.000<br>0.00 4.00 8.00 12.00 16.00 20.00 24.00 28.00 32.00 36.00 40.00<br>Cycle<br>C105_S<br>C105_C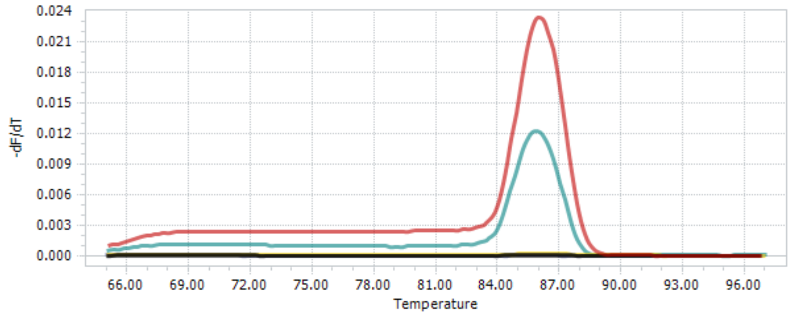-dF/dT<br>0.024<br>0.021<br>0.018<br>0.015<br>0.012<br>0.009<br>0.006<br>0.003<br>0.000<br>66.00 69.00 72.00 75.00 78.00 81.00 84.00 87.00 90.00 93.00 96.00<br>Temperature

89	C106_D 0	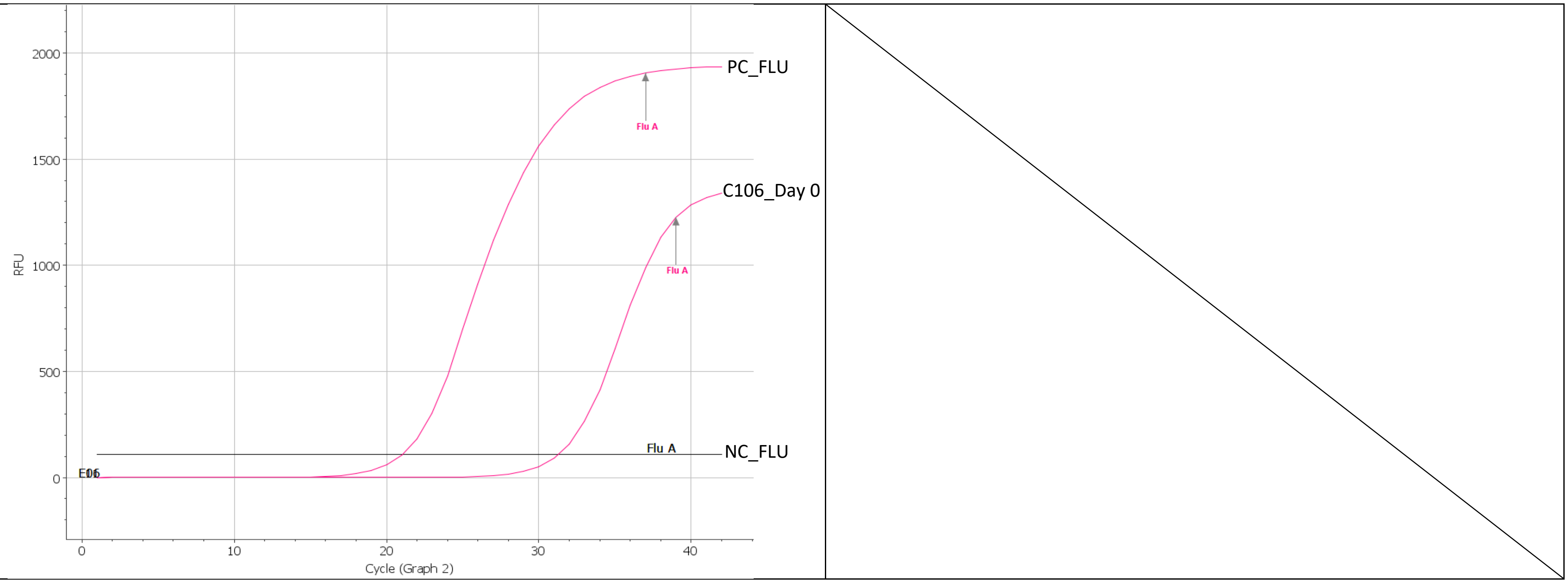PC_FLU
Flu A
C106_Day 0
Flu A
Flu A
NC_FLU
E06
RFU
2000
1500
1000
500
0
0
10
20
30
40
Cycle (Graph 2)	

<table>
<tr>
<td></td>
<td>C106_D
2</td>
<td>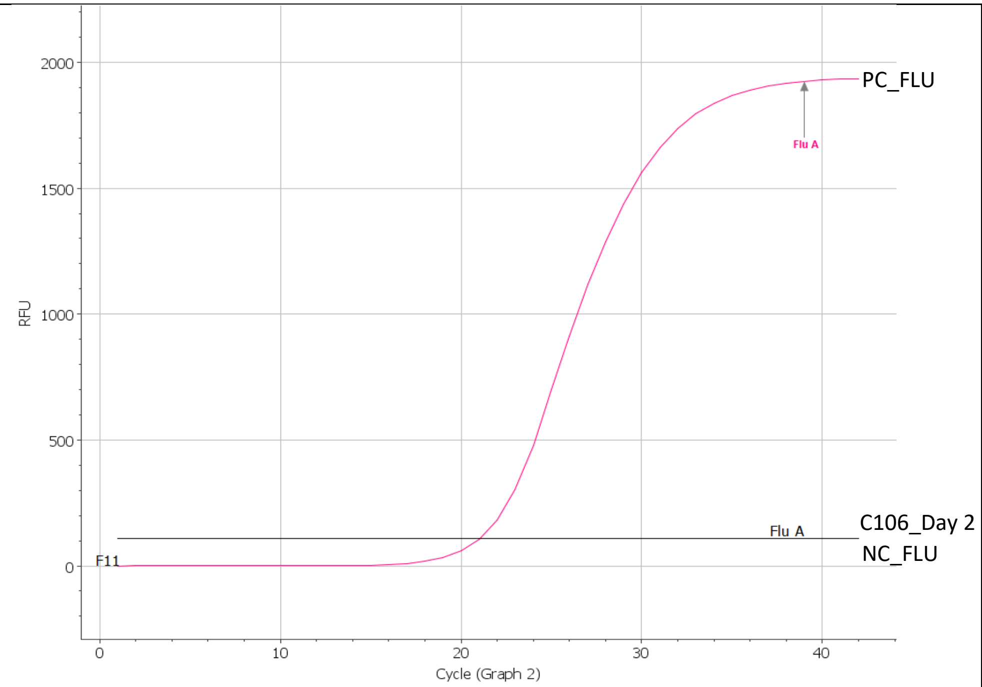
</td>
<td>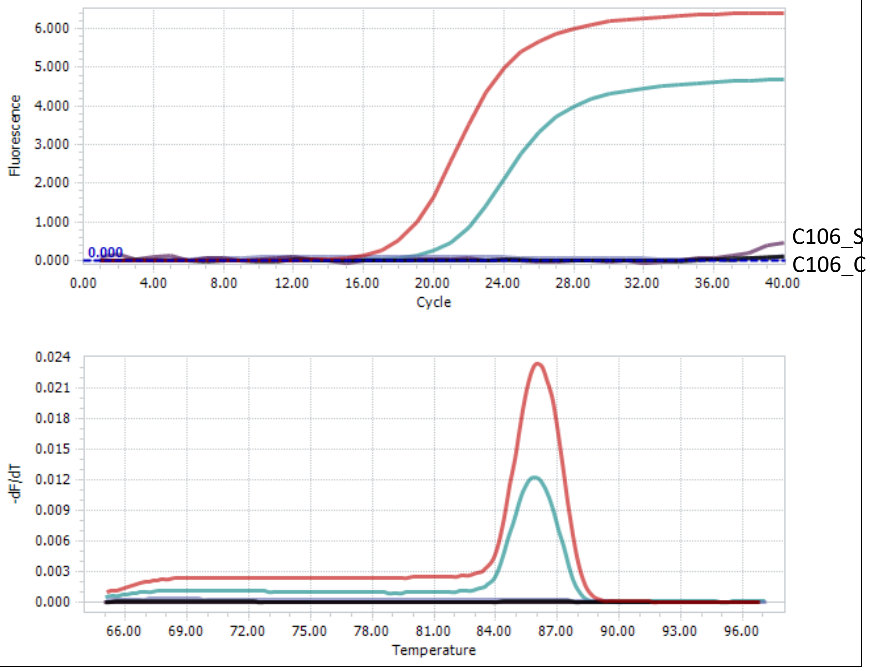
</td>
</tr>
</table>

90	C107_D 0	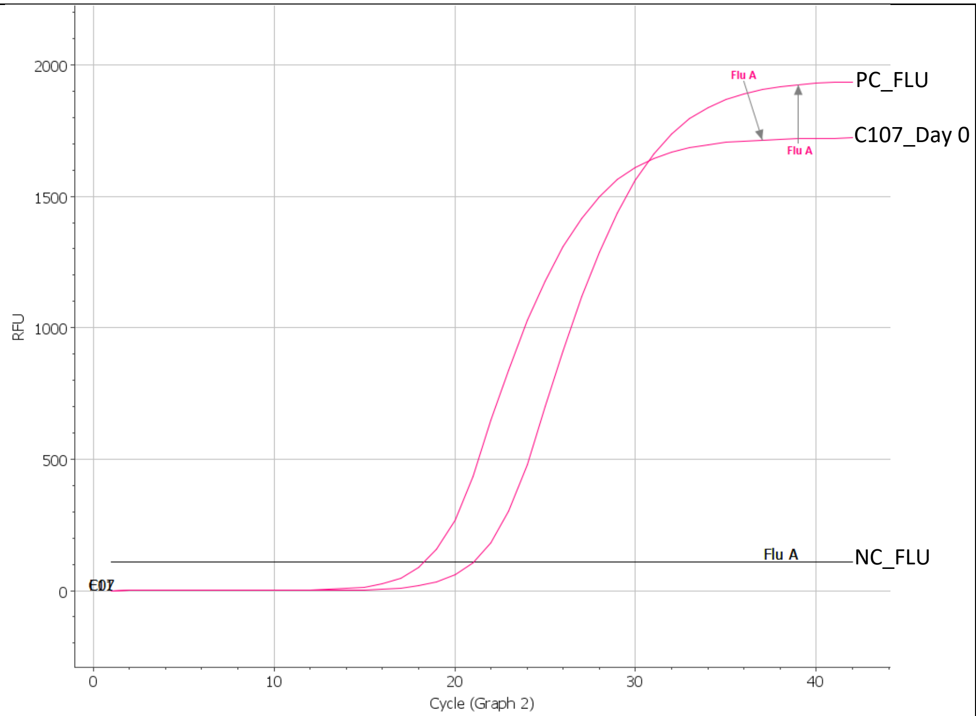2000
1500
1000
500
0
RFU
0
10
20
30
40
Cycle (Graph 2)
Flu A
PC_FLU
C107_Day 0
Flu A
NC_FLU	

C107_D 2

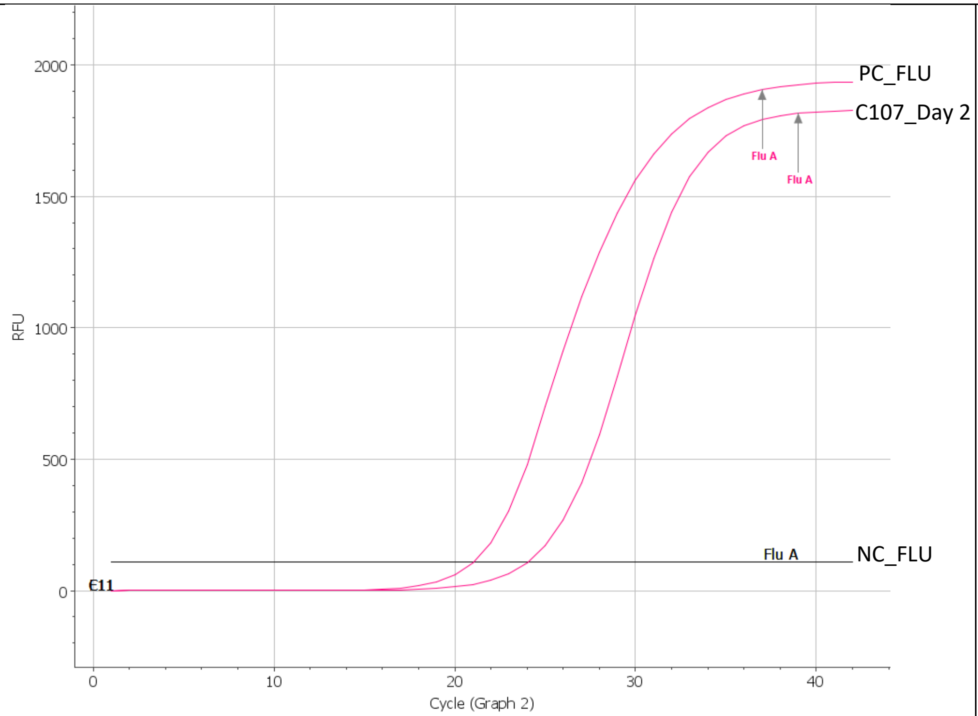

PC_FLU
C107_Day 2
Flu A
Flu A
Flu A
NC_FLU
C11
RFU
2000
1500
1000
500
0
0
10
20
30
40
Cycle (Graph 2)

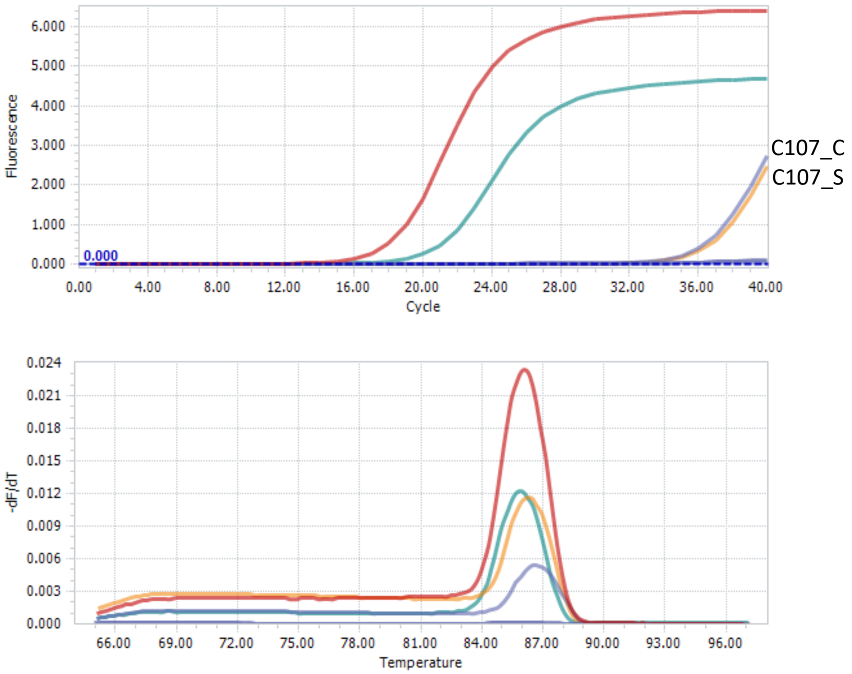

Fluorescence
6.000
5.000
4.000
3.000
2.000
1.000
0.000
0.000
0.00
4.00
8.00
12.00
16.00
20.00
24.00
28.00
32.00
36.00
40.00
Cycle
C107_C
C107_S
-dF/dT
0.024
0.021
0.018
0.015
0.012
0.009
0.006
0.003
0.000
66.00
69.00
72.00
75.00
78.00
81.00
84.00
87.00
90.00
93.00
96.00
Temperature

91	C108_D 0	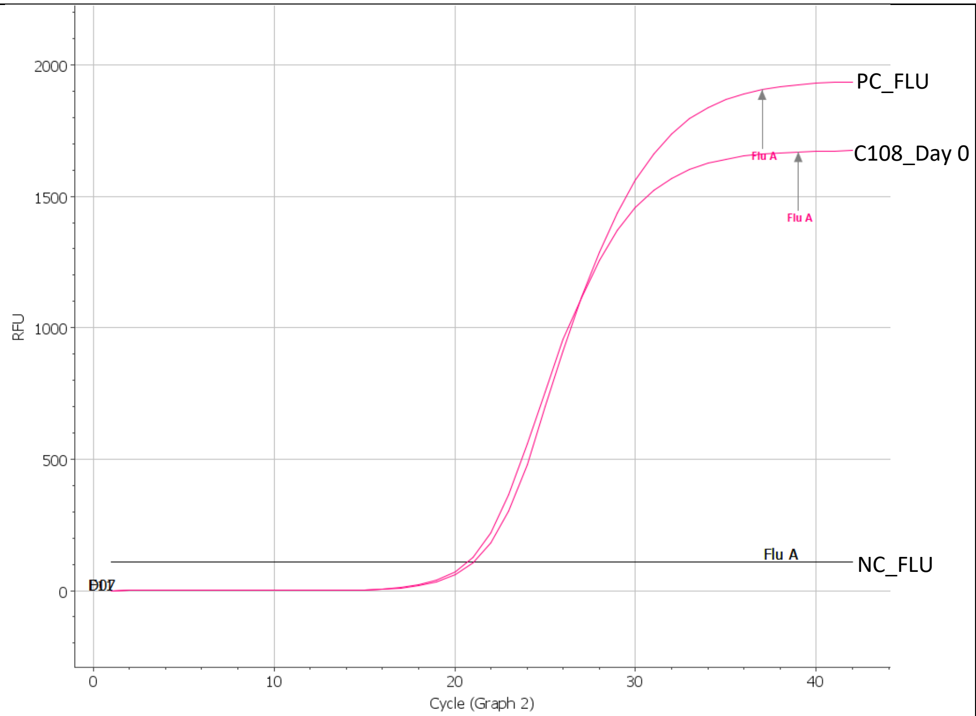RFU
2000
1500
1000
500
0
0
10
20
30
40
Cycle (Graph 2)
PC_FLU
C108_Day 0
Flu A
Flu A
Flu A
NC_FLU	

C108_D
2

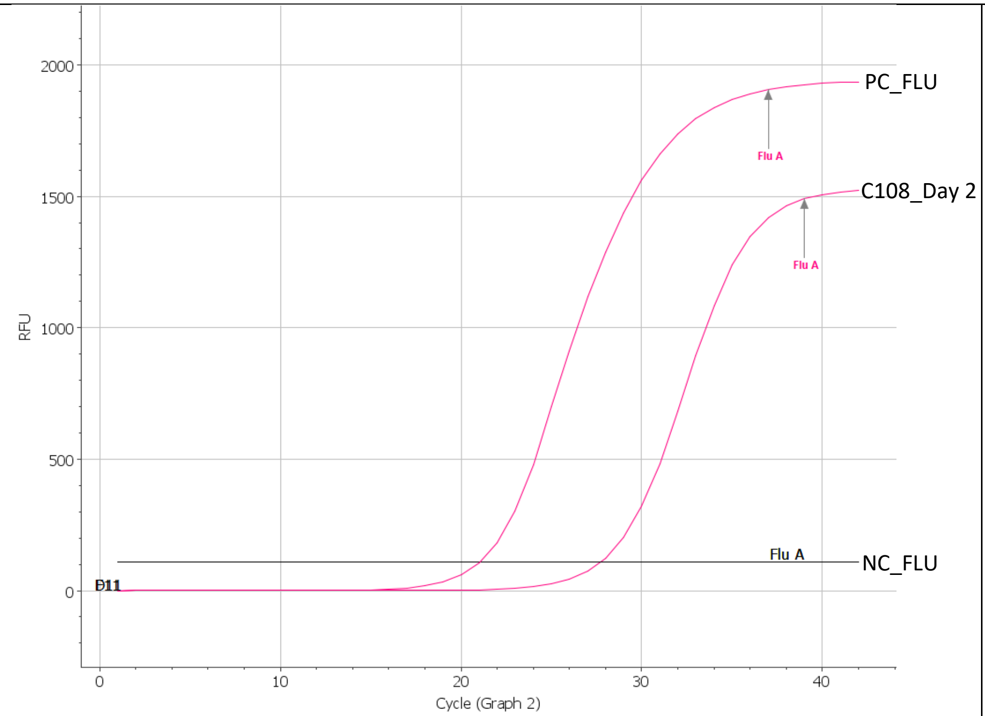

PC_FLU
C108_Day 2
NC_FLU
Flu A
Flu A
Flu A
D11
RFU
2000
1500
1000
500
0
0
10
20
30
40
Cycle (Graph 2)

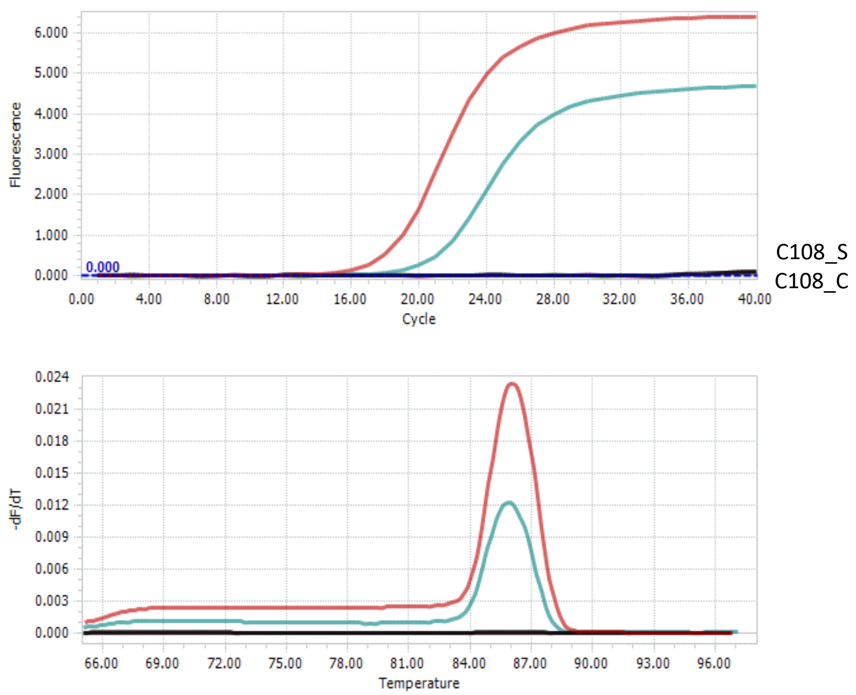

Fluorescence
6.000
5.000
4.000
3.000
2.000
1.000
0.000
0.000
0.00
4.00
8.00
12.00
16.00
20.00
24.00
28.00
32.00
36.00
40.00
Cycle
C108_S
C108_C
-dF/dT
0.024
0.021
0.018
0.015
0.012
0.009
0.006
0.003
0.000
66.00
69.00
72.00
75.00
78.00
81.00
84.00
87.00
90.00
93.00
96.00
Temperature

92	C109_D0	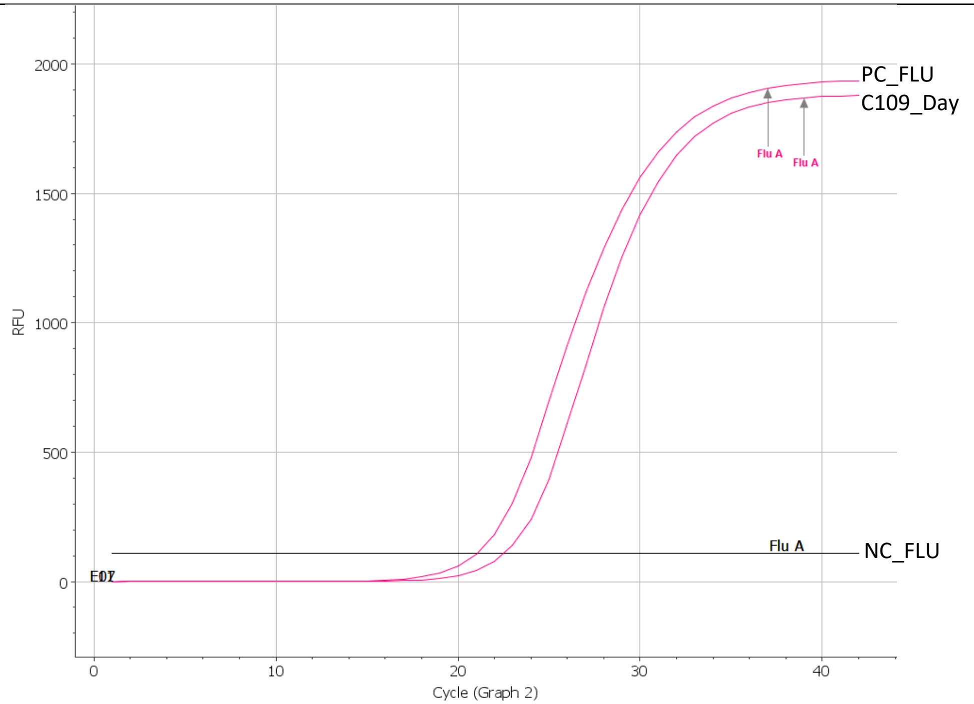 2000<br>1500<br>1000<br>500<br>0<br>RFU<br>0<br>10<br>20<br>30<br>40<br>Cycle (Graph 2)<br>PC_FLU<br>C109_Day<br>Flu A<br>Flu A<br>Flu A<br>NC_FLU	

C109_D 2

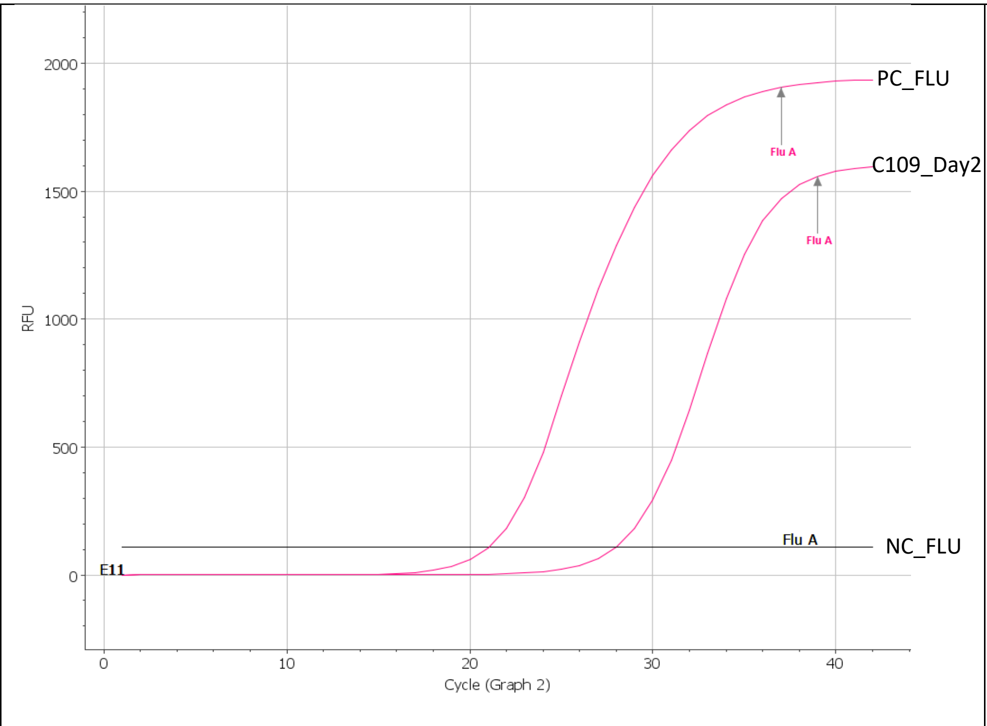

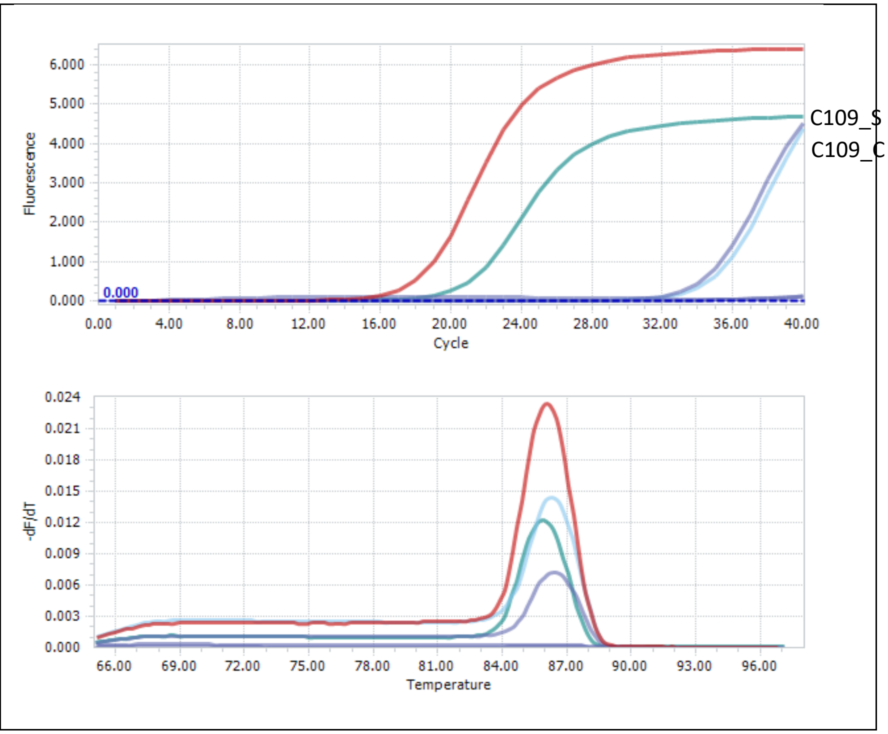

Fig. S1. A) Real-time PCR TaqMan probes amplification curves specifically for influenza virus taken from nasopharyngeal samples of the Control and Navax groups at day 0 and 2 of treatment. PC, NC are positive and negative controls of influenza virus; **B)** SYBR Green real-time PCR amplification curves specifically for *B. subtilis* and *B. clausii* taken from nasopharyngeal samples of Navax group at day 2 of treatment. PC, NC are positive and negative controls of *B. subtilis* and *B. clausii*. By lottery, the patient's number is coded at random. The sequence of the image's appearance corresponds to the order of the patient's hospitalization.
