# Supplementary material for: Efficient symptomatic treatment and viral load reduction for children with influenza virus infection by nasal-spraying Bacillus spore probiotics
Source: Sci Rep. 2023 Sep 8;13:14789. doi: 10.1038/s41598-023-41763-5 (PMC10491672; doi:10.1038/s41598-023-41763-5)
Supplement: Supplementary file 2 — Supplementary Information. [file 41598_2023_41763_MOESM2_ESM.pdf]

Table S1. Primers and probes used for specific amplification of influenza virus by real-time RT-PCR Taqman probe and for specific amplification of *B. subtilis* and *B. clausii* by real- time PCR SYBR Green

**Primer/Probe name**	**Sequence (5' - 3')**	**Target gene or region**	**Reference***	**Concentration (nM)**
1. Influenza viruses				
InfA-Fw	GAC CRA TCC TGT CAC CTC TGA C	Matrix protein	[39, 41]	200
InfA-Rv	AGG GCA TTY TGG ACA AAK CGT CTA			200
InfA-FAM probe	/56FAM/-TGC AGT CCT CGC TCA CTG GGC ACG-(BHQ1)			40
InfB-Fw	TCC TCA ACT CAC TCT TCG AGC G	Nonstructural protein	[40, 41]	200
InfB-Rv	CGG TGC TCT TGA CCA AAT TGG			200
InfB-JOE probe	(JOE)-CCA ATT CGA GCA GCT GAA ACT GCG GTG-(BHQ1)			40
RnaseP-Fw	AGA TTT GGA CCT GCG AGC G	Ribonuclease P	[39, 40]	200
RnaseP-Rv	GAG CGG CTG TCT CCA CAA GT			200
RnaseP-Cy5 probe	Cy5/TTC TGA CCT GAA GGC TCT GCG CG/3IABkFQ/			40
2. Bacillus sp.				
subtilis-Fw	ACC ATT GCG GTA GGT GCG	aprE	[42]	250
subtilis-Rv	GCG TTT GTC CAA GTC GGG			250
clausii-Fw	AAT TTT TAC CGC CCC TCA AG	erm34	[43]	250
clausii-Rv	AC TTT TGG AAC ATG CCG AAC			250

References:

[39]Fan, J. *et al.* Detection of a novel avian influenza A (H7N9) virus in humans by multiplex one-step real-time RT-PCR assay. *BMC Infect Dis* **14**, 1-9 (2014).

[40]Shu, B. *et al.* Multiplex real-time reverse transcription PCR for influenza A virus, influenza B virus, and severe acute respiratory syndrome coronavirus 2. *Emerg Infect Dis* **27**, 1821-1830 (2021).

[41]WHO Global Influenza Surveillance Network. Manual for the laboratory diagnosis and virological surveillance of influenza. *World Health Organization* https://apps.who.int/iris/handle/10665/44518 (2011).

[42]Sadeghi, A., Mortazavi, S. A., Bahrami, A. R., Sadeghi, B., & Matin, M. M. Designing a SYBR Green absolute real time PCR assay for specific detection and quantification of *Bacillus subtilis* in dough used for bread making. *JCMR* **6**, 83-92. (2014).

[43]Perotti, M. *et al.* Quantitation of *Bacillus clausii* in biological samples by real-time polymerase chain reaction. *J Microbiol Methods* **65**, 632-636 (2006).
